# Bayesian Optimization over Multiple Experimental Fidelities Accelerates Automated Discovery of Drug Molecules

**DOI:** 10.1021/acscentsci.4c01991

**Published:** 2025-02-05

**Authors:** Matthew A. McDonald, Brent A. Koscher, Richard B. Canty, Jason Zhang, Angelina Ning, Klavs F. Jensen

**Affiliations:** † Massachusetts Institute of Technology, Department of Chemical Engineering, 77 Massachusetts Avenue, Cambridge, Massachusetts 02139, United States; ‡ Drexel University, Department of Chemical and Biological Engineering, 3101 Ludlow St, Philadelphia, Pennsylvania 19104, United States

## Abstract

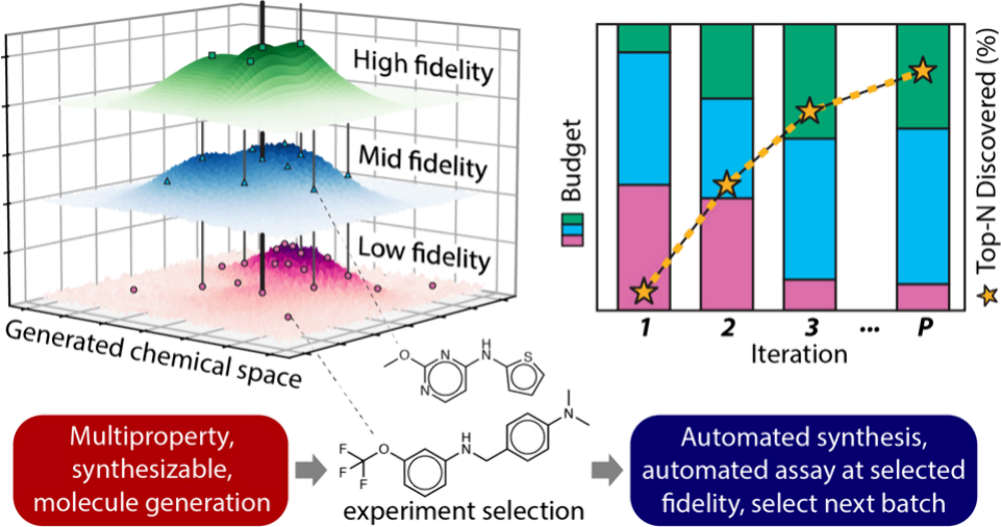

Different experiments of differing fidelities are commonly used in the search for new drug molecules. In classic experimental funnels, libraries of molecules undergo sequential rounds of virtual, coarse, and refined experimental screenings, with each level balanced between the cost of experiments and the number of molecules screened. Bayesian optimization offers an alternative approach, using iterative experiments to locate optimal molecules with fewer experiments than large-scale screening, but without the ability to weigh the costs and benefits of different types of experiments. In this work, we combine the multifidelity approach of the experimental funnel with Bayesian optimization to search for drug molecules iteratively, taking full advantage of different types of experiments, their costs, and the quality of the data they produce. We first demonstrate the utility of the multifidelity Bayesian optimization (MF-BO) approach on a series of drug targets with data reported in ChEMBL, emphasizing what properties of the chemical search space result in substantial acceleration with MF-BO. Then we integrate the MF-BO experiment selection algorithm into an autonomous molecular discovery platform to illustrate the prospective search for new histone deacetylase inhibitors using docking scores, single-point percent inhibitions, and dose–response IC_50_ values as low-, medium-, and high-fidelity experiments. A chemical search space with appropriate diversity and fidelity correlation for use with MF-BO was constructed with a genetic generative algorithm. The MF-BO integrated platform then docked more than 3,500 molecules, automatically synthesized and screened more than 120 molecules for percent inhibition, and selected a handful of molecules for manual evaluation at the highest fidelity. Many of the molecules screened have never been reported in any capacity. At the end of the search, several new histone deacetylase inhibitors were found with submicromolar inhibition, free of problematic hydroxamate moieties that constrain the use of current inhibitors.

## Abstract 2

Potential new drug molecules are automatically generated, synthesized, and tested for potency with assays of differing fidelities in each iteration of a batched Bayesian optimization campaign.

Small molecule pharmaceutical discovery campaigns are often organized as an experimental funnel. Such a campaign begins with rapid, low-cost assays or computations to screen large numbers of molecules, followed by progressively higher fidelity, more expensive assays with correspondingly smaller throughput to maintain a balance between scale and accuracy.^1^ Along the way, measurements of pharmacological requirements may be inserted into the funnel at the appropriate level of experimental cost. Moving from low to high-fidelity experiments, a subset of previously screened molecules is often rescreened at the next assay level, perhaps with some changes and additions as determined by experienced medicinal chemists. Recently, transfer learning methods starting from low-fidelity experiments, such as single-point assays, to predict the outcome of high-fidelity experiments, such as dose–response curves, have been developed, but they lack generality, and different methods best suit different drug targets.^2^

In contrast to the linear trajectory of the experimental funnel, iterative design of experiments enables optimal selection of the next set of experiments through feedback from each iteration (cf. Figure 1A).^3^ Iterative methods typically require screening fewer compounds to find high-performing molecules. However, current methods typically only make use of a single level of experimental fidelity, often the highest and most expensive fidelity.^4^ Bayesian optimization has proved to be a particularly powerful iterative method with general applicability to many optimization problems.^5−7^ Integrating the multifidelity (MF) approach of the experimental funnel into Bayesian optimization could enable more compounds to be screened or fewer resources to be used in each iteration while maintaining optimal selection of experiments and rapid identification of promising drug compounds.

**Figure 1:**
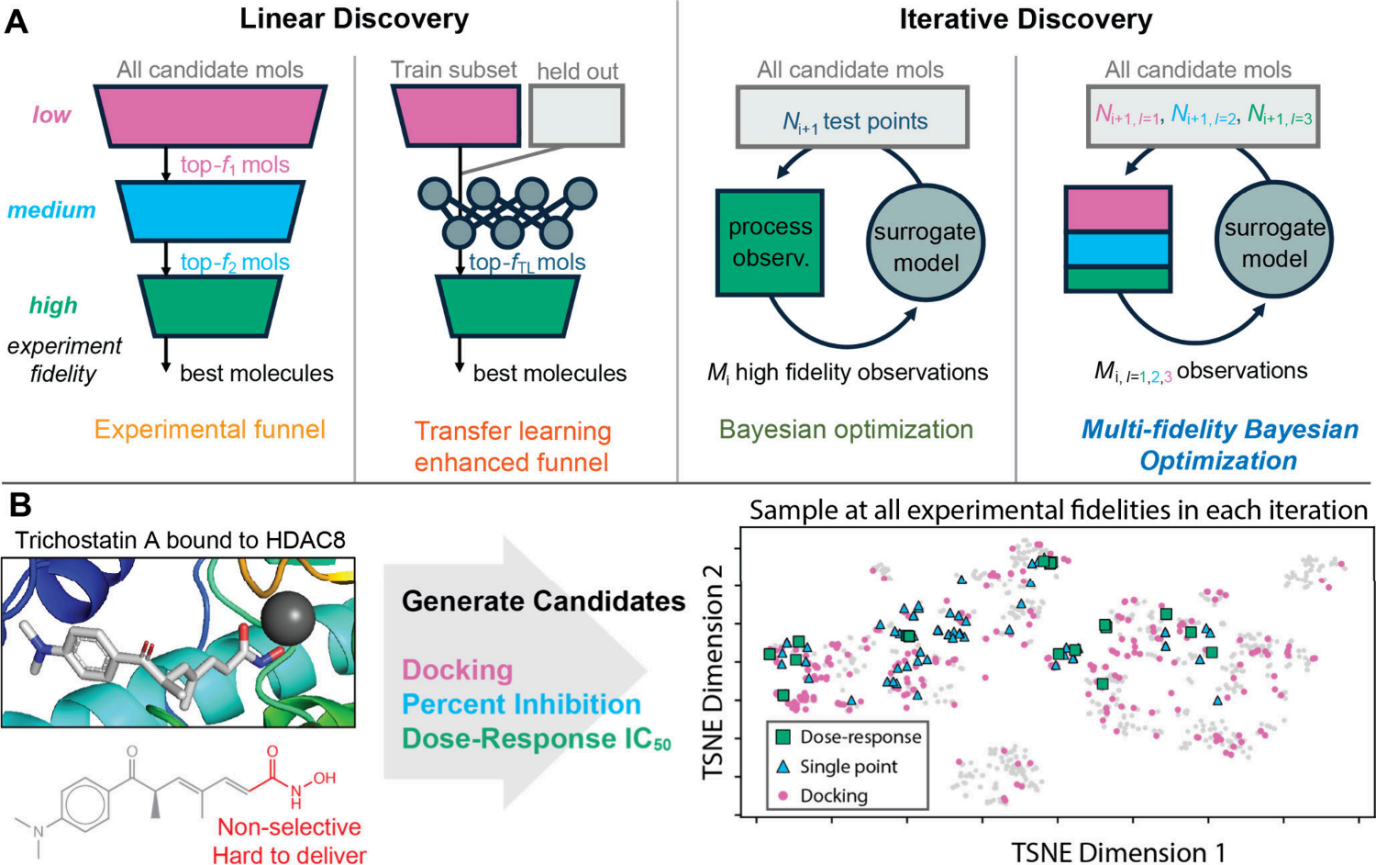
(A) A comparison of different types of design of experiments in small molecule drug discovery, divided into linear designs and iterative designs. (B) Application of MF-BO to the prospective discovery of new HDACIs. Shown at left is the crystal structure of trichostatin A bound to human HDAC8. The hydroxamic acid moiety (red) makes current HDACIs hard to deliver and dose safely. Generated molecules can be tested at three levels: docking, which is weakly correlated to potency; single-point percent inhibition, which is moderately correlated to potency; and dose–response measurement, which is a direct measurement of potency. The t-distributed stochastic neighbor embedding (TSNE) plot shows the selection of experiments of different fidelities over the generated chemical space in a single experimental iteration.

Several challenges have limited development of multifidelity Bayesian optimization in molecular discovery. Unknown degrees of correlation between experiment levels and uncertainty about the cost of experiment levels have led to the greatest advancements being made in purely computational settings.^8^ For example, in finite element models, the costs and error bounds of simulations using different numbers of grid points are well-known, and multiple means of iterative multifidelity optimization have been published.^9,10^ In materials chemistry, multifidelity optimization approaches have been demonstrated for electronic structure calculations with functionals of different costs,^11^ for organic framework discovery using molecular simulations of different costs,^12^ and for a co-orchestration approach to coordinate multiple characterization instruments to optimize a material but without making a distinction between assay costs.^13^ In all examples, optimization occurred sample-by-sample and was performed in continuous search spaces, as opposed to the typical batched assays and discrete search space of small molecule drugs.

In this work, we present a multifidelity Bayesian optimization (MF-BO) iterative discovery algorithm for molecular property optimization. First, we demonstrate the superiority of MF-BO by applying the algorithm to a variety of literature data sets, where it outperforms an experimental funnel (EF), transfer learning with low-fidelity data (TL), and Bayesian optimization exclusively with high-fidelity data (BO), as measured by the rate of (re)discovery of top-performing molecules. Then, we integrate MF-BO into an autonomous chemical synthesis and testing platform to search for new histone deacetylase inhibitors (HDACIs) automatically. Figure 1A breaks down how experiments are typically allocated using these different methods. Low fidelity measurements are cheap experiments and poor predictors of the ground truth while high fidelity measurements reflect the ground truth but at high experimental cost. In this study, docking is always used as the low fidelity experiment (represented as pink data points in figures), single point inhibition measurements are used as the medium fidelity experiment (blue), and dose–response curves represent the high fidelity experiment (green). DiffDock, a machine learning docking algorithm,^14^ was used for docking experiments because it outperformed traditional docking programs in terms of both speed and accuracy for our targets (see the Supporting Information (SI) for a comparison to Autodock Vina).

To facilitate the discovery of HDACIs, we use a genetic algorithm based on reaction templates known to be executable by the autonomous platform. Histone deacetylase plays a prominent role in regulating gene expression and epigenetics, and HDAC inhibition is used to treat a number of diseases, including neurodegenerative diseases and some cancers.^15−17^ Most HDACIs are hydroxamic acid derivatives, which are potent but difficult to deliver and target, making new modes of inhibition desired.^18,19^ The platform combines the genetic algorithm to generate diverse candidate molecules (Figure 1B), the MF-BO algorithm to select candidates and the optimal level of fidelity at which to evaluate the candidate, and computer-aided synthesis planning (CASP) tools to plan synthesis execution. Physically, the platform brings together a liquid handler, high-performance liquid chromatography with mass spectrometry (HPLC-MS) with fraction collection, plate reader, robot arm, and custom-made reactors to execute planned experiments automatically. When experiments at the highest fidelity are selected, the molecules are manually synthesized and analyzed with NMR to ensure their identities, concentrations, purities, and activities are accurate. Since MF-BO is iterative, the platform automatically chooses a subsequent batch of experiments once the previous batch is complete.

## Results and Discussion

### Part 1: Evaluation of MF-BO

The concept of allocating limited resources to tasks of differing cost and fidelity to build surrogate models of complex processes was originally developed for simulations, for which the problem is typically constrained to running single experiments in series with known correlation between fidelities. Several heuristics to solve this challenge have been published, including targeted variance reduction (TVR),^11^ which was originally developed for simulation-based materials screening using a 20-dimensional material representation. In this work, we extend TVR for use in molecular discovery in a batched setting, where the correlation between fidelities is both unknown and varies over chemical space.

We examined three surrogate model architectures, random forest, natural gradient boosting, and Gaussian process (GP), and three molecular representations, mol2vec,^20^ Mordred descriptors,^21^ and Morgan fingerprints (radius 2, 1024 bit).^22^ The combination of Gaussian processes with Morgan fingerprints, using a Tanimoto Kernel,^23^ performed best; a detailed comparison and discussion of methods can be found in the SI. A Monte Carlo approach was used to select batches of molecule-fidelity pairs. Like in Kriging methods, a molecule-fidelity pair was selected based on maximum expected improvement (EI), then multiple estimations were sampled using the mean and variance of the GP at the selected pair, and multiple new surrogates were regressed based on each sample. Using each new surrogate model, new pairs were selected, new estimates were sampled, and new models were trained; combinations of sampled pairs with poor total EI were pruned to prevent the number of models from exploding. This process continued until the budget was exhausted. While the poor scaling of GPs, along with the dimensionality of Morgan fingerprints and size of the search space, made iterative regression of new GP models slow, experiment execution is slower, so we chose to forego additional optimization such as implementation of scalable GPs^24^ and GPs for sparse inputs.^25^

The per-iteration budget was determined based on a desired throughput of one autonomous iteration per week. We had previously synthesized approximately 100 molecules per week for single-point assays,^26^ and we estimated we could run 1,000 docking simulations and manually conduct 10 dose–response curve assays per week. The relative cost of each fidelity was set at 0.01, 0.2, and 1.0, for docking, single-point, and dose–response assays, respectively, with a per iteration budget of 10.0. These costs assumed that the primary cost associated with each assay is time, and that all assays of the same type require the same amount of time. For scenarios where materials contribute significantly to cost, experiments could be priced individually based on material needs (or a combination of materials and time) without any change to the algorithm. The Monte Carlo approach to batching also does not have a holistic view of experiment selection and may fail to select molecules with synergistic synthesis routes, as demonstrated in recent work in the single-fidelity setting.^27^

In a multifidelity setting using TVR, the surrogate model predicts a mean and variance for each fidelity; each mean is scaled from 0 to 1 and each variance is scaled to the inverse cost of the fidelity. The expected improvement acquisition function selects the molecule-experiment pair that maximizes the expected value of the improvement of the molecule’s performance at the highest fidelity measurement. Intuitively, this means low-cost samples are drawn more often because their higher variance requires several measurements to decrease the expected improvement to the scale of higher fidelity samples. For some molecules, scores on each assay will be similar, for other molecules each assay will paint a different picture. The surrogate model learns assay outcomes *and* correlation between assays from molecular structure.

**Figure 2:**
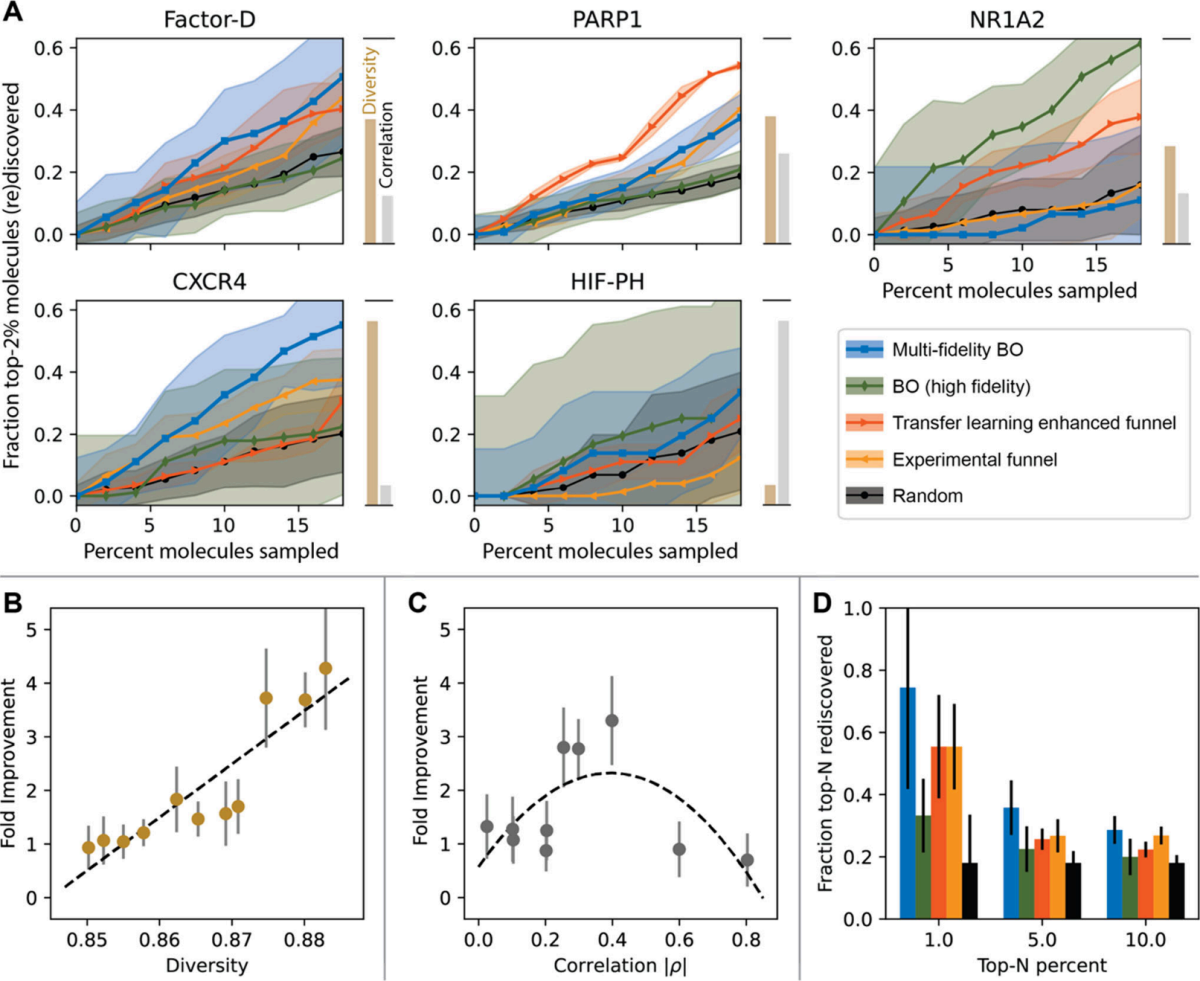
(A) Comparison of MF-BO against other designs of experiments. Markers indicate the mean fraction of top-2% molecules found after sampling the search space in discrete 2% batches. Shaded regions are the standard deviation from 6 repeats of each experiment. Blue ■ - MF-BO, green ◆ - BO, red ▶ - transfer learning, orange ◀ - experimental funnel, black ● - random selection. Gold and silver bars represent scaled diversity and correlation, respectively, within target data sets. (B, C) Enhancement of MF-BO over BO using structured data sets sampled from acetylcholinesterase inhibitors to probe the effect of diversity (B, gold) and fidelity correlation (C, silver) on MF-BO usefulness. Dashed curves are to guide the eye. (D) Assessment of different designs of experiments when rediscovering top-N% molecules in the Factor-D data set after sampling 18% of the data set. Colors are the same as in (A) and error bars represent the standard deviation of 6 repeats.

We evaluated the method on five precompiled data sets: IC_50_ values for each protein target were taken from ChEMBL;^28^ docking scores were computed using DiffDock;^14^ and single-point assay values (percent inhibition) were simulated from IC_50_ values with the Hill equation,^29^ as detailed in the SI. The MF-BO search was initialized by providing measurements at each fidelity for 5% of the molecules so the model could learn the relationship between fidelities. We measured performance as the cumulative (re)discovery of inhibitors in the top-N% of the data set. Figure 2A shows the rediscovery of molecules in the top-2% of inhibitors for complement factor D (n = 1263), chemokine receptor 4 (CXCR4, n = 891), poly[ADP-ribose] polymerase 1 (PARP1, n = 2530), hypoxia-inducible factor prolyl hydroxylase (HIF-PH, n = 358), and thyroid hormone receptor beta (NR1A2, n = 374), as well as the relative diversity and correlation between fidelities within each data set. Each iteration of the search had a budget equivalent to testing 2% of the molecules in the respective data set at the highest fidelity, this enabled comparison to both linear and iterative search methods. The variability between the six repeats of each heuristic, shown as the shaded regions in Figure 4A, stems from different initializations, where molecules excluding those in the top-N% were randomly sampled. For small data sets, the initial molecules chosen lead to large variations in performance; larger data sets like PARP1 are less sensitive to the initial sample.

The Factor-D and CXCR4 data sets show that for drug targets with diverse search spaces and weak-to-moderate correlation between experimental fidelities, MF-BO substantially outperforms the other discovery algorithms, yielding a roughly 30% improvement in the rate of (re)discovery for the Factor-D example compared to the next best method, (cf. Figure 2A). MF-BO can explore more of the search space without the cost incurred for exploration using Bayesian optimization on the highest fidelity alone. As expected, when the correlation between fidelities is strong, experimental funnel approaches (both traditional and transfer learning enhanced) perform best (PARP1): if the correlation were perfect, there would never be a need to do costly high-fidelity experiments as the low fidelity experiments perfectly predict the high-fidelity outcome. In the case of weak/no correlation between fidelities, Bayesian optimization on the highest fidelity alone is most efficient (this is especially true when the search space is not large or diverse, see NR1A2) since low fidelity experiments do not contain pertinent information.

To investigate these hypotheses about when MF-BO can be expected to outperform other discovery heuristics, we built subsets with tailored diversity and correlation from a sixth ChEMBL data set, acetylcholinesterase inhibition. The acetylcholinesterase data set (of nearly 10,000 molecules) was clustered into 25 clusters, and 1,000 molecules were either drawn from a small number of clusters to build a narrow search space or from all the clusters to build a diverse search space. The correlation between fidelities, measured as the absolute value of the multiple correlation coefficient, |ρ|, was controlled by weighting the selection of each molecule by its residual when regressing the relationship between fidelities. Figures 2B and 2C show that the trends seen across the five ChEMBL data sets are consistent with the trends of these structured subsets, measured as fold improvement compared to BO after 5 search iterations (10% of the space). Increasing diversity enables MF-BO to gather exploratory data at a low cost to avoid getting trapped in local minima. An optimal extent of correlation exists at which the algorithm can learn where (in chemical space) the information contained at each fidelity is useful. The improvement is more substantial when targeting higher ranked molecules; Figure 2D shows that MF-BO is able to find 70% of the top-1% of Factor-D inhibitors but only 30% of the top-10% of inhibitors.

The literature data sets are composed entirely of biologically active molecules, biasing the results in favor of methods that find the most potent molecules in a pool of active compounds. To test that MF-BO discovers potent molecules when nonactive compounds are included, data sets with decoy molecules were created for CXCR4, sampling property-matched decoys for CXCR4 from the DUDE-Z database.^30^ A docking score is still calculated for these decoys, but their activity is expected to be negligible in comparison to the actives from the ChEMBL CXCR4 set. When a quarter of the search space was composed of decoys, MF-BO rediscovered 37% of the top-2% of inhibitors. The number rediscovered stayed relatively constant at 38% when the fraction of decoys was increased to 50% (see Figure S3). These results assuage concerns that, in a prospective setting, the enhancement from MF-BO may be diminished by nonactives or biased docking scores. They also show that biases from docking do not mislead iterative experimental designs and that learning when to trust docking scores provides a substantial enrichment.

### Part 2: Prospective Application of MF-BO to Histone Deacetylase Inhibition

**Figure 3:**
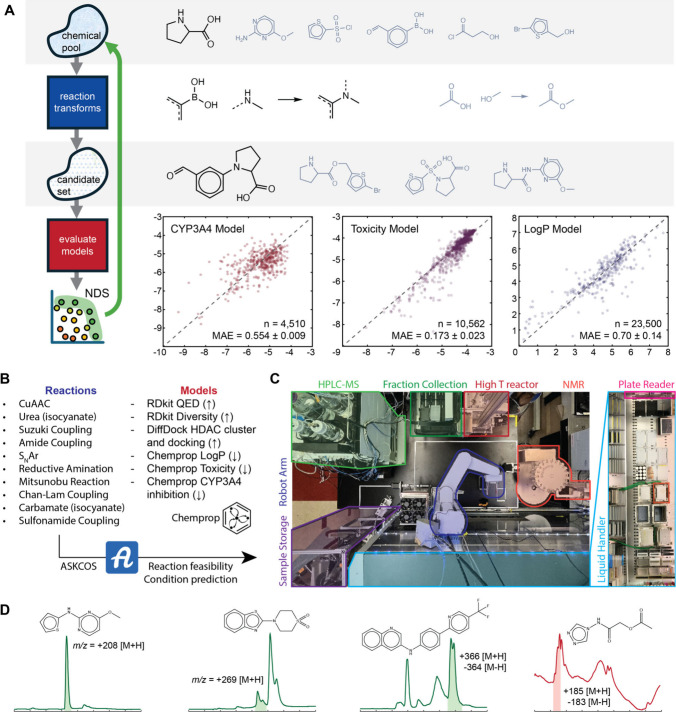
Schematic of molecular generation, reaction planning, reaction execution, and property testing in the MF-BO led discovery of new HDACIs. (A) The genetic algorithm for molecular generation selects building block molecules (top row) and applies reaction templates (middle row) to produce candidates (bottom row) iteratively. The candidates are ranked by several models, sorted by NDS, and top performers are recombined with building blocks to evolve more candidates. (B) Lists of the reactions and models used to generate candidates. ASKCOS is used to transform synthesis plans built during generation into executable experiments for the automated platform. (C) Top-down view of the automated platform. (D) Sample chromatograms of reactions yielding target products, as indicated by the expected mass-to-charge ratio, to demonstrate automated concentration analysis, with three passing reactions (green) and one failing (red) sample that could not be automatically deconvoluted.

In the second part of this study, we used MF-BO integrated into an autonomous molecular discovery platform to discover HDACIs with minimal experimental budget. The first step was generating a search space of molecules that could be synthesized automatically by the platform. In addition to being synthesizable, candidate molecules should be diverse and drug-like, with low logP for bioavailability, low toxicity, and low off-target activity. To achieve these characteristics simultaneously, a genetic algorithm, pictured in Figure 3A, was devised similar to Autogrow4.^31^ The key developments in the generative algorithm are the use of multiple machine learning models to score molecules,^32^ nondominated sorting (NDS) to select molecules that perform well across all of the design criteria,^33^ and a specific set of reaction templates that are compatible with automated liquid handling in air.^26^ The genetic operations of mutation, crossover, and selection are based on applying (or removing) reaction templates, mixing molecules that share a common template, and ranking molecules by NDS, respectively.

The algorithm is seeded with a random sampling of building block molecules available in the platform library (Figure 3A, top row). Compatible reaction templates are randomly applied to the seeds, with suitable reactants randomly selected from the library to build the first generation of molecules (Figure 3A, middle row). Each of the candidates is evaluated with a suite of models (bottom row): quantitative estimate of drug-likeness (QED) is calculated with RDkit,^34^ diversity is scored as one minus the average of the pairwise Tanimoto similarities (based on Morgan fingerprints) between the candidate and each of the other candidates, toxicity, water-octanol partitioning (logP), and off target activity toward cytochrome P450 3A4 (CYP3A4) are scored with Chemprop,^32^ and HDAC binding is assessed with DiffDock.^14^ Even with DiffDock^35^ docking limits algorithm throughput. To alleviate this, molecules are clustered,^36^ the medoid molecule is docked, and the medoid docking score is assigned to each of the molecules in the cluster. The molecules are ranked by NDS and each of the nondominated molecules becomes a seed for the next generation (elitism); the remainder of the next generation is filled by stochastic universal sampling^37^ with fitnesses equal to the inverse of NDS rank. In subsequent iterations of the genetic algorithm, in addition to applying reaction templates, templates can be undone or two molecules crossed-over a common template to create two new molecules. The choice to apply, remove, or crossover a template is proportional to the molecular weight of the molecule, so that generated molecules have molecular weights between 100–500 Da. Furthermore, molecules are filtered to remove those with undesirable substructures based on PAINS rules.^38^ Emphasis was placed on removing reactive substructures, which are difficult for the platform to test automatically. For HDACIs in particular, hydroxamic acids are removed to prevent the algorithm from converging on the well-known but troublesome hydroxamate local optimum. Multiple independent runs with a limited number of generations tend to uncover multiple local optima and perform better than single runs of many generations, which can accumulate undesirable functionalities. More details of molecular generation can be found in the SI.

The models for toxicity, logP, and CYP3A4 were developed using Chemprop, which uses a directed message passing neural network to learn a molecular representation for the prediction task at hand. Predictions are then made using the learned representation and a feed forward neural network. In each case, ensembles of five models were trained, and the variance between predicted values from each model in the ensemble is used as a proxy for the uncertainty of that prediction. The toxicity model was trained on more than 10,000 results from a cellular toxicity assay reported in the Tox21 dataset.^39,40^ The assay exposes HEK293 cells to serial dilutions of the toxicant for a set period, then measures residual ATP in the lysed cells by means of a luminescent luciferase assay as a gauge of viability, with nonviable cells having no ATP remaining. The log-10 toxicant concentration at which the luminescence intensity was halved in 8 h, interpolated from the serial dilution data, was used to train the model. The training data were weighted by the coefficient of determination (R^2^) from the logistic fit of serial dilution data, resulting in a model with a mean absolute error (MAE) of 0.173 ± 0.023. This measure of toxicity was chosen because the automated platform can easily execute the assay (RealTime-Glo MT Cell Viability Assay). The logP model is based on the work of Vermeire et al., which uses a predicted value of the free energy of solvation (Δ*G*_*solv*_) as an additional feature appended to the learned representation from the training molecules.^41,42^ The model is trained on more than 20,000 literature logP values^43^ and attained an MAE of 0.70 ± 0.14. LogP is automatically measured experimentally by calibrating the retention time in the HPLC column against compounds of known logP.^26^ The CYP3A4 model is based on approximately 5,000 pIC_50_ values (log-10 inhibitor concentration at which enzymatic activity is reduced by 50%) in the publicly available ChEMBL Database.^44^ Using default Chemprop settings a model with an MAE of 0.554 ± 0.009 was obtained. The platform can automatically measure CYP3A4 IC_50_ with commercially available fluorogenic assays (Abcam CYP3A4). The performance of each model on a held-out validation set is shown in Figure 3A.

The genetic algorithm produces multistep partial synthesis plans while generating candidates. To complete the synthesis plans, reaction conditions need to be predicted so that the platform can know how to execute the plans automatically. We use ASKCOS, a suite of CASP tools, to complete and evaluate synthesis plans. The condition recommender in ASKCOS is used to predict multiple sets of solvent, reagents, and catalysts for a reaction based on reactants and product from candidate generation.^45^ Each set of solvent, reagents, catalysts, reactants, and product are then screened using the ASKCOS forward prediction tool to eliminate conditions sets have low probability of succeeding.^46^ This prevents attempting synthesis plans with mismatched reactivity, for example trying a Mitsunobu esterification between the alcohol in 3-hydroxypropanoyl chloride and the acid in proline shown in Figure 3A; this reaction will fail because the acyl chloride and amine will react to form an amide instead. Lastly, conditions that cannot be safely executed on the platform, such as those using hydrogen as a reductant, are eliminated.

To execute synthesis plans, the autonomous platform used a Tecan Freedom Evo liquid handling system to prepare reactions in 96 well plates and work-up reactions by liquid–liquid extraction and filtration. Custom reactors enable access to high temperature and nitrogen purged environments. HPLC-MS is used to analyze reaction outcomes and isolate target products. Reaction success is determined by observation of the target product mass charge ratio (*m*/*z*) in the chromatogram. The concentration of the target is resolved by deconvolving photodiode array data coupled to a model for molar extinction coefficient.^47^ Isolated peaks are automatically collected in semiprepartive mode. A robot arm shuttles well plates between reactors and instruments.

HDAC inhibition is automatically measured in a plate reader with a fluorometric assay (Abcam ab283378). In the plate-based assay, the liquid handler first dispenses individual purified products in dimethyl sulfoxide into each well, then adds buffer containing HDAC and a peptide substrate containing an acetylated lysine residue. The solution is agitated and incubated for 30 min at 37 °C, then a developer, which reacts with primary amines to form a fluorescent product, is dispensed and incubated for another 30 min. After the second incubation, the plate is transferred to the plate reader to measure fluorescence corresponding to uninhibited HDAC activity. The platform handles all aspects of the assay; dispensing reagents, timing incubation, transferring plates, preparing positive and negative controls, and analyzing fluorescent readouts without human input.

**Figure 4:**
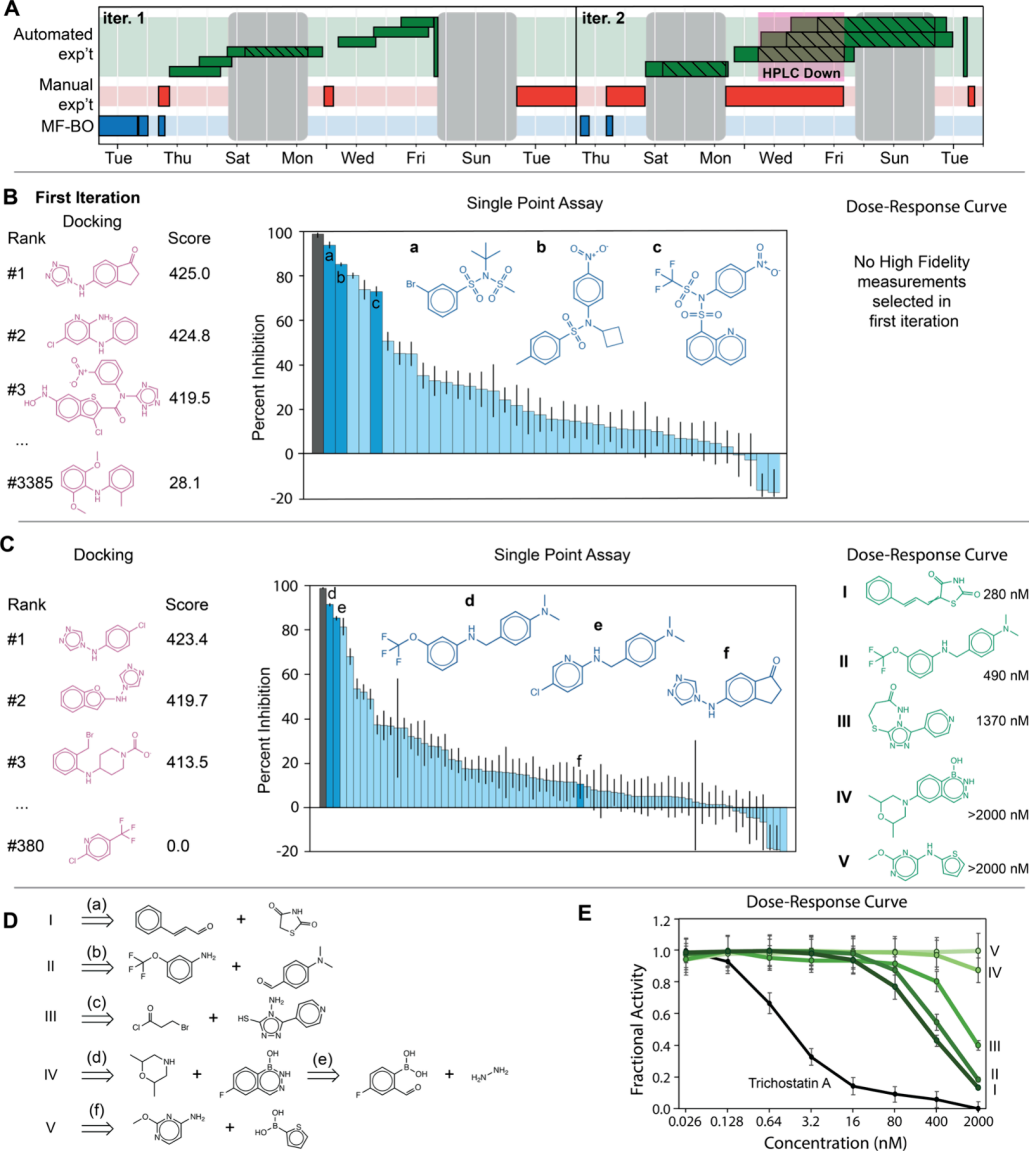
(A) Timeline of automated discovery of HDACIs. Blue represents computational tasks, red represents manual tasks, and green represents automated physical experiments. Each green bar groups the actions required to prepare, process, and analyze a single plate of reactions. Cross hatched bars indicate pauses during which new operations were not started but operations underway were allowed to complete. The magenta box indicates an isolated equipment failure. The gray boxes represent weekends during which starting new work was suspended. Results of the first (B) and second (C) iterations of the prospective application of MF-BO to discover new HDACIs. A sample of generated molecules selected for docking, with docking rank and scores, are shown in pink. Some of the molecules automatically synthesized and tested for single-point percent inhibition are shown in blue, along with the distribution of percent inhibitions for the successfully synthesized molecules: gray bar indicates trichostatin A, a potent natural product, dark blue bars correspond to highlighted molecules with inset structures. Structures of molecules tested at the highest fidelity along with IC_50_ values are shown in green (cf. trichostatin A IC_50_ of 1.8 nM); note that in the first iteration, no dose–response experiments were proposed. (D) The retrosyntheses of the high-fidelity molecules in (C) along with reaction conditions and yield as determined by HPLC. Conditions. (a) dioxane, 100 °C, 4 h, 75% yield, 5:4 cis:trans isomers. (b) DCM, 20 °C, 1 h then 3 eq. sodium triacetoxyborohydride, 20 °C, 3 h, 42% yield. (c) 2 eq. acid chloride, dioxane, 50 °C, 1 h, then reflux, 16 h, 32% yield. (d) 3 eq. DIPEA, dioxane, reflux, 16 h, 5% yield. (e) 1 eq. hydrazine hydrate, THF, 20 °C, 15 min, quantitative yield. (f) 1 eq. Cu(OAc)_2_·H_2_O, 3 eq. pyridine, DCM, 20 °C, 4 h, air, 96% yield. (E) Dose–response curves from which the IC_50_ values in (C) were derived.

The platform control architecture is modular, with modules organized around each of the main pieces of equipment outlined above and shown in Figure 3C. The modules are assigned high level goals by the master controller, and each module attempts to complete its assigned goal based on its capabilities, physical resources, and information available in the platform database (such as location of labware, contents of wells, *etc*.). If a module is unable to achieve its goal, it notifies the master controller that it has failed and, if possible, updates the controller with the tasks that must be accomplished to enable completion of the original goal. Modularity also ensures that when one instrument experiences an error, operation can continue in other modules (see Figure 4A, HPLC failure did not halt operation). In this way, orchestration is seamless, flexible, and robust, and operation can continue allowing the platform to handle up to 200 reactions per day. Human intervention is minimal, mainly limited to managing the reagent library and resolving fatal errors like collisions and clogs.

The platform is also adaptable to chemical outcomes in addition to automation outcomes. Figure 3D shows a sample of four HPLC-MS chromatograms. In all four cases, the desired *m*/*z* was detected, but the purity of the peak in the fourth sample resulted in the platform ignoring that candidate when it came time to run the HDAC inhibition assay.

The molecular generator was used to build a synthesizable search space of approximately 5,000 molecules. The generated molecules, combined with the reported HDAC8 inhibitors in ChEMBL, constituted a diverse search space, *D* = 0.87, with a weak correlation between fidelities, |ρ| = 0.38—ideal for MF-BO. While 5,000 molecules is not a large search space, the space was constructed with two aims, making synthetically realizable molecules given the platform’s capabilities, and finding the Pareto front defined by models of HDACI activity, logP, CYP3A4, and toxicity. The second aim pushes the search space toward “more active” molecules, but results in molecules with high variance on their predicted properties; they are outside the scope of the generative models. Measurements of the molecules’ properties, selected by MF-BO, are needed to efficiently find optima in the enriched search space.

The autonomous platform was initialized with docking scores, simulated single-point measurements, and IC_50_ values for the HDAC8 inhibitors in ChEMBL. Each iteration of MF-BO was organized into a campaign to be executed straight through. Between campaigns the results were manually checked, and the platform was restocked. Iterations could have been run without pause; however, the intermissions allowed for the manual fine-tuning of operations, which resulted in higher reaction success rates in the second iteration (see bar charts in Figures 4B and 4C).

Figure 4A shows the timeline for the complete MF-BO powered discovery of new HDACIs. Two iterations of the entire discovery cycle required one month, working Monday through Friday; weekend breaks are shown as gray boxes in Figure 4A. Molecular generation, MF-BO selection, and ASKCOS synthesis plan completion are shown as blue boxes in Figure 4A, representing the computational part of each cycle. Platform operation is shown as green boxes, with each box indicating the operations required to process a well plate of reactions, and hatched boxes indicating pauses during which new operations were not started (but operations already underway were allowed to automatically complete); this represents the automated experiment part of each cycle. Manual experiments are shown in red, and they include stocking the platform, analysis of results, synthesis of purification for dose–response experiments, and error handling. The magenta box labeled “HPLC down” resulted in an unplanned pause during which the HPLC was repaired while other platform modules continued their synthesis operations. A labeled version of Figure 4A is shown in Figure S5.

In the first iteration, the experimental design algorithm recommended that 3,385 candidates be docked (39% of budget), 261 candidates screened for single-point percent inhibition (61% of budget), and no candidates be manually synthesized for full dose–response characterization (0% of budget). The total budget was 100.0 with the same experiment cost structure of 0.01 for docking, 0.2 for single-point, and 1.0 for dose response curve. Docking was automatically performed with DiffDock, and the resulting conformers scored based on their binding to the catalytic zinc ion and overlap with bound known inhibitors from X-ray crystallography. For the candidates selected for automatic synthesis, the reactions were batched by temperature into well plates to be run in parallel on the automated platform. With a maximum allowance of 6 well plates, the platform attempted to synthesize 79 candidates, with an average of 2.4 reaction steps required for each candidate. Based on observation of the expected mass-to-charge ratio (*m*/*z*) during automatic HPLC-MS analysis, 40 of the syntheses were successful. The successful syntheses were automatically purified with semipreparative HPLC, the concentration of the inhibitor was automatically estimated,^47^ and the fluorometric inhibition assay was automatically run at 20 μM scale. The structures, synthesis plans, assay scores, and statistics for the attempted molecules are in the SI.

Figure 4B shows some of the selected candidates from the first iteration, their performance in docking and their performance in the single-point screen in comparison to Trichostatin A, a potent natural product inhibitor. In the initial round, while casting a wide net for docking, the algorithm mostly clustered the selection of single-point screens around tertiary sulfonamides. These structures are found in many inhibitors of zinc-dependent metalloproteases but are barely explored for HDAC inhibition (of the ∼700 examples of tertiary sulfonamides with HDACI activity in Reaxys, ∼600 contain a hydroxamate moiety, a moiety which was intentionally excluded from this study). Several of the molecules showed appreciable inhibitory activity, although they would likely need further optimization for high potency and other drug properties. The single point inhibitions for compounds **a**, **b**, and **c** correspond to IC_50_ values of 1140 nM, 3310 nM, and 7250 nM, respectively, assuming that binding of each ligand follows the Hill equation and is noncooperative. These are weak assumptions, and in a classical experimental funnel these top performers would be checked with a dose–response experiment to measure the actual IC_50_ value. The data from the first iteration (docking and single-point percent inhibition scores) were added to the training data and the MF-BO algorithm was used to select a second iteration of experiments.

In the second round the budget allocation shifted away from docking experiments toward more dose–response assays. 380 candidates were automatically docked and scored (4% of budget), 336 candidates were chosen for single-point screening (67% of budget), and 8 candidates were manually synthesized and purified for full dose–response characterization (29% of budget). The platform planned and attempted to execute 106 single-point syntheses over four well plates (again, roughly half the molecules did not pass the combined reaction + condition evaluation), with 70 successful syntheses as indicated by HPLC-MS, shown in Figure 4C. The higher execution success rate may be attributed to the shorter average synthesis plan, with the average product requiring only 1.4 steps compared to 2.4 steps in the first iteration. In the second round, the algorithm recommended running the single-point assay on the top docked candidate from the first round, 5-((1,2,4-triazol-4-yl)amino)-indan-1-one, showing how elements of the experimental funnel are captured by the algorithm. However, this molecule shows only 11% inhibition in the single-point assay, demonstrating the weak correlation between docking and single-point screening. Several other 4-amino-1,2,4-triazoles were selected as well; this substructure performed well in docking but less so as an actual inhibitor. Instead, derivatives of 4-(*N*,*N*-dimethylamino)-benzaldehyde were the top performing molecules in the single-point assay.

At the highest fidelity, the model chose to measure a top performing 4-(*N*,*N*-dimethylamino)-benzaldehyde derivative to learn the covariance with the middle fidelity. Other molecules selected for dose response were a biheterocyclic amine, a thiazolidinedione derivative, a 4-amino-1,2,4-triazole derivative, and a benzodiazoborinol (the last of which has been discussed as an underexplored zinc binding warhead).^48^ The selected molecules were synthesized manually while the platform synthesized the single-point candidates automatically, as shown by the concurrent green and red bars in the second iteration in Figure 4A. The syntheses of these molecules (I–V) are shown in Figure 4D, and the dose–response curves from which the IC_50_ values were derived are shown in Figure 4E with trichostatin A for reference. Unsuccessful molecules selected for dose–response measurement included a phenylalanine derivative, an imidazolidine derivative, and a 2-aminobenzothiazole derivative. While none of the successfully synthesized molecules match Trichostatin A in terms of potency, other inhibitors have been approved by the FDA with IC_50_ values on par with the best molecules discovered in this campaign, such as valproate, a nonhydroxamate inhibitor with an IC_50_ of 180000 nM,^49^ and givinostat, a hydroxamate with an IC_50_ of 837 nM.^50^ The therapeutic utility of a drug depends not only on potency, but also on properties like bioavailability and off-target activity, which were considered during molecular generation, and in just two rounds of optimization, several diverse lead compounds were uncovered with appreciable potency and pharmacological properties. Moreover, a better understanding of the HDACI chemical landscape at multiple fidelities was developed.

## Conclusions

We have developed a method to make use of multiple fidelities of experimental data to accelerate the discovery of new drug molecules. Compared to previous methods using multiple experimental fidelities, the algorithm efficiently explores diverse chemical space by learning how correlation between different types of experiments vary across the chemical search space. This approach is especially useful for large/diverse search spaces where different fidelities are correlated in a nonobvious way. In cases of narrow search spaces or strong fidelity correlation, Bayesian optimization or an experimental funnel approach outperforms our method. We first observed these utility trends across five data sets for different druggable protein targets, then confirmed the trends using structured data sets sampled from measured acetylcholinesterase inhibitors. Intuitively, the model started by allocating the experimental budget to low-cost experiments to explore the entire space, then all fidelities of experimentation to learn the correlation mapping, and finally to high-fidelity experiments to discover top performers.

We then integrated this optimization approach, along with a tailored genetic algorithm for molecular generation, into an autonomous molecular discovery platform, which was used to search for new HDACIs. In the first iteration, the platform automatically selected and ran >3,000 docking experiments and >100 single-point percent inhibition experiments; this required automatically planning, executing, and working up >300 reactions and quantifying 79 concentrations and fluorescence-based assay outcomes. In the second iteration, more docking and single-point experiments were selected, as well as several manual syntheses and dose–response curve measurements. After two iterations of the optimization algorithm, the method uncovered diverse structures with HDAC inhibitory activity, which could act as a starting point for lead optimization. Of note, other structures that docking suggests have tight binding proved to be poor performers.

## Data Availability

All code, data, synthesis plans, and models are available in the Zenodo repository at 10.5281/zenodo.13983042.

## Supporting Information Available

Details of the genetic algorithm for molecule generation, MF-BO algorithm, implementations of other search algorithms, evaluation of other surrogate models and representations, overviews of the test data sets from part 1, details of DiffDock use and comparison to Autodock Vina, details of ASKCOS use, synthesis details and ^1^H-NMR spectra for compounds I–V, and details of the automated platform (PDF)
All synthesis pathways from iteration 1 (PDF)
All synthesis pathways from iteration 2 (PDF)

## Abbreviations

BO: Bayesian optimization
MF-BO: multifidelity Bayesian optimization
GP: Gaussian process
TVR: targeted variance reduction
HDACI: histone deacetylase inhibitor
PARP1: poly[ADP-ribose] polymerase 1
HIF-PH: hypoxia-inducible factor prolyl hydroxylase
CXCR4: chemokine receptor 4
NR1A2: thyroid hormone receptor beta
CYP3A4: cytochrome P450 3A4

## References

[ref1] Yan X. C.; Sanders J. M.; Gao Y.-D.; Tudor M.; Haidle A. M.; Klein D. J.; Converso A.; Lesburg C. A.; Zang Y.; Wood H. B. Augmenting Hit Identification by Virtual Screening Techniques in Small Molecule Drug Discovery. J. Chem. Inf. Model. 2020, 60 (9), 4144–4152. 10.1021/acs.jcim.0c00113. 32309939

[ref2] Buterez D.; Janet J. P.; Kiddle S. J.; Oglic D.; Lió P. Transfer learning with graph neural networks for improved molecular property prediction in the multi-fidelity setting. Nat. Commun. 2024, 15 (1), 1517 10.1038/s41467-024-45566-8. 38409255 PMC11258334

[ref3] Ghiandoni G. M.; Evertsson E.; Riley D. J.; Tyrchan C.; Rathi P. C. Augmenting DMTA using predictive AI modelling at AstraZeneca. Drug Discovery Today 2024, 29 (4), 103945 10.1016/j.drudis.2024.103945. 38460568

[ref4] Wesolowski S. S.; Brown D. G. The Strategies and Politics of Successful Design, Make, Test, and Analyze (DMTA) Cycles in Lead Generation. In Lead Generation 2016, 487–512. 10.1002/9783527677047.ch17.

[ref5] Pyzer-Knapp E. O. Bayesian optimization for accelerated drug discovery. IBM J. Res. Dev. 2018, 62 (6), 2:1–2:7. 10.1147/JRD.2018.2881731.

[ref6] Bellamy H.; Rehim A. A.; Orhobor O. I.; King R. Batched Bayesian Optimization for Drug Design in Noisy Environments. J. Chem. Inf. Model. 2022, 62 (17), 3970–3981. 10.1021/acs.jcim.2c00602. 36044048 PMC9472273

[ref7] Colliandre L.; Muller C. Bayesian Optimization in Drug Discovery. In High Performance Computing for Drug Discovery and Biomedicine; Heifetz A., Ed.; Springer: 2024; pp 101–136. 10.1007/978-1-0716-3449-3_5 37702937

[ref8] Song J.; Chen Y.; Yue Y. A General Framework for Multi-fidelity Bayesian Optimization with Gaussian Processes. In Proceedings of the Twenty-Second International Conference on Artificial Intelligence and Statistics, Proceedings of Machine Learning Research; 2019.

[ref9] Le Gratiet L.; Cannamela C. Cokriging-Based Sequential Design Strategies Using Fast Cross-Validation Techniques for Multi-Fidelity Computer Codes. Technometrics 2015, 57 (3), 418–427. 10.1080/00401706.2014.928233.

[ref10] Ehara A.; Guillas S. AN ADAPTIVE STRATEGY FOR SEQUENTIAL DESIGNS OF MULTILEVEL COMPUTER EXPERIMENTS. International Journal for Uncertainty Quantification 2023, 13 (4), 61–98. 10.1615/Int.J.UncertaintyQuantification.2023038376.

[ref11] Fare C.; Fenner P.; Benatan M.; Varsi A.; Pyzer-Knapp E. O. A multi-fidelity machine learning approach to high throughput materials screening. npj Computational Materials 2022, 8 (1), 257 10.1038/s41524-022-00947-9.

[ref12] Gantzler N.; Deshwal A.; Doppa J. R.; Simon C. M. Multi-fidelity Bayesian optimization of covalent organic frameworks for xenon/krypton separations. Digital Discovery 2023, 2 (6), 1937–1956. 10.1039/D3DD00117B.

[ref13] Kalinin S. V.; Slautin B. N.; Pratiush U.; Ivanov I. N.; Liu Y.; Pant R.; Zhang X.; Takeuchi I.; Ziatdinov M. A. Multimodal Co-orchestration for Exploring Structure-Property Relationships in Combinatorial Libraries via Multi-Task Bayesian Optimization. Microscopy and Microanalysis 2024, 30 (Supplement_1), ozae044.157 10.1093/mam/ozae044.157.

[ref14] Corso G.; Stärk H.; Jing B.; Barzilay R.; Jaakkola T. Diffdock: Diffusion steps, twists, and turns for molecular docking. arXiv preprint arXiv:2210.01776, 2022.

[ref15] Miller T. A.; Witter D. J.; Belvedere S. Histone Deacetylase Inhibitors. J. Med. Chem. 2003, 46 (24), 5097–5116. 10.1021/jm0303094. 14613312

[ref16] Zhou M.; Yuan M.; Zhang M.; Lei C.; Aras O.; Zhang X.; An F. Combining histone deacetylase inhibitors (HDACis) with other therapies for cancer therapy. Eur. J. Med. Chem. 2021, 226, 113825 10.1016/j.ejmech.2021.113825. 34562854 PMC9363153

[ref17] Shukla S.; Tekwani B. L. Histone Deacetylases Inhibitors in Neurodegenerative Diseases, Neuroprotection and Neuronal Differentiation. Frontiers in Pharmacology 2020, 11, 537 10.3389/fphar.2020.00537. 32390854 PMC7194116

[ref18] Frühauf A.; Meyer-Almes F.-J. Non-Hydroxamate Zinc-Binding Groups as Warheads for Histone Deacetylases. Molecules 2021, 26, 5151 10.3390/molecules26175151. 34500583 PMC8434074

[ref19] Muri E. M.; Nieto M. J.; Sindelar R. D.; Williamson J. S. Hydroxamic acids as pharmacological agents. Curr. Med. Chem. 2002, 9 (17), 1631–1653. 10.2174/0929867023369402. 12171558

[ref20] Jaeger S.; Fulle S.; Turk S. Mol2vec: Unsupervised Machine Learning Approach with Chemical Intuition. J. Chem. Inf. Model. 2018, 58 (1), 27–35. 10.1021/acs.jcim.7b00616. 29268609

[ref21] Moriwaki H.; Tian Y.-S.; Kawashita N.; Takagi T. Mordred: a molecular descriptor calculator. Journal of Cheminformatics 2018, 10 (1), 4 10.1186/s13321-018-0258-y. 29411163 PMC5801138

[ref22] Rogers D.; Hahn M. Extended-Connectivity Fingerprints. J. Chem. Inf. Model. 2010, 50 (5), 742–754. 10.1021/ci100050t. 20426451

[ref23] Griffiths R.-R.; Klarner L.; Moss H.; Ravuri A.; Truong S.; Du Y.; Stanton S.; Tom G.; Rankovic B.; Jamasb A. Gauche: A library for Gaussian processes in chemistry. Advances in Neural Information Processing Systems 2023, 36.

[ref24] Liu H.; Ong Y. S.; Shen X.; Cai J. When Gaussian Process Meets Big Data: A Review of Scalable GPs. IEEE Transactions on Neural Networks and Learning Systems 2020, 31 (11), 4405–4423. 10.1109/TNNLS.2019.2957109. 31944966

[ref25] Eriksson D.; Jankowiak M. High-dimensional Bayesian optimization with sparse axis-aligned subspaces. In Proceedings of the Thirty-Seventh Conference on Uncertainty in Artificial Intelligence, Proceedings of Machine Learning Research; 2021.

[ref26] Koscher B. A.; Canty R. B.; McDonald M. A.; Greenman K. P.; McGill C. J.; Bilodeau C. L.; Jin W.; Wu H.; Vermeire F. H.; Jin B.; et al. Autonomous, multiproperty-driven molecular discovery: From predictions to measurements and back. Science 2023, 382 (6677), eadi1407 10.1126/science.adi1407. 38127734

[ref27] Fromer J. C.; Coley C. W. An algorithmic framework for synthetic cost-aware decision making in molecular design. Nature Computational Science 2024, 4 (6), 440–450. 10.1038/s43588-024-00639-y. 38886590

[ref28] Gaulton A.; Bellis L. J.; Bento A. P.; Chambers J.; Davies M.; Hersey A.; Light Y.; McGlinchey S.; Michalovich D.; Al-Lazikani B.; et al. ChEMBL: a large-scale bioactivity database for drug discovery. Nucleic Acids Res. 2012, 40 (D1), D1100–D1107. 10.1093/nar/gkr777. 21948594 PMC3245175

[ref29] Goutelle S.; Maurin M.; Rougier F.; Barbaut X.; Bourguignon L.; Ducher M.; Maire P. The Hill equation: a review of its capabilities in pharmacological modelling. Fundamental & Clinical Pharmacology 2008, 22 (6), 633–648. 10.1111/j.1472-8206.2008.00633.x. 19049668

[ref30] Stein R. M.; Yang Y.; Balius T. E.; O’Meara M. J.; Lyu J.; Young J.; Tang K.; Shoichet B. K.; Irwin J. J. Property-Unmatched Decoys in Docking Benchmarks. J. Chem. Inf. Model. 2021, 61 (2), 699–714. 10.1021/acs.jcim.0c00598. 33494610 PMC7913603

[ref31] Spiegel J. O.; Durrant J. D. AutoGrow4: an open-source genetic algorithm for de novo drug design and lead optimization. Journal of cheminformatics 2020, 12 (1), 1–16. 10.1186/s13321-020-00429-4. 33431021 PMC7165399

[ref32] Heid E.; Greenman K. P.; Chung Y.; Li S.-C.; Graff D. E.; Vermeire F. H.; Wu H.; Green W. H.; McGill C. J. Chemprop: A Machine Learning Package for Chemical Property Prediction. J. Chem. Inf. Model. 2024, 64 (1), 9–17. 10.1021/acs.jcim.3c01250. 38147829 PMC10777403

[ref33] Fromer J. C.; Graff D. E.; Coley C. W. Pareto optimization to accelerate multi-objective virtual screening. Digital Discovery 2024, 3 (3), 467–481. 10.1039/D3DD00227F.

[ref34] RDKit: Open-source cheminformatics; 2006. http://www.rdkit.org.

[ref35] Eberhardt J.; Santos-Martins D.; Tillack A. F.; Forli S. AutoDock Vina 1.2.0: New Docking Methods, Expanded Force Field, and Python Bindings. J. Chem. Inf. Model. 2021, 61 (8), 3891–3898. 10.1021/acs.jcim.1c00203. 34278794 PMC10683950

[ref36] Schubert E.; Rousseeuw P. J. Fast and eager k-medoids clustering: O(k) runtime improvement of the PAM, CLARA, and CLARANS algorithms. Information Systems 2021, 101, 101804 10.1016/j.is.2021.101804.

[ref37] Baker J. E. Reducing bias and inefficiency in the selection algorithm. Proceedings of the second international conference on genetic algorithms 1987, 206, 14–21.

[ref38] Baell J. B.; Holloway G. A. New Substructure Filters for Removal of Pan Assay Interference Compounds (PAINS) from Screening Libraries and for Their Exclusion in Bioassays. J. Med. Chem. 2010, 53 (7), 2719–2740. 10.1021/jm901137j. 20131845

[ref39] Huang R.; Xia M.; Sakamuru S.; Zhao J.; Lynch C.; Zhao T.; Zhu H.; Austin C. P.; Simeonov A. Expanding biological space coverage enhances the prediction of drug adverse effects in human using in vitro activity profiles. Sci. Rep 2018, 8 (1), 3783 10.1038/s41598-018-22046-w. 29491351 PMC5830476

[ref40] Huang R.; Xia M.; Sakamuru S.; Zhao J.; Shahane S. A.; Attene-Ramos M.; Zhao T.; Austin C. P.; Simeonov A. Modelling the Tox21 10 K chemical profiles for in vivo toxicity prediction and mechanism characterization. Nat. Commun. 2016, 7 (1), 10425 10.1038/ncomms10425. 26811972 PMC4777217

[ref41] Vermeire F. H.; Chung Y.; Green W. H. Predicting Solubility Limits of Organic Solutes for a Wide Range of Solvents and Temperatures. J. Am. Chem. Soc. 2022, 144 (24), 10785–10797. 10.1021/jacs.2c01768. 35687887

[ref42] Vermeire F. H.; Green W. H. Transfer learning for solvation free energies: From quantum chemistry to experiments. Chemical Engineering Journal 2021, 418, 129307 10.1016/j.cej.2021.129307.

[ref43] Chung Y.; Vermeire F. H.; Wu H.; Walker P. J.; Abraham M. H.; Green W. H. Group Contribution and Machine Learning Approaches to Predict Abraham Solute Parameters, Solvation Free Energy, and Solvation Enthalpy. J. Chem. Inf. Model. 2022, 62 (3), 433–446. 10.1021/acs.jcim.1c01103. 35044781

[ref44] Zdrazil B.; Felix E.; Hunter F.; Manners E. J.; Blackshaw J.; Corbett S.; de Veij M.; Ioannidis H.; Lopez D. M.; Mosquera Juan F.; et al. The ChEMBL Database in 2023: a drug discovery platform spanning multiple bioactivity data types and time periods. Nucleic Acids Res. 2024, 52 (D1), D1180–D1192. 10.1093/nar/gkad1004. 37933841 PMC10767899

[ref45] Gao H.; Struble T. J.; Coley C. W.; Wang Y.; Green W. H.; Jensen K. F. Using Machine Learning To Predict Suitable Conditions for Organic Reactions. ACS Central Science 2018, 4 (11), 1465–1476. 10.1021/acscentsci.8b00357. 30555898 PMC6276053

[ref46] Jin W.; Coley C.; Barzilay R.; Jaakkola T. Predicting organic reaction outcomes with weisfeiler-lehman network. Advances in neural information processing systems 2017, 30.

[ref47] McDonald M. A.; Koscher B. A.; Canty R. B.; Jensen K. F. Calibration-free reaction yield quantification by HPLC with a machine-learning model of extinction coefficients. Chemical Science 2024, 15 (26), 10092–10100. 10.1039/D4SC01881H. 38966367 PMC11220585

[ref48] Kazmi M. Z. H.; Schneider O. M.; Hall D. G. Expanding the Role of Boron in New Drug Chemotypes: Properties, Chemistry, Pharmaceutical Potential of Hemiboronic Naphthoids. J. Med. Chem. 2023, 66 (19), 13768–13787. 10.1021/acs.jmedchem.3c01194. 37752013

[ref49] Mustafa M.; Abd El-Hafeez A. A.; Abdelhamid D.; Katkar G. D.; Mostafa Y. A.; Ghosh P.; Hayallah A. M.; Abuo-Rahma G. E.-D. A. A first-in-class anticancer dual HDAC2/FAK inhibitors bearing hydroxamates/benzamides capped by pyridinyl-1,2,4-triazoles. Eur. J. Med. Chem. 2021, 222, 113569 10.1016/j.ejmech.2021.113569. 34111829 PMC8818328

[ref50] Ho T. C. S.; Chan A. H. Y.; Ganesan A. Thirty Years of HDAC Inhibitors: 2020 Insight and Hindsight. J. Med. Chem. 2020, 63 (21), 12460–12484. 10.1021/acs.jmedchem.0c00830. 32608981

