# Supplementary material for: Bayesian Optimization over Multiple Experimental Fidelities Accelerates Automated Discovery of Drug Molecules
Source: ACS Cent Sci. 2025 Feb 5;11(2):346–56. doi: 10.1021/acscentsci.4c01991 (PMC11869128; doi:10.1021/acscentsci.4c01991)
Supplement: Supplementary file 1 — oc4c01991_si_001.pdf [file oc4c01991_si_001.pdf]

Supplemental Information for Bayesian Optimization over multiple experimental fidelities accelerates automated discovery of drug molecules

Matthew A. McDonald[1,2], Brent A. Koscher[1], Richard B. Canty[1], Jason Zhang[1], Angelina Ning[1], Klavs F. Jensen[1]*

[1] Massachusetts Institute of Technology, Department of Chemical Engineering, 77 Massachusetts Avenue, Cambridge, Massachusetts 02139, USA

[2] Drexel University, Department of Chemical and Biological Engineering, 3101 Ludlow St, Philadelphia, Pennsylvania 19104, USA

* email: kfjensen@mit.edu

Contents

List of Figures in Supplemental Information S2

Details of Molecular Generation S3

Table S1. Summary of all reaction templates used by the platform, including smarts strings used with RDKit to construct products S6

ASKCOS[1] use in generative model and platform operations S8

Implementation of MF-BO S8

Implementation of other search algorithms S10

Details MF-BO performance analysis S10

Details MF-BO performance analysis S14

Details of Docking, DiffDock implementation, and DiffDock Validation S16

Comparison to other surrogate model architectures and molecule representations S18

Characterization of literature datasets S20

Details of the autonomous platform S24

Details of automated operations (Figure 4A labels) S26

Synthesis and 1H NMR Spectra from select scaled-up molecules S27

Summary of automated reaction outcomes S32

Summary of assay results from HDACI discovery campaign S37

REFERENCES S42

List of Figures in Supplemental Information

Figure S1. TSNE plot of generated HDACIs colored based on the run from which they originated, as well as literature inhibitors with a hydroxamate group (black) and without (gray). S5

Figure S2. Simulated single point data based on log10 IC50 values from ChEMBL, the Hill equation, and the assumptions discussed in the paragraph above. Without any of the simulated uncertainty, noise, and limits of detection, the points would fall on a straight line. S9

Figure S3. The fraction of top-N% performers found for different values of N, for MFBO, BO, and random sampling against CXCR4 datasets containing decoy molecules from the DUDE-Z set. S12

Figure S4. The tSNE visualization of the datasets with decoys. Decoy molecules are represented by dark blue points, all other molecules are colored by their potency, with the most potent molecules colored yellow. S13

Figure S5. Performance of MF-BO on structured subsets of the Factor-D (top) and PARP1 (bottom) datasets. The figures were generated following the same procedure as outlined for Figures 2B and C in the main text. S15

Figure S6. Comparison of DiffDock pose (orange), Vina pose (lavender), and crystallized ligand (green) for vorinostat in HDAC8. Both docked poses closely coordinate the catalytic zinc (pink) and overlap considerably with the crystallized ligand. S16

Figure S7. Scatter plot of DiffDock and Vina scores for HDACIs reported in the literature. S17

Figure S8. Neither DiffDock nor Vina provide an obvious indication of which molecules are top performers in terms of pIC_{50}. Left, scatter plot of DiffDock scores for HDACIs with published IC_{50} values. Right, scatter plot of Vina binding energies for the same HDACIs. Arrows indicate the direction of the expected trend; higher DiffDock scores and larger magnitude (but negative sign) binding energies should correlate with higher potency (smaller pIC_{50}) ..S17

Figure S9. comparison of different molecular representations used with a GP. S18

Figure 10. Comparison of different model architectures using Morgan fingerprints. S19

Figure S11. Summary statistics for each of the datasets collected from CHEMBL. S22

Figure S12. tSNE visualizations demonstrating the degree of clustering in each dataset. The colorbars show pIC_{50} values scaled from 0 to 1. Those datasets that are more tightly clustered, with top performing molecules contained within a single cluster, tend not to be suitable for MF-BO. S24

Figure S13. The timeline of automated operation during the MF-BO campaign to discover new HDAC inhibitors. .S26

Figure S14. 1H-NMR spectrum of N,N-dimethyl-4-(((3-(trifluoromethoxy)phenyl)amino)methyl)aniline S27

Figure S15. 1H-NMR spectrum of 5-((E)-3-phenylallylidene)thiazolidine-2,4-dione. S28

Figure S16. 1H-NMR spectrum of 2-methoxy-N-(thiophen-3-yl)pyrimidin-4-amine. S29

Figure S17. 1H-NMR of intermediate benzodiazaborininol in synthesis of 6-((2S,6R)-2,6-dimethylmorpholino)benzo[d][1,2,3]diazaborinin-1(2H)-ol. S30

Figure S18. 1H-NMR spectrum of 6-((2S,6R)-2,6-dimethylmorpholino)benzo[d][1,2,3]diazaborinin-1(2H)-ol S31

Details of Molecular Generation

Molecules were generated by a genetic algorithm that uses reaction templates to perform mutation and crossover operations and non-dominated sorting (NDS) in the genetic selection operation. Molecules generated by adding and removing reaction templates are inherently synthesizable, as the method of generation simultaneously builds a synthesis plan. The reactions were defined with the SMARTS strings shown in Table S1, on the next page. The algorithm was implemented in Python using RDkit (and additional packages for specific evaluation models) and is available in the project repository.

Mutation is performed by applying compatible reaction templates to candidate molecules. In each iteration of the algorithm, the selected population (survivors) is randomly sampled with replacement and reaction templates are applied to generate new candidates (children). A template may be applied in one of three ways: (1) in an additive manner to build up the molecule with a new compatible template, (2) in a subtractive manner to remove a template that has already been applied to the candidate, or (3) in crossover (detailed below). At each mutation, there is a twenty percent chance of attempting crossover while the probability of adding or removing a template is dependent on the molecular weight (MW) of the candidate. If MW < 100 Da a template is added, if MW > 500 Da a template is removed, and if 100 Da < MW < 500 Da the probability varies linearly from only addition to only removal of templates. This probability ensures that the generated molecules have molecular weights appropriate for small molecule drugs. Candidates with more than four templates can only have templates removed, to prevent generating molecules with unfeasibly long synthesis plans. The decision to limit molecular weights to between 100 and 500 Da may cause the algorithm to miss some novel potentially therapeutic molecules. However, we found that when molecules start to larger than 500 Da, the platform cannot isolate sufficient quantities for the necessary assays.

When a template is added to a candidate, templates from the curated list (see table S1) are randomly sampled until a compatible template is selected; a template is considered compatible if the candidate contains exactly one instance of the reactive substructure of one of the reactant(s). Once the template is selected, if the template requires multiple reactants, a reactant(s) with matching substructure to the other part(s) of the reaction is(are) randomly sampled from the library of available chemicals. The template is then applied using RDkit to generate the child molecule. If no templates match the candidate, or no reactants in the library match the selected template, the candidate becomes a child with no modification.

When a template is removed from a candidate, one of the templates associated with that candidate is randomly selected, and the reaction is reversed to give the reactants for that reaction. The largest of the reactants (based on MW) then becomes the child molecule. If the selected template cannot be undone (it was modified by the addition or removal of other templates), then the candidate becomes a child with no modification. Addition and removal combine to account for 80% of mutations, crossover accounts for the remaining 20%.

Crossover occurs when two candidates share a common reaction template that has two reactants. Both candidates are broken down into their respective reactants, and the reactants are recombined to produce two new molecules. For example, assume candidates AB and CD are produced by the reactions A + B → AB and C + D → CD using the same template. The crossover children become A + D → AD and C + B → CB. If the candidates with common templates also share a common reactant, e.g. AB and CB, then crossover fails and no children are generated. If

either of the candidates cannot be broken down along the selected template (substructure modified by other operation) then no children are generated.

Selection occurs in between generations, where a predetermined number of children are selected to become the candidates in the next iteration. Selection required tuning of many hyperparameters based on molecule performance and generation speed. Those hyperparameters are explained below.

For this work, each generation consisted of 150 candidates, of which 50 were selected for the next generation. Selection occurs by two mechanisms, elitism, where all of the molecules tied for top rank are selected, and stochastic universal sampling (SUS), where the remainder of the survivors are selected with an unbiased adaptation of fitness proportional sampling. Elitism ensures that the top molecules make it to the next generation while SUS samples molecules with poor rank to provide diversity. Molecules are ranked using non-dominated sorting (NDS) on a subset of 6 properties (minimizing logP, toxicity, and off target activity [CYP3A4 inhibition], and maximizing quantitative estimate of drug-likeness [QED], Diversity, and clustered docking). Which properties were considered in the NDS depended on the generation number. The first 6 generations are warmup generations, where only QED and Diversity are used in selection to build a diverse drug-like starting set for application of drug-specific models. The next 15-30 generations are explore generations, where docking, logP, toxicity, and diversity are considered during NDS. Exploration occurs for at least 15 generations then halts if the center-of-mass of the candidates in TSNE-space stops moving substantially or the maximum 30 generations is reached. Lastly, 2-5 refinement generations that add CYP3A4 inhibition to the sorting criteria are used to remove candidates with suspected off target activity. The refinement generations also limit the available chemicals to those present in the final exploration generation to encourage generation of candidates with synergistic synthesis pathways.

The generative algorithm wsa written to use arbitrary models. The toxicity, logP, and CYP3A4 models were constructed with Chemprop v1. The training data for the models are provided in the data repository so that models can be retrained from the command line using Chemprop v1 with the command:

```
chemprop_train --save_dir ‘path_to_save’ --data_path ‘path_to_data.csv’ --dataset_type regression
--num_epochs 150 --ensemble_size 5
```

However, since the models were built Chemprop v1 has been replaced with v2 without backwards compatibility. A conversion script can be found in the Chemprop GitHub repository to upgrade models to work with Chemprop v2. QED was calculated with the RDkit QED function, and the diversity (minimized similarity) was calculated using RDkit BulkTanimotoSimilarity to get all pairwise scores, then averaging the score between each molecule and all the others in the generation one at a time to get a final similarity for each molecule. For docking, the candidates were first clustered into between 5 and 20 clusters (as determined by the FasterPAM k-medoid algorithm) and the medoid candidate was docked to human HDAC8 (PDB entry 1w22) using DiffDock with default parameters.

In addition to SUS, other forms of fitness proportional sampling were used, including roulette wheel sampling, tournament sampling, and linear ranking, however SUS paired with

elitism offered a simple and unbiased way to select both top performers and candidates that may be building towards higher activity.

The algorithm was run independently 10 times, as it tended to find local minima without thoroughly exploring the entire chemical space. All the candidates from the refine generations across all 10 runs formed the search space (roughly 8000 molecules) for prospective application of the MF-BO algorithm to discovery of new HDACIs. The candidates, as well as the inhibitors with IC_{50} values in ChEMBL, are plotted in TSNE-space in Figure S1, with different colors representing different initializations of the genetic algorithm. The figure shows that the generated molecules span a large and distinct chemical space compared to known inhibitors, especially hydroxamates in black. The grouping of points with the same color demonstrates how to the algorithm tends to find local minima quickly, but the roughness of the chemical space, and uncertainty of predictions, prevents a single generation from sampling the entire chemical space.

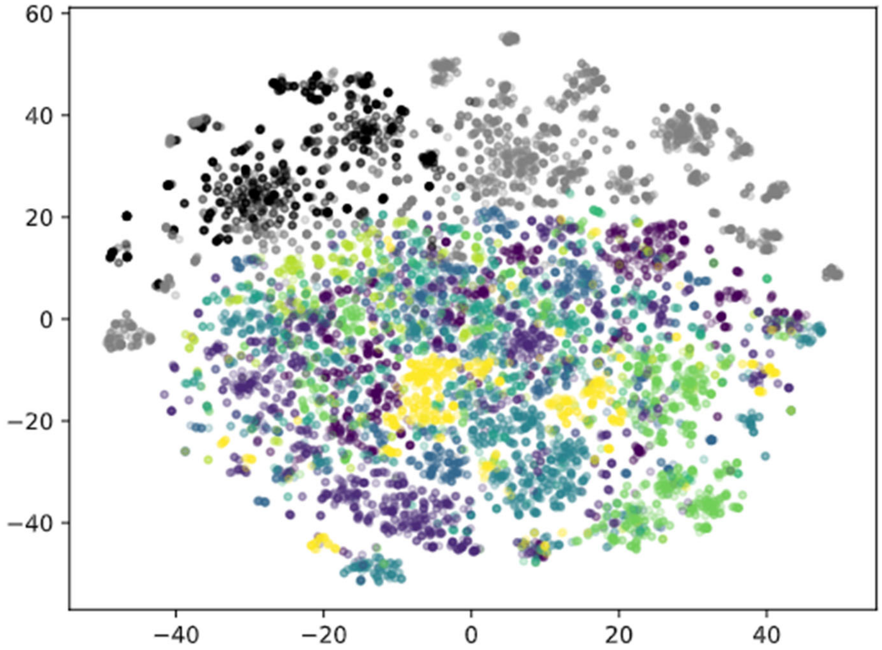

Figure S1. TSNE plot of generated HDACIs colored based on the run from which they originated, as well as literature inhibitors with a hydroxamate group (black) and without (gray).

Table S1. Summary of all reaction templates used by the platform, including smarts strings used with RDKit to construct products

Reaction	reactant1	reactant2	smarts reactant 1	smarts reactant 2	product smarts
Amide_amine_acid	amine	acid	[NX3;H2,H1;!$(NC=O):1]	[CX3:2](=[O:3])[OX1H0-,OX2H1]	[N:1]-[C:2](=[O:3])
Amide_amine_acidChloride	amine	acid_chloride	[NX3;H2,H1;!$(NC=O):1]	[CX3:2](=[OX1:3])[F,Cl,Br,I]	[N:1]-[C:2](=[O:3])
Carbamate_isocyanate	alcohol	isocyanate	[#6:1][OX2H:2]	[#6:3]-[N:4]=[C:5]=[O:6]	[#6:1]-[O:2]-[C:5](=[O:6])[NX3;H:4]-[#6:3]
Carbamate_chloroformate	amine	chloroformate	[#6:1][NX3;H2,H1;!$(NC=O):2]	[Cl]-[CX3:6](=[O:5])[OX2:4]-[#6:3]	[#6:3]-[O:4]-[C:6](=[O:5])[NX3;H:2]-[#6:1]
Carbamate_alcohol_acid	alcohol	acid	[#6:1][OX2H:2]	[#6:5][CX3:3](=[O:4])[OX1H0-,OX2H1]	[#6:1]-[O:2]-[C:3](=[O:4])[NX3;H][#6:5]
Carbamate_alcohol_acid_cl	alcohol	acid_chloride	[#6:1][OX2H:2]	[#6:5][CX3:3](=[OX1:4])[F,Cl,Br,I]	[#6:1]-[O:2]-[C:3](=[O:4])[NX3;H][#6:5]
Urea_isocyanate	amine	isocyanate	[#6:1][NX3;H2,H1;!$(NC=O):2]	[#6:3]-[N:4]=[C:5]=[O:6]	[#6:1]-[NX3;H:2]-[C:5](=[O:6])[NX3;H:4]-[#6:3]
Urea_amine_acid	amine	acid	[#6:1][NX3;H2,H1;!$(NC=O):2]	[#6:5][CX3:3](=[O:4])[OX1H0-,OX2H1]	[#6:1]-[NX3;H:2]-[C:3](=[O:4])[NX3;H][#6:5]
Urea_amine_acid_chloride	amine	acid_chloride	[#6:1][NX3;H2,H1;!$(NC=O):2]	[#6:5][CX3:3](=[OX1:4])[F,Cl,Br,I]	[#6:1]-[NX3;H:2]-[C:3](=[O:4])[NX3;H][#6:5]
Urea_CDI	amine	amine	[#6:1][NX3;H2,H1;!$(NC=O):2]	[#6:3][NX3;H2,H1;!$(NC=O):4]	[#6:1][N:2]C(=O)[N:4][C:3]
Suzuki_aryl_aryl	boronate	aryl_bromide	[c:1]-B(O)O	[c:2]-Br	[c:1]-[c:2]
Suzuki_vinyl_aryl	vinyl_boronate	aryl_bromide	[C:1]=[C:2]-B(O)O	[c:3]-Br	[C:1]=[C:2]-[c:3]
Suzuki_aryl_vinyl	boronate	vinyl_bromide	[c:1]-B(O)O	[C:2]=[C:3]-Br	[c:1]-[C:3]=[C:2]
Suzuki_vinyl_vinyl	vinyl_boronate	vinyl_bromide	[C:1]=[C:2]-B(O)O	[C:4]=[C:3]-Br	[C:1]=[C:2]-[C:3]=[C:4]
ClickChem_azide1	azide	alkyne	[#6:1]-[NX2:4]=[NX2+:5]=[NX1-:6]	[#6:7]-[CX2:3]#[C:2]	[#6:1][n+0:4]1[n+0:5][n+0:6][c:3]([#6:7])[c:2]1
ClickChem_azide2	azide	alkyne	[#6:1]-[NX2-:4]-[NX2+:5]#[NX1:6]	[#6:7]-[CX2:3]#[C:2]	[#6:1][n+0:4]1[n+0:5][n+0:6][c:3]([#6:7])[c:2]1
ClickChem_aryl_amine2azide	aryl_amine	alkyne	[c:1]-[NX3;H2:2]	[#6:5]-[CX2:3]#[C:4]	[c:1][n+0:2]1[n+0][n+0][c:3]([#6:5])[c:4]1
ClickChem_alkyl_halide2azide	alkyl_halide	alkyne	[C:1]-[Cl,Br,I]	[#6:4]-[CX2:3]#[C:2]	[C:1][n+0]1[n+0][n+0][c:2]([#6:4])[c:3]1
SnAr_ForCl	amine	aryl_fluoride	[NX3;H2,H1;!$(NC=O):1]	[c:2]-[F,Cl]	[N:1]-[c:2]
Mitsunobu_ester	acid	alcohol	[CX3:1](=[O:2])[OX1H0-,OX2H1]	[#6:4][OX2H:3]	[#6:4][O:3][CX3:1](=[O:2])
Mitsunobu_ether	alcohol	alcohol	[#6:2][OX2H:3]	[#6:1][OX2H]	[#6:2][OX2:3][#6:1]
Mitsunobu_amine	alcohol	amine	[#6:1][OX2H]	[NX3;H2,H1;!$(NC=O):3]	[#6:1][N:3]
ChanLam	amine	boronate	[c:1]-B(O)O	[NX3;H2,H1:2]	[c:1]-[N:2]
reductive_amination	amine	aldehyde	[NX3;H2;!$(NC=O):1]	[#6:2][CX3H1:3](=O)	[N:1]-[CX4:3]-[#6:2]
Sulfonamide	sulfonyl_chloride	amine	[#16X4:1](=[OX1:2])([ClX1])=[OX1:3]	[NX3;H2,H1;!$(NC=O):4]	[#16X4:1](=[OX1:2])([N:4])=[OX1:3]
DessMartin_oxidation	alkyl_alcohol		[C;H2,H1:1][OX2H:2]		[C:1]=[O:2]
Nboc_protection	amine		[NX3;H2,H1;!$(NC=O):1]		[NX3:1]-C(=O)OC(C)(C)C
Nboc_deprotection	N-boc_amine		[NX3:1]-C(=O)OC(C)(C)C		[NX3:1]

ASKCOS[1] use in generative model and platform operations

After applying the templates in Table S1, the reactions are screened to make sure that the required conditions will be accessible on the platform. For example, reactions requiring gaseous or pyrophoric reagents cannot be performed safely and need to be removed. For each reaction required to make a generated candidate, the ASKCOS condition recommender produces a rank ordered list of reaction conditions. The conditions are then adjusted based on human curated rules to improve compatibility with the platform (for example substituting all palladium catalysts with the single palladium catalyst that is stocked in the platform library). The adjusted conditions are then reranked using the ASKCOS forward prediction model and the top predicted adjusted condition is selected by the platform. There are often reactions where several of the conditions are likely to work; the platform chooses to run the one with the highest score even if this may not be the true optimal condition. The reaction classes that are run were chosen in part because they are more robust to variable conditions than some other reactions; for example, the Buchwald-Hartwig coupling can be very sensitive to the identity of catalyst and base used and so this transformation was left out of the templates used in molecule generation.

The platform can autonomously retry reactions that failed using different conditions and can optimize reaction conditions for low yielding reactions. However, this process substantially slowed synthesis progress, and a retrospective analysis (performed prior to the start of this project) showed that it was often employed for reactions that were never going to have high yields (for example, electrophilic aromatic substitution reactions on deactivated substrates). For this study, we therefore chose to accept that some reactions would fail. We decided that products of failed reactions would be withheld from the MF-BO surrogate model instead of recording an activity score of zero because the surrogate model is not meant also to learn which molecules are synthesizable. In theory, a single model could learn each fidelity level and the synthetically accessible chemical space. However, that is beyond the scope of this study and a challenging topic in itself; see, for example reference [2].

Implementation of MF-BO

The MF-BO algorithm was integrated into the Bayesian Back End v0.9.0 (BayBE) from EMD-Millipore. The method makes use of the open source infrastructure in BayBE, such as the ability to run campaigns, track data, and generate molecule representations. MF-BO can be used in BayBE by importing a custom Surrogate, Recommender, and SubstanceParameter, all of which are provided in the project repository. The plots in Figure 2 can also be recreated with the scripts provided in the repository.

Simulated single point data were generated from the IC_{50} values in ChEMBL and the Hill Equation. The spreadsheets used to generate the data are provided in the data repository. The generation of single point data required making assumptions about the level of noise, limits of detection, error in liquid handling, error in concentration of prepared inhibitor, and hill coefficient value. The noise and lower limit of detection were determined based on the Tecan Spark plate reader used in this study and are both relatively small compared to other sources of uncertainty. The upper limit of detection, which requires measuring a small change in a large

amount of fluorescence contributes more uncertainty in measurement of weak inhibitors. The error in liquid handling was estimated based on the Tecan Freedom Evo LiHa pipetting arm and contributes 10-20% error in small (<2uL) volume pipetting required for the fluorometric assay. The error in concentration simulates using the optical absorption data from the HPLC and a model of molar extinction coefficient to determine the concentration of the new materials without having reference material available as a standard. The concentration at which the single point assay is (desired to be) conducted also had to be determined before generating simulated data; it was selected such that approximately half of inhibitors would have IC50 values above and half below the chosen concentration. Below (Figure S2) is a sample plot of simulated single point inhibition values versus IC_{50}. In general, the correlation between single point measurements and IC_{50} values was about $\rho = 0.75$. Unfortunately, the correlation did not vary in chemical space the way the correlation between docking score and IC_{50} did. If there were a principled way to simulate such a correlation it could be used to substantially improve the simulated data, rather than forcing the MF-BO algorithm to learn that the single point measurement correlation is not location dependent.

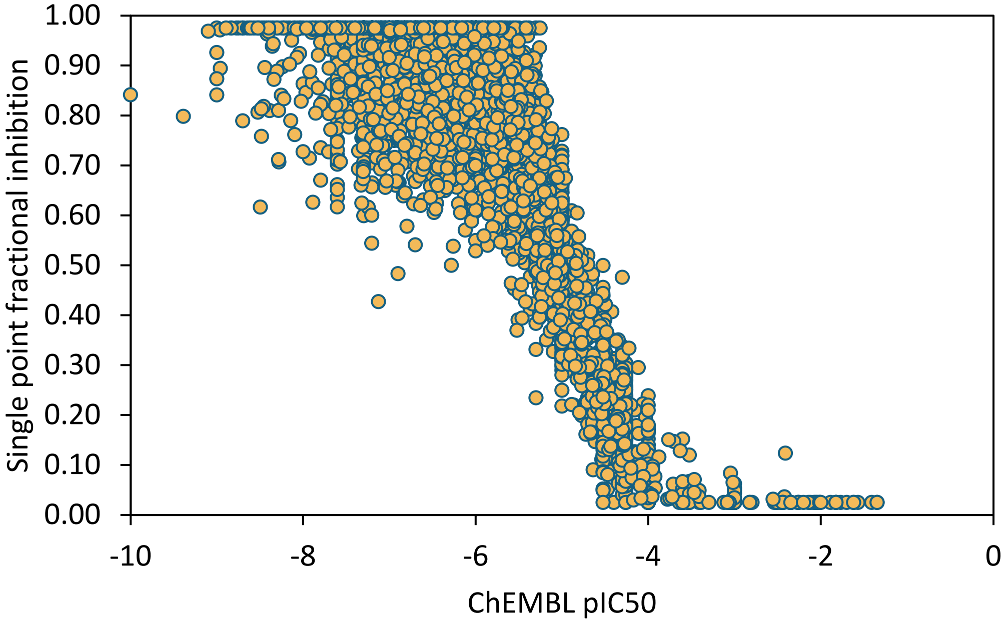

Figure S2. Simulated single point data based on log10 IC50 values from ChEMBL, the Hill equation, and the assumptions discussed in the paragraph above. Without any of the simulated uncertainty, noise, and limits of detection, the points would fall on a straight line.

Implementation of other search algorithms

Bayesian optimization using a single fidelity was implemented with an unmodified version of BayBE, using Morgan fingerprints, the same GP with Tanimoto kernel as MF-BO, the sequential greedy recommender (closest analog to MF-BO recommender) using expected improvement (EI), and a batch size equal to testing 2% of the dataset. The optimization campaign was randomly initialized with 5% of the dataset, just as the MF-BO optimization was. Since the dataset is all the highest level of fidelity, comparing cost to MF-BO is simple, a molecule is only considered (re)discovered if it is selected for evaluation at the highest fidelity.

The experimental funnel was implement in Python using the IC_{50} values for inhibitors of each target from ChEMBL, simulated single point data as discussed in the previous section, and docking scores (DiffDock default confidence score) from docking all of the inhibitors using DiffDock. Since the experimental funnel is deterministic (compared to MF-BO and BO, which are initialized with a random 5% of the dataset), for each independent run of the experimental funnel, an initial dataset was created by randomly sampling half the full dataset for each protein target. All of the molecules in the initial dataset were docked, and ranked by their docking score, this initial set of docking experiments counted towards the 5% initialization budget of the experimental funnel, not the budget for active optimization (for comparison to MF-BO and single fidelity BO). The top-P percent of molecules based on docking scores were then measured at the single point fidelity, which has a much stronger correlation with IC_{50} than docking scores do. From this set of single point data, the same top-P percent of molecules were measured at the highest fidelity. The percentage, P, was tuned so that, on average, at each iteration an amount of budget equivalent to testing 2% of the dataset at the highest fidelity was expended. This fixed the proportion of budget spent at each iteration on single point and dose response measurements. If the correlation between experiments was very high, the funnel performed very well.

The transfer learning enhanced funnel was inspired by the work of Buterez et al. (Nat. Comm. 2024) but was implemented using Chemprop to implement the graph neural network as a message passing neural network. The workflow was the same as the experimental funnel, but at each iteration, a Chemprop model was trained to predict single point inhibition percentages and IC_{50} values based on the docking scores, single point inhibitions, and IC_{50} values that had already been measured. That model was used to predict scores for all the unselected molecules at both the single point and IC_{50} fidelity, and the molecules were then rank ordered by their predicted scores. The top-P percentage of molecules was then selected, with P being tuned so that on average 2% of the budget was expended on each iteration, and those molecules were added to the measured sets in the same manner as the normal experimental funnel. Before starting the next iteration, the chemprop model was retrained with the expanded measured set to improve its predictive power.

For random selection, molecules were randomly selected to be measured at the highest fidelity.

Details MF-BO performance analysis

The CXCR4 target has decoys available in the DUDE-Z dataset. These are molecules that sample the same properties as real CXCR4 ligands (charge, molecular weight, etc.) but are

topologically distinct from real CXCR4 inhibitors. As such, they are expected to have negligible inhibitory activity, so for a retrospective analysis their single point and dose-response values were set to 0. DiffDock was still used to dock the decoys to test both the robustness of the docking algorithm and how MF-BO handles large numbers of inactive molecules. The dataset size was kept constant at 600 molecules, datasets contained 0%, 25%, 50%, and 75% decoys. The results of MF-BO and traditional BO are shown in Figure S3. Linear designs of experiments were not considered because it is very difficult to compare the experimental expenditure required for linear designs that sample the same fraction as the iterative designs do at each iteration (2% of budget spent at each iteration). Both MF-BO and BO consistently beat random sampling. MF-BO tended to outperform BO, although this was not the case for the top-1%. There is no clear trend with fraction of decoys and fraction of top performers found.

A visualization of the search spaces using tSNE showed that the decoy molecules do not cluster with the real inhibitors or with each other, see Figure S4. Bayesian Optimization is expected to work well in these scenarios because most of the landscape is flat, so once a molecule near an optimum is found, the algorithm quickly finds other nearby top performers. Contrast the flat landscape of decoys with the ragged landscape of real molecules, and the explorative propensity of MF-BO leads to a larger enhancement.

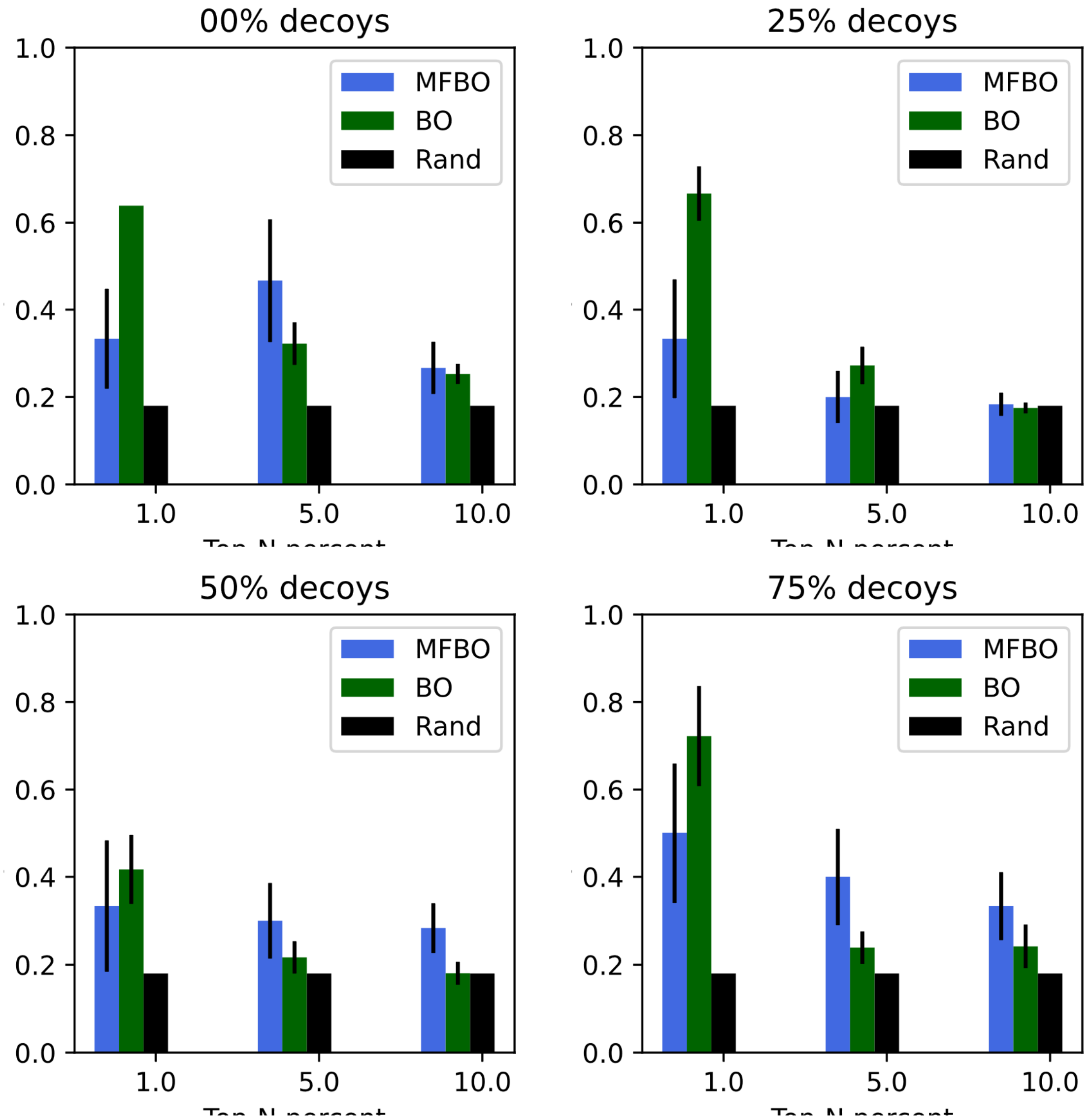

Figure S3. The fraction of top-N% performers found for different values of N, for MFBO, BO, and random sampling against CXCR4 datasets containing decoy molecules from the DUDE-Z set.

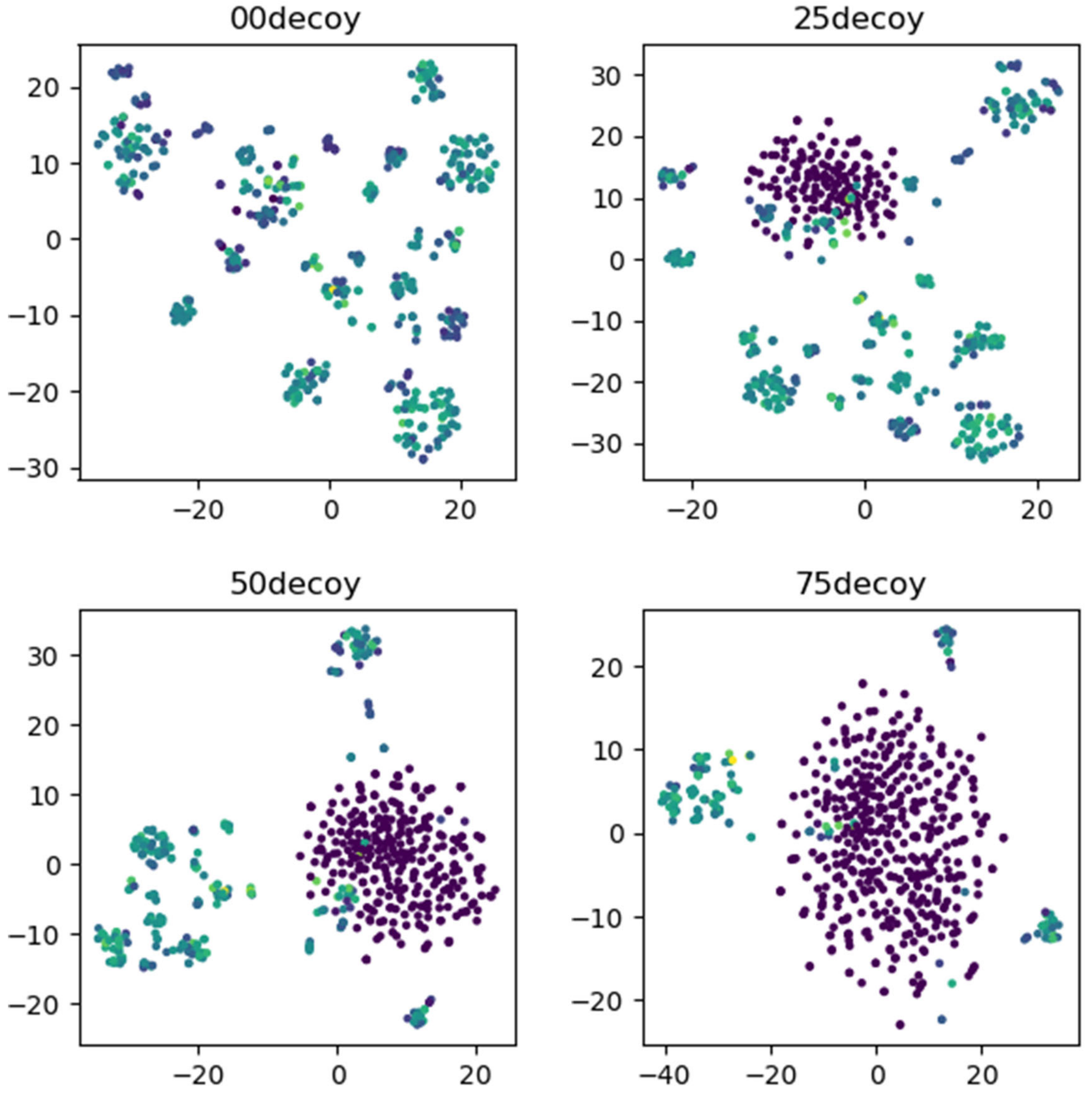

Figure S4. The tSNE visualization of the datasets with decoys. Decoy molecules are represented by dark blue points, all other molecules are colored by their potency, with the most potent molecules colored yellow.

Details MF-BO performance analysis

The diversity is measured as one minus the average Tanimoto Similarity (TS). TS measures the fraction of bits that need to be edited to transform one molecule's bit-fingerprint into another's. While in principle TS should scale from 0 to 1, hashing of fingerprints means even very similar molecules will have values much less than 1. Despite having a small range of values, TS has been shown to correlate well with distances in chemical-property spaces. For example, benzene and fluorobenzene have a TS = 0.33 and trichostatin A and vorinostat (a similar FDA approved HDAC) have a TS = 0.22, while benzene and trichostatin A only have a TS = 0.03. In this context, a difference in average diversity (which is on the same scale as similarity) from 0.85 to 0.9, as in Figure 2B, is quite substantial.

The correlation was quantified using the average of pairwise Spearman's rank correlation coefficients between all fidelities. This metric quantifies non-linear correlations, which is necessary when comparing assays with different ranges: docking score derived from energy $(-\infty, \infty)$, single point inhibition $[0, 1]$, and concentration $(0, \infty)$. The surrogate model learns the result of each assay for a given molecule *and* the correlation between assays for a given molecule. For example, trichostatin A scores highly in docking, single point, and dose-response assays, indicating that for trichostatin A and similar molecules all three fidelities are highly correlated, while compound F (Figure 4C) scored well in docking but poorly in the single point assay, indicating that molecules like F have low correlation between assays. This varying correlation across chemical space manifests as a weak overall correlation when quantified using a scalar like Spearman's rho. If no families of molecules ever show any correlation between fidelities, the correlation coefficient will be very near to zero, and MF-BO will not be useful, only testing the highest fidelity using BO is productive

To make sure that the procedure for generating datasets with defined diversity and correlation produces the same results regardless of the protein target, we also performed experiments on structured datasets of PARP1 and Factor-D. We did not analyze all targets in this manner because the number of compounds was insufficient for the CXCR4, NR1A2, and HIF-PH, datasets. We ran the same experiment as for Acetylcholinesterase. Each set was clustered using dynamic k-medoids resulting in 25 and 15 clusters, and subsets of 400 and 240 molecules, for PARP1 and Factor-D respectively. The trends agree with those in Figure 2B & C and are shown in Figure S5. The Factor-D analysis shows the difficulty in building structured subsets from such a small starting set; the subsets sampled from only 3 of the 15 clusters show very low diversity, unrealistic for actual drug exploration.

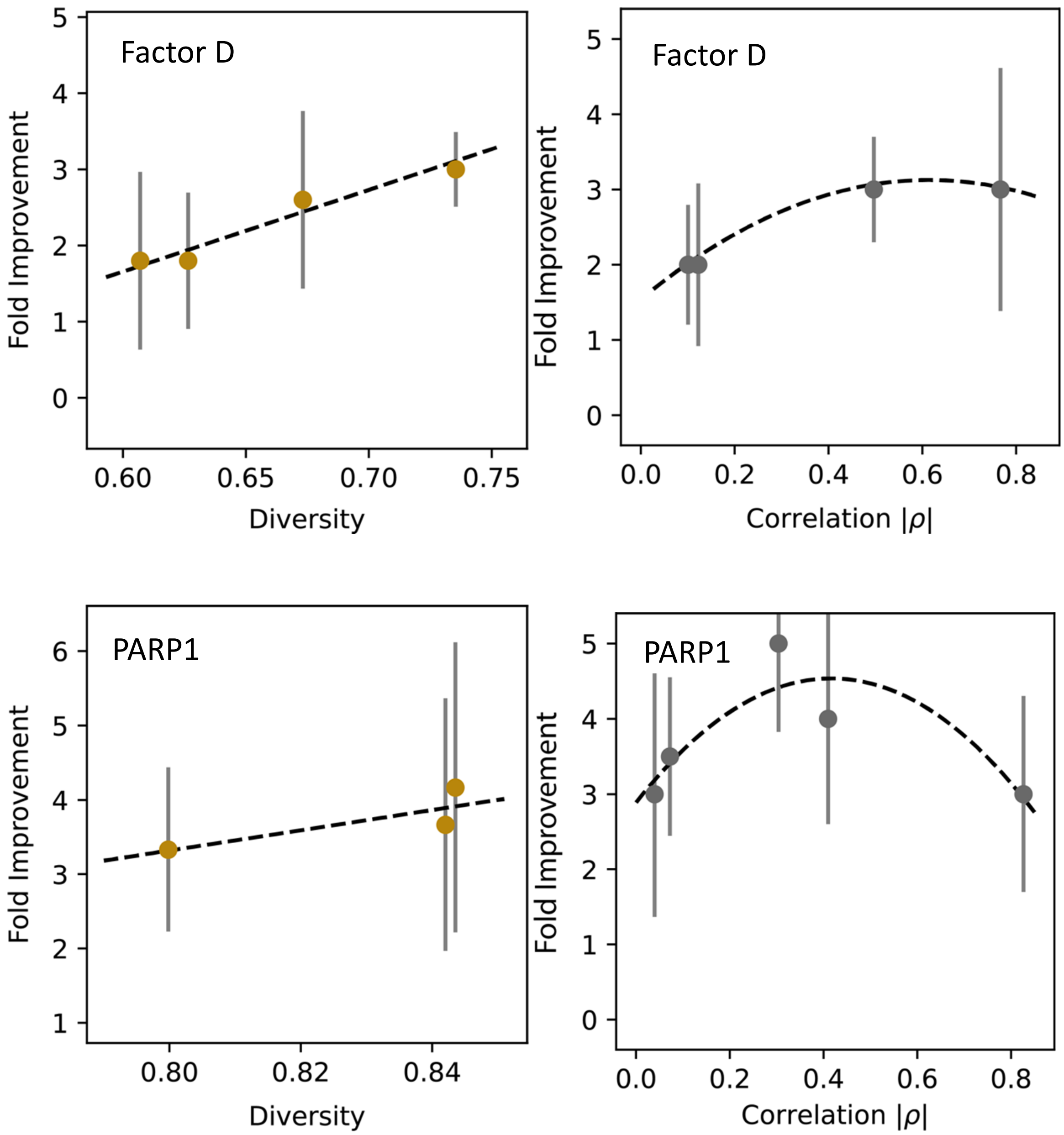

Figure S5. Performance of MF-BO on structured subsets of the Factor-D (top) and PARP1 (bottom) datasets. The figures were generated following the same procedure as outlined for Figures 2B and C in the main text.

Details of Docking, DiffDock implementation, and DiffDock Validation

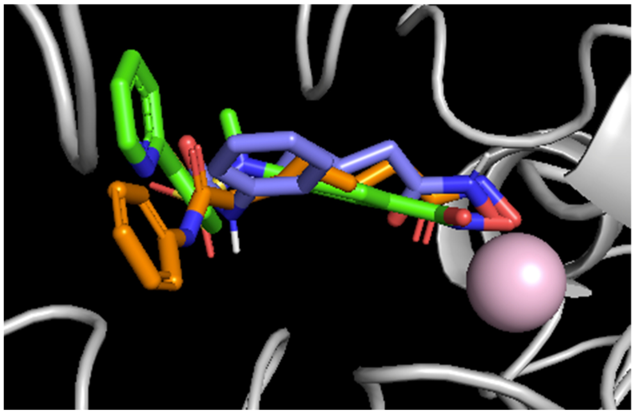

Figure S6. Comparison of DiffDock pose (orange), Vina pose (lavender), and crystallized ligand (green) for vorinostat in HDAC8. Both docked poses closely coordinate the catalytic zinc (pink) and overlap considerably with the crystallized ligand.

DiffDock was selected as the docking program because it outperforms Vina in terms of both pose accuracy and speed. DiffDock is a machine learning model that uses reverse diffusion to learn how ligands typically dock to proteins. Vina is a physics-based model that tries to approximate how ligands interact with proteins with coarse grained force fields. When tested on a subset of the PDBbind dataset, DiffDock shows a lower RMSD than Vina, with 38% of the top ranked ligand poses from the PDBbind set showing an RMSD < 2 Å while only 5.5% of top ranked ligand poses from Autodock Vina having an RMSD < 2 Å.

Figure S6, above, shows vorinostat (an FDA approved HDACI) docked by both DiffDock and Vina, as well as the bound ligand in the crystal structure. Both docked poses clearly show strong interaction between the catalytic zinc and hydroxamate moiety of vorinostat, with some minor differences in how the conformation of the flexible aliphatic part of vorinostat. Figure S7, below, shows the correlation between DiffDock score and Vina score for HDACIs with reported IC_{50} values. Figure S8 compares the IC_{50} values with DiffDock scores and Vina binding energies. The DiffDock score indicates how confident the model is that the diffused pose is the same as the crystal structure ligand pose, while the Vina binding energy is a summation of the calculated interactions of the ligand with the binding pocket. The two scores are therefore not directly comparable, however the Spearman correlation coefficient between them is 0.39, indicating moderate correlation.

For this study, we did not use the DiffDock score directly, because the pose produced by the DiffDock contains more information than the scalar representing model confidence (the same could be applied to poses generated with Vina). Instead, we reanalyzed the pose by calculating the overlap between the docked candidate and the binding pocket of HDAC8 and adding a term for the proximity of a hydrogen bond donating or accepting group to the catalytic zinc. The overlap was found by comparing the docked pose to all known holo structures and the different ligands they contain (PDB reference codes: 1T64, 1W22, 2V5X, 5BWZ, 5D1B, 5DC5, 5FCW, 6HSK, 7JVU). We found that this revised score was better correlated to the pIC_{50} value than the binding score alone. ($\rho = -0.12$ vs $\rho = -0.08$), although neither is a strong predictor of IC_{50}. The choice to target molecules that would also bind the catalytic zinc allows for easier

comparison to the test molecule trichostatin A but may have excluded some novel therapeutics that work by allosteric inhibition or active site tunnel blocking.

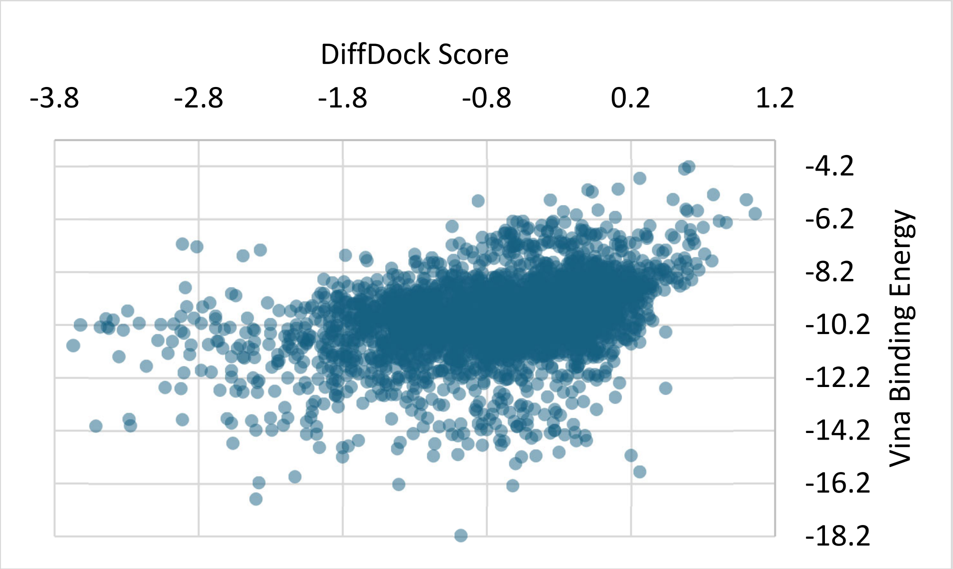

Figure S7. Scatter plot of DiffDock and Vina scores for HDACIs reported in the literature.

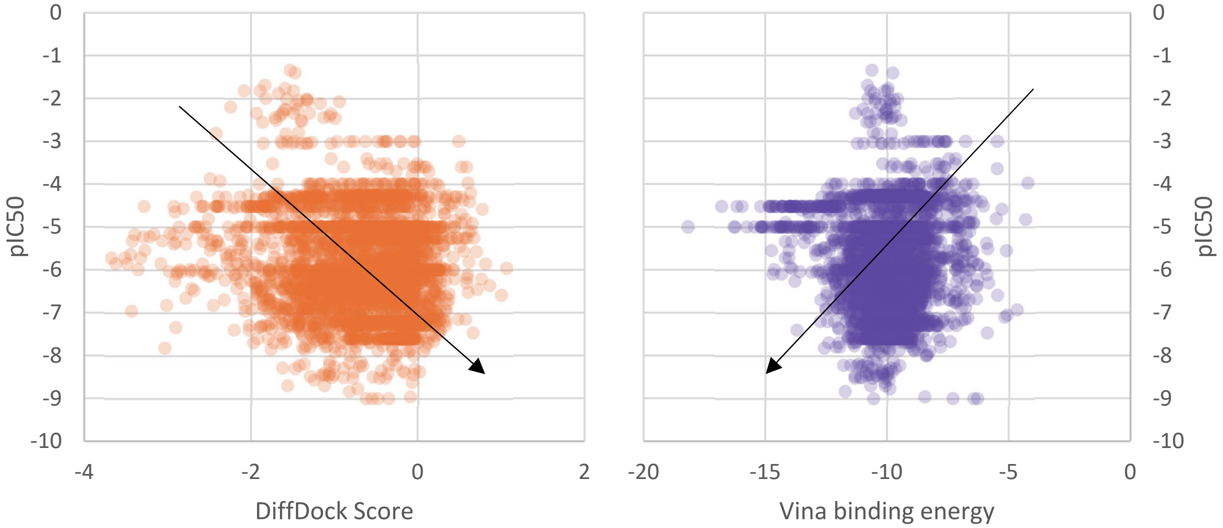

Figure S8. Neither DiffDock nor Vina provide an obvious indication of which molecules are top performers in terms of pIC_{50}. Left, scatter plot of DiffDock scores for HDACIs with published IC_{50} values. Right, scatter plot of Vina binding energies for the same HDACIs. Arrows indicate the direction of the expected trend; higher DiffDock scores and larger magnitude (but negative sign) binding energies should correlate with higher potency (smaller pIC_{50})

Comparison to other surrogate model architectures and molecule representations

We examined several different surrogate models and molecular representations. The models tested were Random Forest (RF), Natural Gradient Boosting (NGBoost), and Gaussian Process models (GP). For RF and NGBoost, an ensemble of models were bootstrapped to predict the mean and variance needed for targeted variance reduction (TVR). We tested three different representations for drug molecules, Morgan fingerprints (MFP, radius 2, 1024 bit),[3] Mordred descriptors (>1800-dimension),[4] and Mol2vec embedding (default model, 300-dimension).[5] The kernel used for the GP models depended on the molecule representation; for Mordred and Mol2vec, which are vector representations, a Matern 5/2 kernel was used, but for Morgan fingerprints, which are sparse bit vectors, a Tanimoto kernel was used to describe the distance between molecules in the same manner as the familiar Tanimoto Similarity.[6] Figure S9 compares the different molecular representations' performance using a GP, measured by the cumulative best inhibitor found versus number of batches, where each batch corresponds to 5% of the total dataset. The HDAC8 data from ChEMBL was used since HDAC was the original target when developing the MF-BO method.

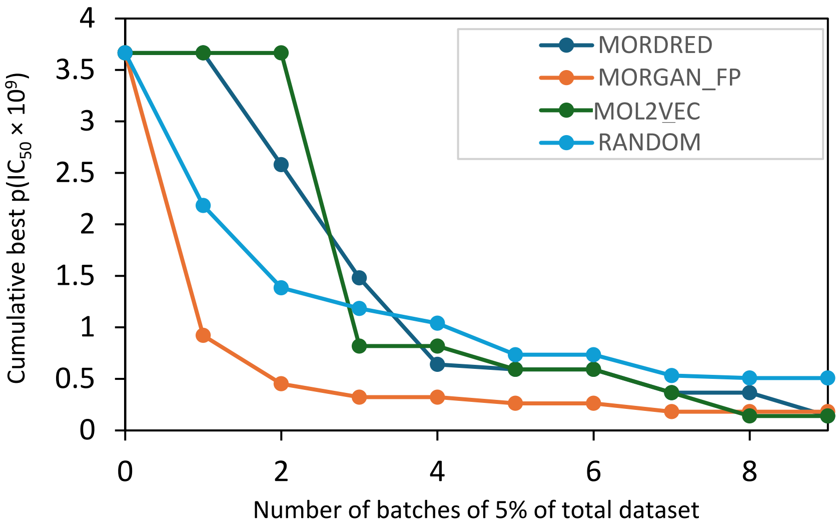

Figure S9. comparison of different molecular representations used with a GP.

Using only the high-fidelity data (IC_{50} values) from the HDAC dataset, a GP model using Morgan fingerprints consistently outperformed the other models and representations. This finding was surprising as Morgan fingerprints are the smallest representation (1024 bits, vs 1800, vs 300 dimension floating point vectors) and Mol2vec has previously been reported to outperform Morgan FPs in a variety of prediction problems. We hypothesize that the Tanimoto kernel, which is not isotropic, more accurately captures the "closeness" of molecules compared to the Matern kernel. Regardless, we assumed that since the GP-MFP model performed best on the highest fidelity, it would perform well in the multi-fidelity setting, and therefore only consider the GP-Morgan fingerprint model in developing and evaluating multi-fidelity BO. A comparison of the different model architectures, all using the Morgan fingerprint, is shown in Figure S10.

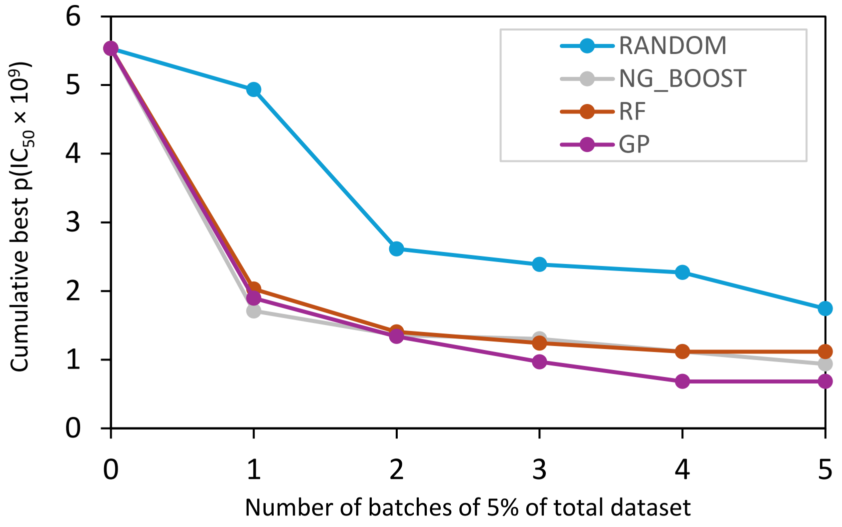

Figure 10. Comparison of different model architectures using Morgan fingerprints.

Other combinations of model and fingerprint were attempted, however none outperformed the combination of Morgan fingerprint and Gaussian process on the ChEMBL HDAC dataset. On the other ChEMBL datasets this combination was still top performing, although in isolated cases other combinations performed better.

Characterization of literature datasets

Datasets were downloaded from CHEMBL and filtered to remove missing data and outliers. The following histograms (Figure S11) show the distribution of Lipinski's Rule of Five properties for each dataset: Molecular weight (Mol. Wt.), water-octanol partition coefficient (logP), number of hydrogen bond acceptors (# H acc.), and number of hydrogen bond donors (# H don.).

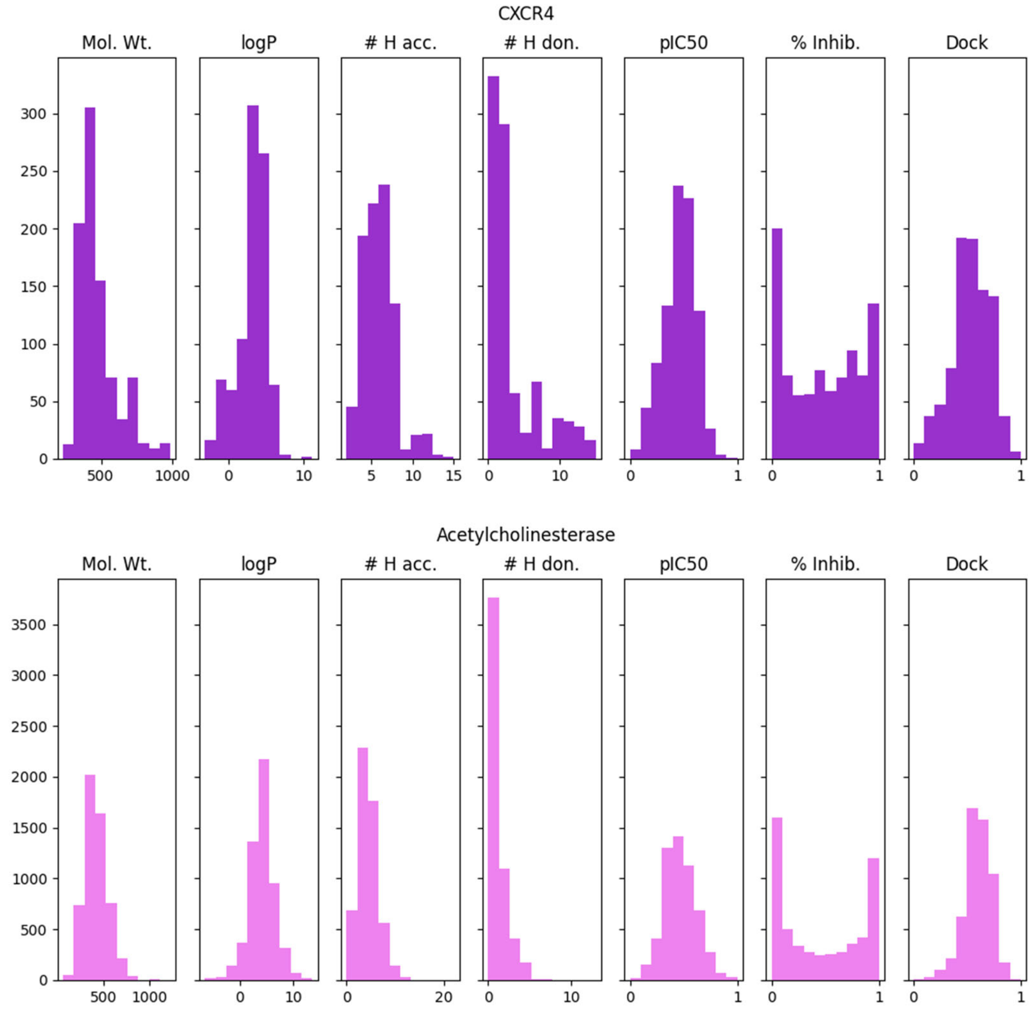

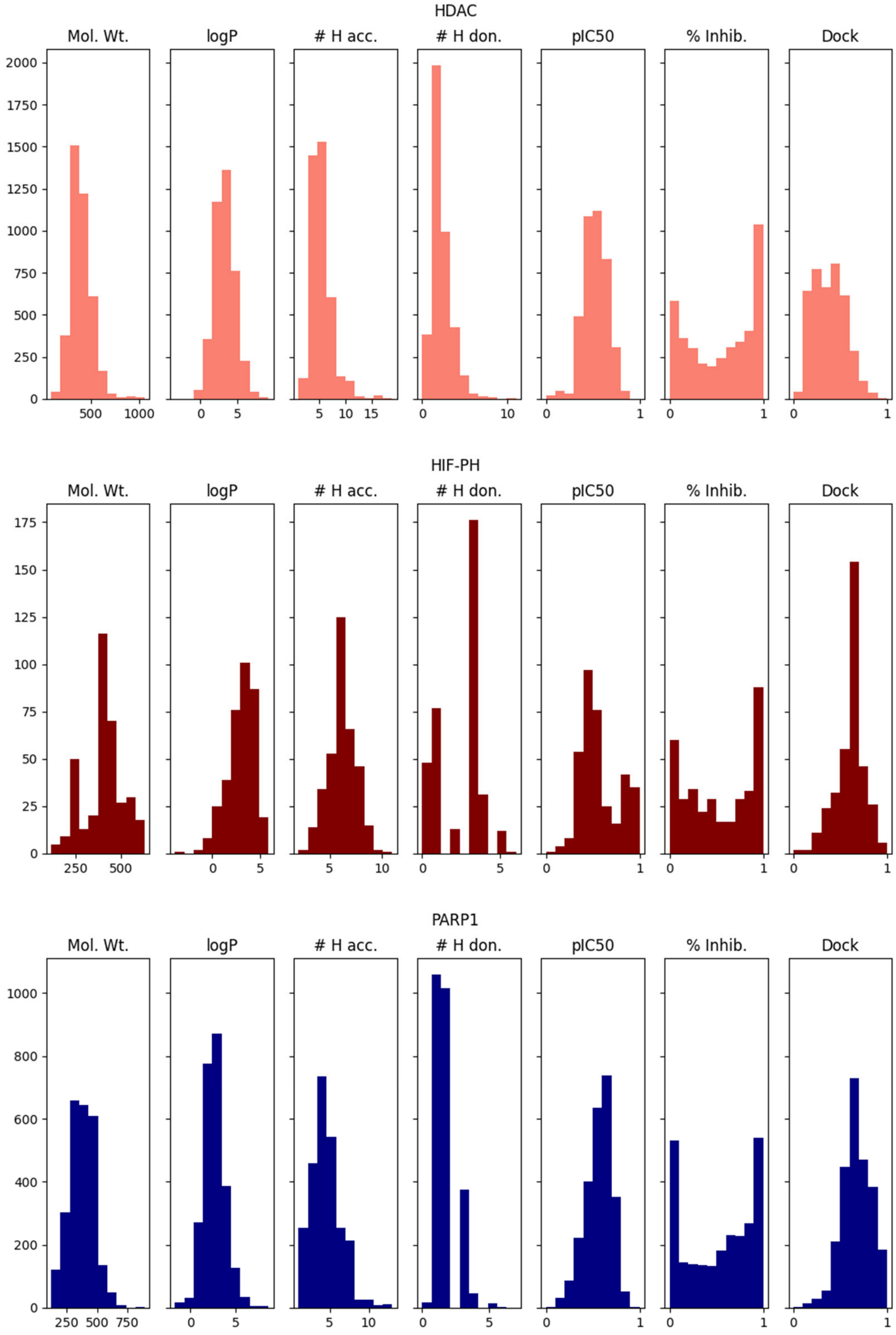
HDAC
Mol. Wt.
logP
H acc.
H don.
pIC50
% Inhib.
Dock
HIF-PH
Mol. Wt.
logP
H acc.
H don.
pIC50
% Inhib.
Dock
PARP1
Mol. Wt.
logP
H acc.
H don.
pIC50
% Inhib.
Dock

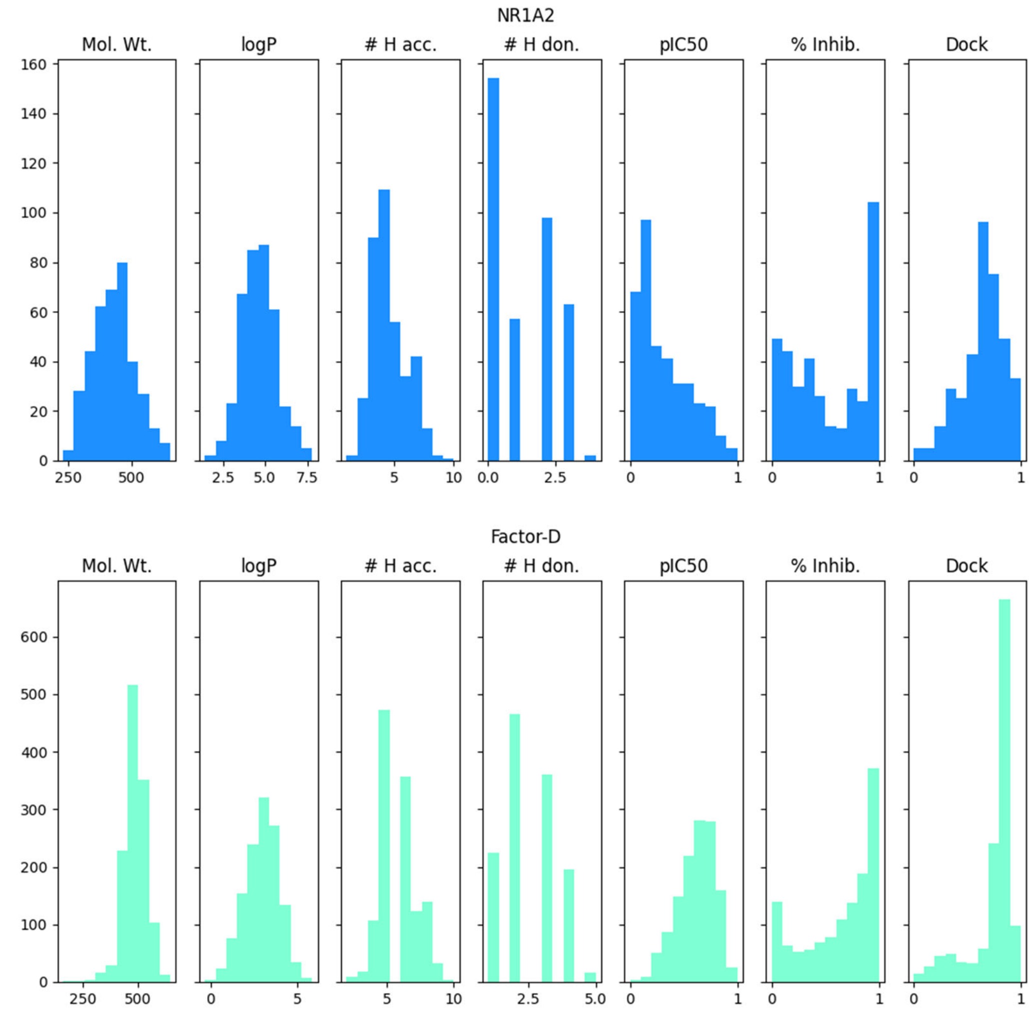

Figure S11. Summary statistics for each of the datasets collected from CHEMBL.

The datasets are also visualized using tSNE projection of the molecules' fingerprints onto two dimensions. Clustering is visible for each dataset in Figure S12, those where clusters are mostly the same color have the same IC_{50} value for each molecule within the cluster (HIF-PH and NR1A2) and tend to be least well suited for MF-BO, while those showing less homogeneity within each cluster perform well. While this is a useful post hoc analysis, cluster homogeneity is not known beforehand.

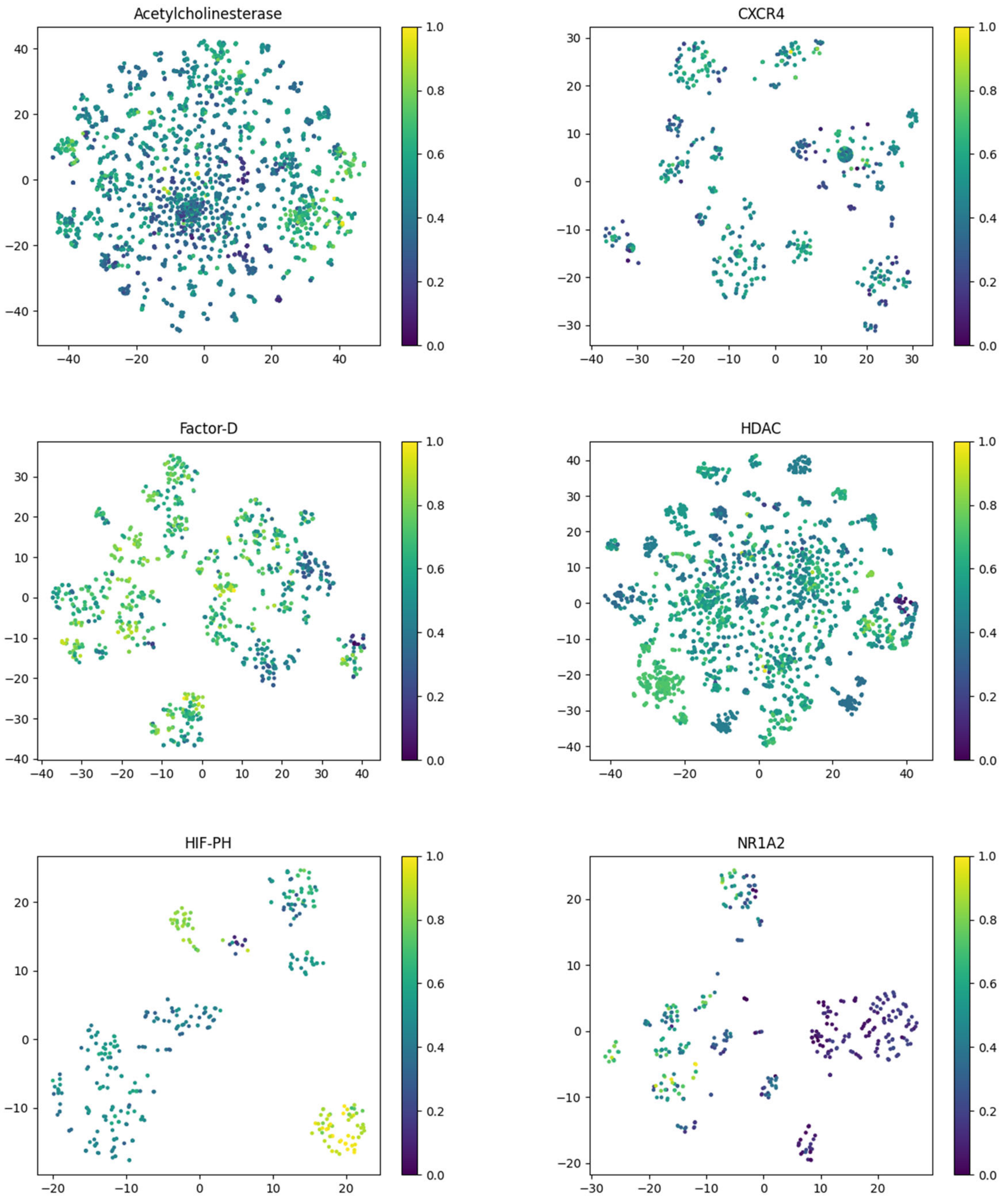
Acetylcholinesterase
CXCR4
Factor-D
HDAC
HIF-PH
NR1A2

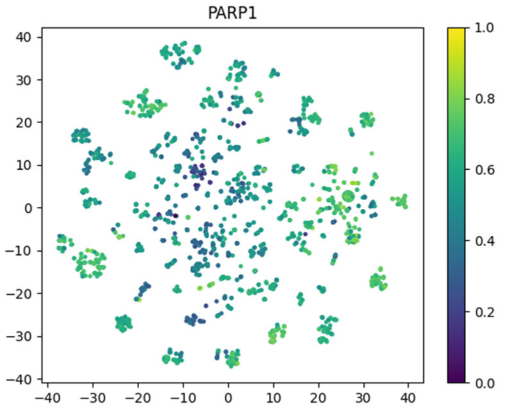

Figure S12. tSNE visualizations demonstrating the degree of clustering in each dataset. The colorbars show pIC_{50} values scaled from 0 to 1. Those datasets that are more tightly clustered, with top performing molecules contained within a single cluster, tend not to be suitable for MF-BO.

Dataset	Size, n
Factor D	1236
CXCR4	891
PARP1	2530
HIF-PH	358
NR1A2	374
Acetylcholinesterase	9981
HDAC8	3990

Details of the autonomous platform

The autonomous platform is described in detail in Koscher et al.[7] Briefly, it consists of a liquid handler, HPLC-MS, plate reader, benchtop NMR, robot arm, storage carousel, and peripheral reactors for specialty chemistry. The platform was designed to autonomously explore a chemical space defined by a molecular scaffold (or scaffolds) and suite of properties. It automatically generates candidate molecules and plans routes to their syntheses. Using what it learned from the successfully synthesized molecules, the platform started the process over, generating and selecting new candidates, attempting their synthesis and purification, and measuring a new slice of biochemical space, all without operator guidance (operators only intervened to provide needed chemicals or correct errors, which almost always had a root cause of operator error).

In this work, we changed the molecular generator to generate molecules and synthesis plans at the same time, specifying which types of reactions we would allow, to address the low reaction success rate previously reported. We ended with a reaction success rate of >70% and a synthesis success rate of 58% (since multiple reactions are required to reach a product) across two iterations. This work also differs in that rather than weighing multiple properties the platform selects experiments based on different fidelities of measurement of the same property. The genetic algorithm was used in place of the previous molecular generator and synthesis planner, and the MF-BO design of experiments was used in place of the scalarization of the multiple factors weighed in previous experiment selection. In this manner, the prospective use of the MF-BO design was also largely autonomous.

Substantial changes/additions to the platform were made to support measuring drug properties as opposed to the optical properties previously investigated. The HPLC-MS data analysis was refined to estimate the concentrations of drug-like molecules without calibration, as discussed in McDonald et al.,[8] as opposed to dye-like molecules. The liquid handler was also trained to run a fluorometric assay which required dosing several chemicals with specified incubation periods at 37 °C in between, requiring complex orchestration not previously demonstrated; the liquid handler is free to work on other tasks during the incubation periods.

The platform crudely scales up syntheses by performing them in multiple wells and pooling each well come time for analysis. In the future, we wish to couple this screening

platform to an automated flow optimization platform, which can scale efficiently optimize single reactions and then produce arbitrary quantities of material by running for longer times.

Unfortunately, the platform is not able to distinguish isomers (or more generally isobars), and it assumes that the expected outcome of the reaction is actual outcome. We have checked several of the reactions manually scaled up with NMR and have found that the expected compound and reaction product are almost always the same (see below). This is expected, as the reaction reranking performed with the ASKCOS forward prediction model will catch instances where reactions have poor regioselectivity and filter them out. However, one counter example we found by NMR is compound I in Figure 4, which is actually a 5:4 ratio of cis:trans isomers.

The platform ensures reproducibility by maintaining detailed records[7] of both tasks and exactly how the task was executed and by propagating known uncertainty in each operation.[8] For example, a task might be "react 15 umol of A and B to form C" (i.e. a reaction from the molecular generator), while the detailed execution is a highly structured record that includes the amount of stock solution dispensed for each A and B to get 15 umol, reagent(s) required to run the reaction and their stock solution volumes, the solvents the stock solution included, the amount of additional solvent added to top off the reaction, the order and timing of addition for each component, the temperature profile the well plate experienced throughout the reaction, and every step that accompanied the workup of the reaction. The uncertainty in pipetting and liquid handling has also been quantified and is carried through to estimates of the concentration of all species. While the detailed record and quantified uncertainty do not guarantee reproducibility, they allow a human to easily recreate the exact experiment that gave those results to check reproducibility. In the future, the platform could be updated to automatically audit a sample of results to check that all calibrations are still appropriate.

Details of automated operations (Figure 4A labels)

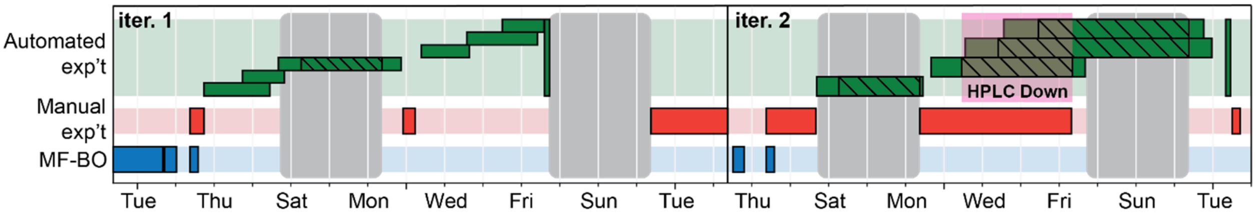

Figure S13. The timeline of automated operation during the MF-BO campaign to discover new HDAC inhibitors.

Figure S13 shows the timeline from main text Figure 4A. The details of each block, reading left to right, are in the tables below.

Automated experiments (green bars)

#	Iteration	Operation(s)	Notes
1	1	Rxn plate 1	
2	1	Rxn plate 2	
3	1	Rxn plate 3	Paused in between reaction workup and HPLC-MS assay for weekend
4	1	Rxn plate 4	
5	1	Rxn plate 5	
6	1	Rxn plate 6	
7	1	Single pt. assay	
8	2	Rxn plate 1	Paused after HPLC-MS assay for weekend
9	2	Rxn plate 2	HPLC breakdown during processing
10	2	Rxn plate 3	Run reaction despite HPLC backlog
11	2	Rxn plate 4	Run reaction despite HPLC
12	2	Single pt. assay	

Manual experiments and platform operations (red bars)

#	Iteration	Operation(s)	Notes
1	1	Stock platform	
2	1	(Re)Stock platform	
3	1	Manual review of exp't outcomes	Analysis of poor candidate synthesis success rate, Analysis of outliers in single pt. assay (due to fluorescent candidates)
4	2	Stock platform	Required manual synthesis of 3 starting materials out of stock
5	2	Dose response assay	Manual synthesis, purification, of 8 candidates (only 5 successful), and preparation for automated assay
5.5	2	HPLC repair (magenta)	Troubleshooting failure of instrument to inject consistent quantity of material; replacement of autosampler needle seal
6	2	Review of exp'ts	Manual processing to extract IC_{50} from dose-response data

MF-BO operations (blue bars)

#	Iteration	Operation(s)	Notes
1	1	Generate candidates	8 independent runs, sampled from same reactions and building blocks. Combined all final candidates for MF-BO selection
2	1	Run MF-BO selection	Selected candidates based on budget discussed above
3	1	Filter selected candidates	Based on ASKCOS predicted synthesizability AND availability of starting materials for selected candidates (chemical pool is not synchronized with chemical supplier inventory)
4	2	Run MF-BO	With data from iteration 1 and ChEMBL
5	2	Filter selected candidates	Based on ASKCOS and availability of starting material (three popular starting materials manual synthesized)

Synthesis and 1H NMR Spectra from select scaled-up molecules

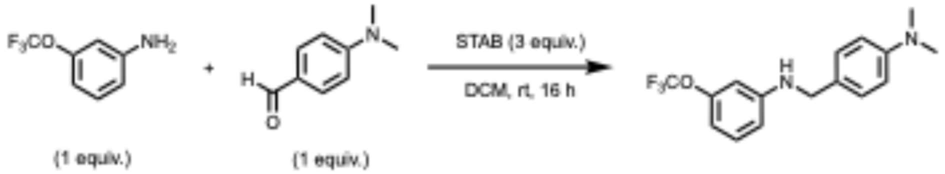

Synthesis of *N*,*N*-dimethyl-4-(((3-(trifluoromethoxy)phenyl)amino)methyl)aniline:
To a 20 mL vial equipped with a magnetic stir bar, 4-(dimethylamino)benzaldehyde (169 mg, 1.13 mmol, 1.0 equiv.) was dissolved in 6 mL of dichloromethane, and then 3-(trifluoromethoxy)aniline (151 µL, 1.13 mmol, 1.0 equiv.) was subsequently added to the solution. After 15 minutes of stirring, sodium triacetoxyborohydride (STAB, 719 mg, 3.39 mmol, 3.0 equiv.) was added in one portion, and the solution was stirred at room temperature for 16 h. The crude mixture was diluted with water, extracted with dichloromethane (x2), washed with brine (x1), and concentrated under reduced pressure. A column was run with 10% ethyl acetate: hexanes, and the column tubes containing the product were collected and concentrated under pressure to afford the product as a white solid (187 mg, 53% yield).
^{1}H NMR (400 MHz, $CDCl_3$): δ 7.25 – 7.20 (m, 2H), 7.14 (t, *J* = 8.1 Hz, 1H), 6.77 – 6.70 (m, 2H), 6.53 (m, 2H), 6.46 (m, 1H), 4.18 (s, 2H), 4.03 (bs, 1H), 2.95 (s, 6H).

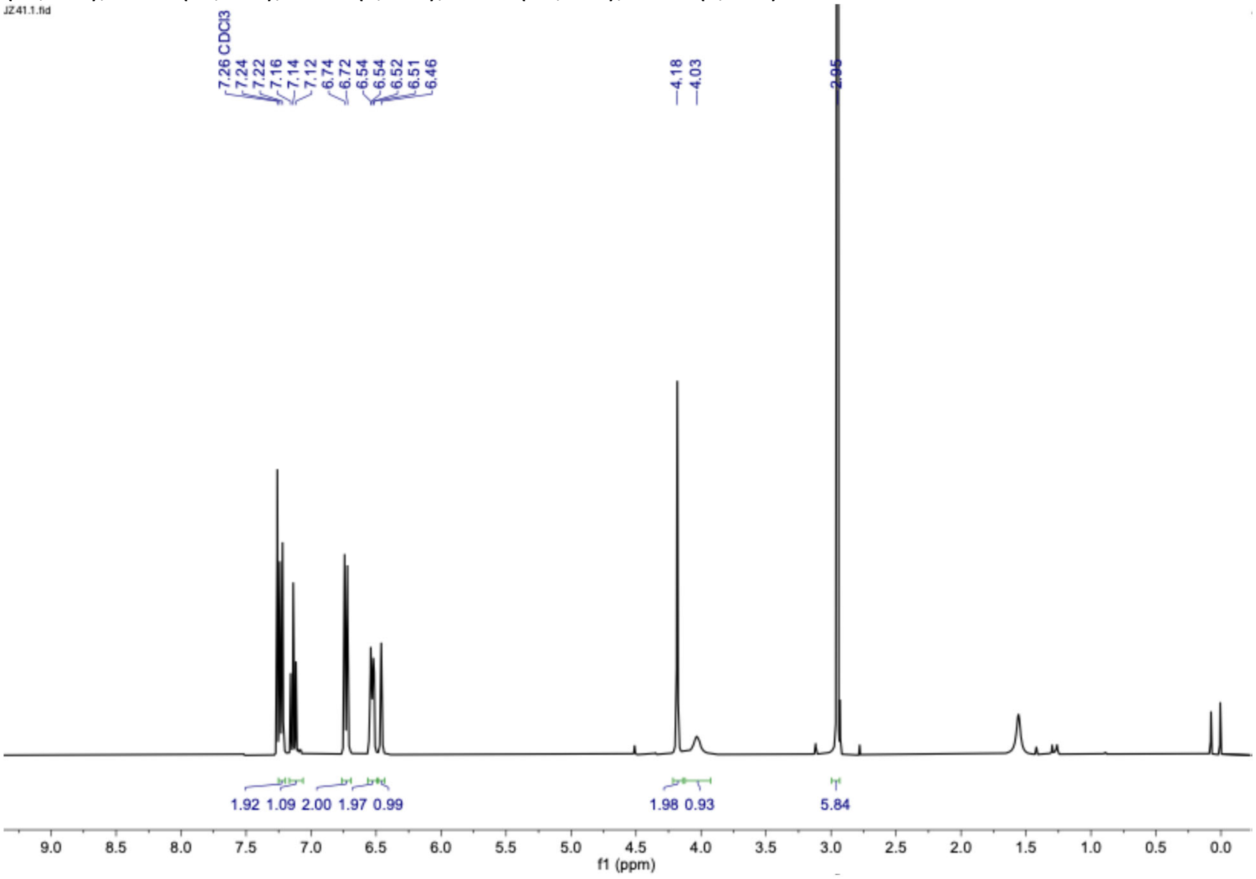

Figure S14. 1H-NMR spectrum of N,N-dimethyl-4-(((3-(trifluoromethoxy)phenyl)amino)methyl)aniline

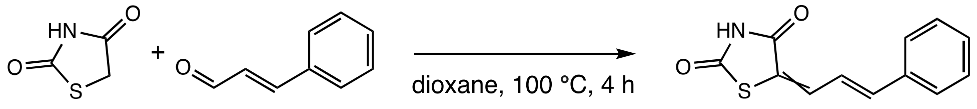

Synthesis of 5-((E)-3-phenylallylidene)thiazolidine-2,4-dione:

To a 1-dram vial equipped with a magnetic stir bar, thiazolidine-2,4-dione (77 mg, 0.66 mmol, 1.0 equiv.) was dissolved in 1 mL of 1,4-dioxane, and then *trans*-cinnamaldehyde (83 μL, 0.66 mmol, 1.0 equiv.) was subsequently added and the solution was stirred at 100 °C for 4 h. Half of the crude mixture was loaded on to a preparative TLC plate. Preparative TLC was run with 50% ethyl acetate: heptanes, and the plate band containing the product was collected, rinsed with ethyl acetate, and the rinse concentrated under pressure to afford the product as a yellow solid (45 mg, 53% yield). NMR revealed the product to be a 5:4 ratio of *E*:*Z* isomers at the 5-position of the thiazolidinedione ring.

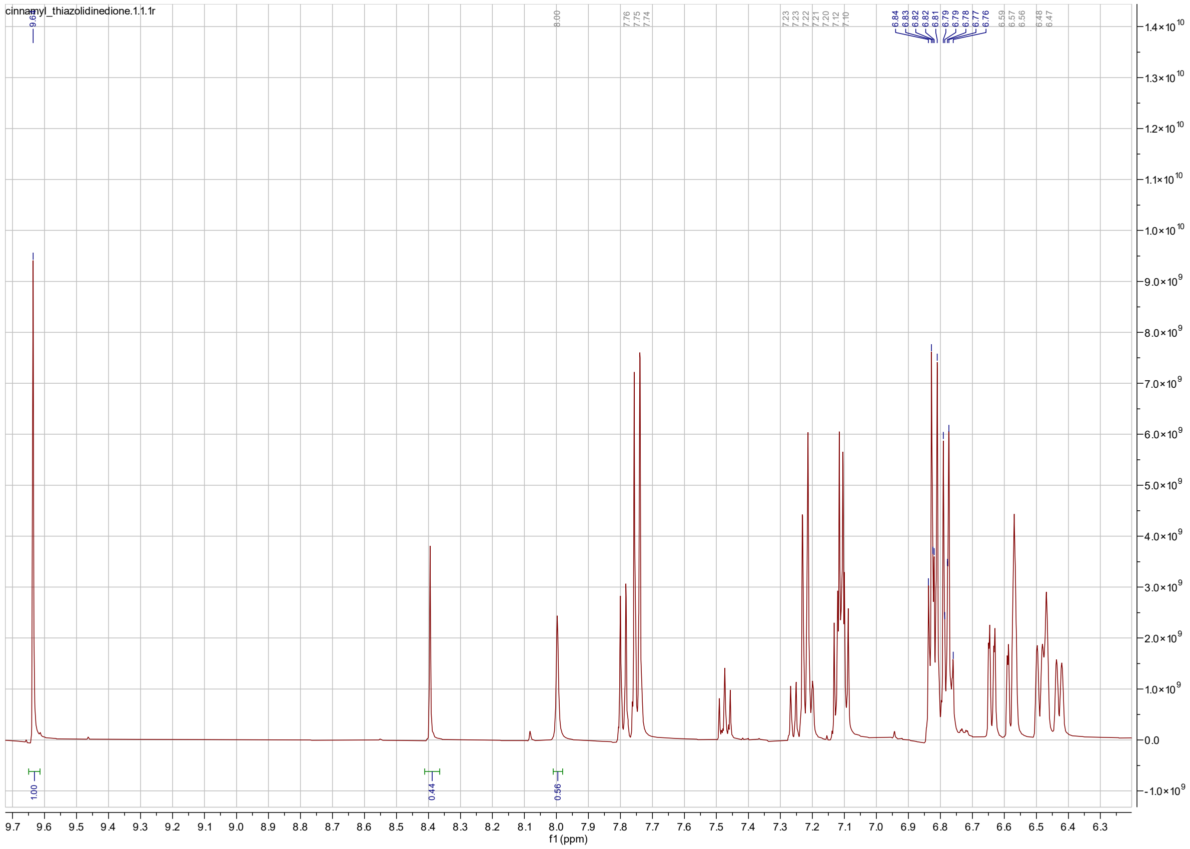

Figure S15. 1H-NMR spectrum of 5-((E)-3-phenylallylidene)thiazolidine-2,4-dione.

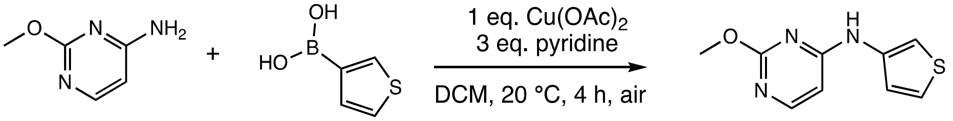

Synthesis of 2-methoxy-*N*-(thiophen-3-yl)pyrimidin-4-amine:
To a 1-dram vial equipped with a magnetic stir bar, 4-amino-2-methoxy-pyrimidine (49 mg, 0.39 mmol, 1.05 equiv.) was dissolved in 2 mL of dichloromethane, and then 3-thienylboronic acid (47 mg, 0.37 mmol, 1.0 equiv.) was subsequently added and the solution was stirred at 20 °C for 15 minutes. Then, 100 μL of pyridine (3.3 equiv.) and 100 μL of saturated aqueous $Cu(OAc)_2$ solution (1.1 equiv.) were added. The solution was stirred in open air for 4 hours. Afterwards, additional DCM and water were added, the organic phase was removed and washed with brine twice. The final organic phase was concentrated under vacuum to give a white powder. TLC revealed only a single spot, so no further purification was used. 73 mg of material was recovered (96% yield).

^{1}H NMR (400 MHz, CD_3OD): δ 7.98 (d, 1H), 7.67 (bs, 1H), 7.37 (m, 1H), 7.15 (dd, 1H), 6.37 (d, 1H), 4.01 (s, 3H)

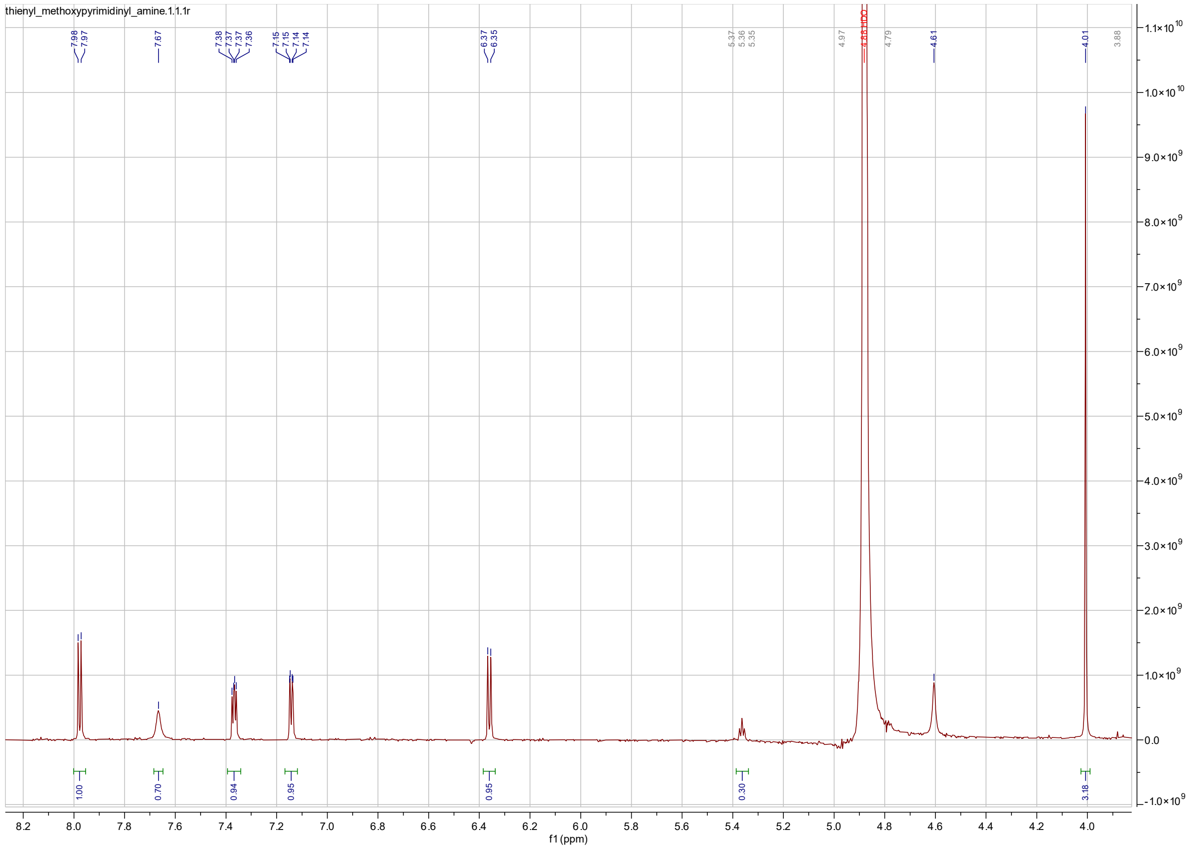

Figure S16. 1H-NMR spectrum of 2-methoxy-N-(thiophen-3-yl)pyrimidin-4-amine.

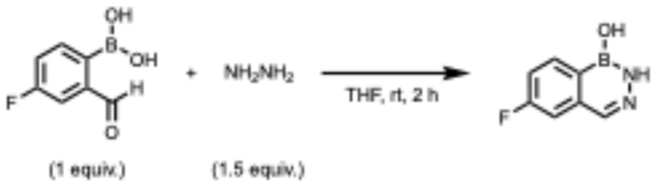

Synthesis of 6-fluorobenzo[*d*][1,2,3]diazaborinin-1(2*H*)-ol:

To a 20 mL vial equipped with a stir bar, (4-fluoro-2-formylphenyl)boronic acid (504 mg, 3.0 mmol, 1.0 equiv.) was dissolved in 10 mL of tetrahydrofuran. Then, hydrazine hydrate (0.3 mL, 1.5 equiv.) was added in one injection, and the reaction was stirred at room temperature for 2 hours. The solvent was then removed under reduced pressure, the crude material was washed with water (3 x 15 mL) and dried under vacuum to furnish the target product as a yellow solid (421 mg, 86% yield).

^{1}H NMR (400 MHz, acetone-d$_6$): δ 9.49 (bs, 1H), 8.31 – 8.20 (m, 1H), 8.00 (s, 1H), 7.48 (dd, 1H), 7.42 – 7.25 (m, 1H), 7.34 (s, 1H).

^{13}C NMR (101 MHz, acetone-d$_6$): δ 166.6, 164.2, 139.1, 134.7, 117.5, 112.4.

These values are in agreement with reported literature.[9]

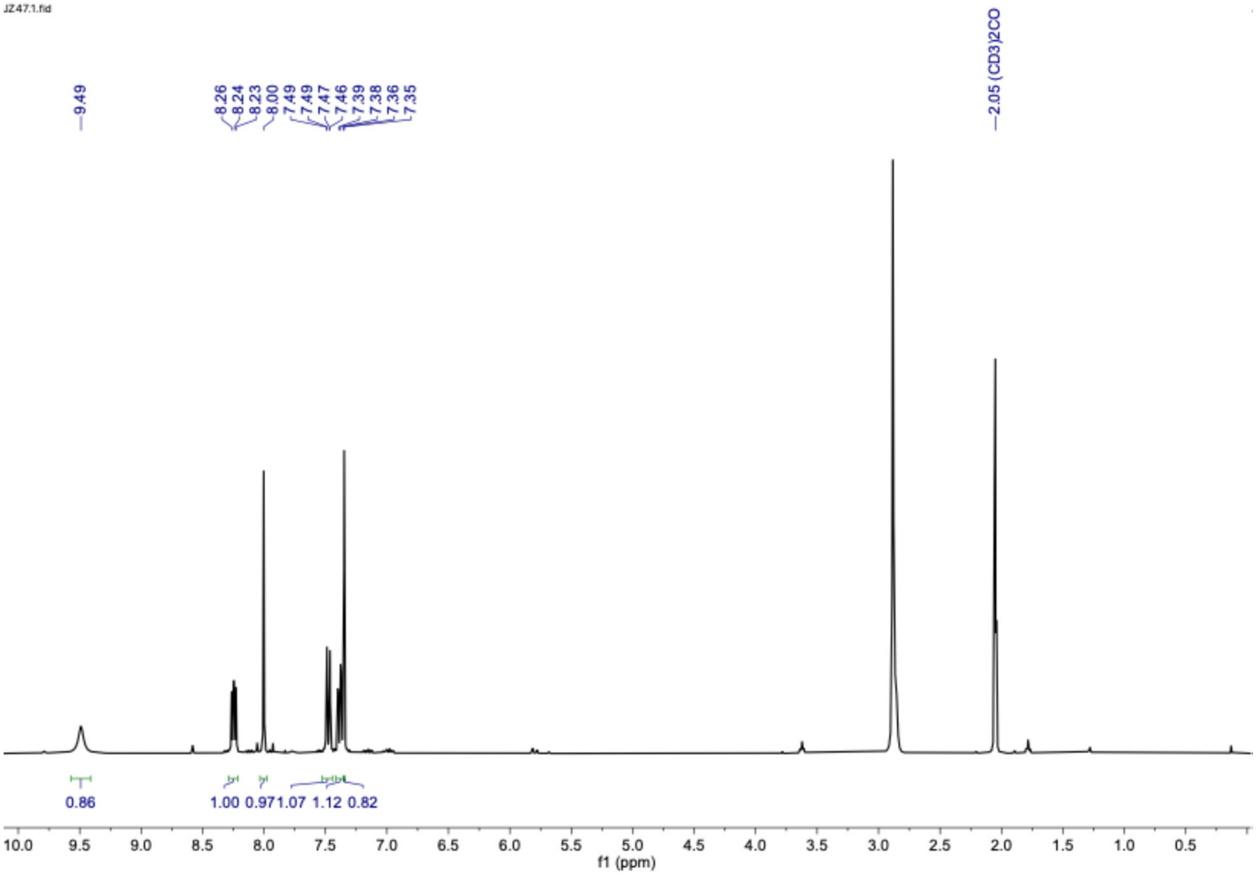

Figure S17. 1H-NMR of intermediate benzodiazaborininol in synthesis of 6-((2S,6R)-2,6-dimethylmorpholino)benzo[d][1,2,3]diazaborinin-1(2H)-ol.

Synthesis of 6-((2*S*,6*R*)-2,6-dimethylmorpholino)benzo[*d*][1,2,3]diazaborinin-1(2*H*)-ol:

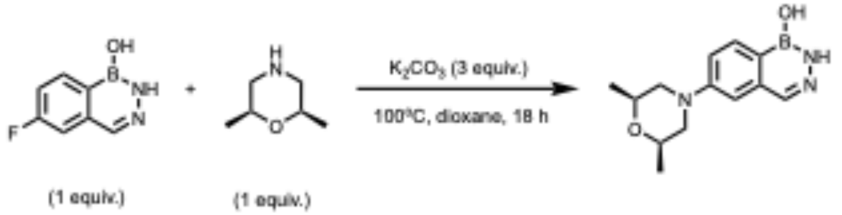

To a 20 mL vial equipped with a stir bar, 6-fluorobenzo[*d*][1,2,3]diazaborinin-1(2*H*)-ol (150 mg, 0.9 mmol, 1.0 equiv.), cis-2,6-dimethylmorpholine (104 mg, 0.9 mmol, 1.0 equiv.), and potassium carbonate (373 mg, 2.7 mmol, 3.0 equiv.) were dissolved in dioxane (5 mL). The reaction was heated to 100°C and stirred for 18 hours. After the reaction was finished, the liquid was filtered, concentrated, and dissolved in THF (1 mL). A preparatory TLC was run with 40% ethyl acetate: hexanes to afford the product as a yellow solid (3 mg, 1.3% yield).

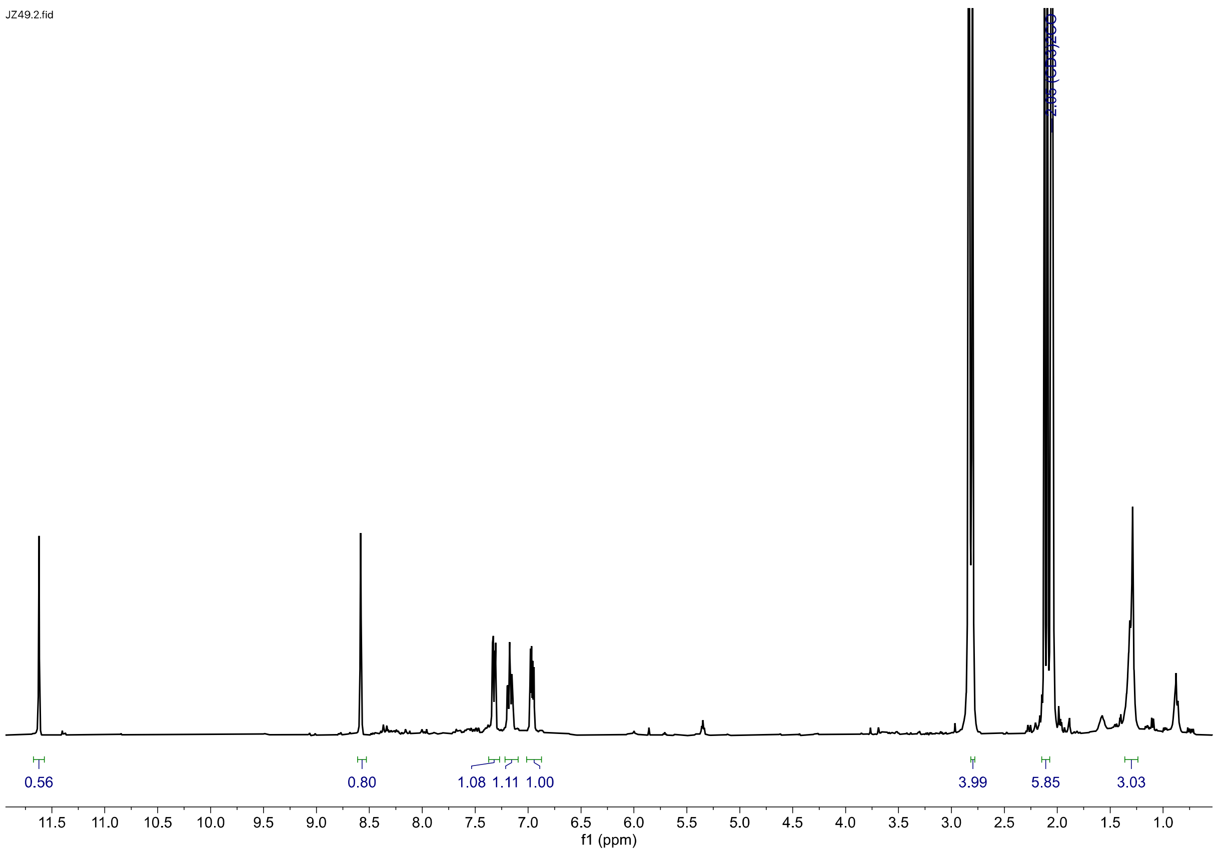

Figure S18. 1H-NMR spectrum of 6-((2S,6R)-2,6-dimethylmorpholino)benzo[d][1,2,3]diazaborinin-1(2H)-ol

Summary of automated reaction outcomes

Reaction outcome was automatically determined with HPLC-MS. Failed reactions have a retention time of (-1.00) minutes. Successful reactions also have the reported mass-to-charge ratio for the most abundant ion corresponding to the product of interest (SMILES). Missing wells indicate reactions that failed preparation, for example, the previous reaction step failed so the subsequent reaction was not prepared. The results are broken down by the iteration of MF-BO and plate they were run in. Each plate (within an iteration of MF-BO) may depend on reactions run in the preceding plates; therefore the final plates tend to have the most reactions. The HPLC-MS data for all reactions are available in the associated Zenodo repository, indexed by the MF-BO iteration, plate number, and associated well (reported in the tables below).

Table S2. First Iteration, 6 total plates

- First plate

Well	SMILES	Retention Time (min)	Detected m/z
A1	COc1ccc(Nn2cnnc2)cc1:	3.38	232.09
A10	COc1ccc(Nn2cnnc2)cc1OC:	7.31	262.1
A11	c1cc2cc(Nn3cnnc3)ccc2[nH]1:	3.32	241.09
A12	O=Cc1ccc(Nn2cnnc2)c([N+](=O)[O-])c1:	3.91	275.05
A2	Cc1ccc(Nn2cnnc2)cc1:	-1	0
A3	c1cc(Nn2cnnc2)cs1:	3.13	208.03
A4	O=C(O)c1ccc(Nn2cnnc2)cc1:	-1	0
A5	Nc1ccncc1Nn1cnnc1:	4.18	218.08
A6	c1ccc(C(Nn2cnnc2)c2ccccc2)cc1:	3.85	292.12
A7	c1ccc2nc(Nn3cnnc3)cnc2c1:	3.37	254.08
A9	CN(c1ccc(cn1)[N+]([O-])=O)c1ccc(Cl)cc1C(=O)c1ccccc1:	-1	0
B1	c1cnc(Nc2ccsc2)nc1:	-1	0
B2	O=[N+]([O-])c1ccc(Nn2cnnc2)nc1:	4.31	248.06
B3	c1ccc2oc(Nn3cnnc3)cc2c1:	3.65	242.07
B4	CC(Nc1ccc([N+](=O)[O-])cc1)c1ccccc1:	5.29	284.11
B5	CC(C)CNc1ccc([N+](=O)[O-])cc1:	7.52	236.11
B7	Brc1cc(Br)cc(Nn2cnnc2)c1:	4.51	357.9
B8	c1ccc2ncc(Nn3cnnc3)cc2c1:	3.64	253.09

- Second plate

Well	SMILES	Retention Time (min)	Detected m/z
A1	O=[N+]([O-])c1ccc(Nc2c(Cl)cccc2Cl)cc1:	7.82	324
A10	Cc1ccc(N(c2ccc(C#N)cc2)n2cnnc2)cc1:	-1	0
A11	CS(=O)(=O)Nc1ccc(N(c2cnc3ccccc3n2)n2cnnc2)cc1:	6.82	423.1
A12	COc1ccc(N(C(c2ccccc2)c2ccccc2)n2cnnc2)cc1:	5.25	398.16
A2	COc1ccc(N(c2c(C)noc2C)n2cnnc2)cc1:	4.88	327.12
A3	CC(C)(C)NS(C)(=O)=O:	7.73	193.07
A4	N#CNc1cnc2ccccc2n1:	6.93	212.06
A5	COC(=O)c1nc(N)ccc1N(c1ccc([N+](=O)[O-])cc1)C(C)c1ccccc1:	7.25	434.15
A6	Cn1nnc(Nc2ccsc2)n1:	4.03	-180.04
A7	O=[N+]([O-])c1ccc(N(c2ccsc2)n2cnnc2)cc1:	4.15	329.05
A8	O=C(O)CCC(=O)Nc1ccc(Nn2cnnc2)cc1:	-1	0
A9	O=[N+]([O-])c1ccc(Nn2cnnc2)cc1:	4.38	247.06
B1	O=[N+]([O-])c1ccc(NS(=O)(=O)C(F)(F)F)cc1:	7.27	311.99
B2	c1ccc(N(c2cc3ccccc3o2)n2cnnc2)cc1:	4.27	318.1
B3	CC(=O)OCC(=O)Nn1cnnc1:	2.48	226.06
B4	O=[N+]([O-])c1ccc(Nc2ccccc2-c2ccccc2)cc1:	4.82	332.11
B5	Nc1ccc(-c2ccccc2)cc1:	7.77	211.09
B6	O=Cc1ccc(Nn2cnnc2)cc1:	4.25	-187.07
B7	O=C(CS)N(c1ccsc1)n1cnnc1:	7.43	282.01
B8	N#Cc1ccccc1Nn1cnnc1:	-1	0
B9	CN(Nc1ccc([N+](=O)[O-])cc1)C(N)=S:	-1	0

- Third Plate

Well	SMILES	Retention Time (min)	Detected m/z
A1	Cc1ccc(S(=O)(=O)N(CC(C)C)c2ccc([N+](=O)[O-])cc2)cc1:	6.28	390.11
A2	COc1nccc(Nc2ccsc2)n1:	5.18	249.05
A4	COc1ccc(N(C(=O)COC(C)=O)n2cnnc2)cc1:	-1	0
A5	O=[N+]([O-])c1cnc(Nc2ccsc2)nc1:	4.9	264.02
A6	COc1ccc(N(C(=O)CCCBr)n2cnnc2)cc1OC:	-1	0
B1	C=CC(=O)Nn1cnnc1:	2	180.05
B2	O=[N+]([O-])c1ccc(N(c2cc3ccccc3o2)S(=O)(=O)C(F)(F)F)cc1:	-1	0
B3	O=S(=O)(Nc1ccc(-c2ccccc2)cc1)c1cccs1:	-1	0
B4	O=C(c1ccc(O)cc1)N(c1cnc2ccccc2c1)n1cnnc1:	-1	0
B5	O=C(Oc1ccccc1)N(c1cc(Br)cc(Br)c1)n1cnnc1:	6.37	477.92
B6	O=Cc1ccc(N(n2cnnc2)S(=O)(=O)c2ccccc2)c([N+](=O)[O-])c1:	4.78	415.05
C1	O=C(O)c1ccc(N(c2ccc(C(F)(F)F)cc2)n2cnnc2)cc1:	-1	0
C2	O=S(=O)(c1cccs1)N(c1cnc2ccccc2c1)n1cnnc1:	-1	0
C3	CS(=O)(=O)NS(C)(=O)=O:	6.56	214.98
C4	CS(=O)(=O)NS(=O)(=O)c1cccc2cccnc12:	6.96	328.01
C5	N#CCC(N)=OS(=O)(=O)C(F)(F)F:	6.81	0
D1	O=S(=O)(c1cccs1)N(c1ccsc1)c1ncccn1:	-1	0
D2	NC(=O)c1ccccc1N(c1cc(Br)cc(Br)c1)n1cnnc1:	-1	0
D3	CC(=O)Nc1ccc(Nc2ccc(-c3ccccc3)cc2)cc1:	4.98	344.14
D4	O=S(=O)(N(c1cnc2ccccc2n1)n1cnnc1)C(F)(F)F:	-1	0
D5	O=[N+]([O-])c1ccc(N(c2cc3ccccc3o2)n2cnnc2)nc1:	-1	0
E1	COc1ccc(NC#N)cc1:	6.99	190.06
E2	N#CCC(NS(=O)(c1ccccc1)=O)=O:	7.64	266.03
E4	CC(C)C(=O)Nn1cnnc1:	2.66	196.09
E5	c1ccc(-c2ccc(Nc3ccsc3)cc2)cc1:	-1	0
F1	COc1ccc(C(=O)N(c2cc(Br)cc(Br)c2)n2cnnc2)c(C)c1:	7.13	505.95
F2	O=Cc1c(Cl)nc(Cl)nc1Nn1cnnc1:	-1	0
F3	c1cncc(Nn2cnnc2)c1:	-1	0
G1	NS(=O)(=O)NS(=O)(=O)C(F)(F)F:	7.12	269.95
G2	Cc1cc(C)c(Nc2ccccc2C#N)c(C)c1:	7.27	278.13
G3	Cc1cc(C)c(NS(=O)(=O)c2ccccc2)c(C)c1:	5.52	317.1
G4	Cc1ccc(N(C(=O)N(C)C)n2cnnc2)cc1:	-1	0
H1	CC(C)(C)OC(=O)n1cccc1N(c1ccc([N+](=O)[O-])cc1)n1cnnc1:	-1	0
H2	CN(C(N)=S)N(c1ccc([N+](=O)[O-])cc1)S(=O)(=O)c1ccc(C(C)(C)C)cc1:	6.83	464.11
H3	Brc1cc(Br)cc(Nc2ncccn2)c1:	7.15	368.9
H4	CCOC(=O)N(c1ccc([N+](=O)[O-])cc1)c1ccccc1-c1ccccc1:	7.28	404.13

- Fourth plate

Well	SMILES	Retention Time (min)	Detected m/z
A1	N#Cc1ccccc1N(C(=O)c1sc2cc(F)ccc2c1Cl)n1cnnc1:	5.27	439.02
A2	O=[N+]([O-])c1ccc(N(S(=O)(=O)c2cccc3cccnc23)S(=O)(=O)C(F)(F)F)cc1:	4.43	503
A3	CS(=O)(=O)N(c1cccnc1)n1cnnc1:	-1	0
A4	CC(C)(C)c1ccc(S(=O)(=O)NS(C)(=O)=O)cc1:	5.17	333.06
B1	O=S(=O)(Nc1ncccn1)c1ccccc1:	2.43	277.04
B2	O=C(c1cc(F)c(F)cc1F)N(c1ccc([N+](=O)[O-])cc1)n1cnnc1:	4.2	405.06
B3	CC(=O)OCC(=O)N(c1cc2ccccc2o1)n1cnnc1:	4.53	342.09
B4	CS(=O)(=O)NS(=O)(=O)c1ccc(Br)cc1:	4.88	354.91
C1	N#Cc1ccc(Nc2cccnc2N)cc1:	3.57	252.09
C2	c1ccc(N(c2ccsc2)n2cnnc2)cc1:	4.85	284.06
C3	COc1ccc(N(C#N)S(=O)(=O)c2cccc(Br)c2)cc1:	-1	0
C4	CC(=O)Oc1ccccc1C(=O)N(c1cccnc1)n1cnnc1:	5.08	365.1
D1	CS(=O)(=O)NC#N:	-1	0
D2	O=C(O)CCC(=O)Nc1ccc(N(n2cnnc2)S(=O)(=O)c2cccc(Br)c2)cc1:	4.78	494.01
D3	COc1ccc(N(n2cnnc2)S(=O)(=O)c2ccccc2)cc1:	-1	0
D4	Cc1cc(C)c(N(C2CCc3ccccc32)S(=O)(=O)c2ccccc2)c(C)c1:	-1	0
E1	CC(C)(C)c1ccc(S(=O)(=O)N(C#N)c2cnc3ccccc3n2)cc1:	-1	0
E2	Cn1nnc(N(c2ccsc2)S(=O)(=O)c2ccccc2)n1:	3.8	0
E3	Cc1ccc(N(c2c(F)c(F)c(F)c(F)c2[N+](=O)[O-])n2cnnc2)cc1:	6.32	409.07
E4	O=Cc1ccc(N(n2cnnc2)S(=O)(=O)c2ccc(Br)cc2)cc1:	-1	0
F1	O=S(=O)(c1cccs1)N(c1ccc2[nH]ccc2c1)n1cnnc1:	5.47	387.04
F2	CC(C)(C)c1ccc(S(=O)(=O)N(c2ccc(-c3ccccc3)cc2)c2ccsc2)cc1:	-1	0
F3	N#CNS(=O)(=O)c1cccc(Br)c1:	-1	0

F4	NS(=O)(=O)N(S(=O)(=O)c1ccccc1)S(=O)(=O)C(F)(F)F:	4.32	409.94
G2	CC(=O)Nc1ccc(N(c2ccc(-c3ccccc3)cc2)S(=O)(=O)c2ccc(C)cc2)cc1:	4.57	498.15
G4	Cc1c(Br)cccc1C(=O)N(c1ccsc1)n1cnnc1:	5.32	403.98
H1	CC(C)(C)N(S(C)(=O)=O)S(=O)(=O)c1cccc(Br)c1:	4.47	410.97
H2	O=S(=O)(Nc1ccc(-c2ccccc2)cc1)c1ccccc1:	-1	0
H3	Cc1c(Br)cccc1C(=O)Nn1cnnc1:	4.42	322
H4	Cc1cc(C)c(NS(=O)(=O)c2cccs2)c(C)c1:	-1	0

- Fifth plate

Well	SMILES	Retention Time (min)	Detected m/z
A1	CS(=O)(=O)N(S(=O)(=O)c1ccccc1)S(=O)(=O)c1cccc2cccnc12:	-1	0
A2	OB(c1ccc(CC(C(O)=O)NS(=O)(c2sccc2)=O)cc1)O:	-1	0
A3	Cc1ccc(S(=O)(=O)N(N)c2ccc([N+](=O)[O-])cc2)cc1:	-1	0
B1	c1cncc(Nc2ncn[nH]2)c1:	-1	0
B2	Fc1ccc(Nc2ncn[nH]2)cc1:	4.67	220.07
B3	CS(=O)(=O)N(c1ccc(B(O)O)cc1)c1ccc([N+](=O)[O-])cc1:	5.3	378.06
C1	Cc1ccc(S(=O)(=O)N(c2cc(Br)cc(Br)c2)c2ncccn2)cc1:	-1	0
C2	O=Cc1c(Cl)nc(Cl)nc1N(C(=O)CCBr)n1cnnc1:	-1	0
D1	Clc1ccccc1Nn1cnnc1:	-1	0
D2	CC(C)N(c1ccc([N+](=O)[O-])cc1)C1CCCCC1:	-1	0
E1	COc1nccc(N(c2ccsc2)S(=O)(=O)c2ccc(C(C)(C)C)cc2)n1:	7.38	445.1
E2	O=[N+]([O-])c1ccc(N(c2ccccc2[N+](=O)[O-])S(=O)(=O)C(F)(F)F)cc1:	-1	0
F1	O=S(=O)(Nn1cnnc1)c1ccc(Br)cc1:	-1	0
F2	COc1nccc(N(c2ccsc2)S(=O)(=O)c2ccc(C)cc2)n1:	-1	0
G1	O=C(O)c1cccc(Nn2cnnc2)c1C(=O)O:	-1	0
G2	CS(=O)(=O)N(S(C)(=O)=O)S(=O)(=O)c1ccccc1:	-1	0
H1	CN(C)C(=O)Nn1cnnc1:	-1	0
H2	N#Cc1ccc(N(c2cnccc2N)n2cnnc2)cc1:	-1	0

- Sixth plate

Well	SMILES	Retention Time (min)	Detected m/z
A1	Cc1ccc(S(=O)(=O)N(c2ccc(-c3ccccc3)cc2)S(=O)(=O)c2ccccc2)cc1:	7.55	505.09
A2	O=[N+]([O-])c1cnc(N(c2ccsc2)S(=O)(=O)c2ccc(Br)cc2)nc1:	7.55	481.92
A3	COc1ccc(N(C#N)S(=O)(=O)c2cccc3cccnc23)cc1:	6.82	381.07
B1	C=CC(=O)N(c1ccc(NS(C)(=O)=O)cc1)n1cnnc1:	4.52	349.07
B2	CN(C)C(=O)N(c1ccc(NS(C)(=O)=O)cc1)n1cnnc1:	5.27	366.1
B3	Cc1cc(C)c(N(S(=O)(=O)c2ccc(Br)cc2)S(=O)(=O)c2cccs2)c(C)c1:	6.72	540.96
C1	Brc1cc(Br)cc(N(c2cccnc2)n2cnnc2)c1:	-1	0
C2	Cn1nnc(Nc2cc3ccccc3o2)n1:	-1	0
D1	COc1ccc(C(=O)N(c2c(C)cc(C)cc2C)S(=O)(=O)c2ccccc2)cc1C(F)(F)F:	6.48	478.12
D2	COc1cccc(C(=O)N(c2ccc(-c3ccccc3)cc2)S(=O)(=O)c2ccccc2)c1:	5.7	485.12
E1	O=C(O)c1[nH]cnc1C(=O)N(c1ccc(-c2ccccc2)cc1)S(=O)(=O)c1ccccc1:	-1	0
E2	Cc1cc(C)c(N(C(=O)c2nc(N)n[nH]2)S(=O)(=O)c2ccccc2)c(C)c1:	7.68	427.12
F1	CC(C)(C)c1ccc(S(=O)(=O)N(C(=O)Cc2ccccc2)S(C)(=O)=O)cc1:	-1	0
F2	O=[N+]([O-])c1cc(CNc2ccncc2Cl)ccc1N(n1cnnc1)S(=O)(=O)c1ccccc1:	-1	0
G1	CC(C)C(=O)N(c1ccc(C#N)cc1)n1cnnc1:	-1	0
G2	CS(=O)(=O)N(C#N)c1ccccc1:	-1	0
H1	N#Cc1ccc(Nc2ncn[nH]2)cc1:	4.87	227.07
H2	Cc1cc(C)c(N(C(=O)CBr)S(=O)(=O)c2ccccc2)c(C)c1:	7.42	437.02

Table S3. Second iteration, 4 total plates

- First plate

Well	SMILES	Retention Time (min)	Detected m/z
A1	COc1ccc(Nn2cnnc2)cc1	-1.00	232.09
A2	Nc1ccc(-c2ccc(C(F)(F)F)cn2)cc1	4.75	280.07
A3	c1ccc2ncc(Nn3cnnc3)cc2c1	3.25	253.09
A5	Nc1ccncc1Nn1cnnc1	-1.00	0.00
A6	CCc1ccc(Nn2cnnc2)cc1	-1.00	0.00
B2	O=Cc1ccc(-c2cc3ccccc3o2)s1	5.58	270.02
B3	c1ccc2nc(Nn3cnnc3)cnc2c1	-1.00	0.00
B4	O=Cc1ccccc1Nc1ccc(CO)cc1	4.68	269.09

B5	Nc1nc(Nn2cnnc2)c2[nH]cnc2n1	3.67	-216.08
C2	O=[N+]([O-])c1ccc(Nn2cnnc2)nc1	3.17	248.06
C4	O=Cc1ccc(-c2ccsc2)s1	3.63	235.99
C6	c1ccc(Nn2cnnc2)nc1	-1.00	0.00
D1	Clc1ccc(Nn2cnnc2)cc1	3.97	236.04
D3	O=[N+]([O-])c1ccc(NCc2ccccc2)cc1	5.36	270.09
D5	c1ccc(C(Nn2cnnc2)c2ccccc2)cc1	4.40	292.12
D6	CS(=O)(=O)Nc1ccc(Nn2cnnc2)cc1	-1.00	0.00
E1	Nc1ncccc1C=Cc1ccccc1	3.42	238.10
E3	c1nc(NC23CC4CC(CC(C4)C2)C3)c2[nH]cnc2n1	3.98	311.16
E4	O=[N+]([O-])c1ccc(N2CCS(=O)(=O)CC2)nc1	4.04	299.05
E5	COc1ccc2c3CCN=C(C)c3n(-c3cccs3)c2c1	-1.00	0.00
E6	O=[N+]([O-])c1ccc(NS(=O)(=O)C(F)(F)F)cc1	6.62	311.99
F1	c1ccc2oc(Nn3cnnc3)cc2c1	1.96	242.07
F2	Nc1ccc(-c2ccsc2)cn1	2.92	218.04
F4	c1ccc(-c2ccc(Nn3cnnc3)cc2)cc1	4.27	278.11
F5	O=Cc1cc(F)ccc1Nn1cnnc1	3.91	248.06
G2	O=S1(=O)CCN(c2cc3ccccc3o2)CC1	6.26	293.06
G3	O=C1CCc2cc(Nn3cnnc3)ccc21	1.60	256.09
G4	CC(Nc1ccc([N+](=O)[O-])cc1)c1ccccc1	5.47	284.11
G5	Brc1cc(Br)cc(Nn2cnnc2)c1	4.43	357.90
H1	COc1ccc2c3CCN=C(C)c3n(-c3cc4ccccc4o3)c2c1	7.12	372.14
H3	c1cc2cc(Nn3cnnc3)ccc2[nH]1	1.94	241.09
H4	O=[N+]([O-])c1ccc(Nc2cccc(OC(F)(F)F)c2)cc1	5.77	340.06
H5	Nc1nc2ccc(-c3ccsc3)cc2s1	2.92	274.01

- Second plate

Well	SMILES	Retention Time (min)	Detected m/z
A1	O=[N+]([O-])c1ccc(Nc2ccccc2-c2ccccc2)cc1	-1.00	0.00
A2	O=[N+]([O-])c1ccc(NC23CC4CC(CC(C4)C2)C3)cc1	6.10	314.15
A3	FC(F)(F)c1ccc(Nc2nc3ccc(Br)cc3s2)nc1	4.65	414.95
A4	O=[N+]([O-])c1ccc2nc(Nc3cc4ccccc4o3)sc2c1	7.22	0.00
B1	Nc1ccc(Nn2cnnc2)cc1	-1.00	0.00
B2	FC(F)(F)c1ccc(Nn2cnnc2)cc1	4.17	270.06
B3	COc1ccc(N(c2c(C)noc2C)n2cnnc2)cc1	3.80	327.12
B4	c1ccc2nc(-c3ccsc3)ccc2c1	4.48	253.05
C1	Cc1nnc(Nc2ccccc2Cl)s1	-1.00	0.00
C2	Nc1ccc(Nn2cnnc2)nc1	-1.00	0.00
D1	CC(C)(C)Nc1cc2ccccc2o1	4.48	231.12
D2	c1ccc2ncc(Nc3ccc(-c4ccsc4)cn3)cc2c1	4.82	345.08
D3	COc1ncc(Nn2cnnc2)cn1	3.72	234.08
D4	N#Cc1c([N+](=O)[O-])ccc(N2CCS(=O)(=O)CC2)c1Cl	4.36	357.01
E1	c1cc2c(cc1Nn1cnnc1)OCO2	3.05	246.06
E2	Nc1ccc(-c2ccccc2)cc1	4.00	211.09
E3	Cc1ccccc1N(C)c1ccc(Cl)cc1C(=O)c1ccccc1	6.55	377.11
E4	Nc1cc(Nn2cnnc2)ncn1	1.55	219.08
F1	COc1ccc(N(C(c2ccccc2)c2ccccc2)n2cnnc2)cc1	5.05	398.16
F2	CC(C)(C)Nc1ncccc1Br	-1.00	0.00
G1	Cc1ccccc1Nc1ccc2c(C(F)(F)F)cc(=O)oc2c1	-1.00	0.00
G2	Cn1nnc(Nc2ccsc2)n1	4.25	223.04
G3	Cc1noc(C)c1NS(C)(=O)=O	-1.00	0.00
H2	Nc1ccc(Nc2ncccn2)nc1	3.01	229.09

- Third Plate

Well	SMILES	Retention Time (min)	Detected m/z
A1	O=[N+]([O-])c1ccc(Nc2nc3ccccc3s2)cc1	5.55	313.04
A2	c1ccc2sc(Nn3cnnc3)nc2c1	4.47	259.04
A3	NCc1ccc(Nc2ncccn2)nc1	4.35	243.10
A4	N#CNc1nc2ccccc2s1	-1.00	0.00
B1	OC1CCN(c2ccccc2Cl)C1	-1.00	0.00
B2	COc1cc(N2CCS(=O)(=O)CC2)ccc1Cl	-1.00	0.00
B3	c1cnc(Nc2cnc3ccccc3n2)nc1	-1.00	0.00
B4	Cc1cc(C)c(Nc2ccc(CN)cn2)c(C)c1	4.33	283.16
C2	O=[N+]([O-])c1ccc(N(Cc2ccc(Cl)cc2)c2ccccc2-c2ccccc2)cc1	5.00	456.11

D1	Cc1ccccc1N(C)c1ccc(Nc2nncs2)cc1C(=O)c1ccccc1	6.52	442.14
D3	N#Cc1cnc(Cl)cc1N1CCS(=O)(=O)CC1	3.77	313.02
D4	O=[N+]([O-])c1ccc(Nc2nn[nH]n2)cc1	-1.00	0.00
E3	COc1ccc2nc(Nc3ccccc3C)sc2c1	3.45	312.08
F1	Cc1ccccc1NC1CCCCC1	3.47	190.15
F2	COc1cccc(Nc2ncccn2)c1	4.35	243.09
G1	CC(C)(C)Nc1ncccc1-c1ccc(C#N)cc1	-1.00	0.00
G3	N#Cc1ccc(Nc2ccc(-c3ccccc3)cc2)cc1	5.64	312.12
H3	Fc1cccc(F)c1-c1ccc2nc(Nc3ccc(C(F)(F)F)cn3)sc2c1	5.07	449.05

- Fourth Plate

Well	SMILES	Retention Time (min)	Detected m/z
A1	CN(C)c1ccc(CNc2cccc(OC(F)(F)F)c2)cc1	4.37	352.13
A2	O=S(=O)(O)c1ccc(NCc2ccc(-c3ccsc3)s2)cc1	5.12	393.01
A3	Fc1ccc(Nc2ncccn2)cc1	4.45	231.07
A4	[O-][N+](=O)c1ccc2nc(sc2c1)N(Cc1cncs1)c1cc2ccccc2o1	4.08	450.04
A5	Cc1c(Br)cccc1C(=O)Nn1cnnc1	3.93	322.00
A6	N#Cc1ccccc1Nn1cnnc1	-1.00	227.07
A7	O=S(=O)(Nn1cnnc1)C(F)(F)F	-1.00	0.00
B1	O=C(Nc1ncccn1)c1sc2cc(F)ccc2c1Cl	-1.00	0.00
B2	O=C(NCc1ccccc1)Nc1nnn(C)n1	3.95	274.11
B3	Clc1ccc(CNc2ccsc2)cn1	4.73	266.02
B4	CN(C)c1ccc(CNc2ccc(Cl)cn2)cc1	4.65	303.10
B5	O=S1(=O)CCN(c2nc3ccccc3s2)CC1	4.20	310.03
B6	NS(=O)(=O)NS(=O)(=O)C(F)(F)F	-1.00	0.00
C1	Cc1cc(C)c(NC(=O)c2ccno2)c(C)c1	4.58	272.11
C2	Fc1ccc(Nn2cnnc2)c(CNNc2ccccc2)c1	3.80	340.13
C4	CS(=O)(=O)NC(=O)c1sc2cc(F)ccc2c1Cl	-1.00	0.00
C5	O=C(Nn1cnnc1)c1ccc(O)cc1	2.86	246.06
C6	O=C(c1sc2ccccc2c1Cl)N(c1ccc([N+](=O)[O-])cc1)c1nnn(C)n1	3.55	456.03
C7	c1ccc(CNCc2cnc3ccccc3n2)cc1	3.37	291.13
D1	COc1ccc(N(c2nc3ccccc3s2)n2cnnc2)c(OC)c1	3.05	395.09
D2	COc1cccc(OC)c1NCc1cncs1	-1.00	0.00
D3	Cc1ccccc1N(c1ccc2c(C(F)(F)F)cc(=O)oc2c1)S(=O)(=O)c1cccs1	6.82	507.03
D4	FC(F)(F)c1ccc(-c2ccc(Nc3cnc4ccccc4c3)cc2)nc1	5.52	407.11
D5	O=C(CO)N(C(c1ccccc1)c1ccccc1)n1cnnc1	4.15	0.00
D6	COc1cc(Br)c(C(=O)N(c2ccc(CO)cc2)c2ccccc2C=O)cc1O	5.92	497.04
D7	c1ccc2sc(N(c3ccc4c(c3)OCO4)n3cnnc3)nc2c1	5.23	379.06
E1	Cc1cccc(CNc2ncn[nH]2)c1	-1.00	0.00
E2	CCn1c2ccccc2c2cc(CNc3ccncc3Nn3cnnc3)ccc21	5.48	425.19
E4	COc1ccc2nc(N(c3ccccc3C)S(=O)(=O)c3cccs3)sc2c1	5.17	458.03
E5	O=S(=O)(Nn1cnnc1)c1cccs1	3.47	271.99
E6	C1=CC=C2C(=C1)C(=CN2)CCNC(C)(C)C	4.03	258.16
E7	COc1ccc(Cl)c(C(=O)Nn2cnnc2)c1	3.40	294.04
F1	COc1ccc(NC(=O)Nc2ccncc2Nn2cnnc2)cc1	3.83	367.13
F2	CN(C)c1ccc(CNCc2ccccc2)cc1[N+](=O)[O-]	7.01	327.15
F3	CCCCCN(c1cnc2ccccc2c1)n1cnnc1	4.68	323.16
F4	FC(F)(F)Oc1ccc(Nc2ncccn2)cc1	5.17	297.06
F5	CS(=O)(=O)NCc1cccc(B(O)O)c1	3.93	271.06
F6	Cc1cccc(CNCc2ccc(Cl)nc2)c1	3.82	288.09
G1	O=S(=O)(c1cccs1)N(c1cnc2ccccc2c1)n1cnnc1	5.05	399.04
G2	c1cc2cn[nH]c2cc1NCC1CCCCC1	4.87	271.16
G3	Clc1ccc(Nc2ccc(-c3ccccc3)cc2)cc1	4.73	321.08
G4	O=S(=O)(Nc1ncccn1)c1cccc(Br)c1	-1.00	0.00
G5	Cc1ccc(S(=O)(=O)Nc2ccc(Nn3cnnc3)nc2)cc1	-1.00	0.00
G6	COc1ccc(N2CCS(=O)(=O)CC2)cc1	3.97	283.08
G7	Clc1ncccc1NCc1ccc(-c2cc3ccccc3o2)s1	5.53	382.04
H1	N#CNS(=O)(=O)c1ccccc1	-1.00	0.00
H2	O=[N+]([O-])c1ccc(N(c2ccccc2CO)S(=O)(=O)C(F)(F)F)cc1	-1.00	0.00
H3	O=Cc1cc(F)ccc1N(c1ccccn1)n1cnnc1	-1.00	0.00
H4	O=C(Nc1ccncc1Nn1cnnc1)Oc1ccccc1	5.38	338.10
H5	N#CN(c1nc2ccccc2s1)S(=O)(=O)c1ccccc1	3.70	357.01
H6	CS(=O)(=O)NCc1ccc(-n2ccnc2)cc1	1.78	293.07
H7	CS(=O)(=O)NS(=O)(=O)c1cccc(Br)c1	-1.00	0.00

Summary of assay results from HDACI discovery campaign

Table S4. Summary of single point assay results for first iteration of MF-BO applied to HDACI discovery.

Assay Well	SMILES	*m/z* detected	Ret. time	Source plate	Source well	Assay Mean	Assay st.dev	Percent inhibited	Scaled error
H3	O=C(/C=C/C(C)=C/[C@@H](C)C(c1ccc(N(C)C)cc1)=O)NO	NA	NA	NA	TRICH. A	2609.060	797.520	0.996	0.017
E2	CC(C)(C)N(S(C)(=O)=O)S(=O)(=O)c1cccc(Br)c1	410.9	4.47	22_15	H1	4794.000	707.724	0.946	0.015
A10	Cc1ccc(S(=O)(=O)N(CC(C)C)c2ccc([N+](=O)[O-])cc2)cc1	390.1	6.28	22_14	A1	8639.222	421.067	0.858	0.009
F9	CS(=O)(=O)N(c1ccc(B(O)O)cc1)c1ccc([N+](=O)[O-])cc1	378	5.30	22_17	B3	10841.778	634.366	0.807	0.014
G2	O=[N+]([O-])c1ccc(N(c2ccccc2[N+](=O)[O-])S(=O)(=O)C(F)(F)F)cc1	0	-1.00	22_17	E2	13459.889	1297.191	0.747	0.028
D2	O=C(O)CCC(=O)Nc1ccc(N(n2cnnc2)S(=O)(=O)c2cccc(Br)c2)cc1	494	4.78	22_15	D2	13618.000	2062.723	0.744	0.045
C6	O=[N+]([O-])c1ccc(N(S(=O)(=O)c2cccc3cccnc23)S(=O)(=O)C(F)(F)F)cc1	502.9	4.43	22_15	A2	14024.778	1065.255	0.734	0.023
F2	O=[N+]([O-])c1cc(CNc2ccncc2Cl)ccc1N(n1cnnc1)S(=O)(=O)c1ccccc1	0	-1.00	22_16	F2	14652.444	1534.786	0.720	0.033
G6	N#Cc1ccc(N(c2cnccc2N)n2cnnc2)cc1	0	-1.00	22_17	H2	20663.333	1579.097	0.582	0.034
E6	CN(C)C(=O)N(c1ccc(NS(C)(=O)=O)cc1)n1cnnc1	366.1	5.27	22_16	B2	23873.889	1967.377	0.509	0.043
F12	CC(C)N(c1ccc([N+](=O)[O-])cc1)C1CCCCC1	0	-1.00	22_17	D2	25633.556	2395.363	0.469	0.052
C4	CCOC(=O)N(c1ccc([N+](=O)[O-])cc1)c1ccccc1-c1ccccc1	404.1	7.28	22_14	H4	26291.778	2411.908	0.453	0.052
B4	O=Cc1ccc(N(n2cnnc2)S(=O)(=O)c2ccccc2)c([N+](=O)[O-])c1	415	4.78	22_14	B6	26379.556	2517.078	0.451	0.055
F8	Cc1ccc(S(=O)(=O)N(N)c2ccc([N+](=O)[O-])cc2)cc1	0	-1.00	22_17	A3	28620.111	2465.262	0.400	0.053
C12	COc1ccc(N(C#N)S(=O)(=O)c2cccc(Br)c2)cc1	0	-1.00	22_15	C3	28914.333	4814.769	0.393	0.104
G4	COc1nccc(N(c2ccsc2)S(=O)(=O)c2ccc(C)cc2)n1	0	-1.00	22_17	F2	29468.778	2939.676	0.381	0.064
A9	O=C(CS)N(c1ccsc1)n1cnnc1	282	7.43	22_13	B7	30705.556	2747.038	0.352	0.060
C3	CN(C(N)=S)N(c1ccc([N+](=O)[O-])cc1)S(=O)(=O)c1ccc(C(C)(C)C)cc1	464.1	6.83	22_14	H2	31754.889	3094.535	0.328	0.067
C9	O=C(c1cc(F)c(F)cc1F)N(c1ccc([N+](=O)[O-])cc1)n1cnnc1	405	4.20	22_15	B2	32071.778	2454.919	0.321	0.053
G5	CS(=O)(=O)N(S(C)(=O)=O)S(=O)(=O)c1ccccc1	0	-1.00	22_17	G2	32096.778	4160.911	0.320	0.090
F5	Cc1cc(C)c(N(C(=O)CBr)S(=O)(=O)c2ccccc2)c(C)c1	437	7.42	22_16	H2	32534.778	2952.621	0.310	0.064
E9	COc1ccc(C(=O)N(c2c(C)cc(C)cc2C)S(=O)(=O)c2ccccc2)cc1C(F)(F)F	478.1	6.48	22_16	D1	32842.000	2819.870	0.303	0.061
D1	CC(=O)Oc1ccccc1C(=O)N(c1cccnc1)n1cnnc1	365.1	5.08	22_15	C4	33415.889	3713.977	0.290	0.081
E1	Cc1c(Br)cccc1C(=O)N(c1ccsc1)n1cnnc1	403.9	5.32	22_15	G4	33725.333	5522.226	0.283	0.120
A1	CN(c1ccc(cn1)[N+]([O-])=O)c1ccc(Cl)cc1C(=O)c1ccccc1	0	-1.00	22_12	A9	33928.111	3519.168	0.279	0.076
B10	O=[N+]([O-])c1ccc(N(c2cc3ccccc3o2)n2cnnc2)nc1	0	-1.00	22_14	D5	34218.889	2548.258	0.272	0.055
F1	CC(C)(C)c1ccc(S(=O)(=O)N(C(=O)Cc2ccccc2)S(C)(=O)=O)cc1	0	-1.00	22_16	F1	35113.889	3616.569	0.251	0.078
E12	Cc1cc(C)c(N(C(=O)c2nc(N)n[nH]2)S(=O)(=O)c2ccccc2)c(C)c1	427.1	7.68	22_16	E2	35518.000	2653.932	0.242	0.058
F3	CC(C)C(=O)N(c1ccc(C#N)cc1)n1cnnc1	0	-1.00	22_16	G1	36026.444	3555.005	0.230	0.077
G1	COc1nccc(N(c2ccsc2)S(=O)(=O)c2ccc(C(C)(C)C)cc2)n1	445.1	7.38	22_17	E1	36615.889	4286.282	0.217	0.093
B1	O=[N+]([O-])c1ccc(N(c2cc3ccccc3o2)S(=O)(=O)C(F)(F)F)cc1	0	-1.00	22_14	B2	36661.556	3507.465	0.216	0.076
F7	OB(c1ccc(CC(C(O)=O)NS(=O)(c2sccc2)=O)cc1)O	0	-1.00	22_17	A2	37293.556	2941.285	0.201	0.064
F4	CS(=O)(=O)N(C#N)c1ccccc1	0	-1.00	22_16	G2	37441.222	3919.758	0.198	0.085
F6	CS(=O)(=O)N(S(=O)(=O)c1ccccc1)S(=O)(=O)c1cccc2cccnc12	0	-1.00	22_17	A1	37602.222	3369.619	0.194	0.073
B12	COc1ccc(C(=O)N(c2cc(Br)cc(Br)c2)n2cnnc2)c(C)c1	505.9	7.13	22_14	F1	37792.111	4832.148	0.190	0.105
A12	COc1ccc(N(C(=O)CCCBr)n2cnnc2)cc1OC	0	-1.00	22_14	A6	37796.556	2869.019	0.190	0.062
A4	COc1ccc(N(C(c2ccccc2)c2ccccc2)n2cnnc2)cc1	398.1	5.25	22_13	A12	38449.111	3582.118	0.175	0.078
B2	O=C(c1ccc(O)cc1)N(c1cnc2ccccc2c1)n1cnnc1	0	-1.00	22_14	B4	38883.111	4047.531	0.165	0.088

D6	Cn1nnc(N(c2ccsc2)S(=O)(=O)c2ccccc2)n1	0	3.80	22_15	E2	39041.333	3731.360	0.161	0.081
E10	COc1cccc(C(=O)N(c2ccc(-c3ccccc3)cc2)S(=O)(=O)c2ccccc2)c1	485.1	5.70	22_16	D2	39324.667	2575.087	0.155	0.056
D4	Cc1cc(C)c(N(C2CCc3ccccc32)S(=O)(=O)c2ccccc2)c(C)c1	0	-1.00	22_15	D4	39357.889	4316.680	0.154	0.094
A5	COc1ccc(N(c2c(C)noc2C)n2cnnc2)cc1	327.1	4.88	22_13	A2	39471.889	3912.645	0.152	0.085
B7	O=S(=O)(c1cccs1)N(c1ccsc1)c1ncccn1	0	-1.00	22_14	D1	39697.556	3380.568	0.146	0.073
A3	CS(=O)(=O)Nc1ccc(N(c2cnc3ccccc3n2)n2cnnc2)cc1	423.1	6.82	22_13	A11	39740.667	3531.708	0.145	0.077
D9	O=S(=O)(c1cccs1)N(c1ccc2[nH]ccc2c1)n1cnnc1	387	5.47	22_15	F1	40049.000	3453.337	0.138	0.075
D7	Cc1ccc(N(c2c(F)c(F)c(F)c(F)c2[N+](=O)[O-])n2cnnc2)cc1	409	6.32	22_15	E3	40410.889	4133.229	0.130	0.090
B9	O=S(=O)(N(c1cnc2ccccc2n1)n1cnnc1)C(F)(F)F	0	-1.00	22_14	D4	40432.111	2878.455	0.130	0.062
A2	Cc1ccc(N(c2ccc(C#N)cc2)n2cnnc2)cc1	0	-1.00	22_13	A10	40834.222	3884.210	0.120	0.084
B11	N#CCC(NS(=O)(c1ccccc1)=O)=O	266	7.64	22_14	E2	40898.111	3159.252	0.119	0.069
D5	CC(C)(C)c1ccc(S(=O)(=O)N(C#N)c2cnc3ccccc3n2)cc1	0	-1.00	22_15	E1	41096.111	4143.238	0.114	0.090
C5	N#Cc1ccccc1N(C(=O)c1sc2cc(F)ccc2c1Cl)n1cnnc1	439	5.27	22_15	A1	41300.889	4079.426	0.110	0.089
B3	O=C(Oc1ccccc1)N(c1cc(Br)cc(Br)c1)n1cnnc1	477.9	6.37	22_14	B5	41415.556	3926.649	0.107	0.085
C11	c1ccc(N(c2ccsc2)n2cnnc2)cc1	284	4.85	22_15	C2	41429.778	5843.576	0.107	0.127
D12	CC(=O)Nc1ccc(N(c2ccc(-c3ccccc3)cc2)S(=O)(=O)c2ccc(C)cc2)cc1	498.1	4.57	22_15	G2	41769.444	3135.258	0.099	0.068
C1	Cc1ccc(N(C(=O)N(C)C)n2cnnc2)cc1	0	-1.00	22_14	G4	41844.444	4662.135	0.097	0.101
D8	O=Cc1ccc(N(n2cnnc2)S(=O)(=O)c2ccc(Br)cc2)cc1	0	-1.00	22_15	E4	41879.222	3691.242	0.096	0.080
G3	O=S(=O)(Nn1cnnc1)c1ccc(Br)cc1	0	-1.00	22_17	F1	42024.222	4243.515	0.093	0.092
E7	Cc1cc(C)c(N(S(=O)(=O)c2ccc(Br)cc2)S(=O)(=O)c2cccs2)c(C)c1	540.9	6.72	22_16	B3	42489.556	3634.696	0.082	0.079
C2	CC(C)(C)OC(=O)n1cccc1N(c1ccc([N+](=O)[O-])cc1)n1cnnc1	0	-1.00	22_14	H1	42908.778	4391.906	0.073	0.095
B5	O=C(O)c1ccc(N(c2ccc(C(F)(F)F)cc2)n2cnnc2)cc1	0	-1.00	22_14	C1	42934.333	3397.667	0.072	0.074
D10	CC(C)(C)c1ccc(S(=O)(=O)N(c2ccc(-c3ccccc3)cc2)c2ccsc2)cc1	0	-1.00	22_15	F2	42966.333	3491.324	0.071	0.076
B6	O=S(=O)(c1cccs1)N(c1cnc2ccccc2c1)n1cnnc1	0	-1.00	22_14	C2	43021.111	3805.868	0.070	0.083
A8	c1ccc(N(c2cc3ccccc3o2)n2cnnc2)cc1	318.1	4.27	22_13	B2	43092.667	3406.855	0.069	0.074
C8	O=S(=O)(Nc1ncccn1)c1ccccc1	277	2.43	22_15	B1	43316.667	3476.762	0.063	0.075
F10	Cc1ccc(S(=O)(=O)N(c2cc(Br)cc(Br)c2)c2ncccn2)cc1	0	-1.00	22_17	C1	43480.333	2315.658	0.060	0.050
E4	COc1ccc(N(C#N)S(=O)(=O)c2cccc3cccnc23)cc1	381	6.82	22_16	A3	43691.000	4231.663	0.055	0.092
F11	O=Cc1c(Cl)nc(Cl)nc1N(C(=O)CCBr)n1cnnc1	0	-1.00	22_17	C2	43698.111	3195.217	0.055	0.069
C7	CS(=O)(=O)N(c1cccnc1)n1cnnc1	0	-1.00	22_15	A3	43948.333	3730.344	0.049	0.081
E5	C=CC(=O)N(c1ccc(NS(C)(=O)=O)cc1)n1cnnc1	349	4.52	22_16	B1	44128.333	4465.930	0.045	0.097
A11	COc1ccc(N(C(=O)COC(C)=O)n2cnnc2)cc1	0	-1.00	22_14	A4	44791.444	3198.924	0.030	0.069
D11	NS(=O)(=O)N(S(=O)(=O)c1ccccc1)S(=O)(=O)C(F)(F)F	409.9	4.32	22_15	F4	44818.000	3410.876	0.029	0.074
E11	O=C(O)c1[nH]cnc1C(=O)N(c1ccc(-c2ccccc2)cc1)S(=O)(=O)c1ccccc1	0	-1.00	22_16	E1	45464.000	3393.020	0.014	0.074
E3	Cc1ccc(S(=O)(=O)N(c2ccc(-c3ccccc3)cc2)S(=O)(=O)c2ccccc2)cc1	505	7.55	22_16	A1	46444.000	4578.709	-0.008	0.099
E8	Brc1cc(Br)cc(N(c2cccnc2)n2cnnc2)c1	0	-1.00	22_16	C1	47022.222	4103.348	-0.021	0.089
D3	COc1ccc(N(n2cnnc2)S(=O)(=O)c2ccccc2)cc1	0	-1.00	22_15	D3	47177.333	8779.405	-0.025	0.190
C10	CC(=O)OCC(=O)N(c1cc2ccccc2o1)n1cnnc1	342	4.53	22_15	B3	47408.111	5571.851	-0.030	0.121
B8	NC(=O)c1ccccc1N(c1cc(Br)cc(Br)c1)n1cnnc1	0	-1.00	22_14	D2	47494.222	4073.805	-0.032	0.088
A6	COC(=O)c1nc(N)ccc1N(c1ccc([N+](=O)[O-])cc1)C(C)c1ccccc1	434.1	7.25	22_13	A5	53466.889	3581.005	-0.169	0.078
A7	O=[N+]([O-])c1ccc(N(c2ccsc2)n2cnnc2)cc1	329	4.15	22_13	A7	53880.333	4847.247	-0.179	0.105

Table S5. Summary of single point assay results for second iteration of MF-BO applied to HDACI discovery.

Assay Well	SMILES	*m/z* detected	Ret. time	Source plate	Source well	Assay Mean	Assay st.dev	Percent inhibited	Scaled error
E10	CN(C)c1ccc(CNc2cccc(OC(F)(F)F)c2)cc1	352.1	4.37	HDAC_2_4	A1	1374.11	55.08	0.921	0.004
F7	CN(C)c1ccc(CNc2ccc(Cl)cn2)cc1	303.1	4.65	HDAC_2_4	B4	2194.33	111.30	0.860	0.008
A11	O=[N+]([O-])c1ccc(N2CCS(=O)(=O)CC2)nc1	299.0	4.04	HDAC_2_1	E4	2721.00	140.07	0.821	0.010
F1	O=[N+]([O-])c1ccc2nc(N(CCNCC3CCCCC3)c3cc4ccccc4o3)sc2c1	450.0	4.08	HDAC_2_4	A4	4556.22	553.71	0.684	0.040
B11	Nc1nc2ccc(-c3ccsc3)cc2s1	274.0	2.92	HDAC_2_1	H5	6496.89	519.36	0.539	0.038
H4	CN(C)c1ccc(CNCc2ccc(C#N)cc2)cc1[N+](=O)[O-]	327.1	7.01	HDAC_2_4	F2	6681.67	646.60	0.525	0.047
A2-2	Cc1ccc(S(=O)(=O)Nc2ccc(Nn3cnnc3)nc2)cc1	0.0	-1.00	HDAC_2_4	G5	7125.07	1984.75	0.492	0.145
F9	Cc1cc(C)c(NC(=O)c2ccno2)c(C)c1	272.1	4.58	HDAC_2_4	C1	7133.89	482.75	0.491	0.035
C7	O=[N+]([O-])c1ccc2nc(Nc3cc4ccccc4o3)sc2c1	#N/A	#N/A	HDAC_2_2	C4	7929.89	492.98	0.432	0.036
B10	Cc1ccccc1Nc1cccc(OC(F)(F)F)c1	340.1	5.77	HDAC_2_1	H4	8703.78	660.29	0.374	0.048
G7	COc1cc(Br)c(C(=O)N(c2ccc(CO)cc2)c2ccccc2C=O)cc1O	497.0	5.92	HDAC_2_4	D6	8759.67	587.76	0.370	0.043
G2	COc1ccc(N(c2nc3ccccc3s2)n2cnnc2)c(OC)c1	395.1	3.05	HDAC_2_4	D1	8784.44	922.99	0.368	0.067
D10	O=[N+]([O-])c1ccc(N(Cc2ccc(Cl)cc2)c2ccccc2-c2ccccc2)cc1	456.1	5.00	HDAC_2_3	C2	8891.22	563.92	0.360	0.041
A3-2	COc1ccc(N2CCS(=O)(=O)CC2)cc1	283.1	3.97	HDAC_2_4	G6	8907.66	3087.84	0.359	0.225
B6	CC(Nc1ccc([N+](=O)[O-])cc1)c1ccccc1	284.1	5.47	HDAC_2_1	G4	9415.33	669.88	0.321	0.049
E7	N#Cc1ccc(Nc2ccc(-c3ccccc3)cc2)cc1	312.1	5.64	HDAC_2_3	G3	9511.89	813.33	0.313	0.059
G1	N#Cc1ccc(CNCc2cnc3ccccc3n2)cc1	291.1	3.37	HDAC_2_4	C7	9823.78	1086.90	0.290	0.079
C9	c1ccc2ncc(Nc3ccc(-c4ccsc4)cn3)cc2c1	345.1	4.82	HDAC_2_2	D2	9989.78	1440.09	0.278	0.105
F6	Clc1ccc(CNc2ccsc2)cn1	266.0	4.73	HDAC_2_4	B3	10051.56	762.56	0.273	0.056
F10	Fc1ccc(Nn2cnnc2)c(CNNc2ccccc2)c1	340.1	3.80	HDAC_2_4	C2	10249.78	658.13	0.258	0.048
C1	Nc1ccc(Nn2cnnc2)cc1	0.0	-1.00	HDAC_2_2	B1	10506.44	1132.21	0.239	0.083
F11	CS(=O)(=O)NC(=O)c1sc2cc(F)ccc2c1Cl	0.0	-1.00	HDAC_2_4	C4	10578.89	1077.77	0.234	0.079
C3	COc1ccc(N(c2c(C)noc2C)n2cnnc2)cc1	327.1	3.80	HDAC_2_2	B3	10806.67	1008.06	0.217	0.074
E1	O=[N+]([O-])c1ccc(Nc2nc3ccccc3s2)cc1	#N/A	#N/A	HDAC_2_3	D2	10840.22	1103.92	0.214	0.081
D11	N#CNc1nc2ccccc2s1	#N/A	#N/A	HDAC_2_3	C3	10858.22	655.78	0.213	0.048
G11	COc1ccc2nc(N(c3ccccc3C)S(=O)(=O)c3cccs3)sc2c1	458.0	5.17	HDAC_2_4	E4	10884.67	659.13	0.211	0.048
A12	c1cnc(Nc2ccsc2)nc1	0.0	-1.00	HDAC_2_1	E5	11004.11	674.36	0.202	0.049
C12	c1cc2c(cc1Nn1cnnc1)OCO2	246.1	3.05	HDAC_2_2	E1	11388.44	773.91	0.173	0.056
E9	Fc1cccc(F)c1-c1ccc2nc(Nc3ccc(C(F)(F)F)cn3)sc2c1	449.1	5.07	HDAC_2_3	H3	11405.78	742.61	0.172	0.054
F12	O=C(c1sc2ccccc2c1Cl)N(c1ccc([N+](=O)[O-])cc1)c1nn[nH]n1	456.0	3.55	HDAC_2_4	C6	11410.11	618.27	0.172	0.045
E5	COc1ccc2nc(Nc3ccccc3C)sc2c1	#N/A	#N/A	HDAC_2_3	F3	11463.00	1021.62	0.168	0.075
B12	O=[N+]([O-])c1ccc(NC23CC4CC(CC(C4)C2)C3)cc1	314.2	6.10	HDAC_2_2	A2	11509.33	861.54	0.164	0.063
A6	O=[N+]([O-])c1ccc(Nn2cnnc2)nc1	248.1	3.17	HDAC_2_1	C2	11517.78	831.40	0.164	0.061
D5	FC(F)(F)c1ccc(Nc2nc3ccc(Br)cc3s2)nc1	#N/A	#N/A	HDAC_2_2	G4	11570.56	961.76	0.160	0.070
E12	Fc1ccc(Nc2ncccn2)cc1	231.1	4.45	HDAC_2_4	A3	11577.22	685.43	0.159	0.050
E3	Cc1ccccc1NC1CCCCC1	190.2	3.47	HDAC_2_3	F1	11602.56	1043.28	0.157	0.076
G3	COc1cccc(OC)c1NCc1cncs1	0.0	-1.00	HDAC_2_4	D2	11604.67	1944.17	0.157	0.142
H1	CC(C)(C)NCCc1ccc(O)cc1	258.2	4.03	HDAC_2_4	E6	11655.22	1594.99	0.153	0.116
C11	N#Cc1c([N+](=O)[O-])ccc(N2CCS(=O)(=O)CC2)c1Cl	357.0	4.36	HDAC_2_2	D4	11729.00	897.23	0.148	0.065
E4	COc1cccc(Nc2ncccn2)c1	243.1	4.35	HDAC_2_3	F2	11768.89	1028.08	0.145	0.075
B2	c1ccc(C(Nn2cnnc2)c2ccccc2)cc1	#N/A	#N/A	HDAC_2_1	F3	11791.00	1088.54	0.143	0.079

F8	O=S1(=O)CCN(c2nc3ccccc3s2)CC1	310.0	4.20	HDAC_2_4	B5	11931.67	752.70	0.133	0.055
C4	c1ccc2nc(-c3ccsc3)ccc2c1	253.0	4.48	HDAC_2_2	B4	12018.33	1355.59	0.126	0.099
H8	O=S(=O)(c1cccs1)N(c1cnc2ccccc2c1)n1cnnc1	399.0	5.05	HDAC_2_4	G1	12076.78	777.08	0.122	0.057
D12	Cc1ccccc1N(C)c1ccc(Nc2nncs2)cc1C(=O)c1ccccc1	442.1	6.52	HDAC_2_3	D1	12105.56	722.43	0.120	0.053
H5	CCCCCN(c1cnc2ccccc2c1)n1cnnc1	323.2	4.68	HDAC_2_4	F3	12151.00	991.84	0.116	0.072
H2	COc1ccc(Cl)c(C(=O)Nn2cnnc2)c1	294.0	3.40	HDAC_2_4	E7	12182.00	1211.60	0.114	0.088
A1	COc1ccc(Nn2cnnc2)cc1	232.1	-1.00	HDAC_2_1	A1	12190.78	1228.82	0.113	0.090
B5	O=C1CCc2cc(Nn3cnnc3)ccc21	256.1	1.60	HDAC_2_1	G3	12331.11	970.57	0.103	0.071
G8	c1ccc2sc(N(c3ccc4c(c3)OCO4)n3cnnc3)nc2c1	379.1	5.23	HDAC_2_4	D7	12456.89	900.76	0.093	0.066
A4	c1ccc2nc(Nn3cnnc3)cnc2c1	0.0	-1.00	HDAC_2_1	B3	12508.78	1076.07	0.090	0.078
G12	O=S(=O)(Nn1cnnc1)c1cccs1	272.0	3.47	HDAC_2_4	E5	12682.11	732.48	0.077	0.053
D2	COc1ccc(N(C(c2ccccc2)c2ccccc2)n2cnnc2)cc1	398.2	5.05	HDAC_2_2	F1	12743.67	1273.55	0.072	0.093
D1	Nc1cc(Nn2cnnc2)ncn1	219.1	1.55	HDAC_2_2	E4	12760.78	1682.58	0.071	0.123
A5	Nc1nc(Nn2cnnc2)c2[nH]cnc2n1	-216.1	3.67	HDAC_2_1	B5	12809.00	1077.15	0.067	0.079
H7	Cc1cccc(CNCc2ccc(Cl)nc2)c1	288.1	3.82	HDAC_2_4	F6	12855.22	842.41	0.064	0.061
D3	Cn1nnc(Nc2ccsc2)n1	223.0	4.25	HDAC_2_2	G2	12995.44	1168.97	0.053	0.085
D9	Cc1cc(C)c(Nc2ccc(CN)cn2)c(C)c1	283.2	4.33	HDAC_2_3	B4	13042.89	851.72	0.050	0.062
F5	O=C(NCc1ccccc1)Nc1nnn(C)n1	274.1	3.95	HDAC_2_4	B2	13045.56	1028.14	0.050	0.075
B3	c1ccc(-c2ccc(Nn3cnnc3)cc2)cc1	278.1	4.27	HDAC_2_1	F4	13053.00	1122.94	0.049	0.082
C2	FC(F)(F)c1ccc(Nn2cnnc2)cc1	270.1	4.17	HDAC_2_2	B2	13060.22	1243.14	0.048	0.091
G6	O=C(CO)N(C(c1ccccc1)c1ccccc1)n1cnnc1	0.0	4.15	HDAC_2_4	D5	13061.00	933.97	0.048	0.068
B9	c1cc2cc(Nn3cnnc3)ccc2[nH]1	241.1	1.94	HDAC_2_1	H3	13063.22	825.17	0.048	0.060
B7	Brc1cc(Br)cc(Nn2cnnc2)c1	357.9	4.43	HDAC_2_1	G5	13067.22	886.29	0.048	0.065
H3	COc1ccc(NC(=O)Nc2ccncc2Nn2cnnc2)cc1	367.1	3.83	HDAC_2_4	F1	13110.56	1308.08	0.045	0.095
D4	Cc1noc(C)c1NS(C)(=O)=O	0.0	-1.00	HDAC_2_2	G3	13131.56	1154.37	0.043	0.084
F2	N#Cc1ccccc1Nn1cnnc1	227.1	-1.00	HDAC_2_4	A6	13145.44	1267.46	0.042	0.092
A7	Clc1ccc(Nn2cnnc2)cc1	236.0	3.97	HDAC_2_1	D1	13206.22	966.23	0.038	0.070
E8	c1ccc2sc(Nn3cnnc3)nc2c1	#N/A	#N/A	HDAC_2_3	H1	13341.44	1605.59	0.027	0.117
C5	Cc1nnc(Nc2ccccc2Cl)s1	0.0	-1.00	HDAC_2_2	C1	13378.78	1095.46	0.025	0.080
A9	CS(=O)(=O)Nc1ccc(Nn2cnnc2)cc1	0.0	-1.00	HDAC_2_1	D6	13381.56	749.63	0.024	0.055
A3	CCc1ccc(Nn2cnnc2)cc1	0.0	-1.00	HDAC_2_1	A6	13383.56	1228.63	0.024	0.090
E6	COc1cc(N2CCS(=O)(=O)CC2)ccc1Cl	#N/A	#N/A	HDAC_2_3	G2	13396.78	1079.69	0.023	0.079
H6	FC(F)(F)Oc1ccc(Nc2ncccn2)cc1	297.1	5.17	HDAC_2_4	F4	13412.56	1053.24	0.022	0.077
A1-2	Clc1ccc(Nc2ccc(-c3ccccc3)cc2)cc1	321.1	4.73	HDAC_2_4	G3	13490.70	3863.07	0.016	0.282
D6	Nc1ccc(Nc2ncccn2)nc1	229.1	3.01	HDAC_2_2	H2	13550.89	1286.87	0.012	0.094
A8	Nc1ccncc1Nn1cnnc1	#N/A	#N/A	HDAC_2_1	D2	13558.00	1017.99	0.011	0.074
C10	COc1ncc(Nn2cnnc2)cn1	234.1	3.72	HDAC_2_2	D3	13579.00	775.89	0.010	0.057
D7	NCc1ccc(Nc2ncccn2)nc1	243.1	4.35	HDAC_2_3	A3	13583.44	1220.04	0.009	0.089
C6	Nc1ccc(Nn2cnnc2)nc1	#N/A	#N/A	HDAC_2_2	C3	13601.00	1042.03	0.008	0.076
B8	N#Cc1ccc(CNc2cc3ccccc3o2)cc1	372.1	7.12	HDAC_2_1	H1	13660.22	898.04	0.004	0.066
H10	DMSO	#N/A	#N/A	DMSO	DMSO	13708.33	1160.34	0.000	0.085
F4	O=C(Nc1ncccn1)c1sc2cc(F)ccc2c1Cl	0.0	-1.00	HDAC_2_4	B1	13913.67	1151.68	-0.015	0.084
A10	c1nc(NC23CC4CC(CC(C4)C2)C3)c2[nH]cnc2n1	311.2	3.98	HDAC_2_1	E3	13950.44	664.84	-0.018	0.048
E2	O=[N+]([O-])c1ccc(Nc2nn[nH]n2)cc1	#N/A	#N/A	HDAC_2_3	E1	13993.44	1389.58	-0.021	0.101
A2	c1ccc2ncc(Nn3cnnc3)cc2c1	253.1	3.25	HDAC_2_1	A3	14085.22	1325.30	-0.028	0.097

D8	c1cnc(Nc2cnc3ccccc3n2)nc1	0.0	-1.00	HDAC_2_3	B3	14108.67	925.06	-0.030	0.067
F3	O=S(=O)(Nn1cnnc1)C(F)(F)F	0.0	-1.00	HDAC_2_4	A7	14247.33	1789.95	-0.040	0.131
C8	CC(C)(C)Nc1cc2ccccc2o1	231.1	4.48	HDAC_2_2	D1	14350.44	889.44	-0.048	0.065
B4	O=S1(=O)CCN(c2cc3ccccc3o2)CC1	293.1	6.26	HDAC_2_1	G2	14390.89	1632.95	-0.051	0.119
B1	c1ccc2oc(Nn3cnnc3)cc2c1	242.1	1.96	HDAC_2_1	F1	14640.22	1362.68	-0.070	0.099
G9	Cc1cccc(CNc2ncn[nH]2)c1	0.0	-1.00	HDAC_2_4	E1	15267.11	883.38	-0.116	0.064
G5	FC(F)(F)c1ccc(-c2ccc(Nc3cnc4ccccc4c3)cc2)nc1	407.1	5.52	HDAC_2_4	D4	16219.67	1741.30	-0.188	0.127
H9	c1cc2cn[nH]c2cc1NCC1CCCCC1	271.2	4.87	HDAC_2_4	G2	16302.89	1478.11	-0.194	0.108
G10	CCn1c2ccccc2c2cc(CNc3ccncc3Nn3cnnc3)ccc21	425.2	5.48	HDAC_2_4	E2	17518.11	1517.24	-0.285	0.111
G4	Cc1ccccc1N(c1ccc2c(C(F)(F)F)cc(=O)oc2c1)S(=O)(=O)c1cccs1	507.0	6.82	HDAC_2_4	D3	26454.22	2650.62	-0.952	0.193
E11	O=S(=O)(O)c1ccc(NCc2ccc(-c3ccsc3)s2)cc1	393.0	5.12	HDAC_2_4	A2	59885.78	4188.98	-3.449	0.306

REFERENCES

(1) Tu, Z.; Choure, S. J.; Fong, M. H.; Roh, J.; Levin, I.; Yu, K.; Joung, J. F.; Morgan, N.; Li, S.-C.; Sun, X. ASKCOS: an open source software suite for synthesis planning. *arXiv preprint arXiv:2501.01835* **2025**.
(2) Fromer, J. C.; Coley, C. W. An algorithmic framework for synthetic cost-aware decision making in molecular design. *Nature Computational Science* **2024**, *4* (6), 440-450. DOI: 10.1038/s43588-024-00639-y.
(3) Rogers, D.; Hahn, M. Extended-connectivity fingerprints. *J. Chem. Inf. Model.* **2010**, *50* (5), 742.
(4) Moriwaki, H.; Tian, Y.-S.; Kawashita, N.; Takagi, T. Mordred: a molecular descriptor calculator. *Journal of Cheminformatics* **2018**, *10* (1), 4. DOI: 10.1186/s13321-018-0258-y.
(5) Jaeger, S.; Fulle, S.; Turk, S. Mol2vec: Unsupervised Machine Learning Approach with Chemical Intuition. *Journal of Chemical Information and Modeling* **2018**, *58* (1), 27-35. DOI: 10.1021/acs.jcim.7b00616.
(6) Griffiths, R.-R.; Klarner, L.; Moss, H.; Ravuri, A.; Truong, S.; Du, Y.; Stanton, S.; Tom, G.; Rankovic, B.; Jamasb, A. Gauche: A library for Gaussian processes in chemistry. *Advances in Neural Information Processing Systems* **2024**, *36*.
(7) Koscher, B. A.; Canty, R. B.; McDonald, M. A.; Greenman, K. P.; McGill, C. J.; Bilodeau, C. L.; Jin, W.; Wu, H.; Vermeire, F. H.; Jin, B.; et al. Autonomous, multiproperty-driven molecular discovery: From predictions to measurements and back. *Science* **2023**, *382* (6677), eadi1407. DOI: doi:10.1126/science.adi1407.
(8) McDonald, M. A.; Koscher, B. A.; Canty, R. B.; Jensen, K. F. Calibration-free reaction yield quantification by HPLC with a machine-learning model of extinction coefficients. *Chemical Science* **2024**, *15* (26), 10092-10100, 10.1039/D4SC01881H. DOI: 10.1039/D4SC01881H.
(9) Kazmi, M. Z. H.; Schneider, O. M.; Hall, D. G. Expanding the Role of Boron in New Drug Chemotypes: Properties, Chemistry, Pharmaceutical Potential of Hemiboronic Naphthoids. *Journal of Medicinal Chemistry* **2023**, *66* (19), 13768-13787. DOI: 10.1021/acs.jmedchem.3c01194.
