# Supplementary material for: Bayesian Optimization over Multiple Experimental Fidelities Accelerates Automated Discovery of Drug Molecules
Source: ACS Cent Sci. 2025 Feb 5;11(2):346–56. doi: 10.1021/acscentsci.4c01991 (PMC11869128; doi:10.1021/acscentsci.4c01991)

All Synthesis Pathways from iteration 1

Product 1

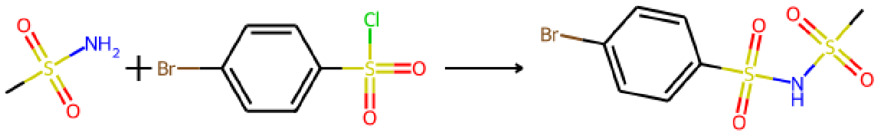

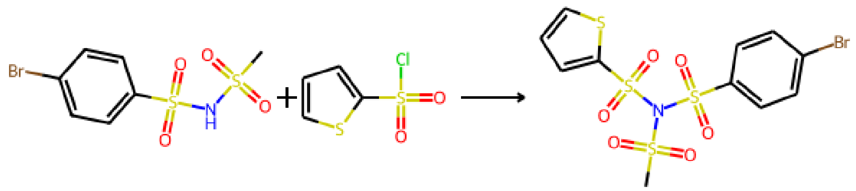

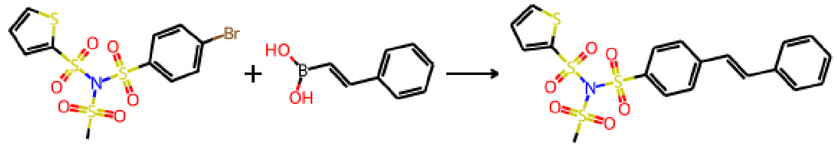

Product 2

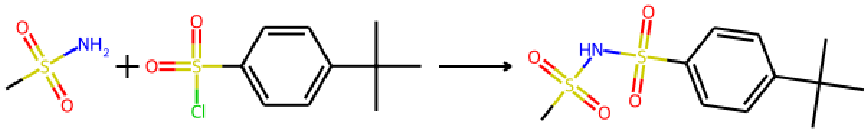

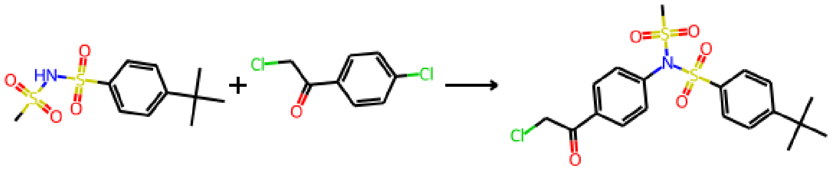

Product 3

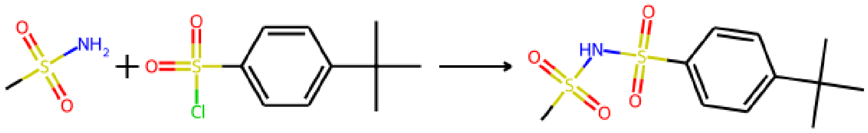

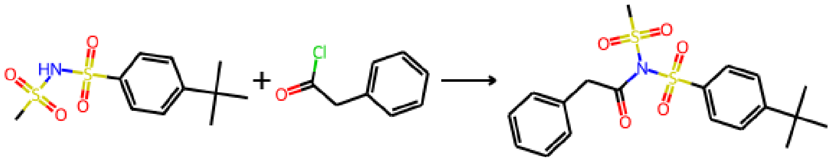

Product 4

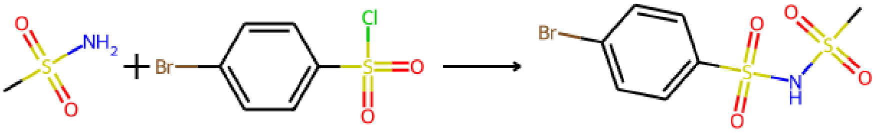

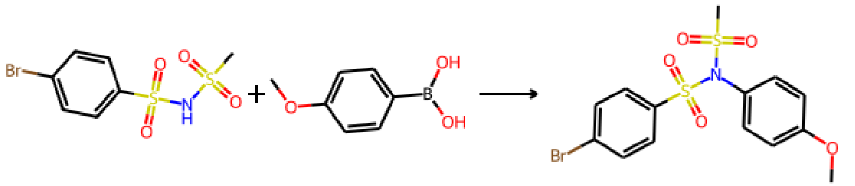

Product 5

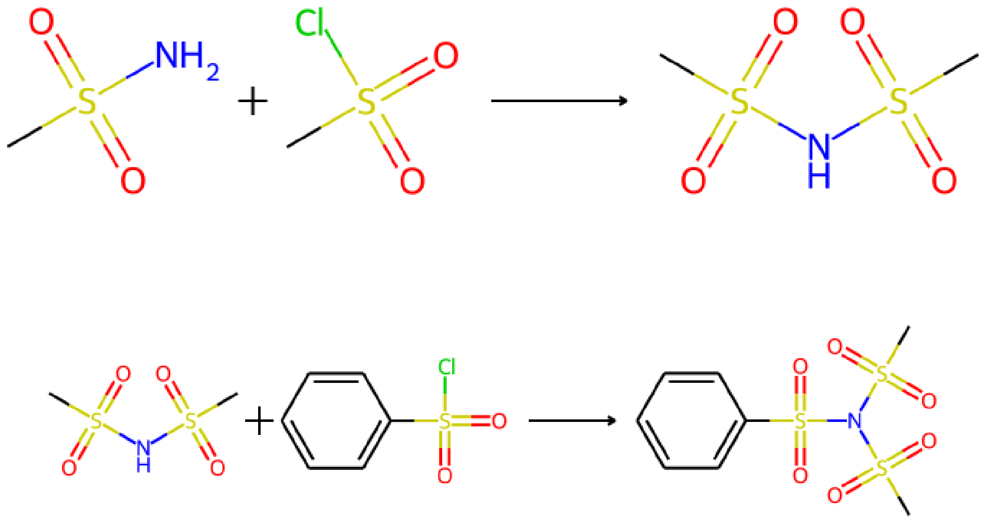

Product 6

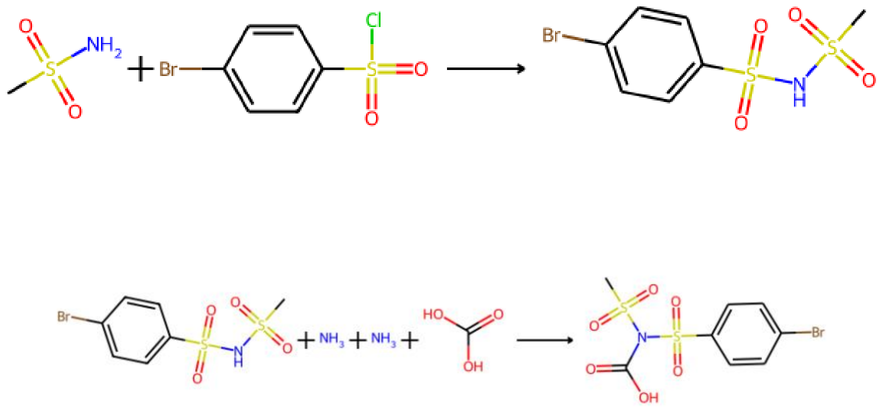

Product 7

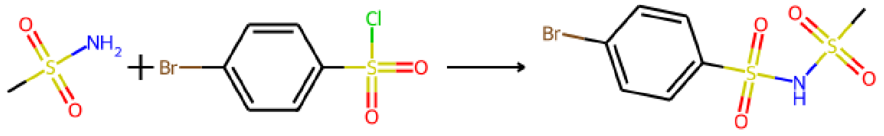

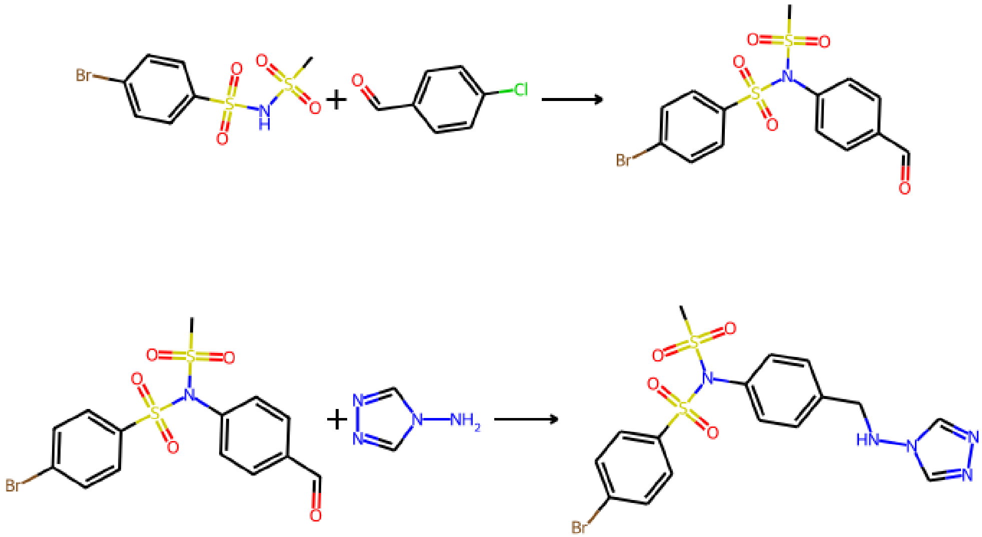

Product 8

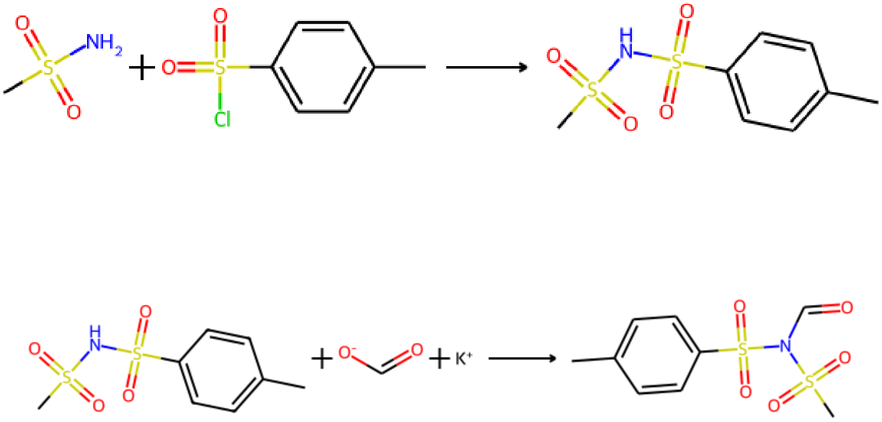

Product 9

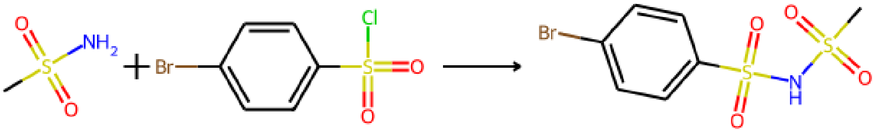

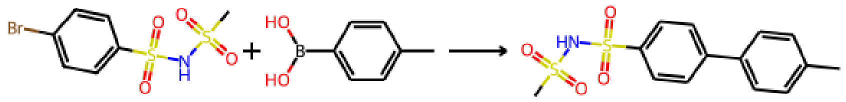

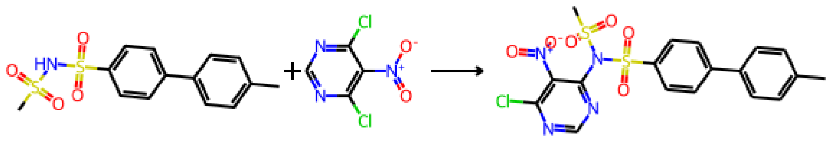

Product 10

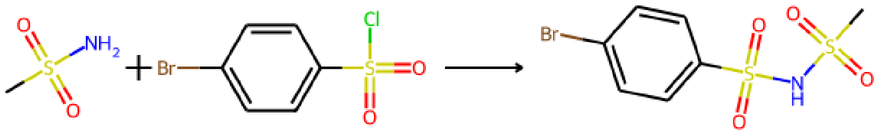

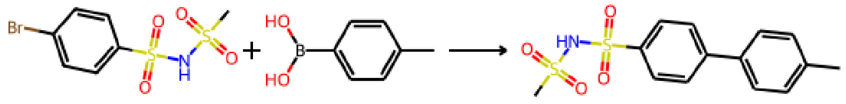

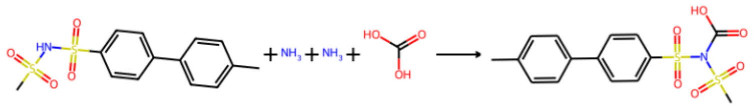

Product 11

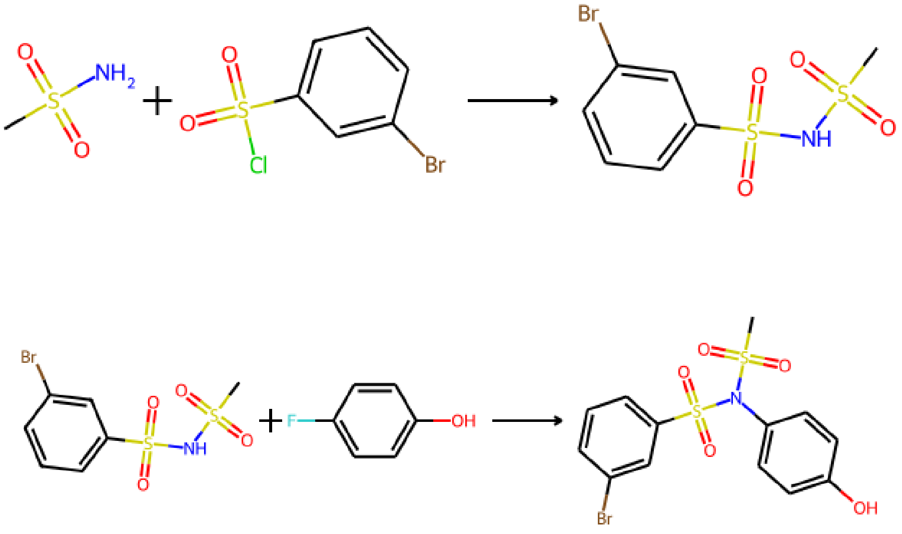

Product 12

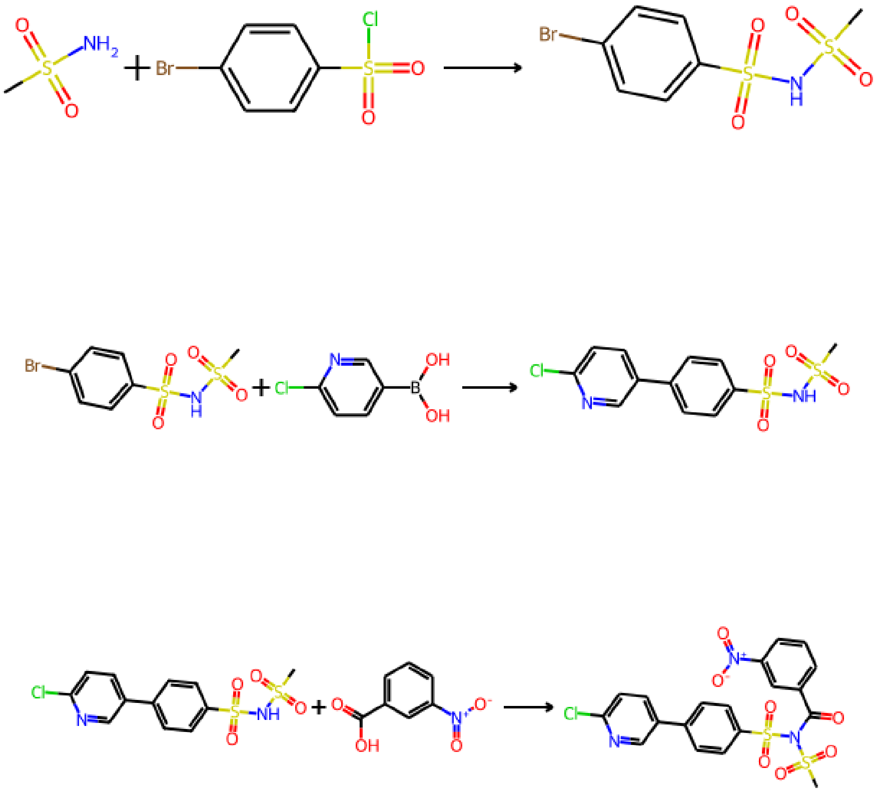

Product 13

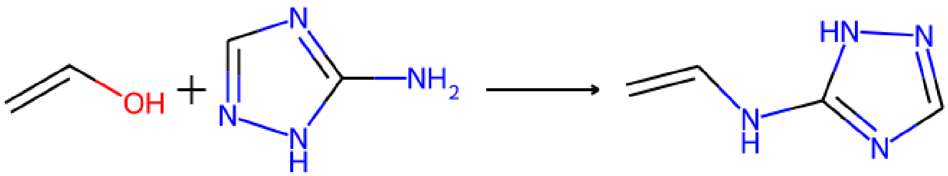

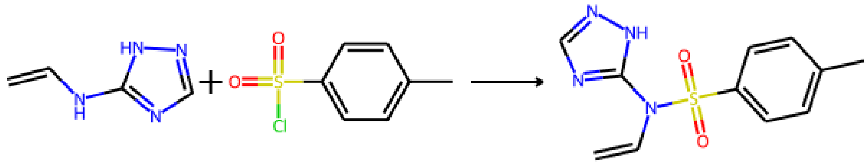

Product 14

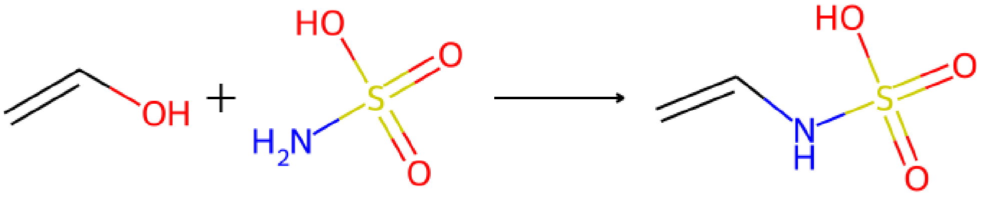

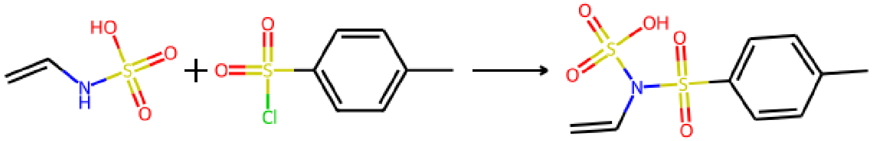

Product 15

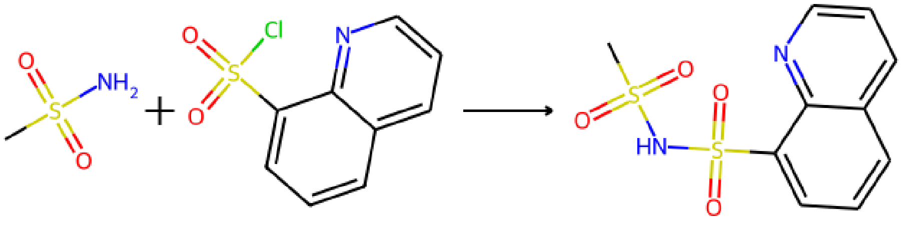

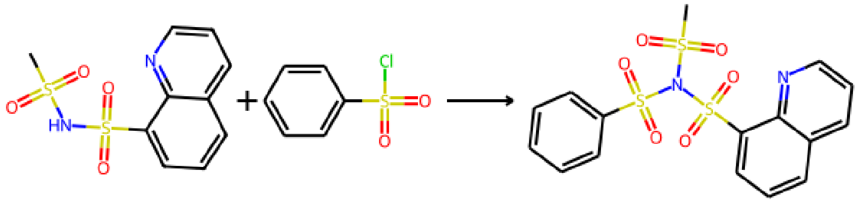

Product 16

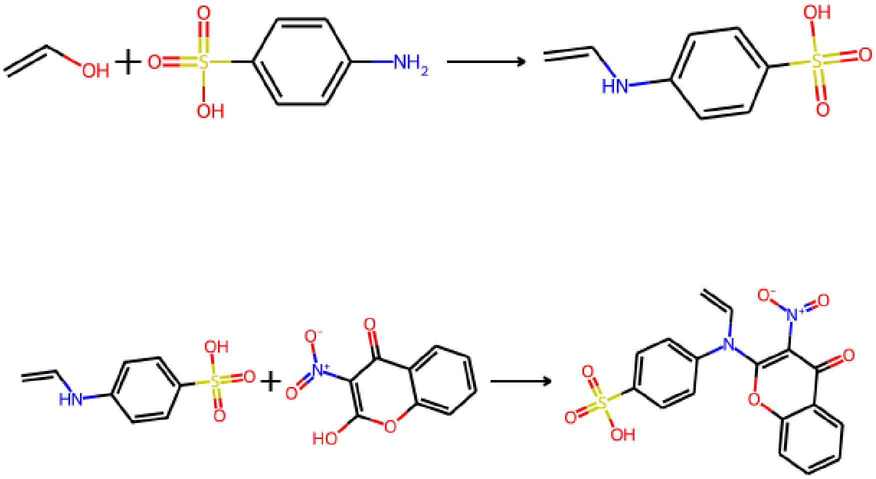

Product 17

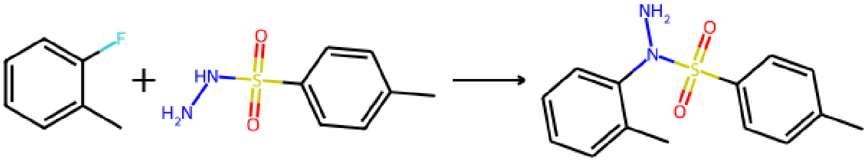

Product 18

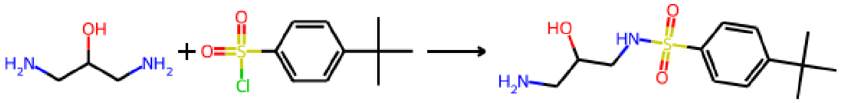

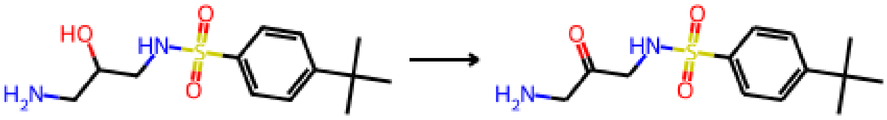

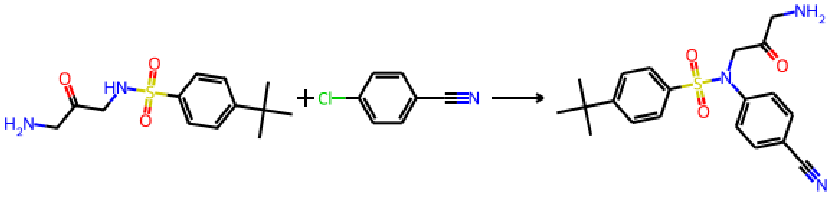

Product 19

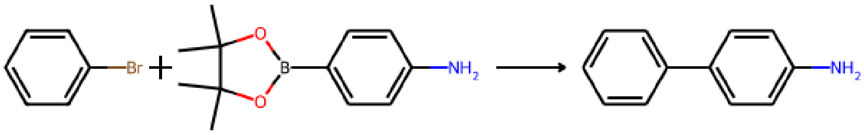

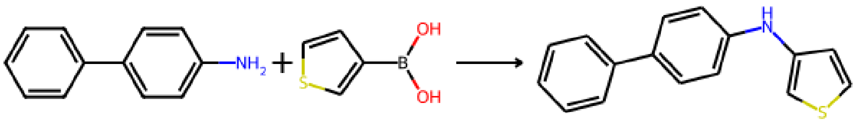

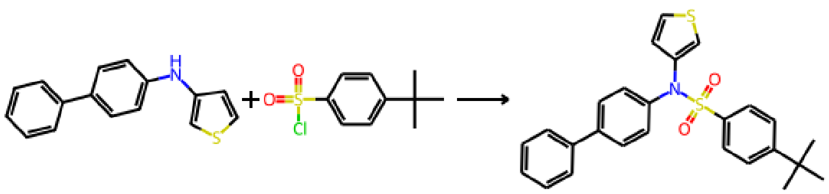

Product 20

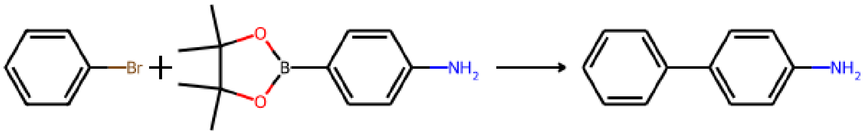

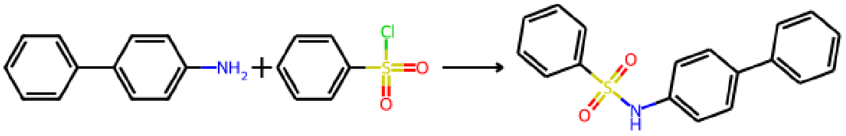

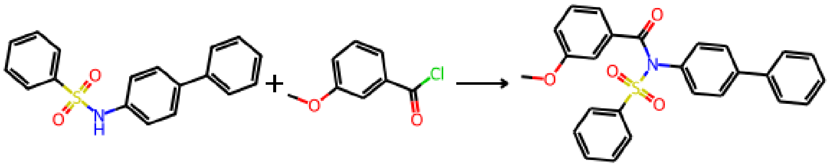

Product 21

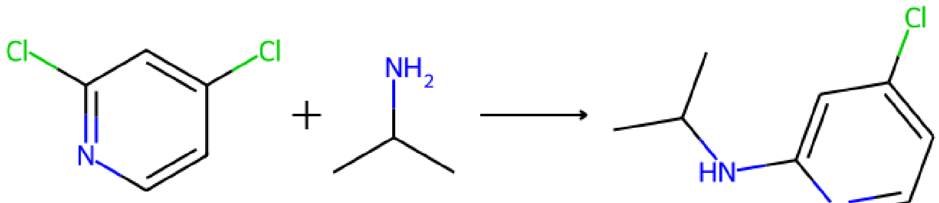

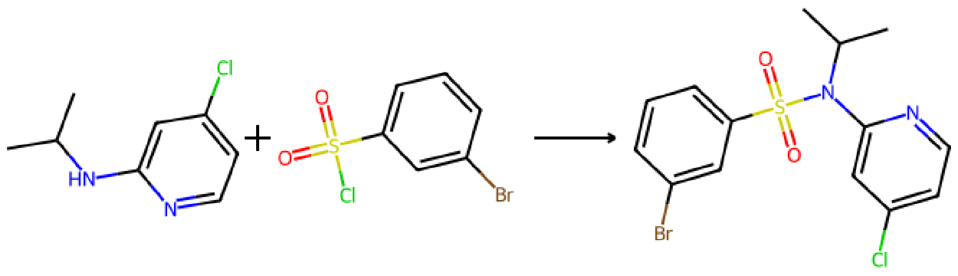

Product 22

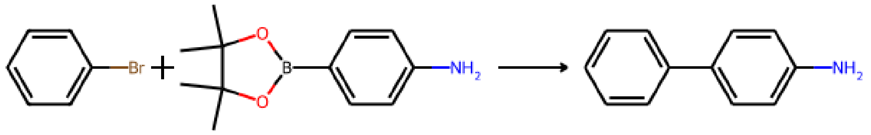

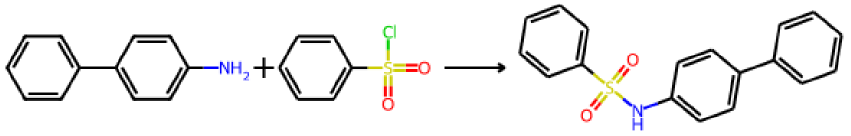

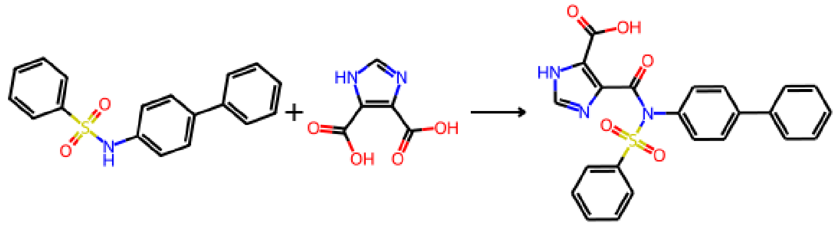

Product 23

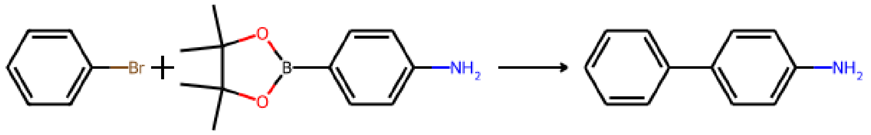

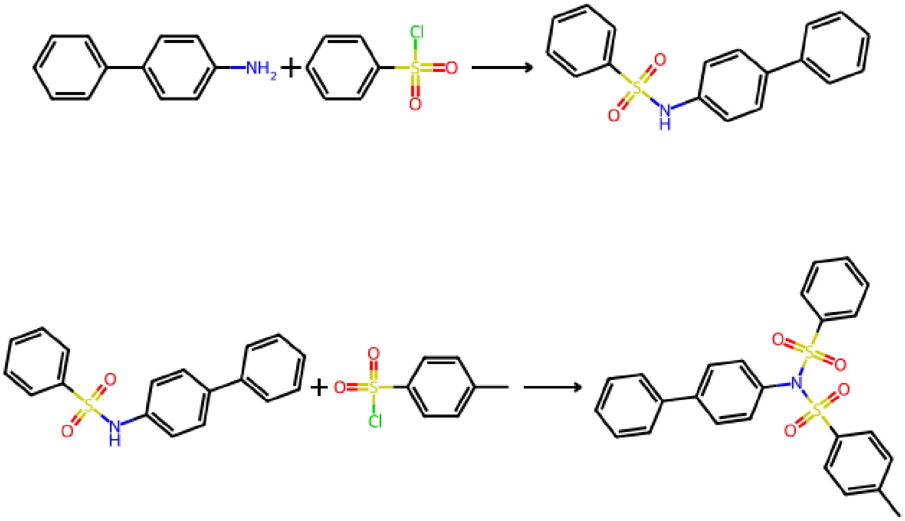

Product 24

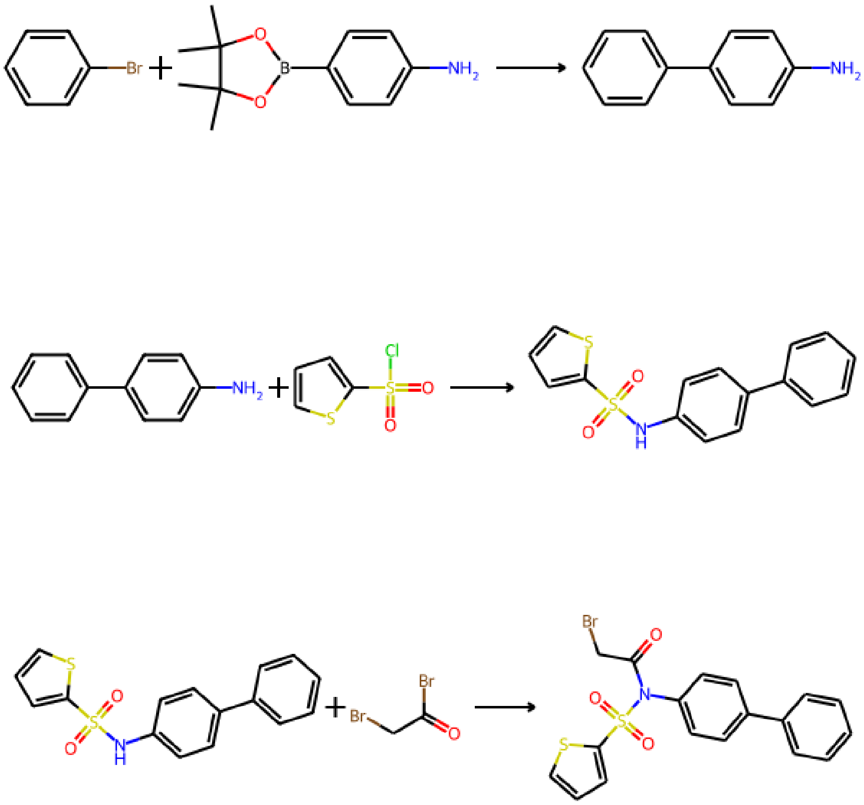

Product 25

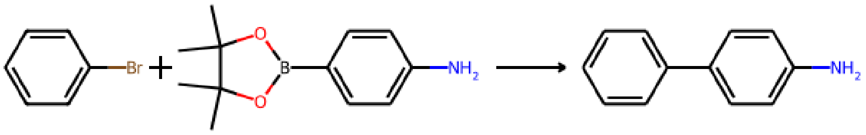

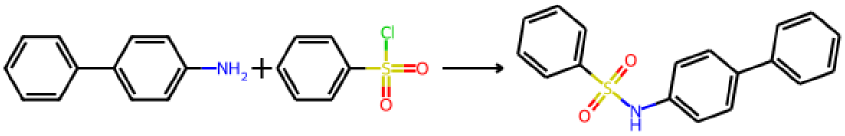

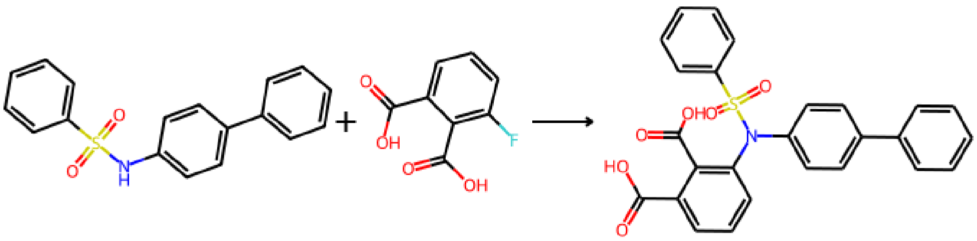

Product 26

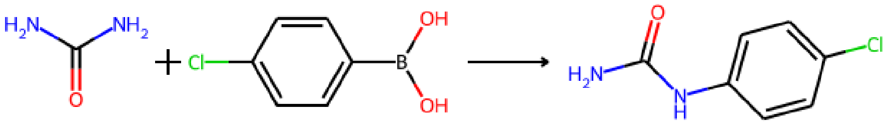

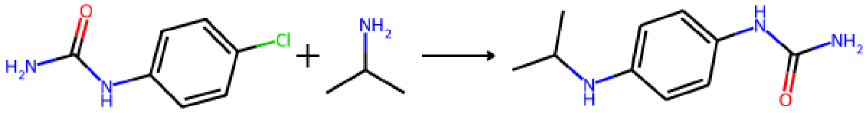

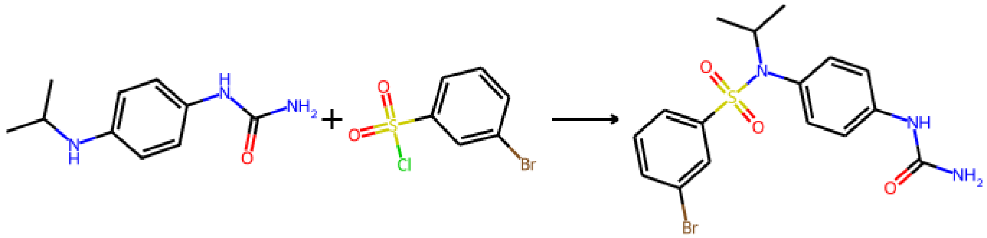

Product 27

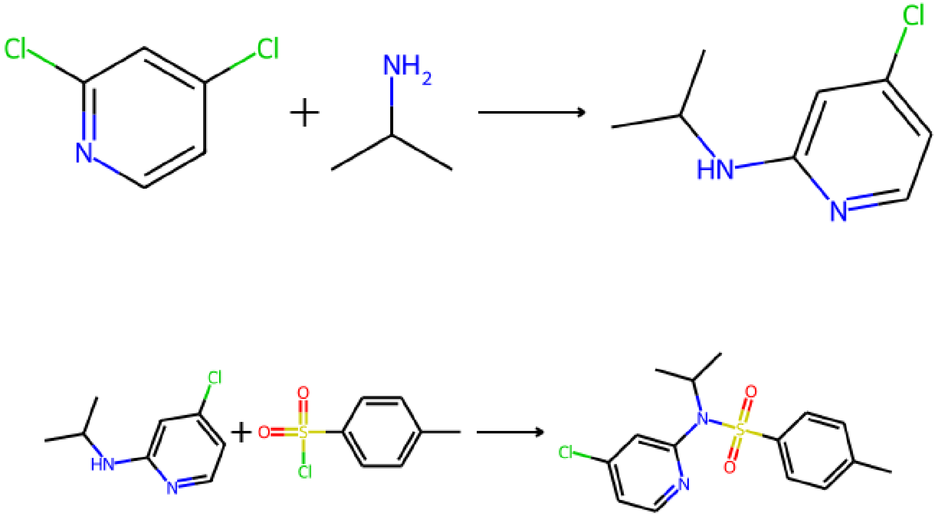

Product 28

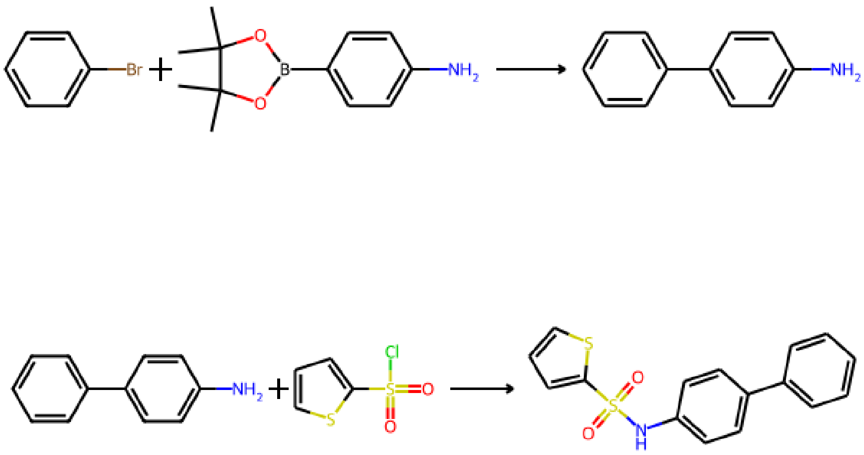

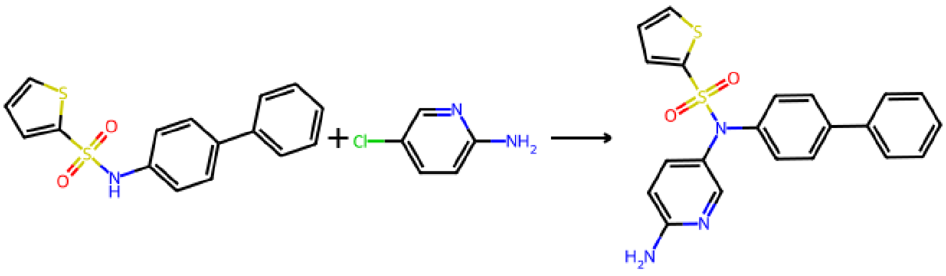

Product 29

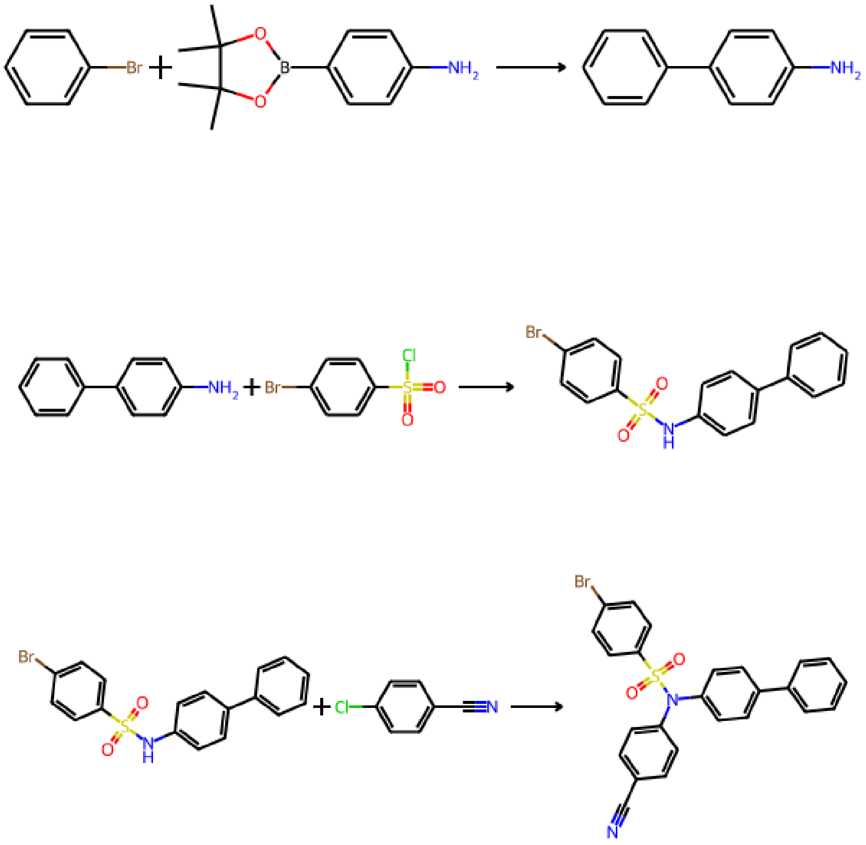

Product 30

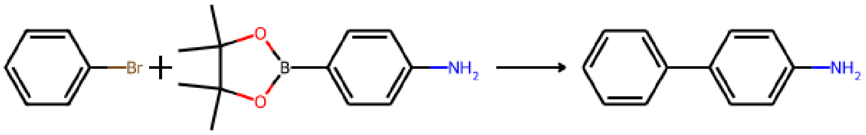

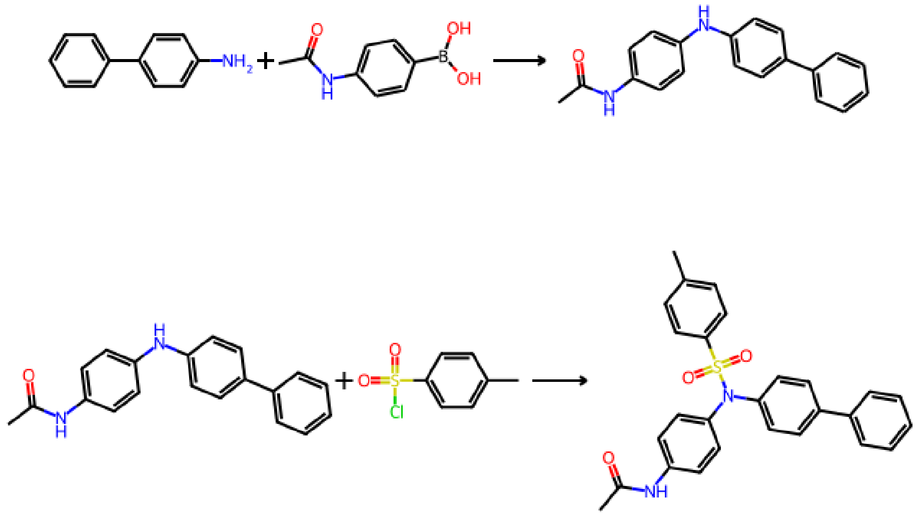

Product 31

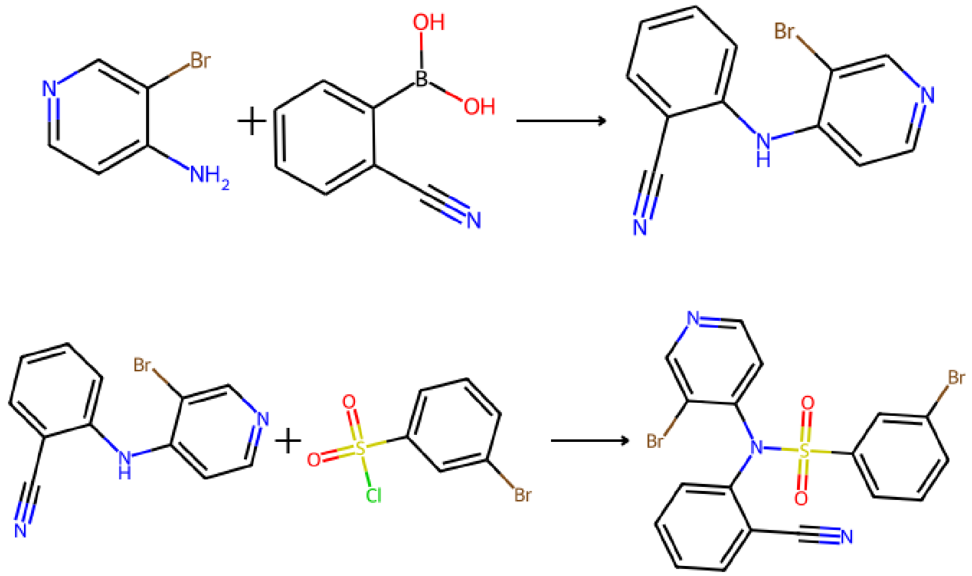

Product 32

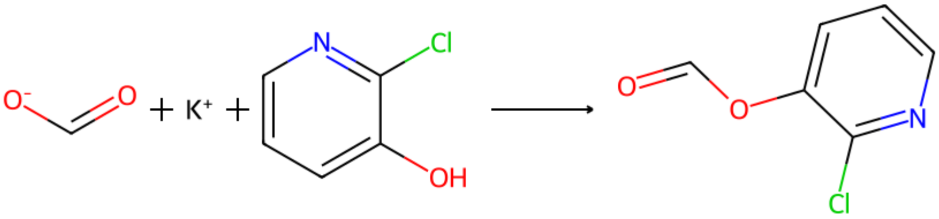

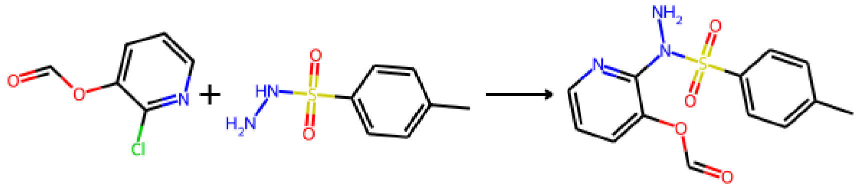

Product 33

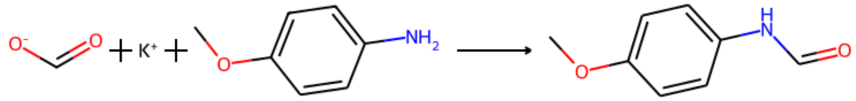

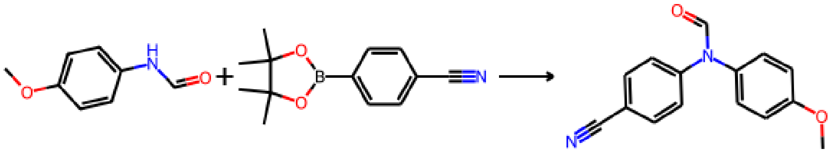

Product 34

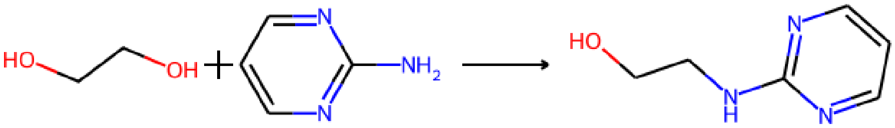

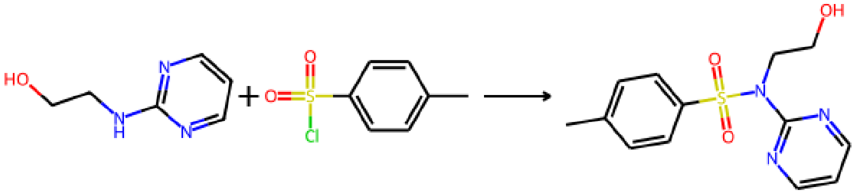

Product 35

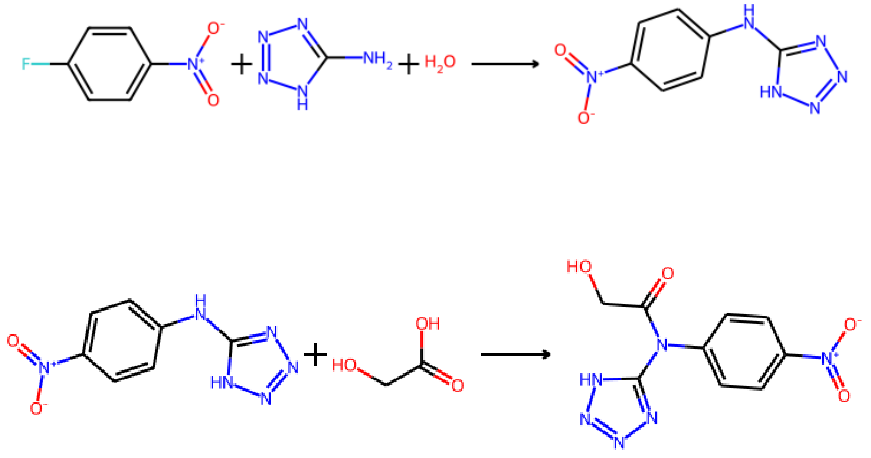

Product 36

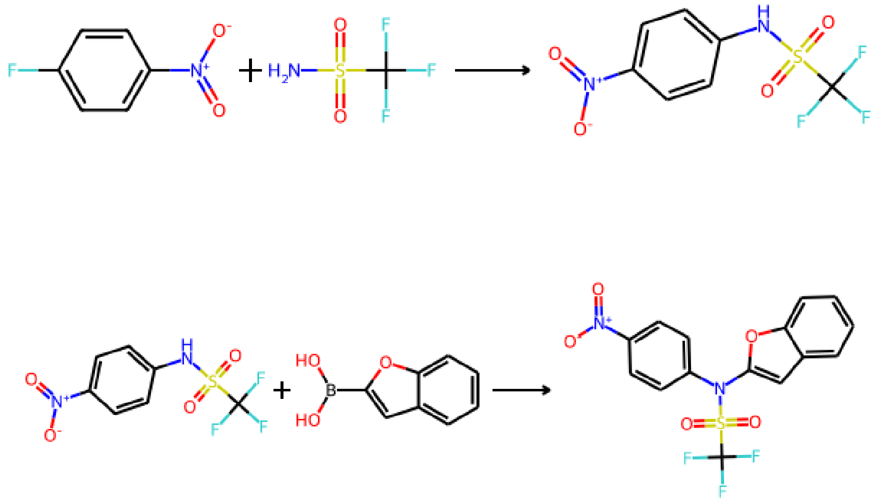

Product 37

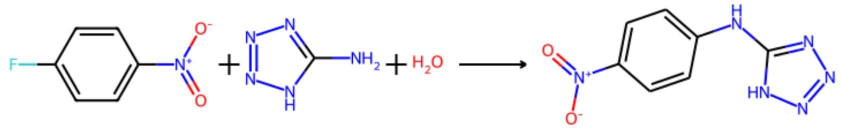

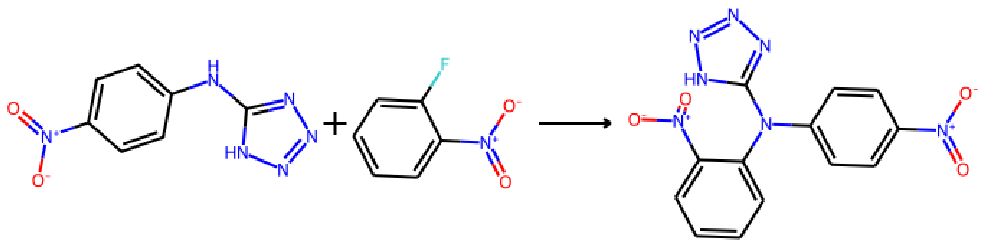

Product 38

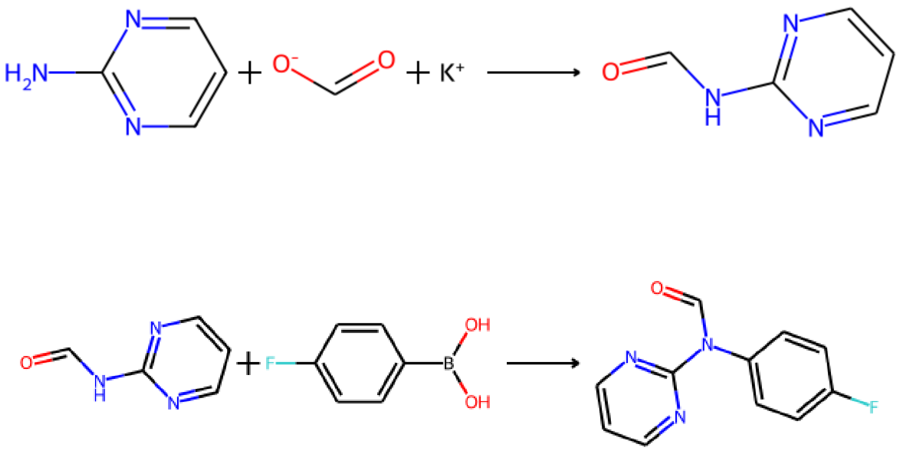

Product 39

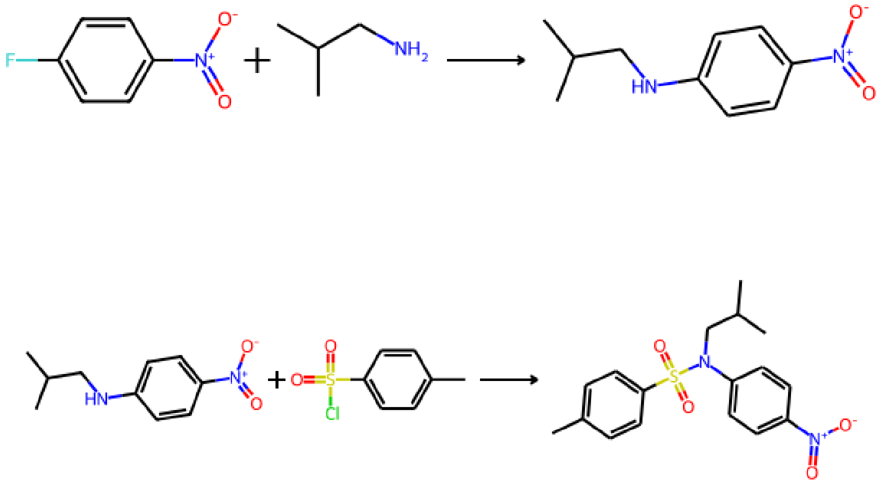

Product 40

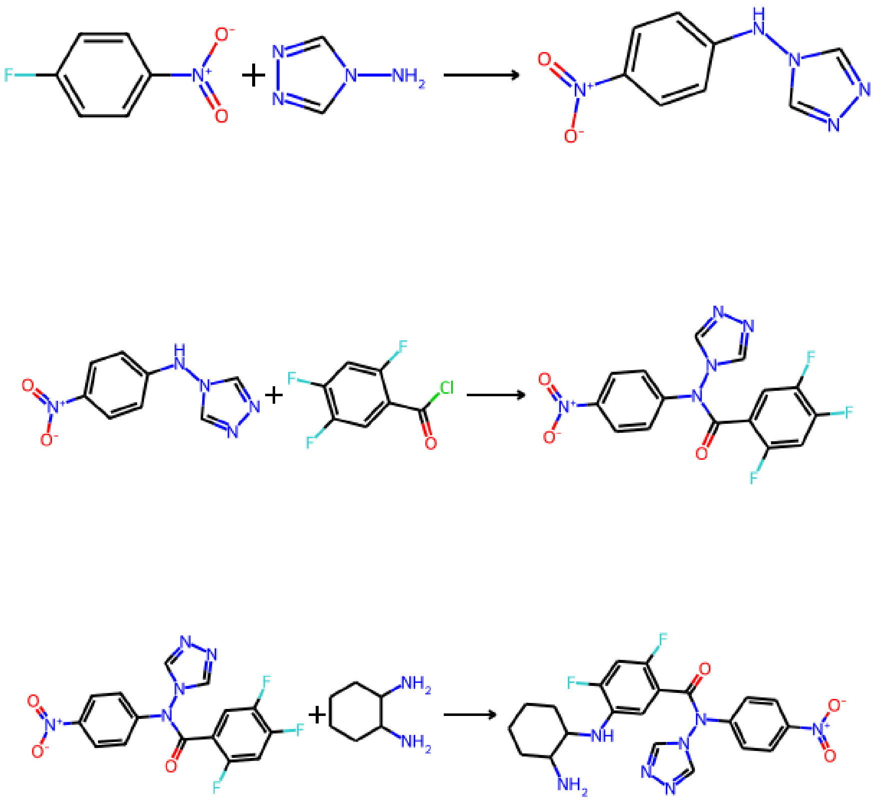

Product 41

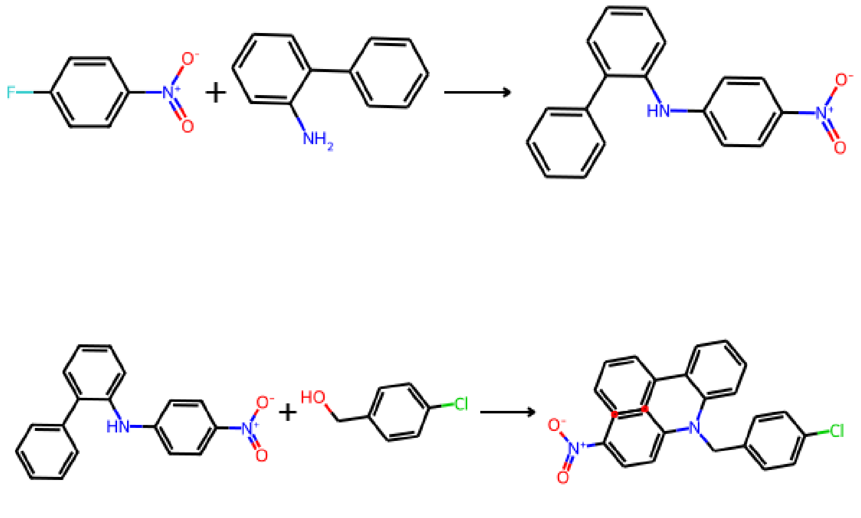

Product 42

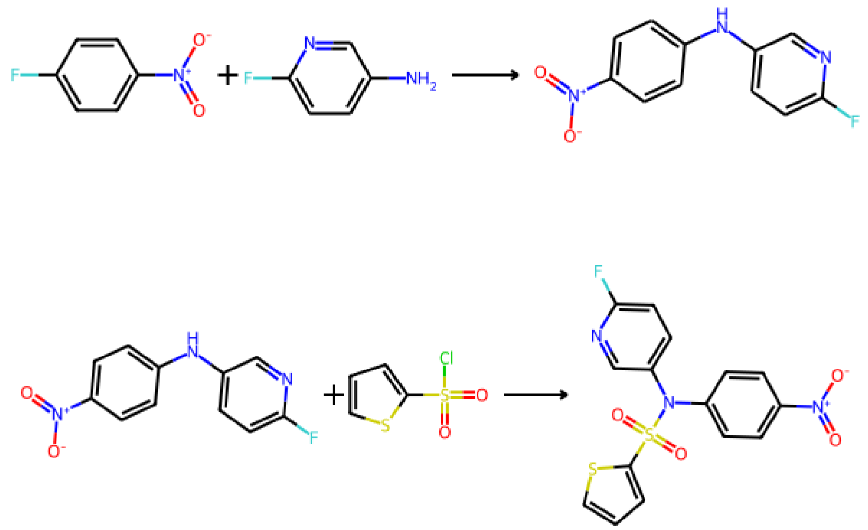

Product 43

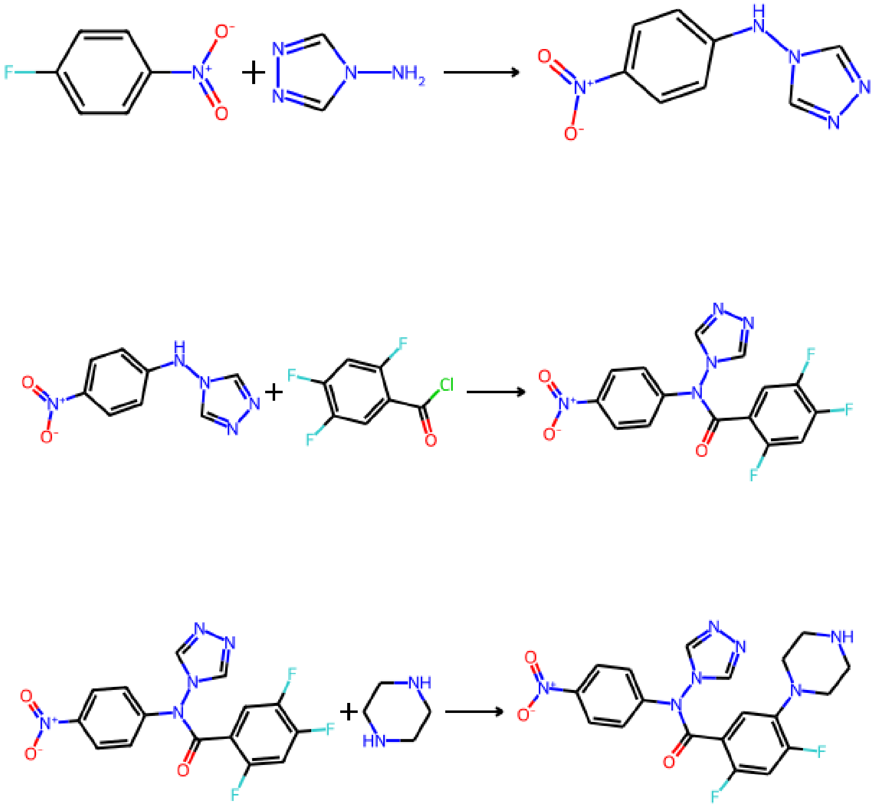

Product 44

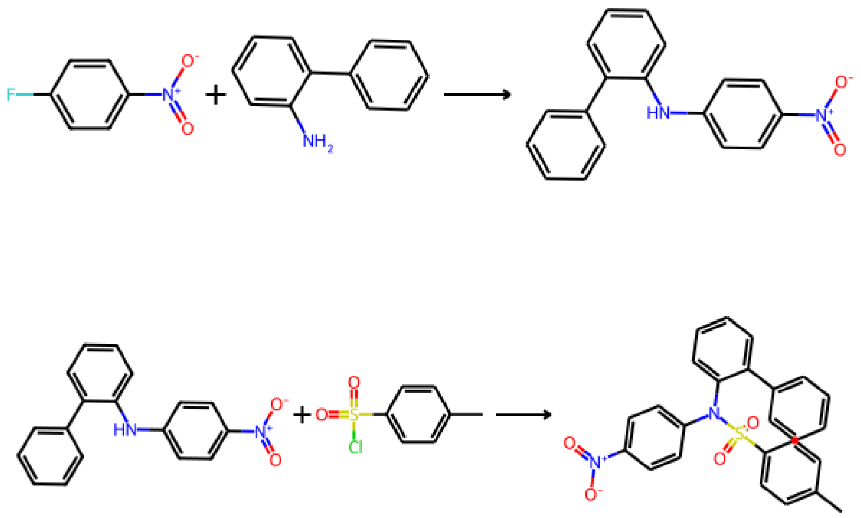

Product 45

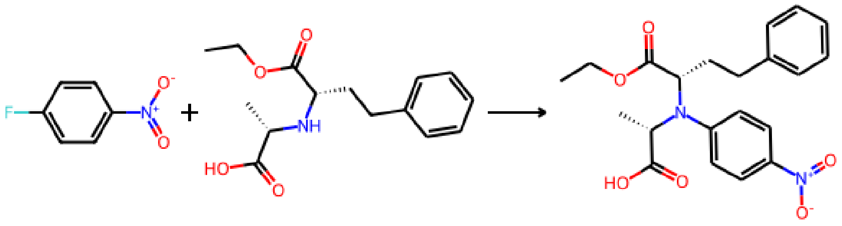

Product 46

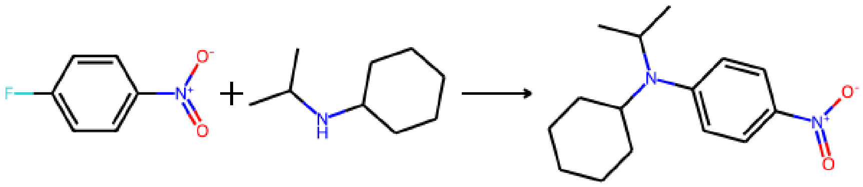

Product 47

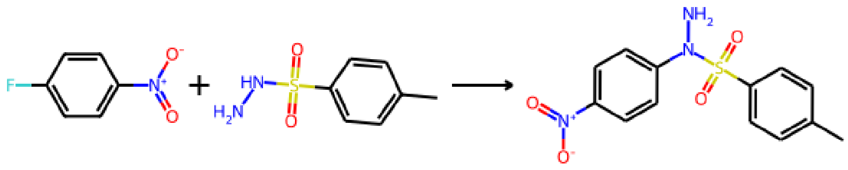

Product 48

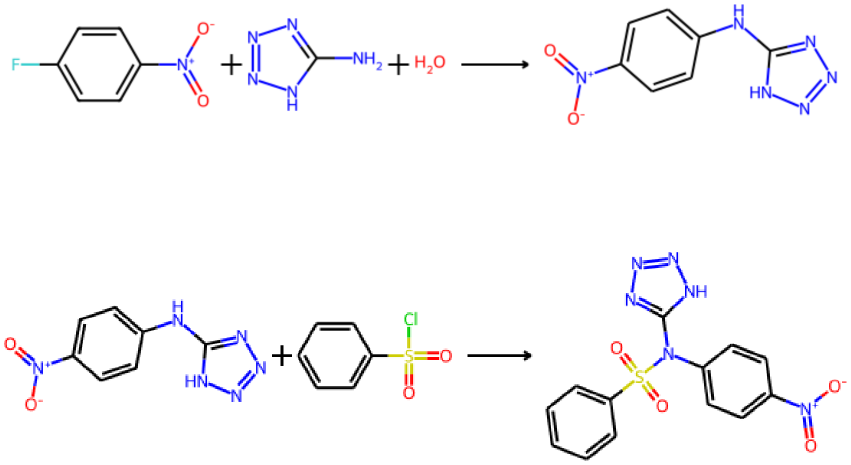

Product 49

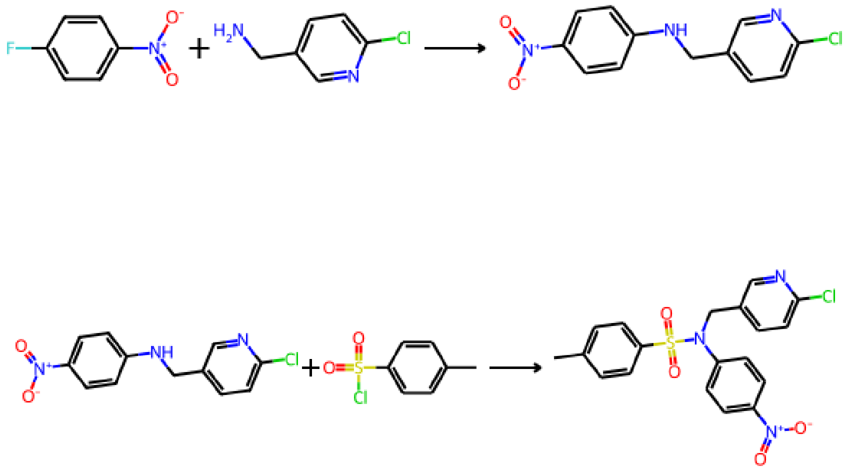

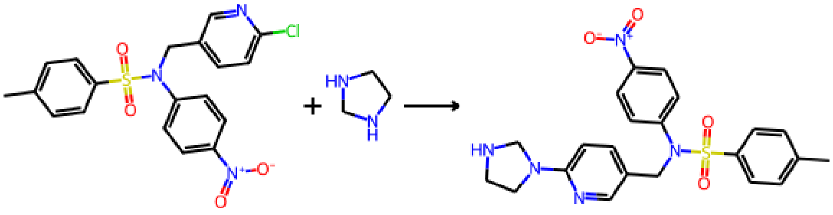

Product 50

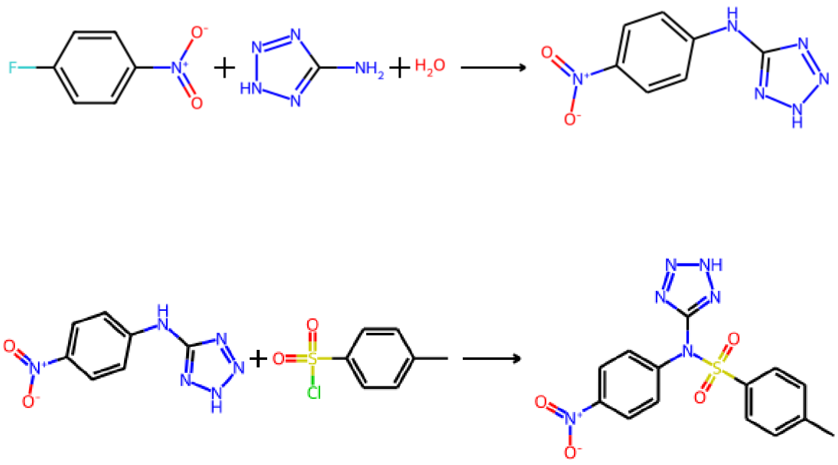

Product 51

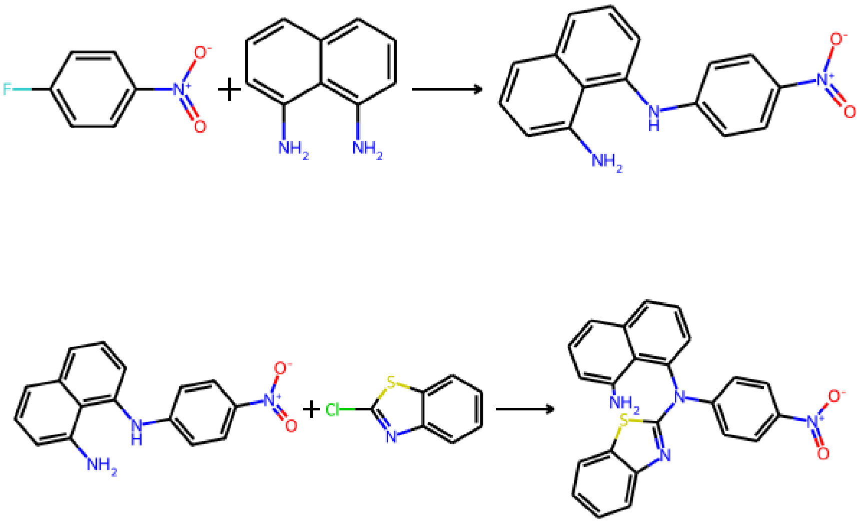

Product 52

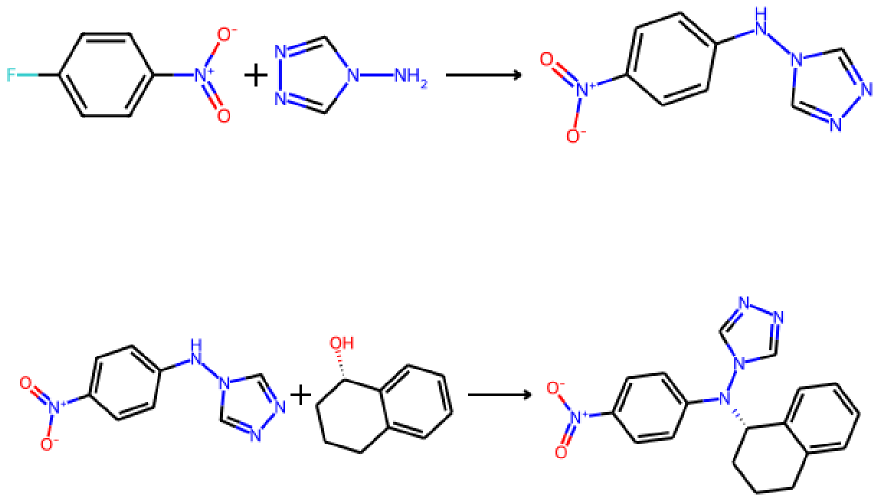

Product 53

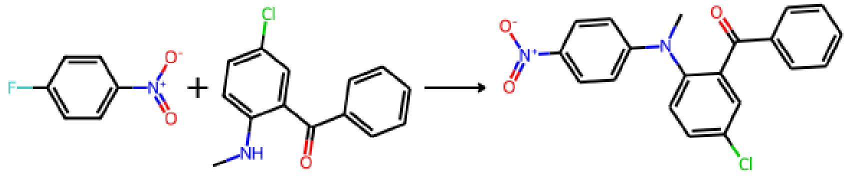

Product 54

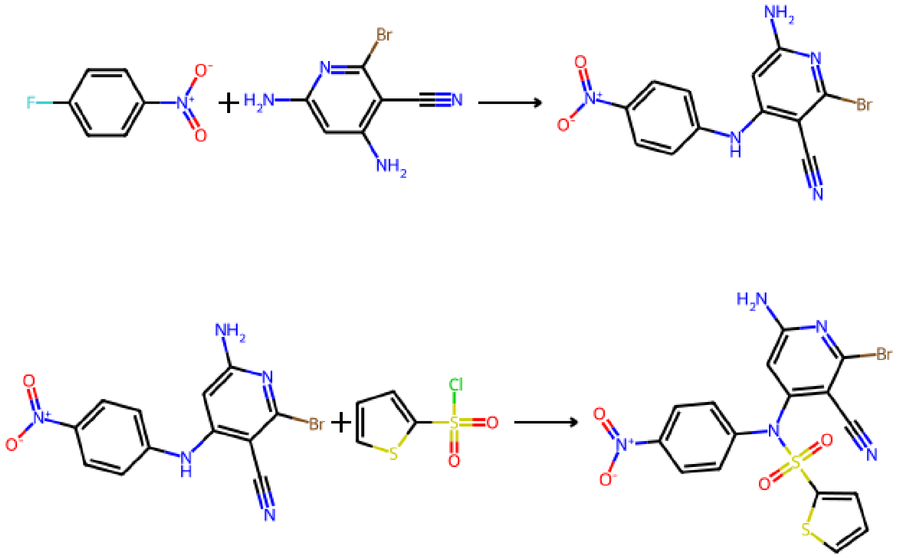

Product 55

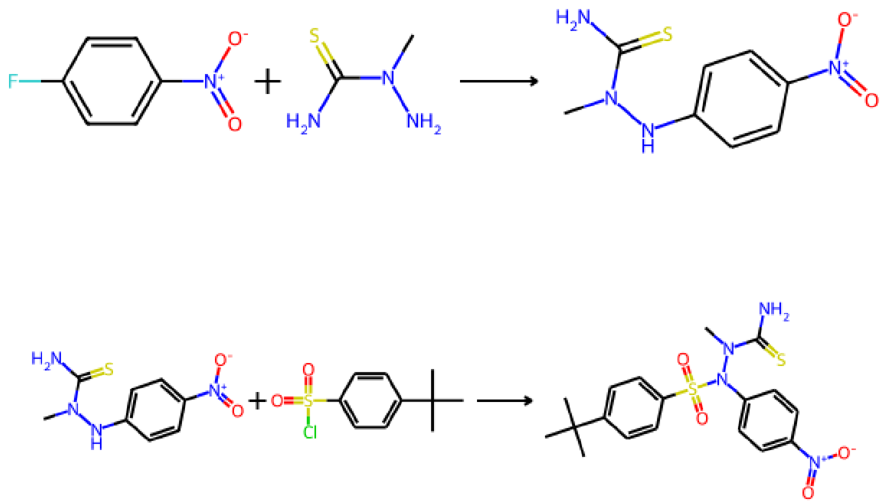

Product 56

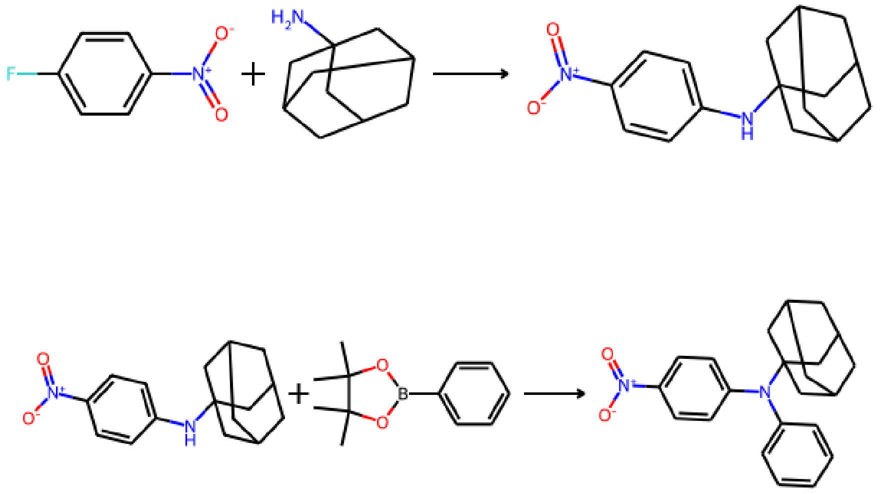

Product 57

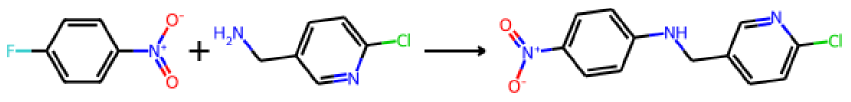

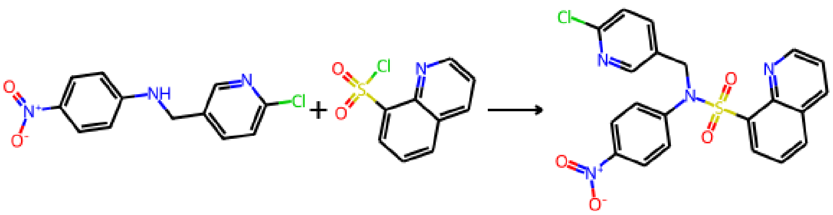

Product 58

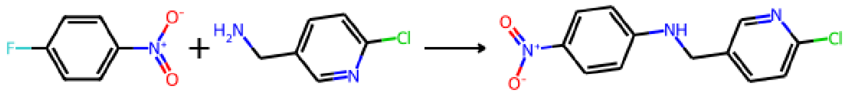

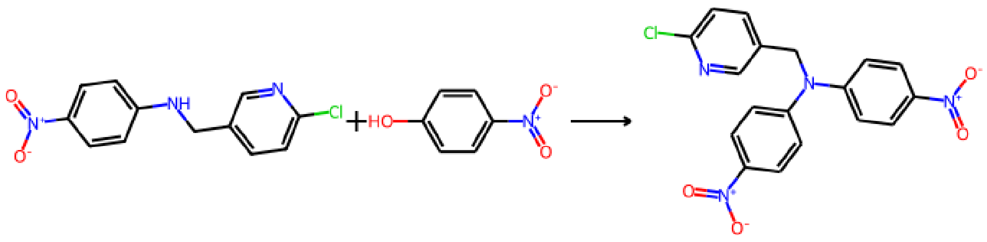

Product 59

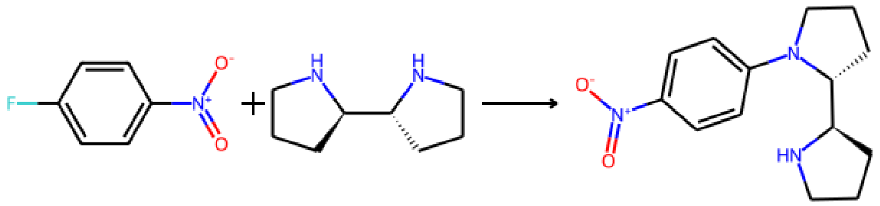

Product 60

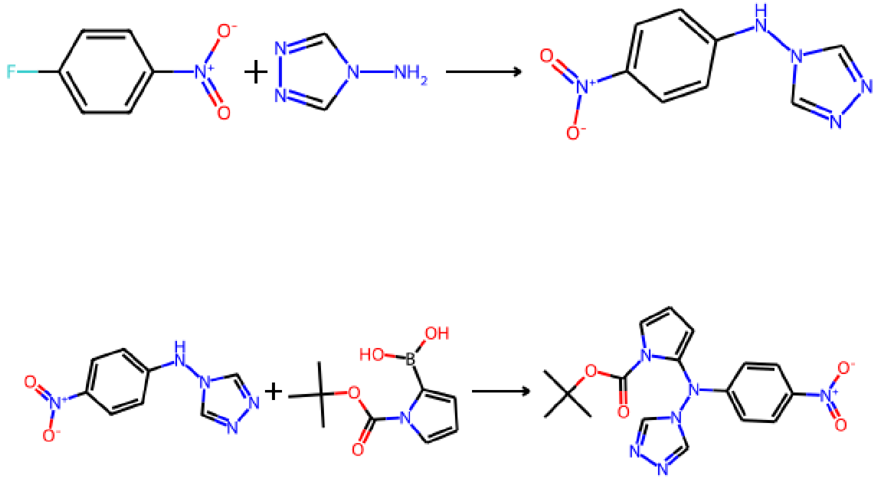

Product 61

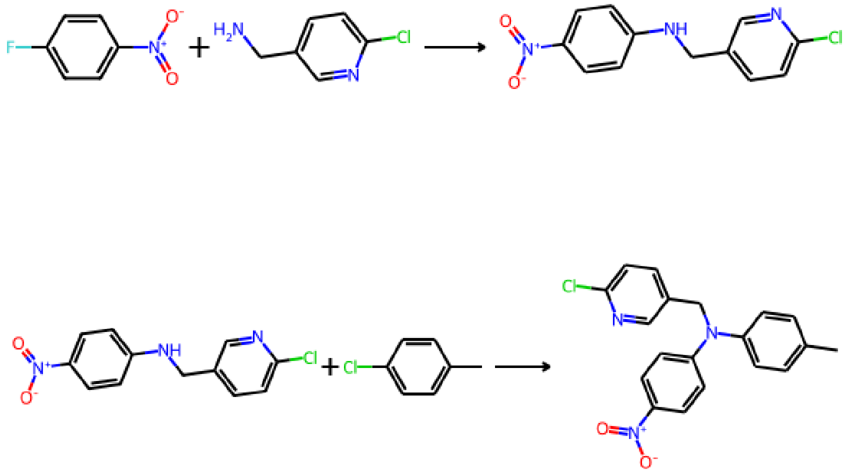

Product 62

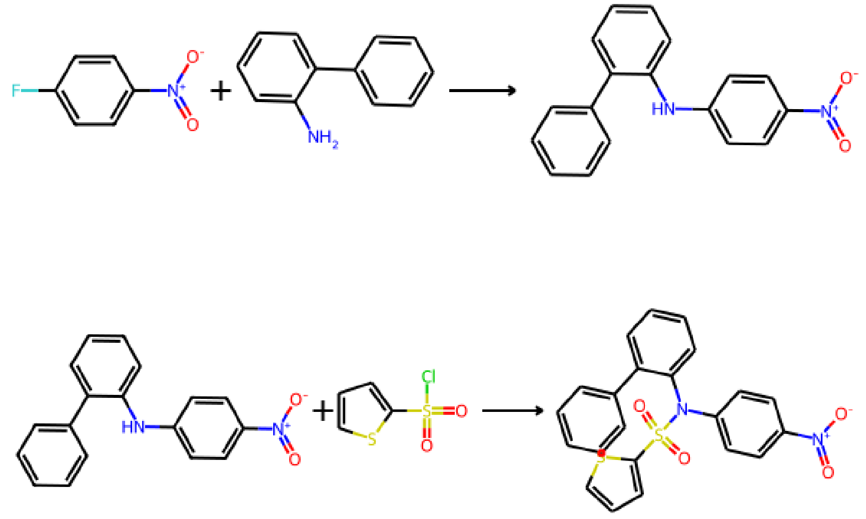

Product 63

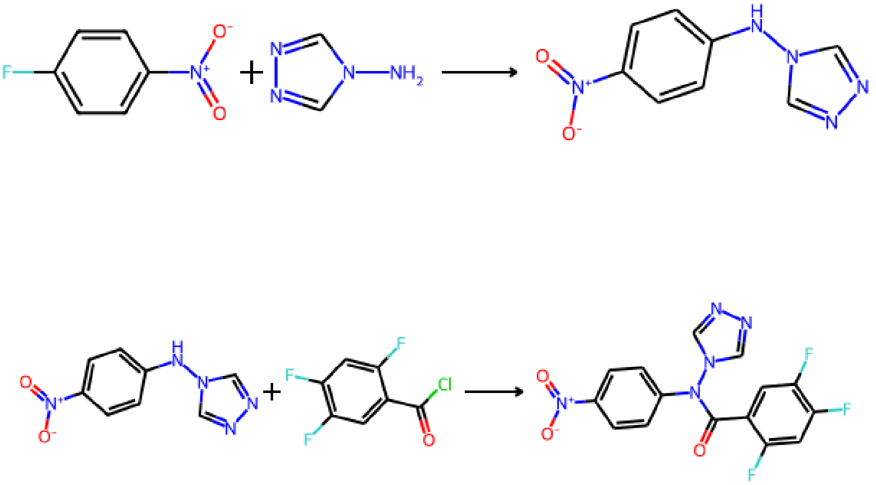

Product 64

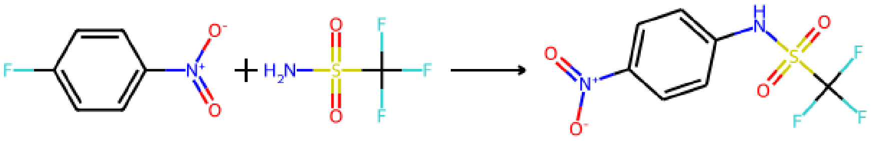

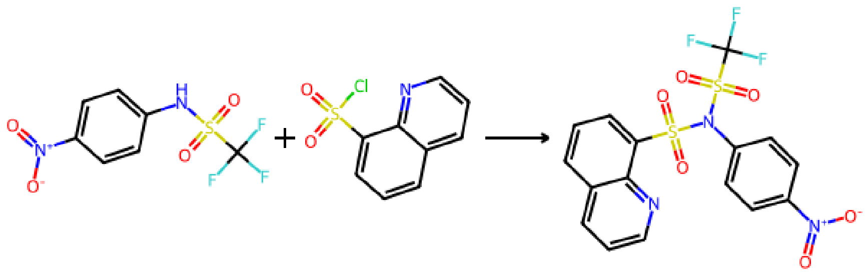

Product 65

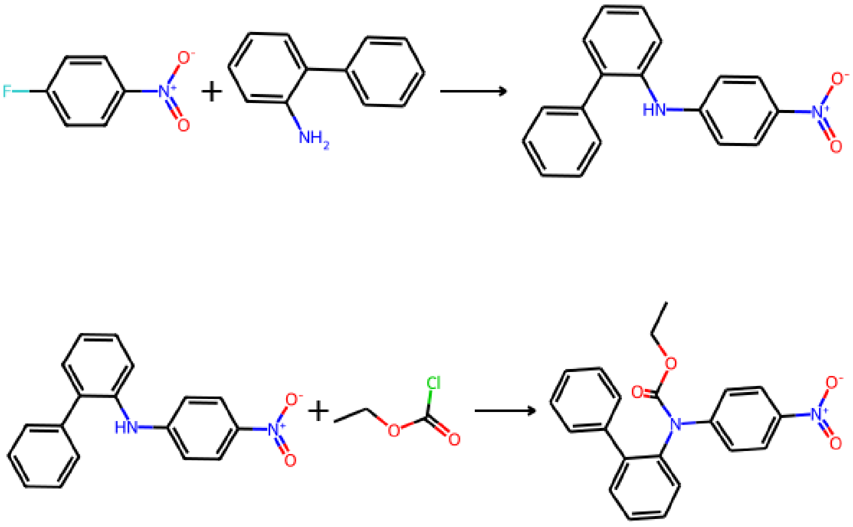

Product 66

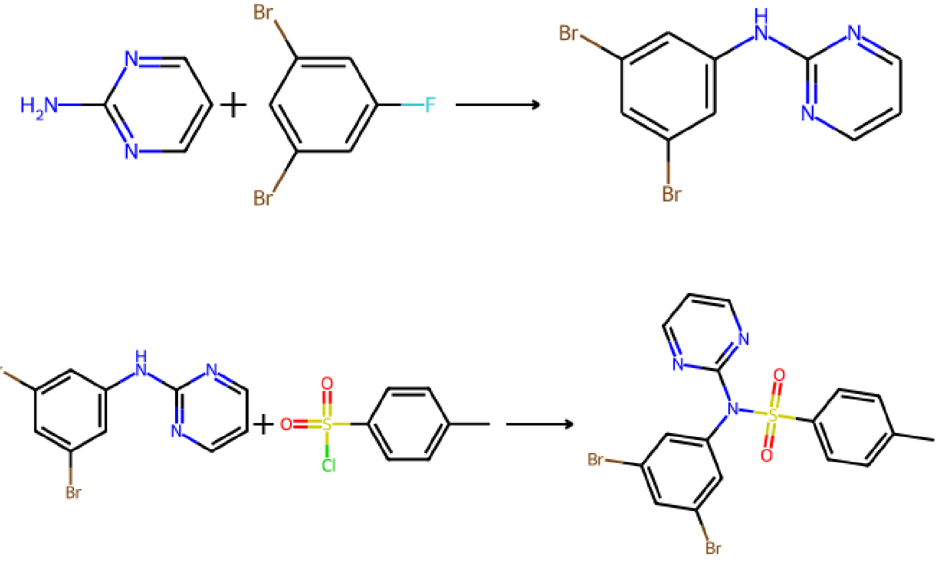

Product 67

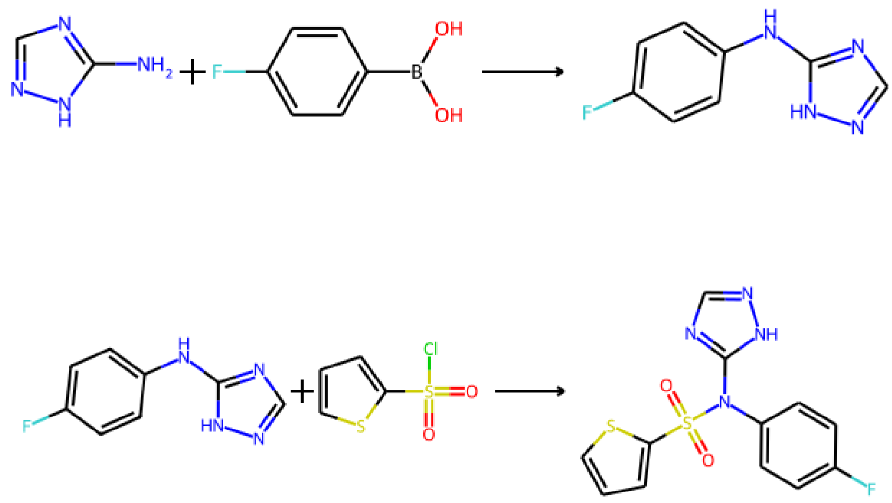

Product 68

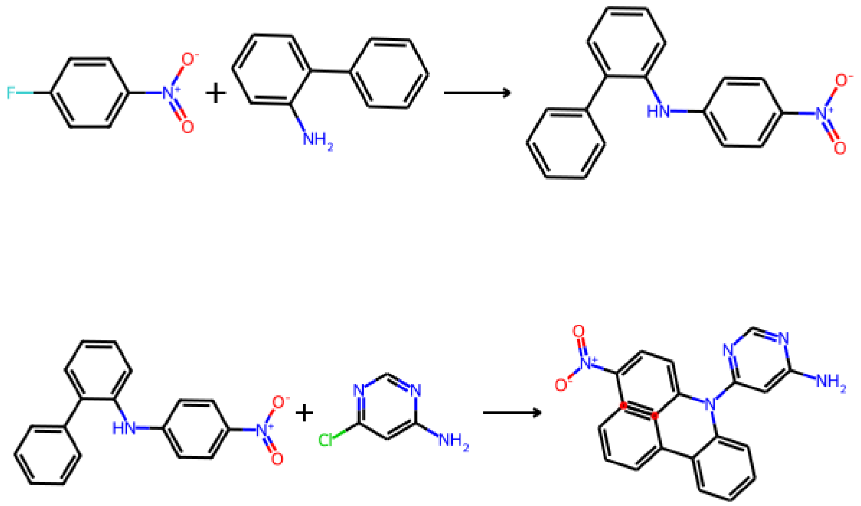

Product 69

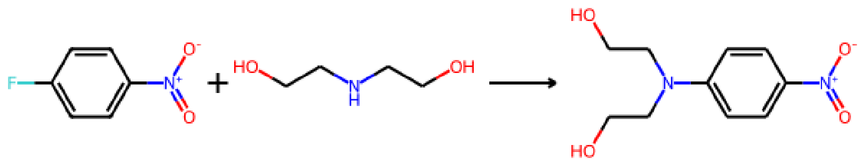

Product 70

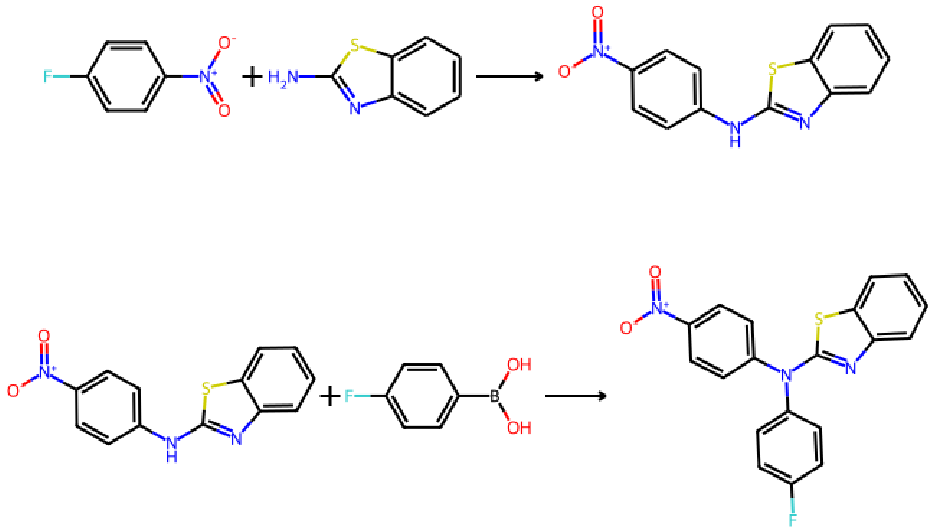

Product 71

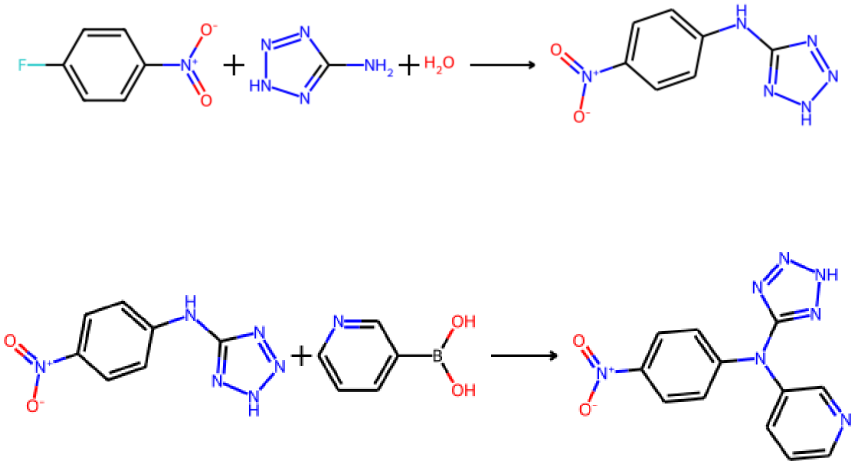

Product 72

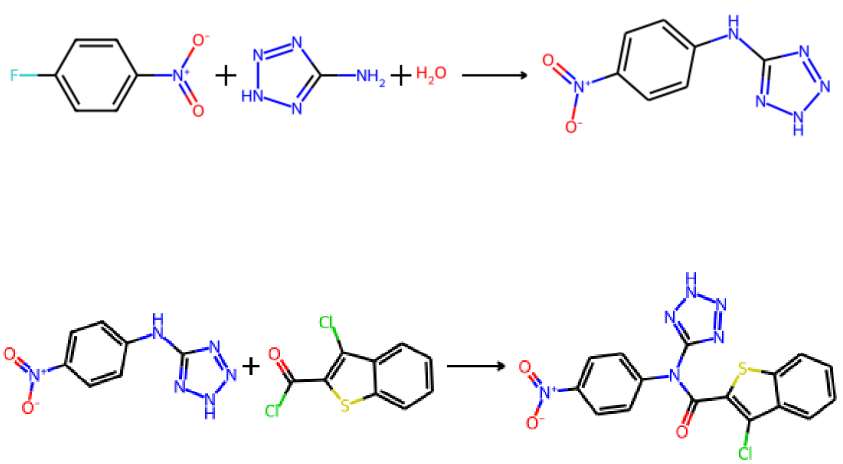

Product 73

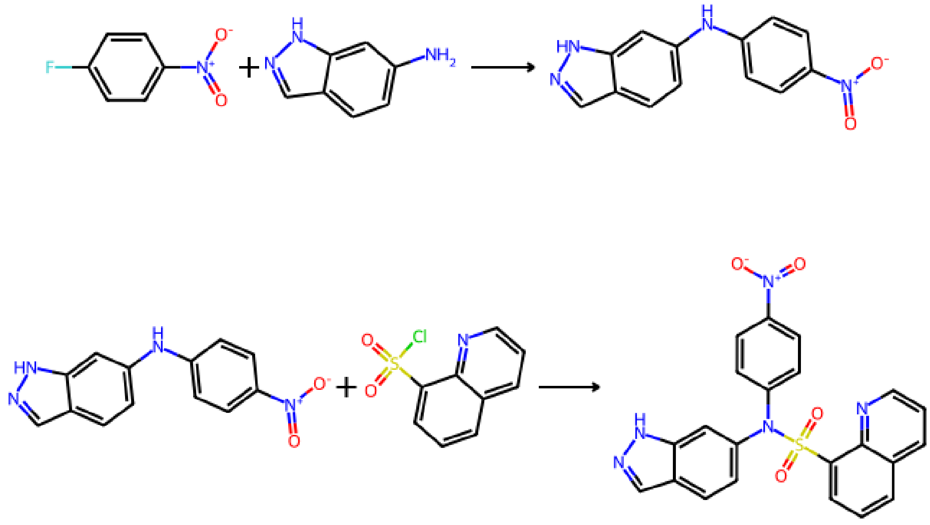

Product 74

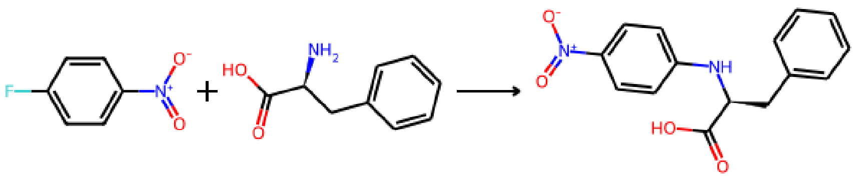

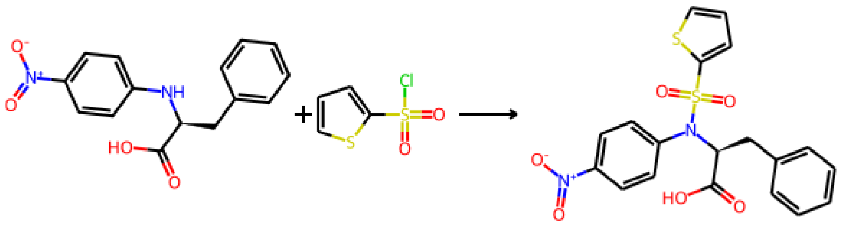

Product 75

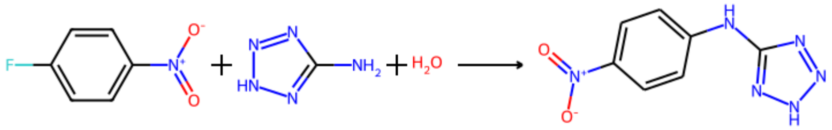

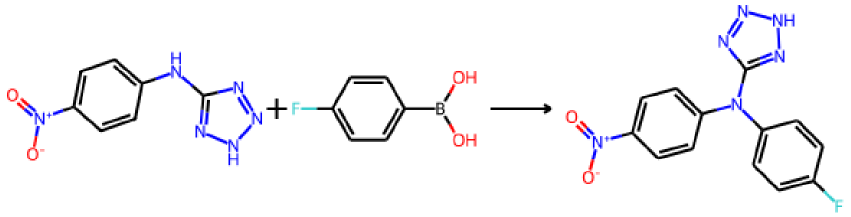

Product 76

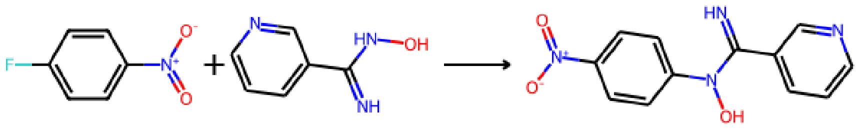

Product 77

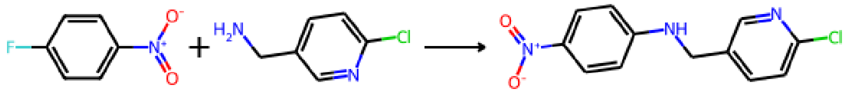

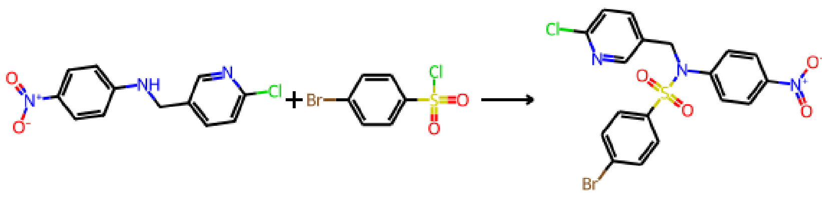

Product 78

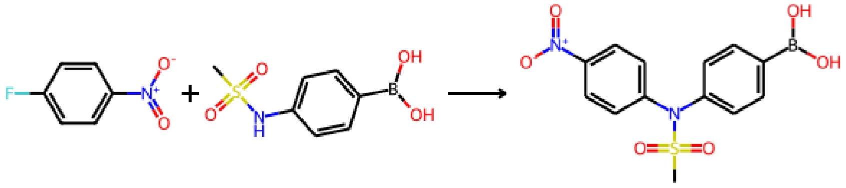

Product 79

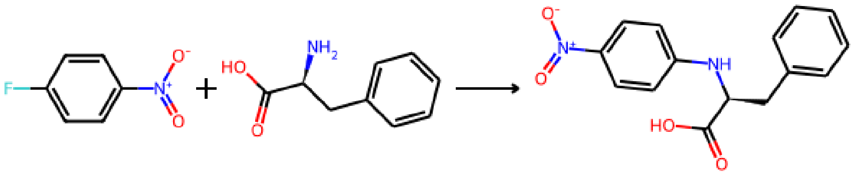

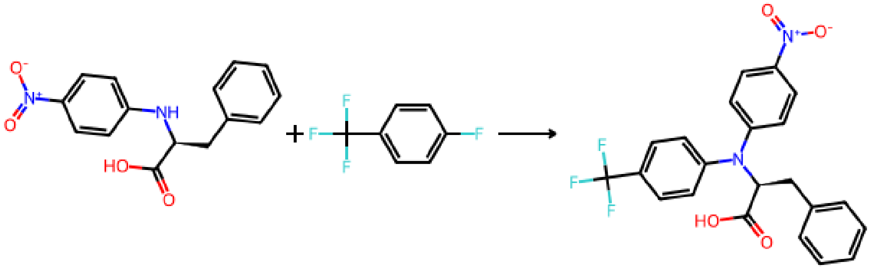

Product 80

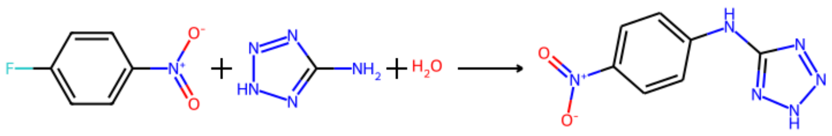

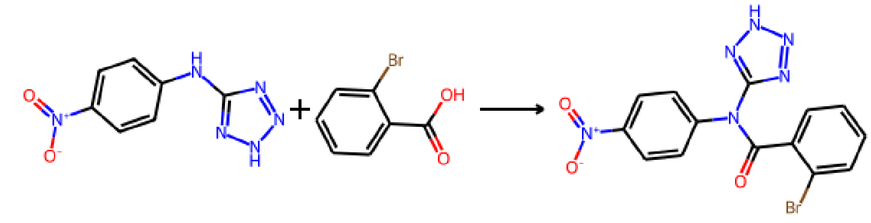

Product 81

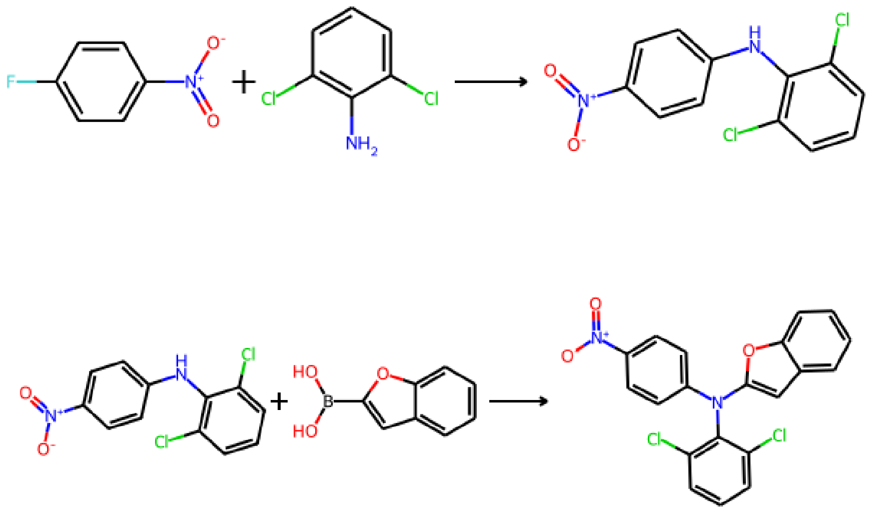

Product 82

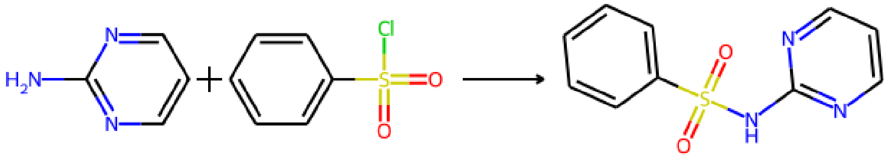

Product 83

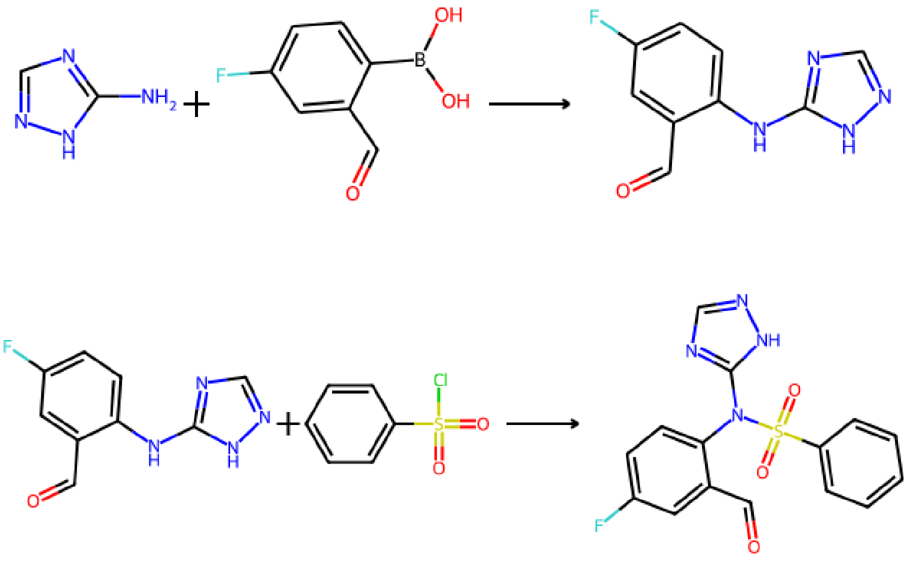

Product 84

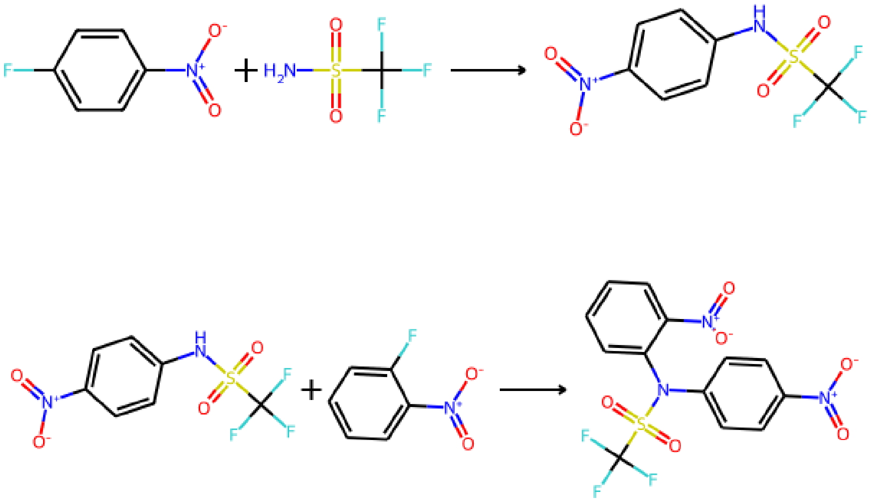

Product 85

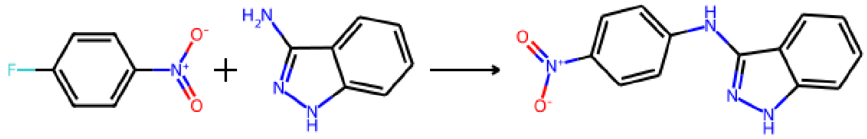

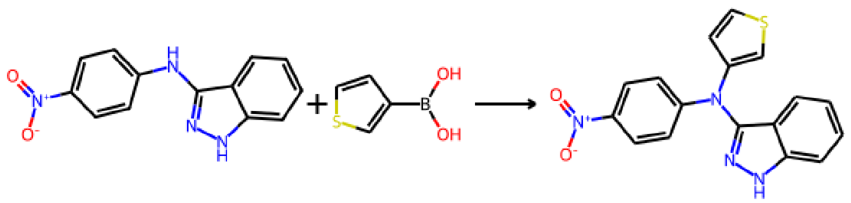

Product 86

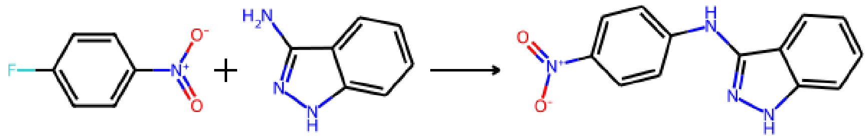

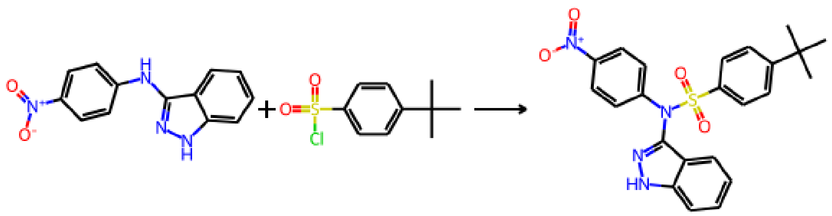

Product 87

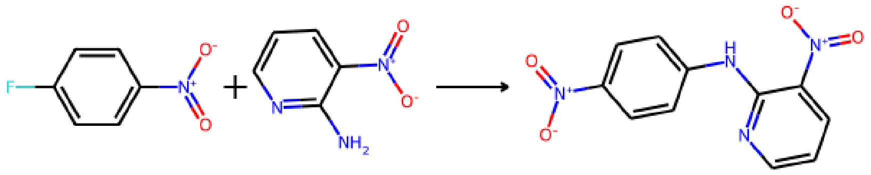

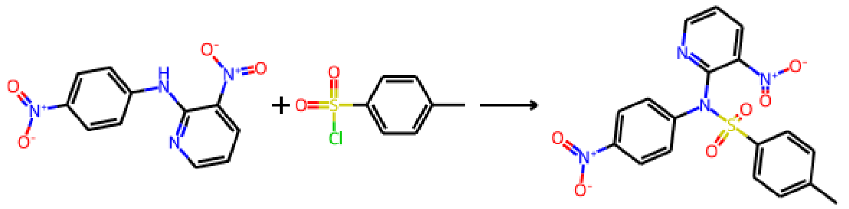

Product 88

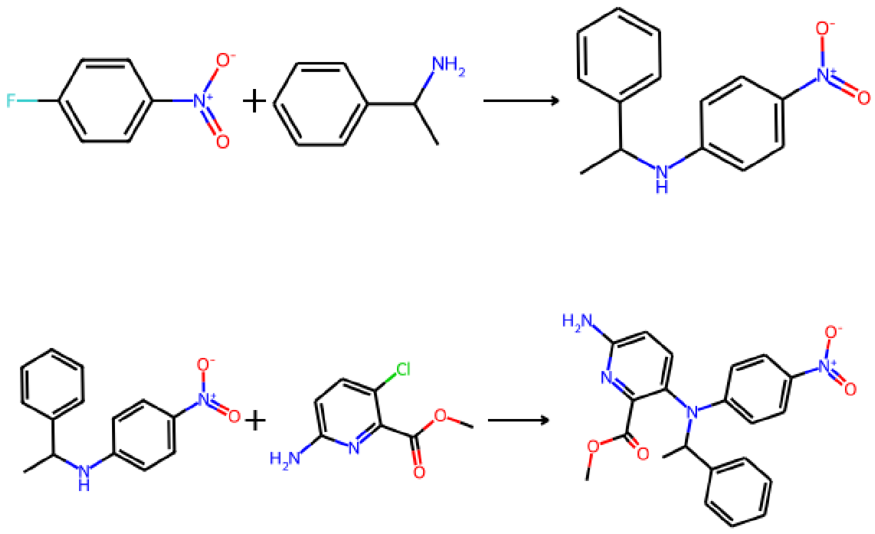

Product 89

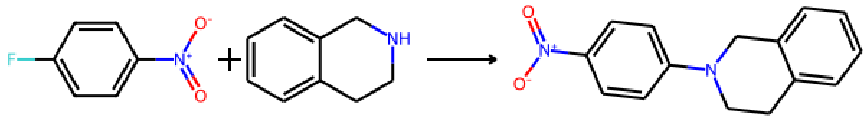

Product 90

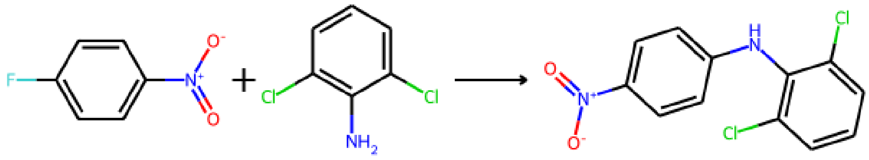

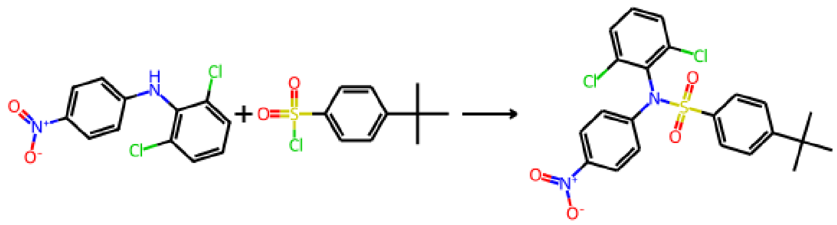

Product 91

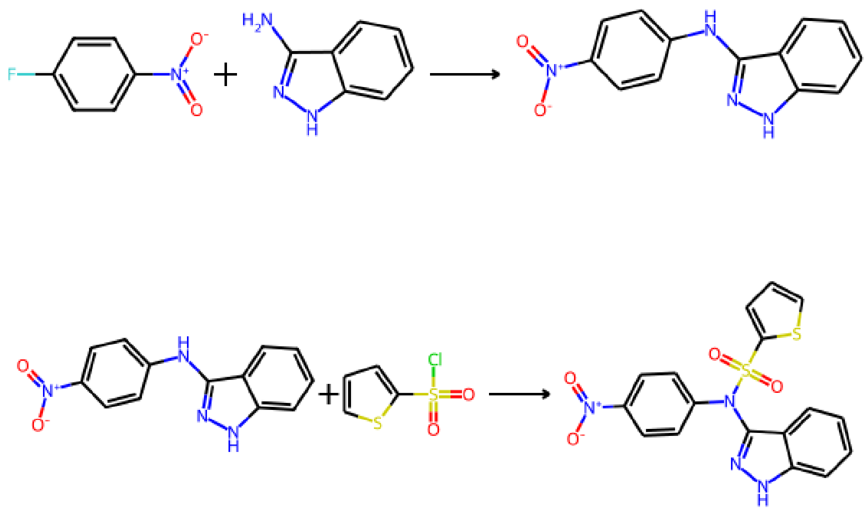

Product 92

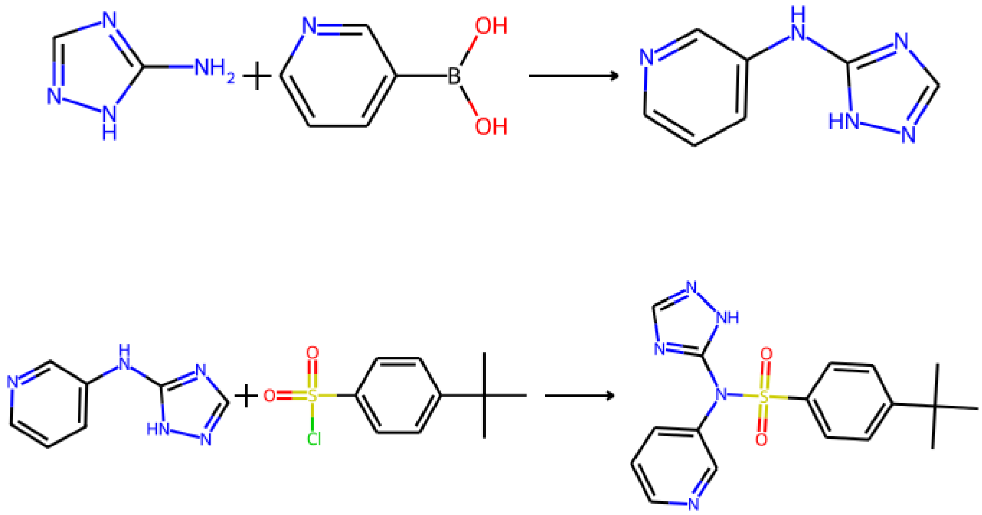

Product 93

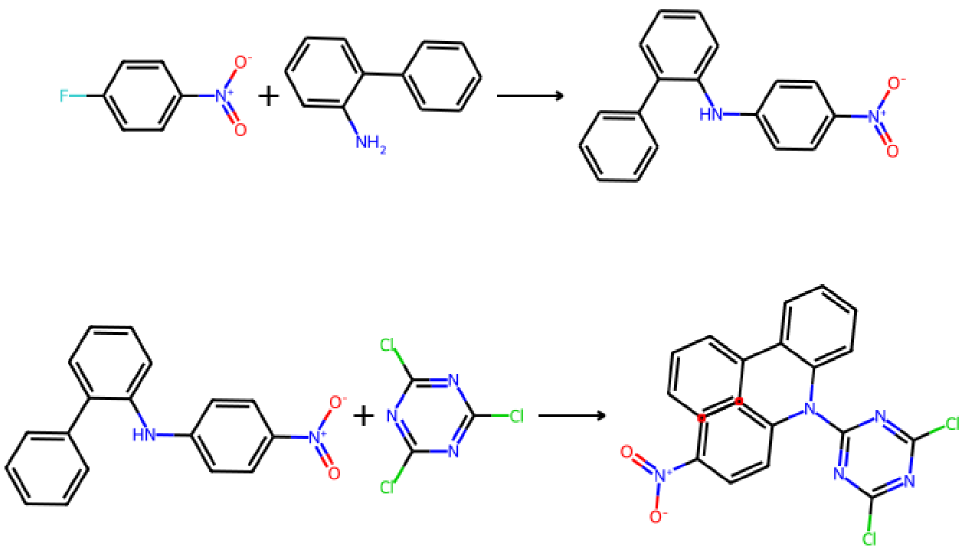

Product 94

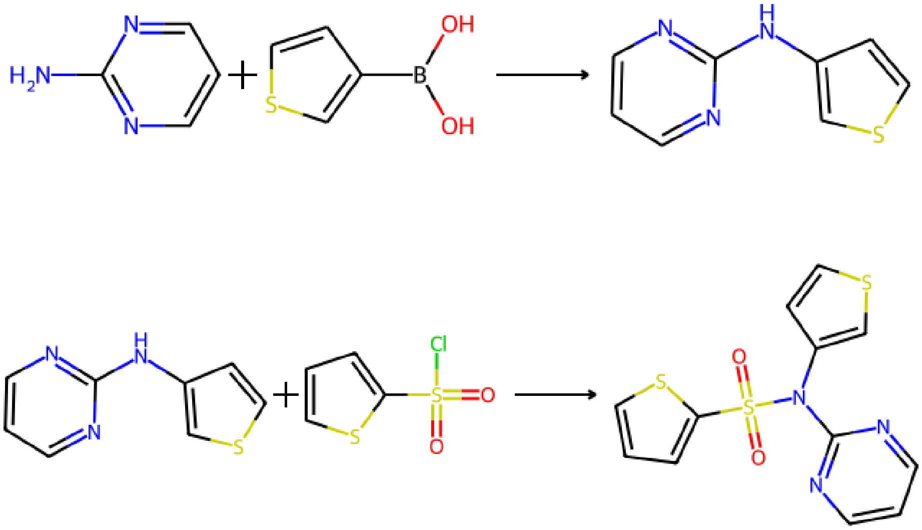

Product 95

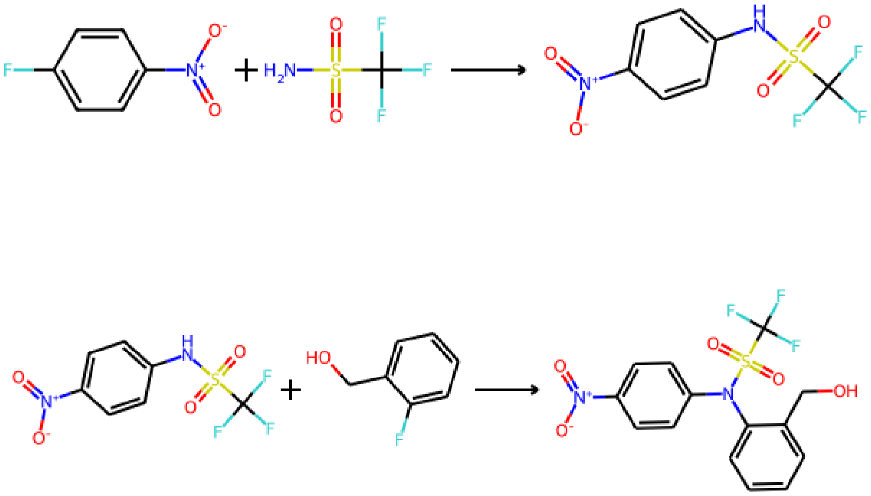

Product 96

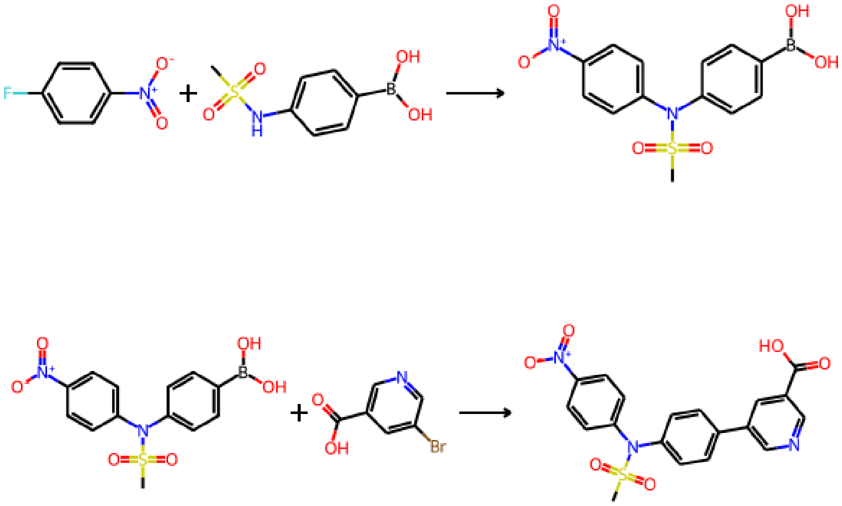

Product 97

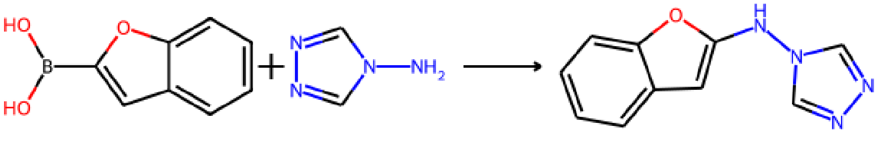

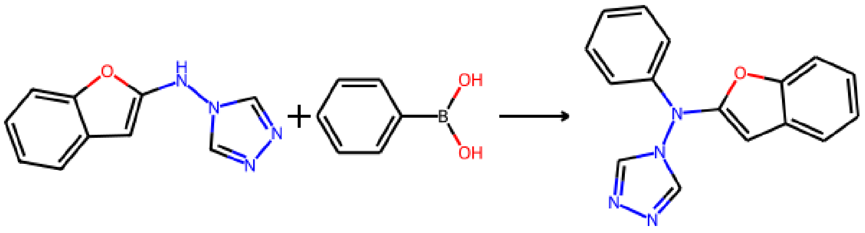

Product 98

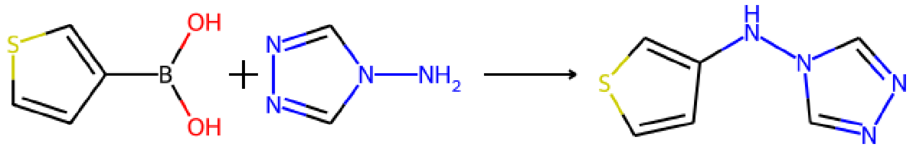

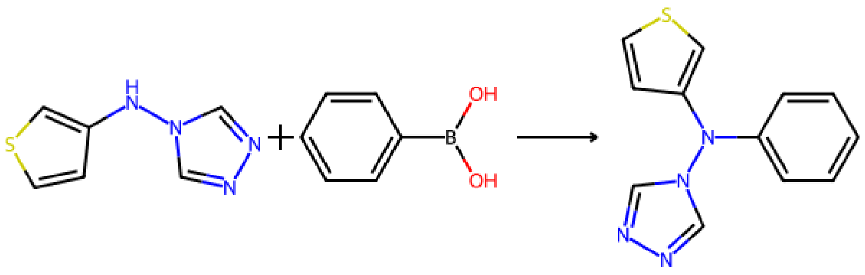

Product 99

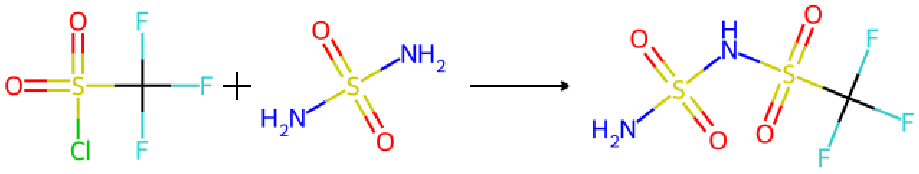

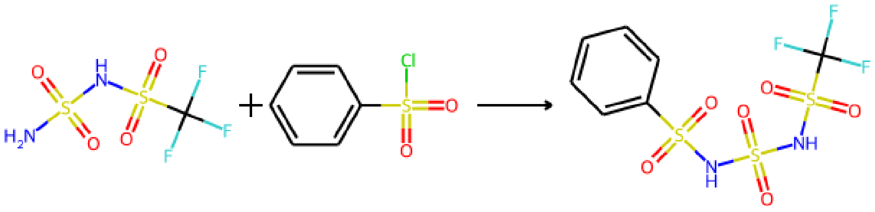

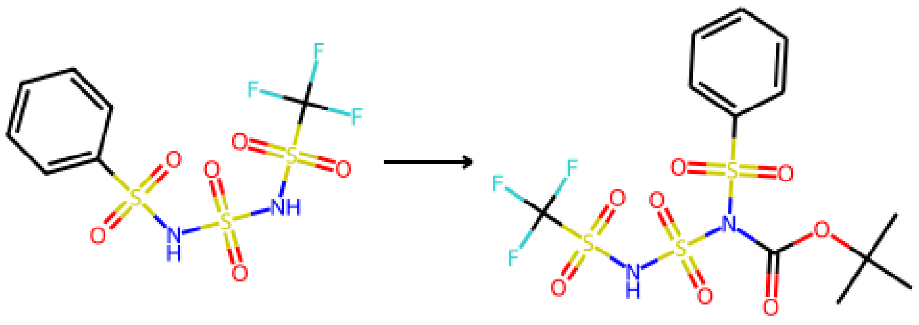

Product 100

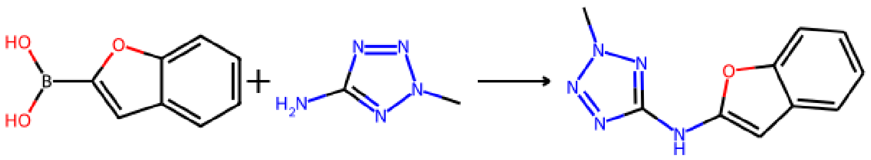

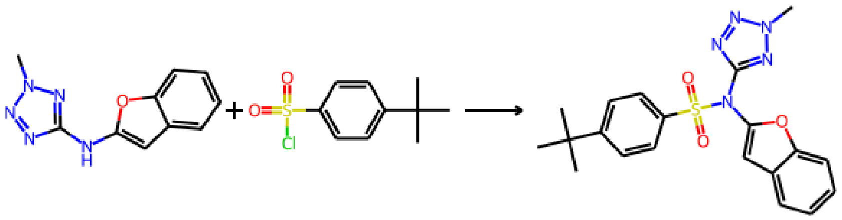

Product 101

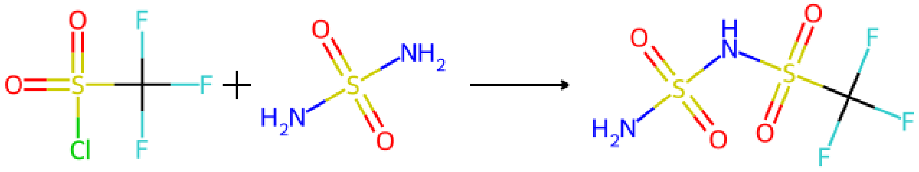

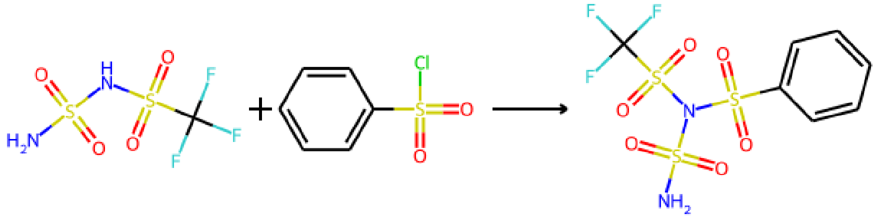

Product 102

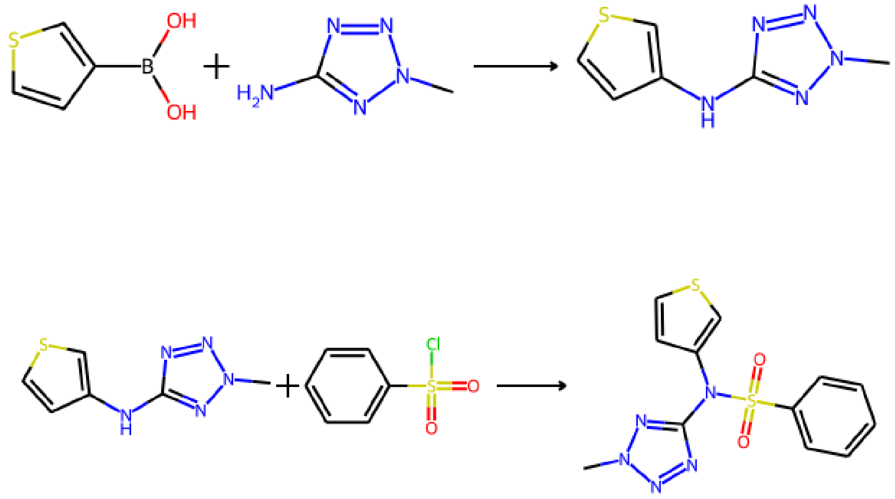

Product 103

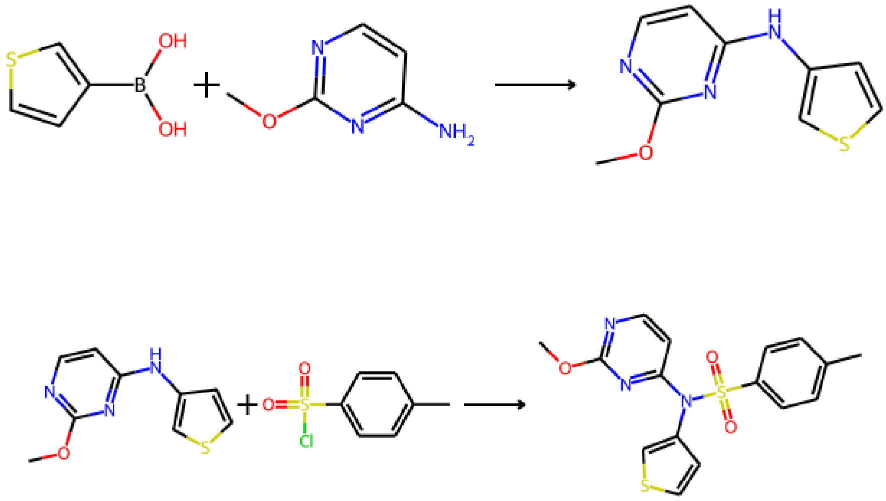

Product 104

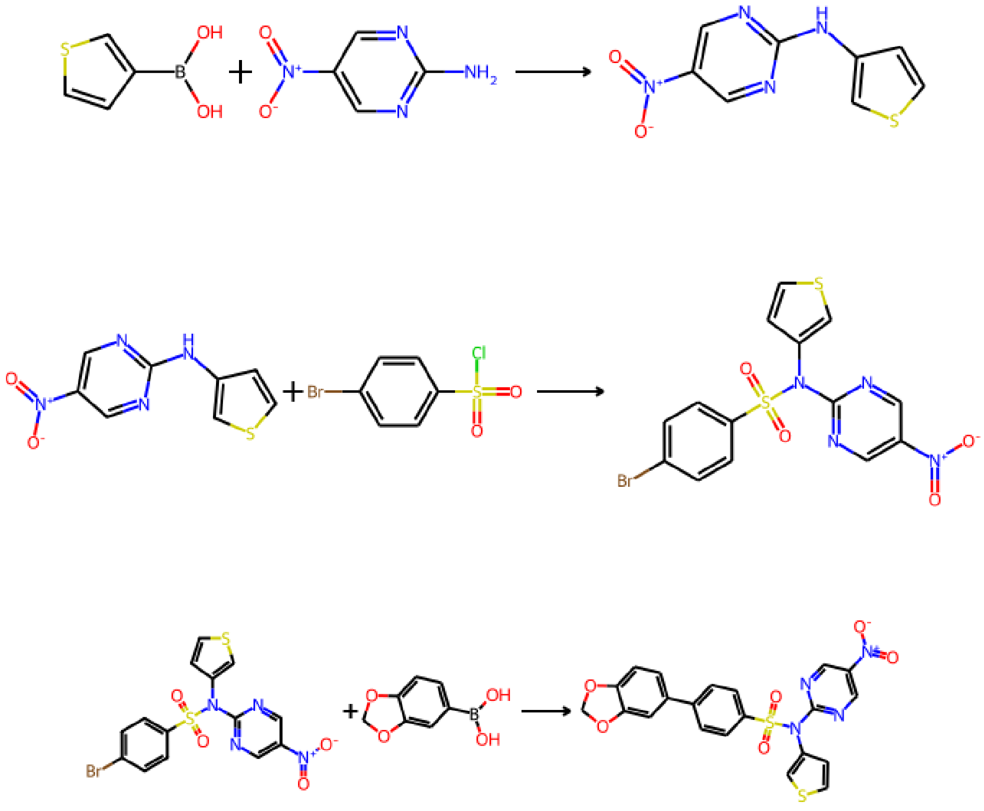

Product 105

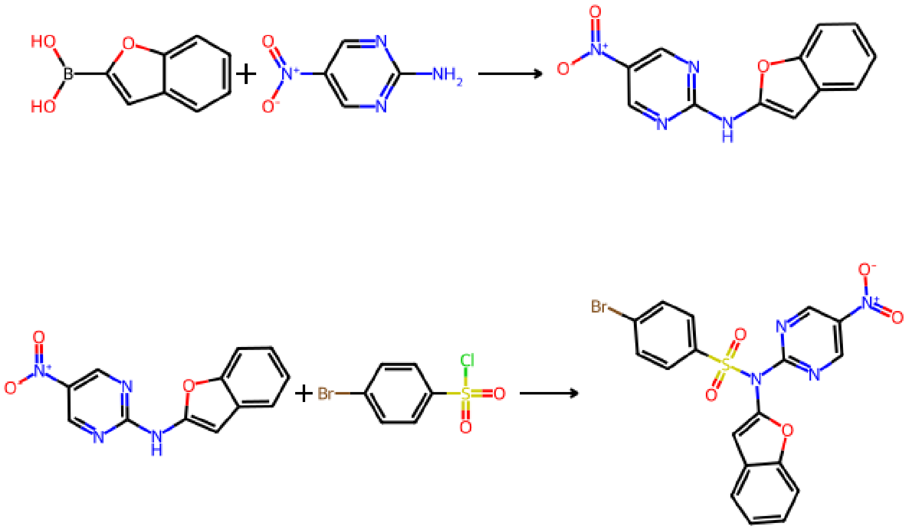

Product 106

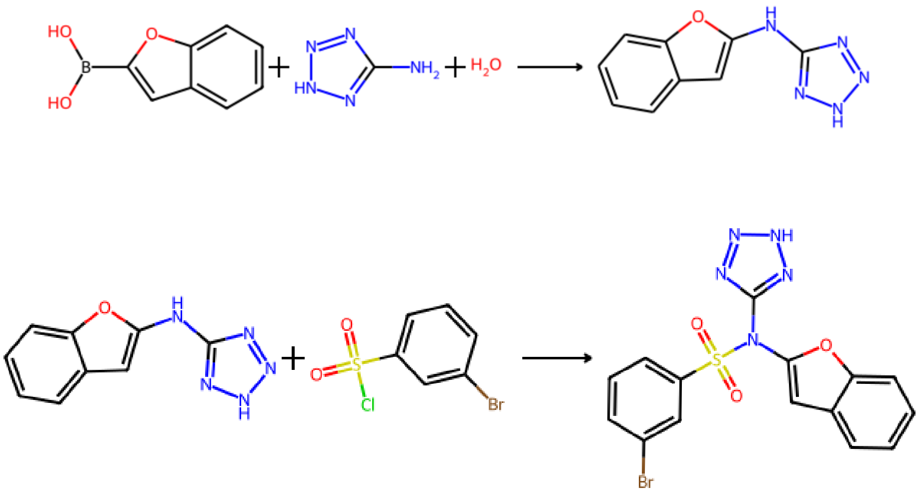

Product 107

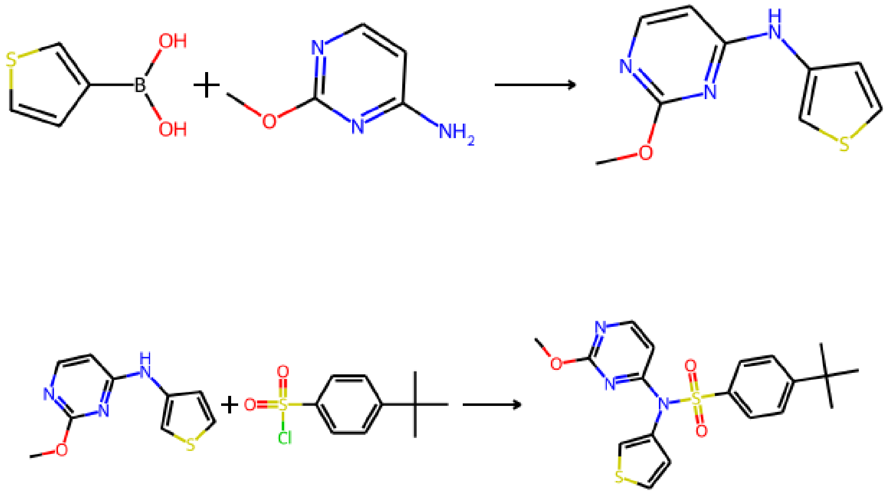

Product 108

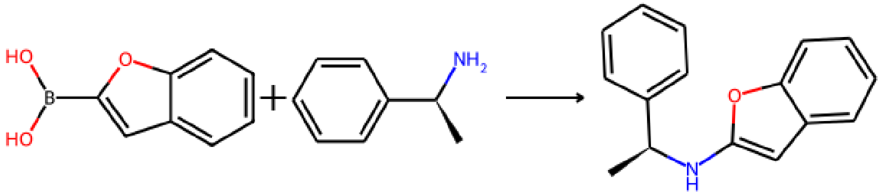

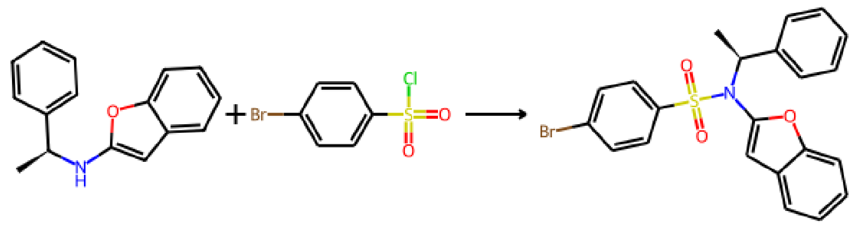

Product 109

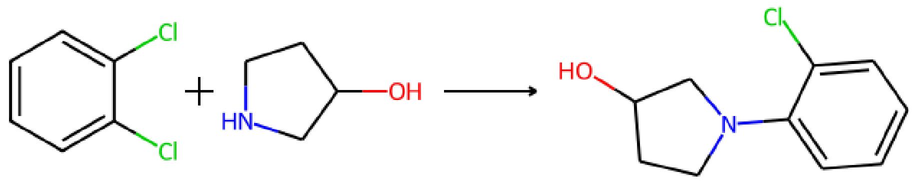

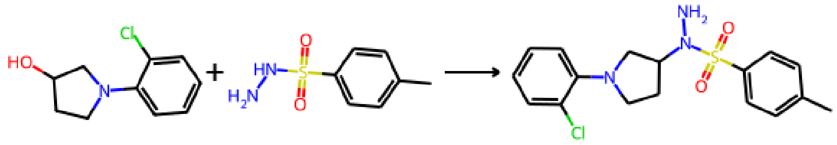

Product 110

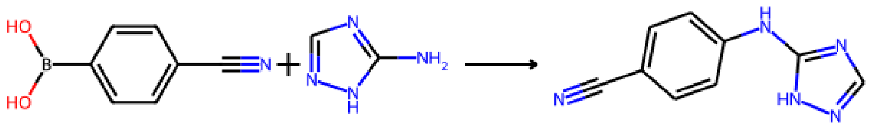

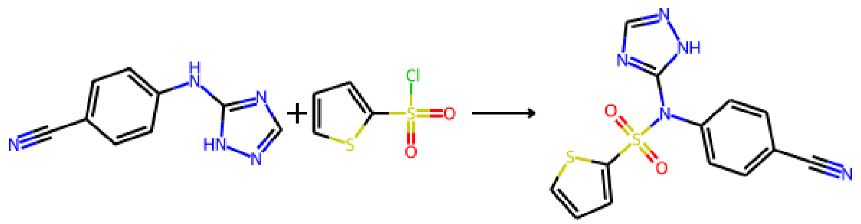

Product 111

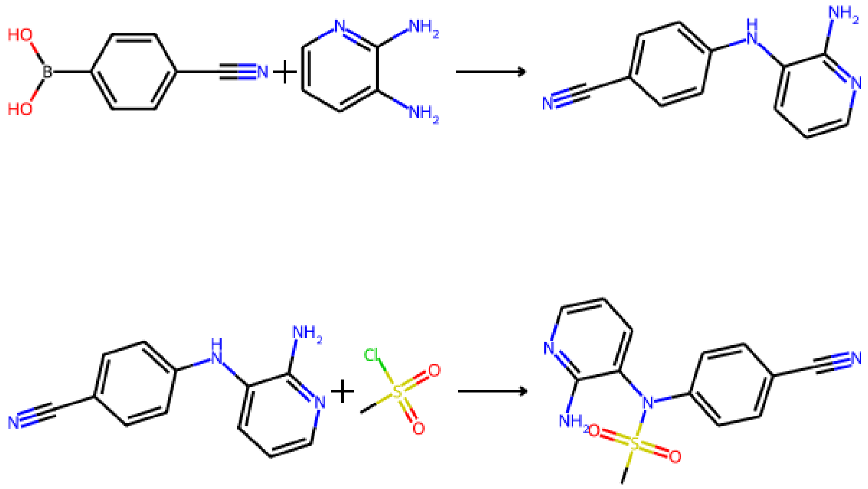

Product 112

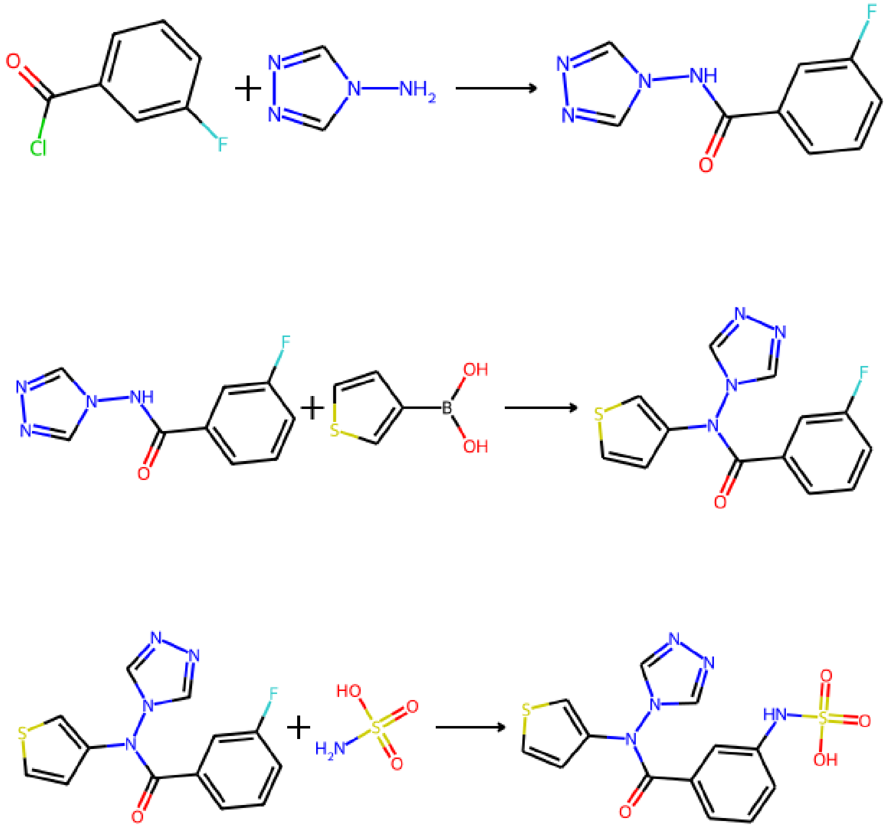

Product 113

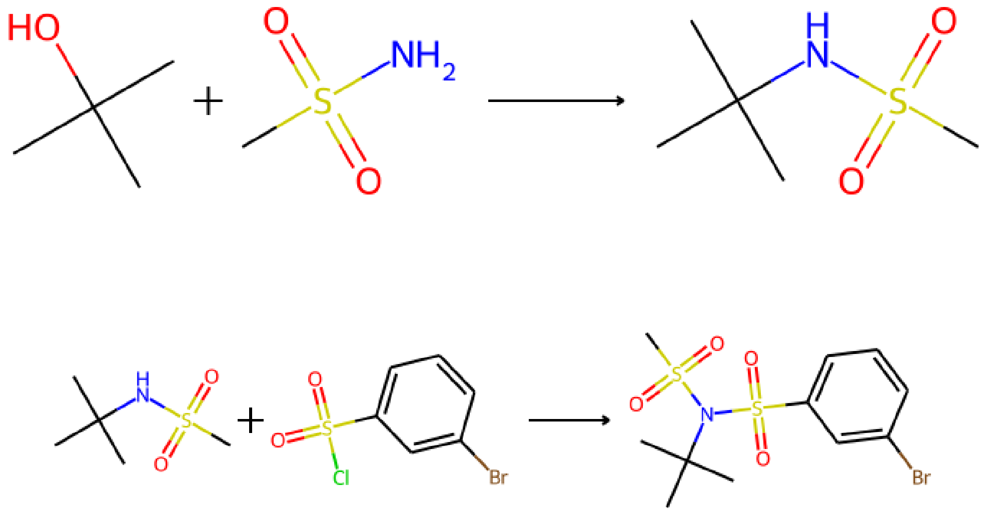

Product 114

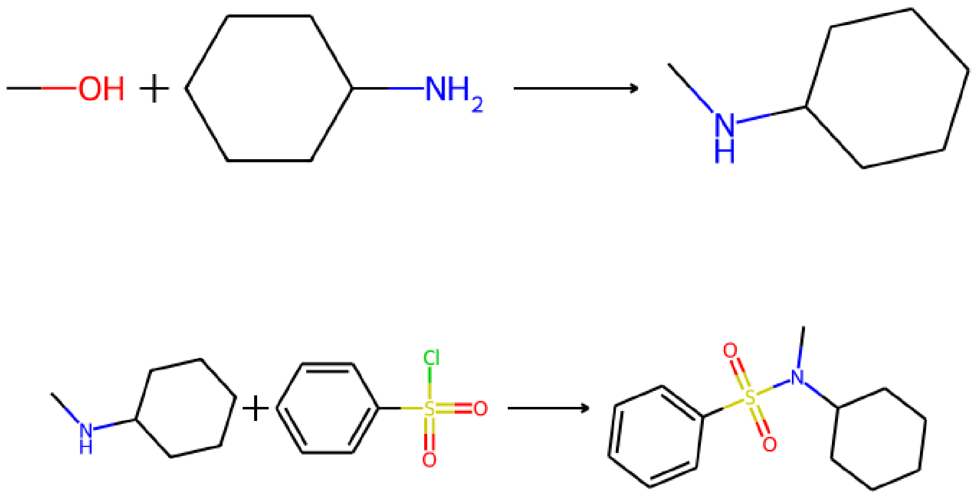

Product 115

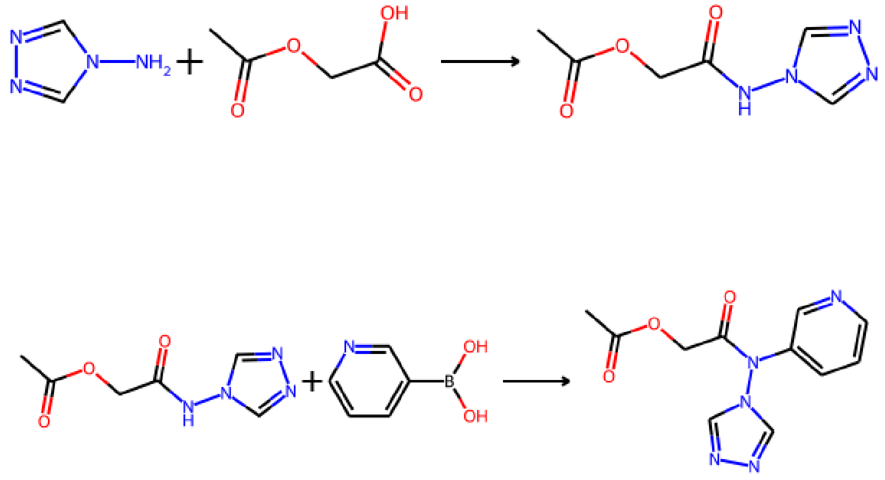

Product 116

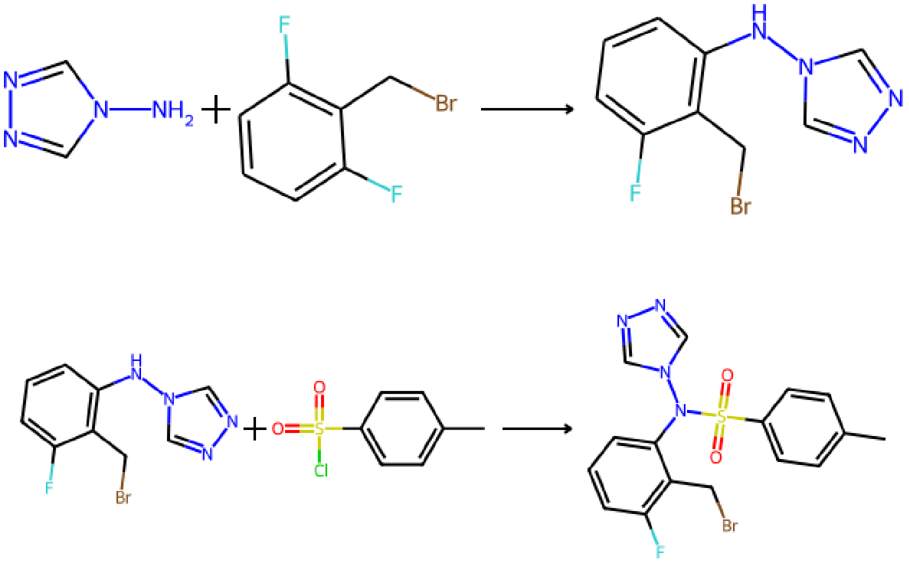

Product 117

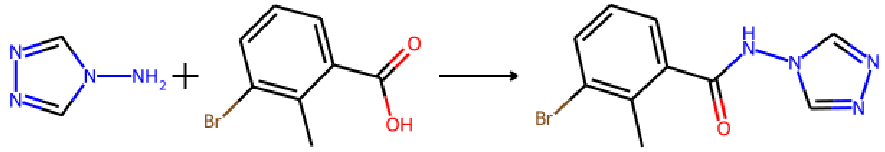

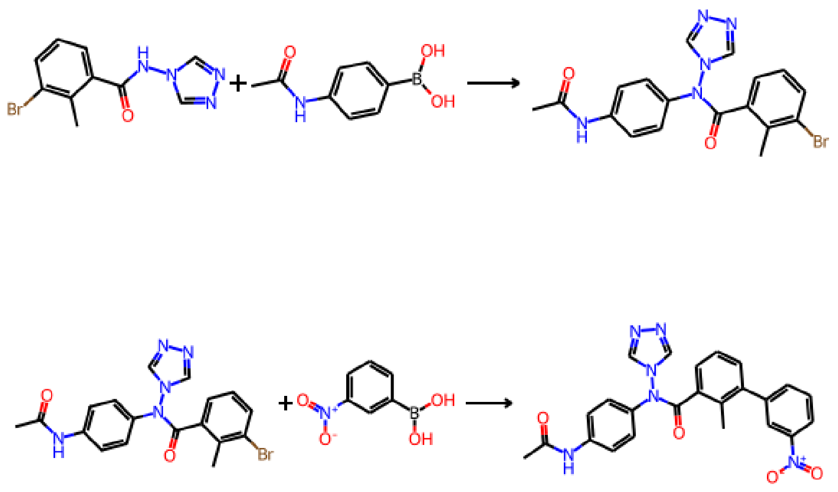

Product 118

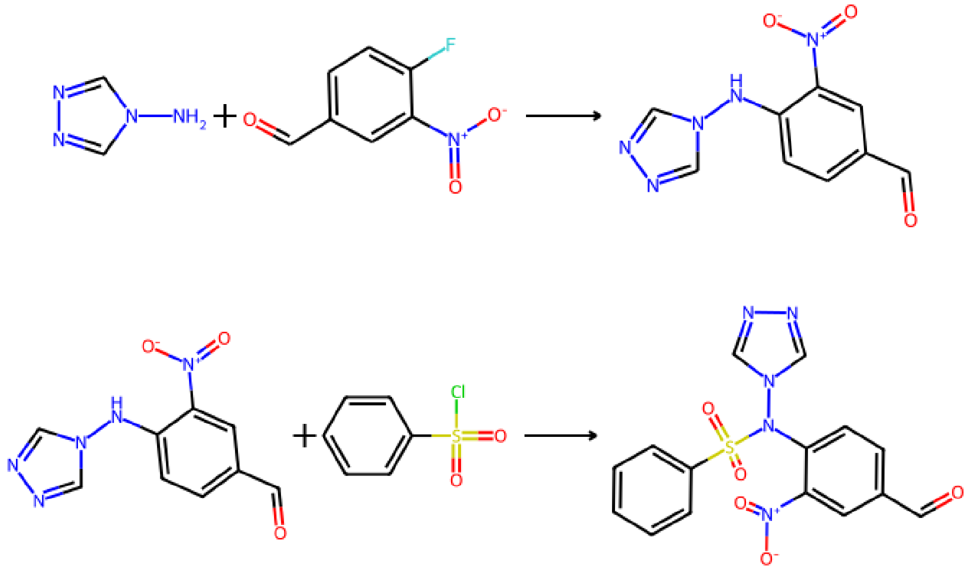

Product 119

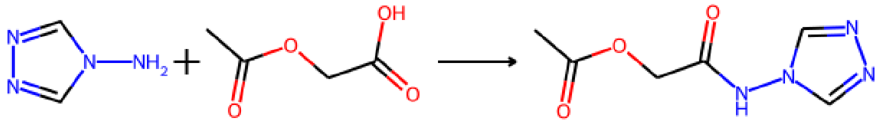

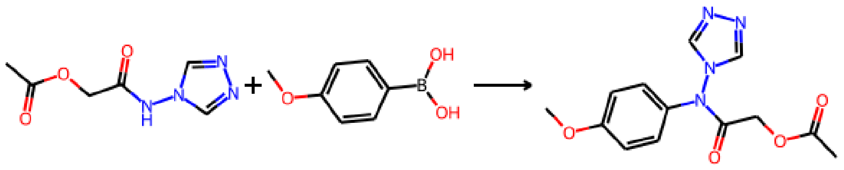

Product 120

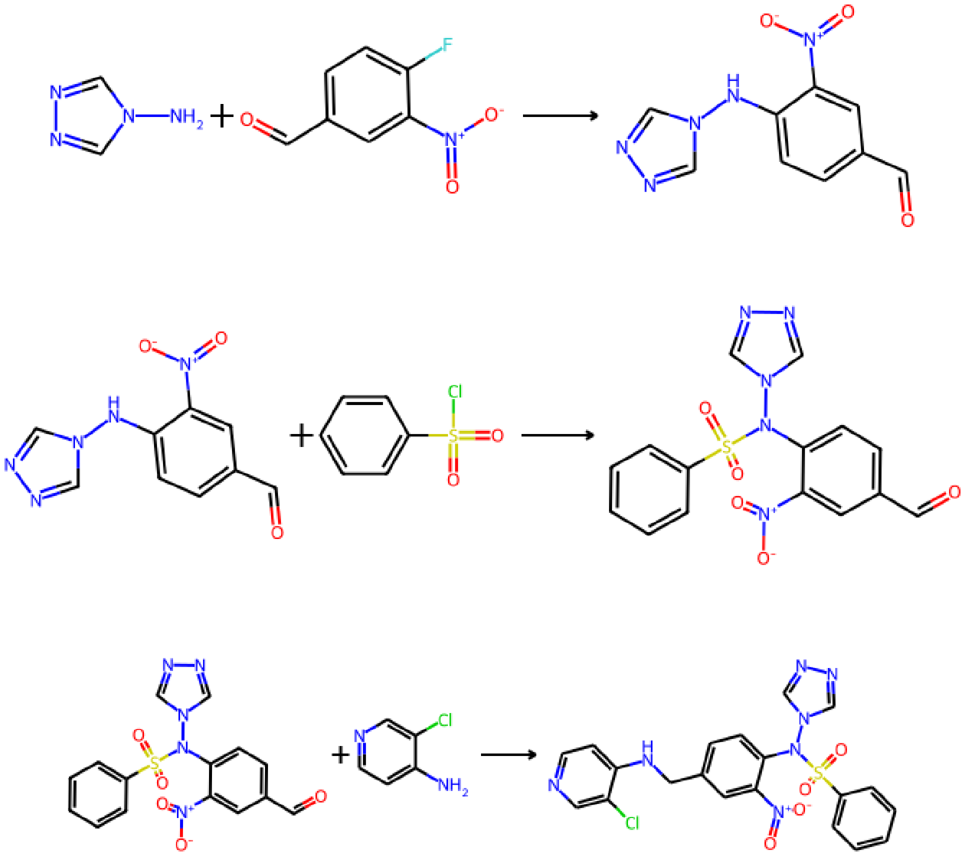

Product 121

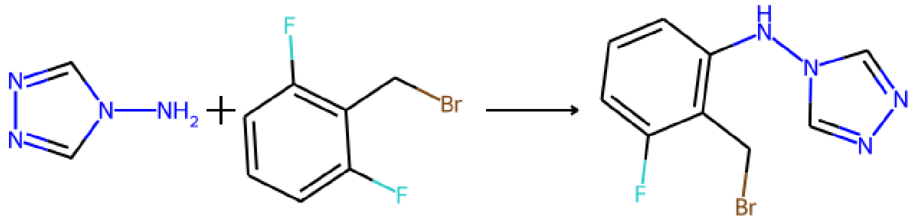

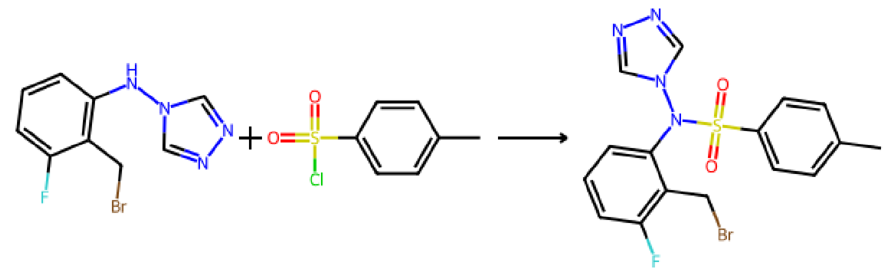

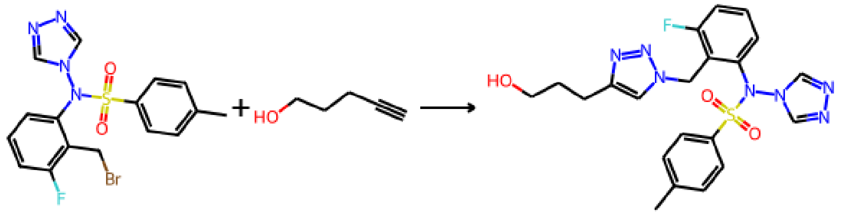

Product 122

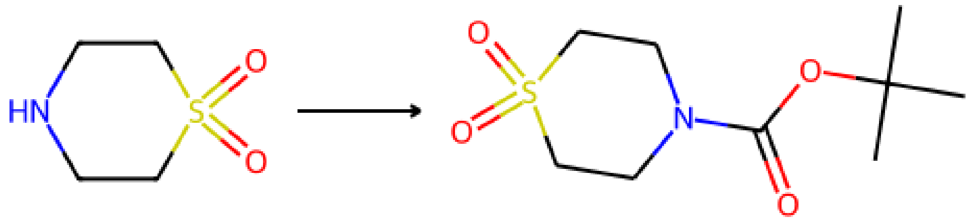

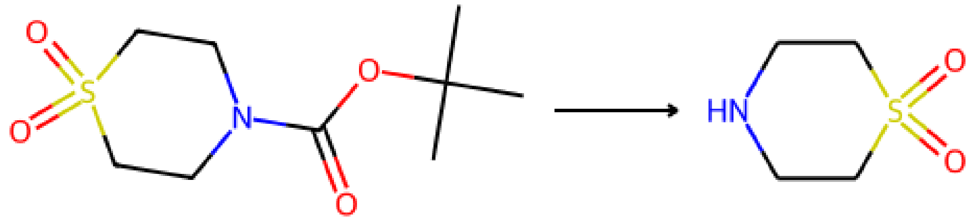

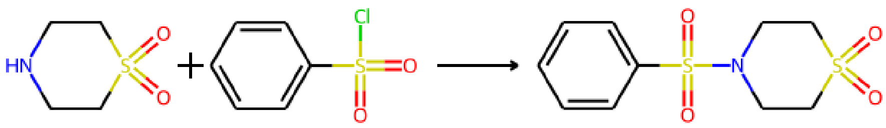

Product 123

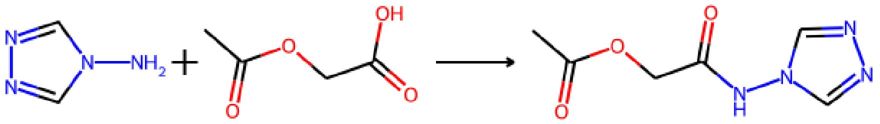

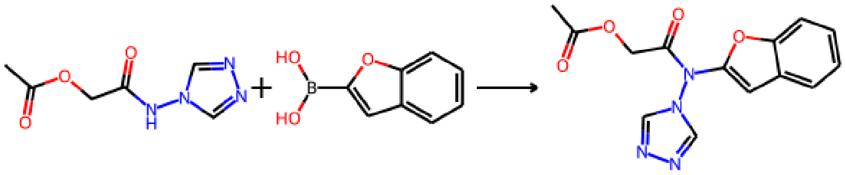

Product 124

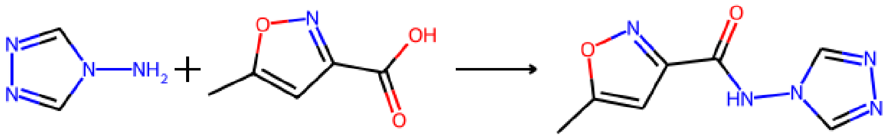

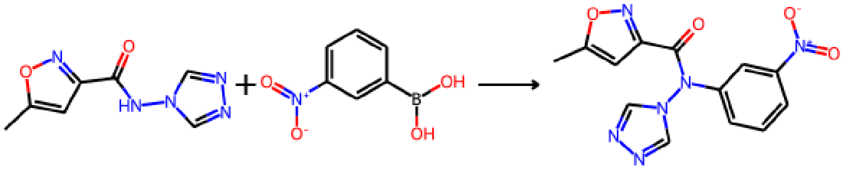

Product 125

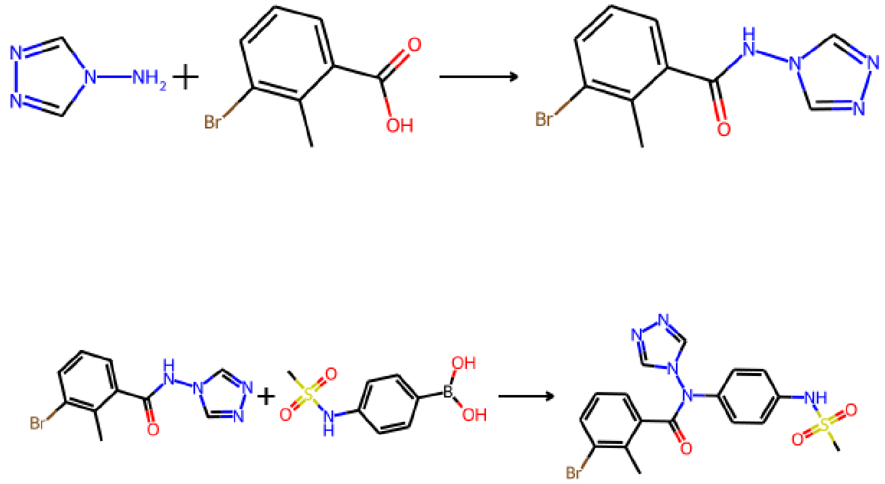

Product 126

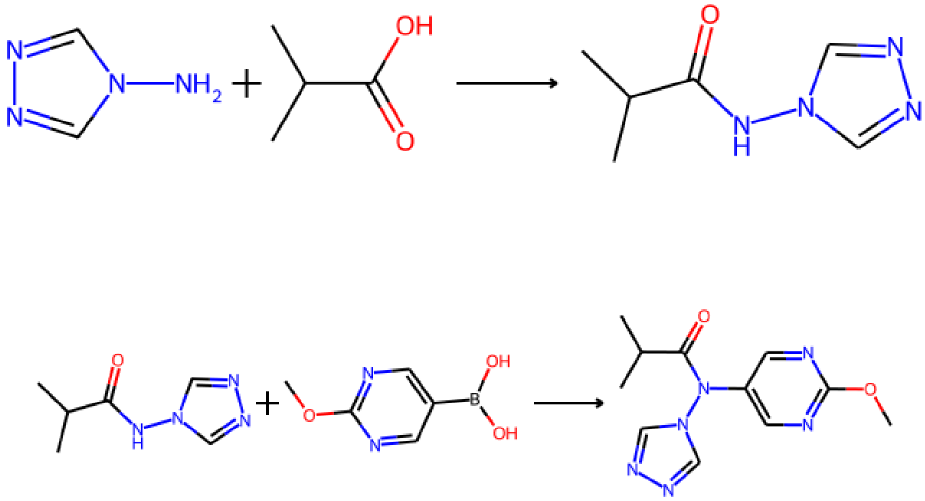

Product 127

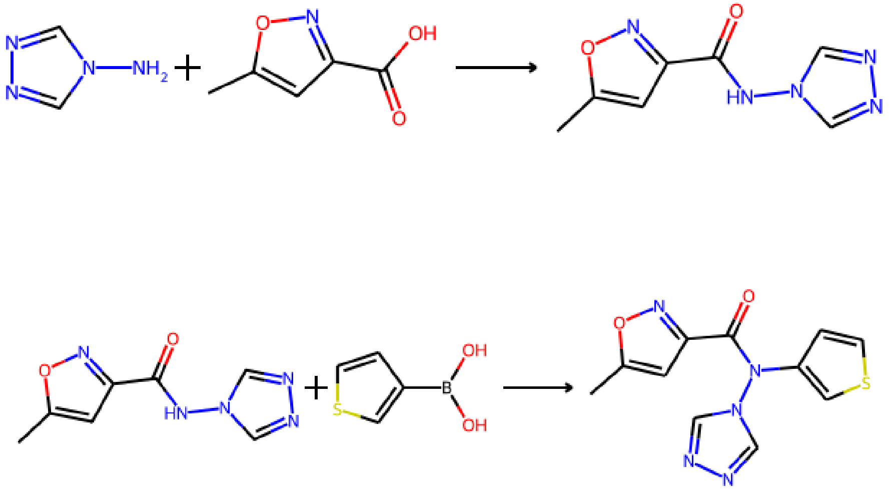

Product 128

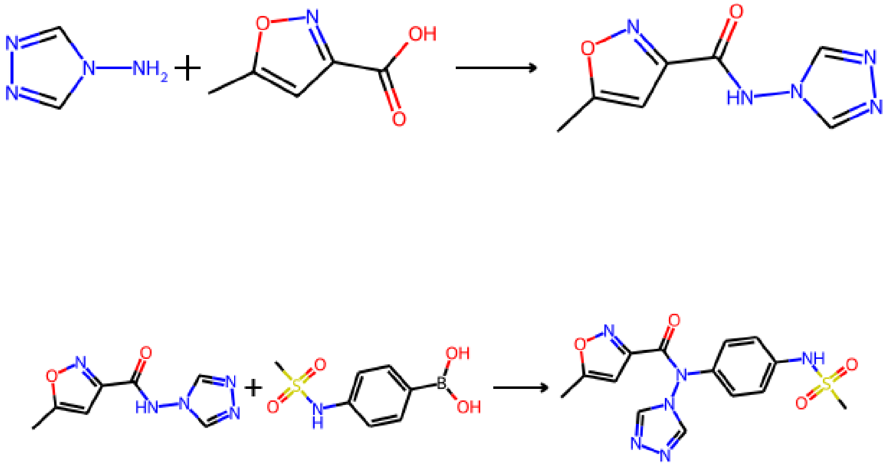

Product 129

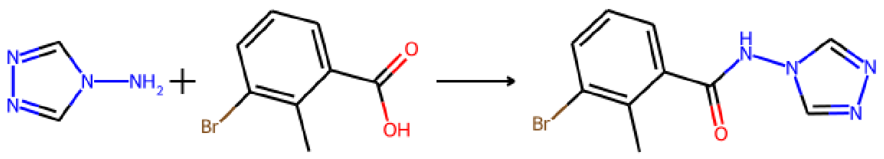

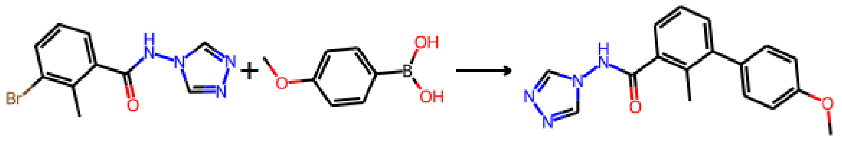

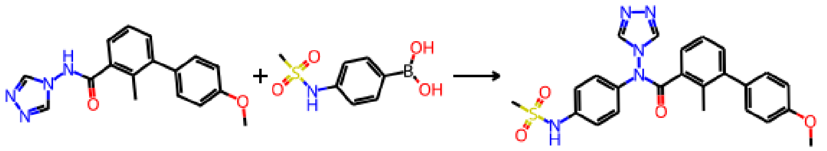

Product 130

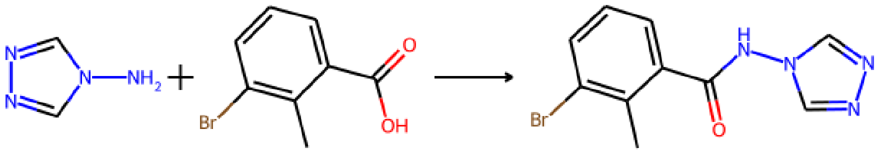

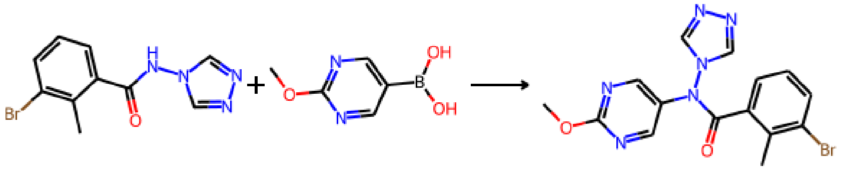

Product 131

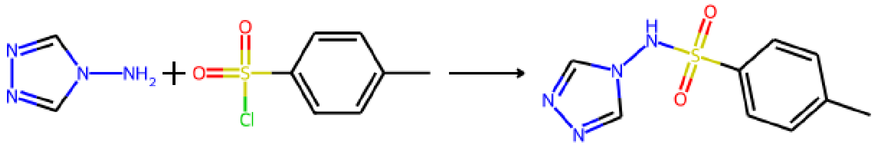

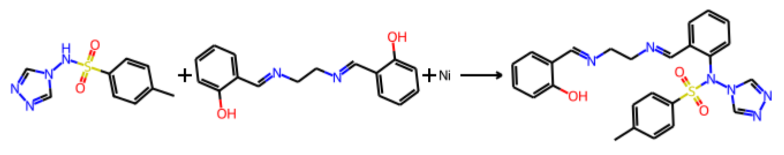

Product 132

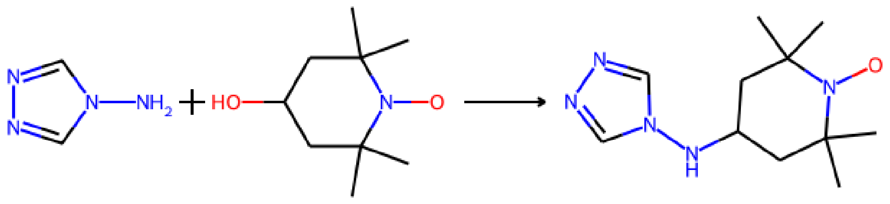

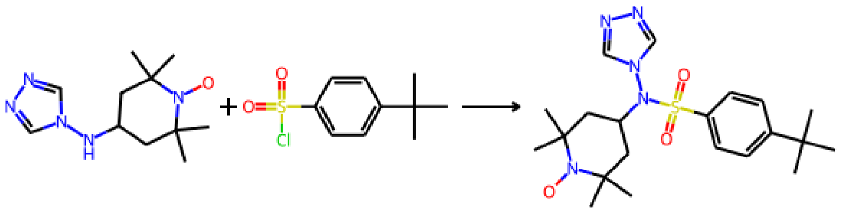

Product 133

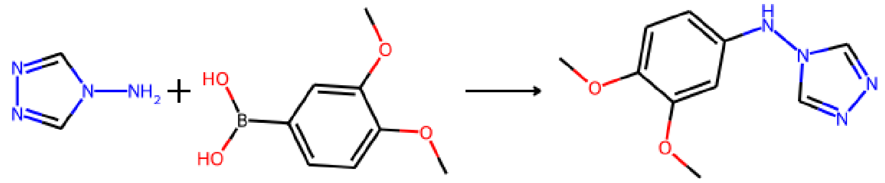

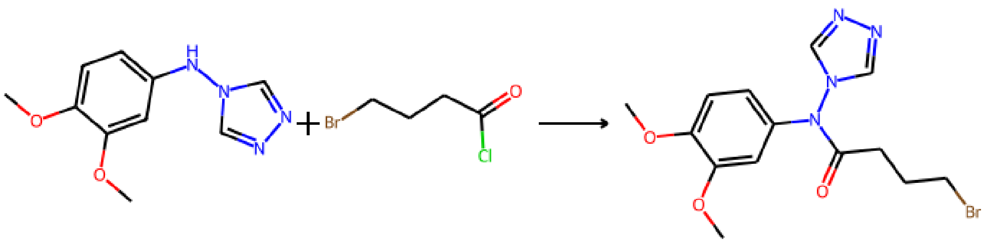

Product 134

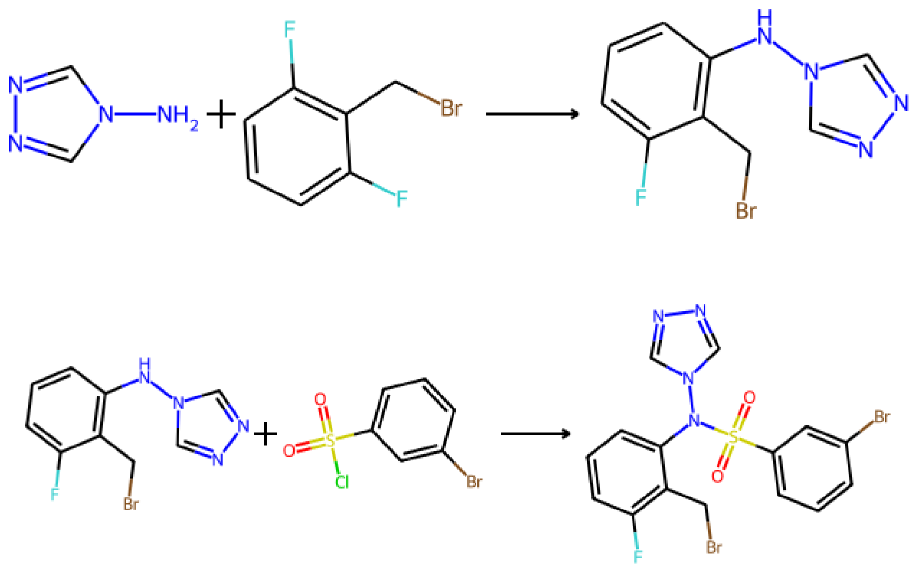

Product 135

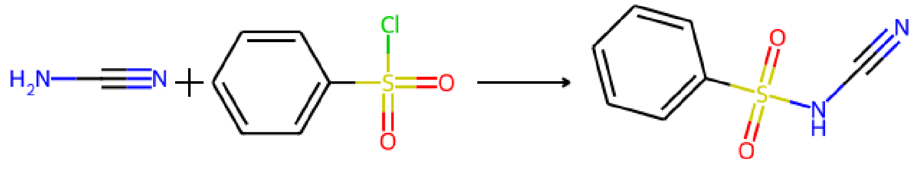

Product 136

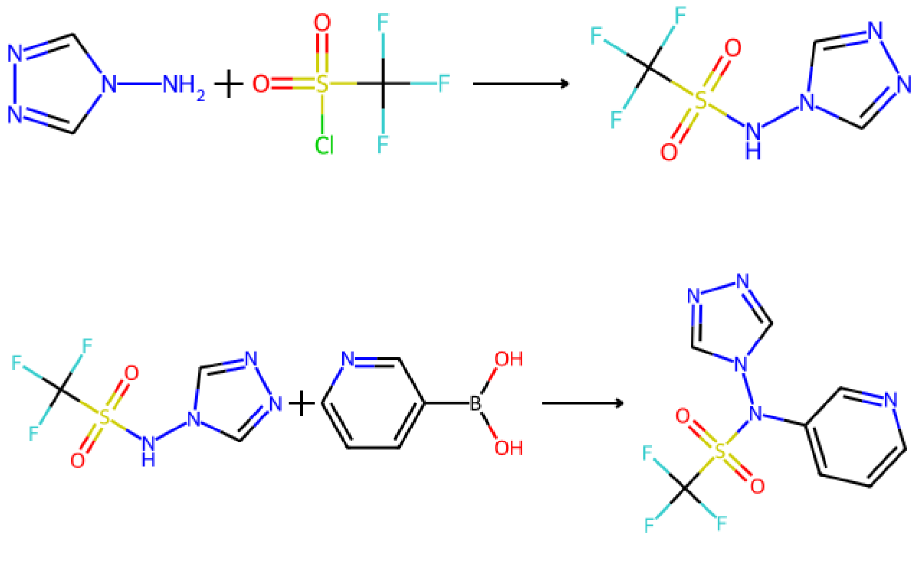

Product 137

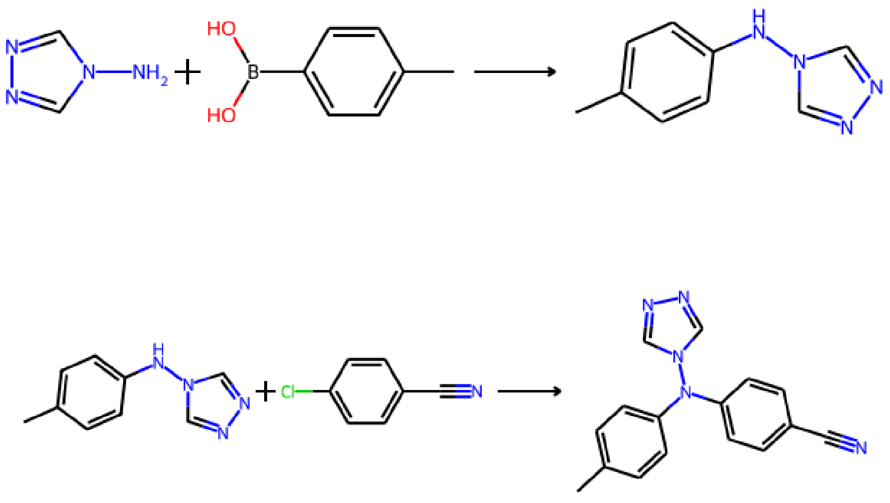

Product 138

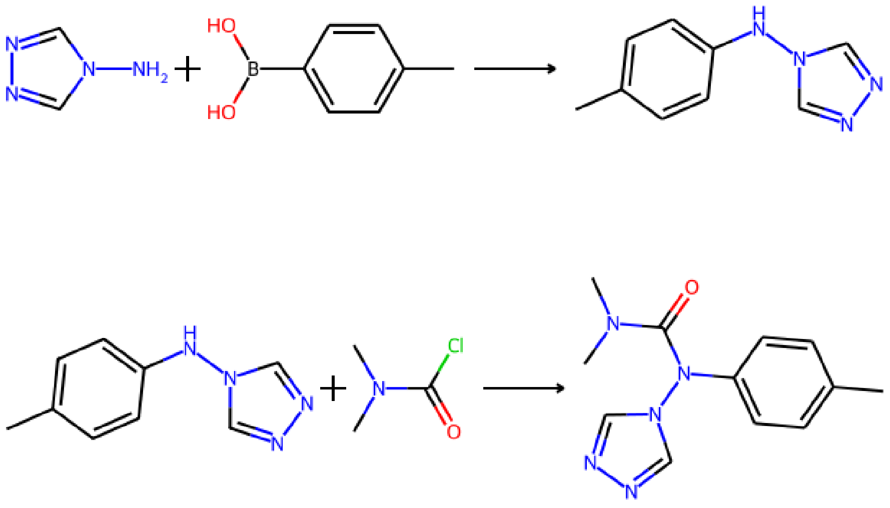

Product 139

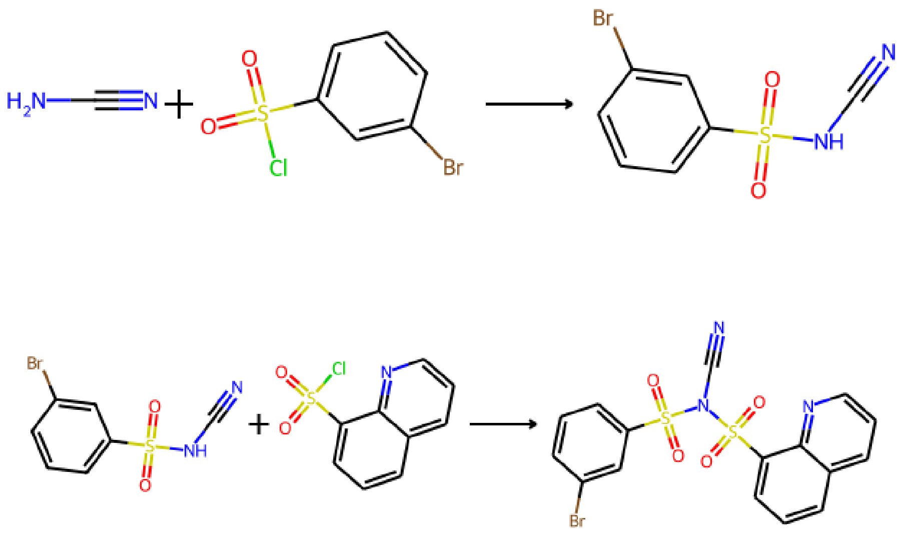

Product 140

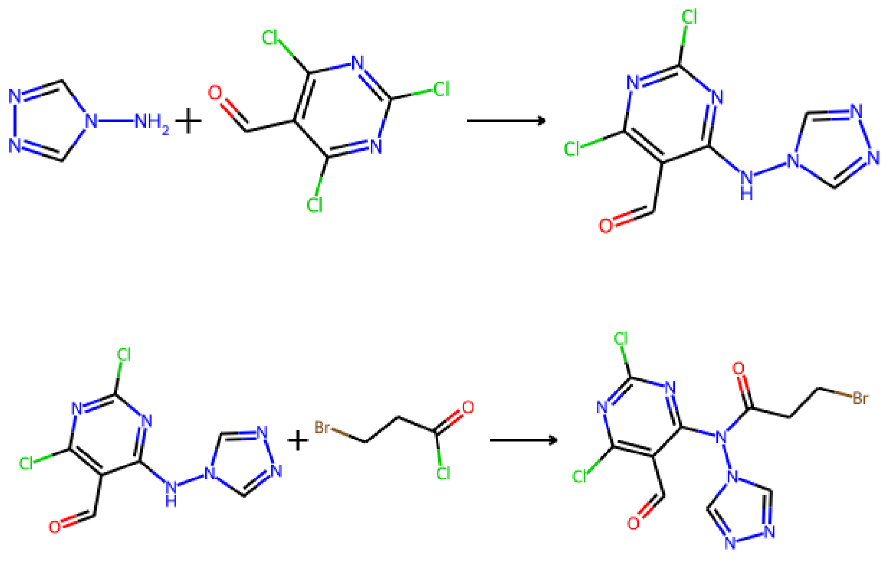

Product 141

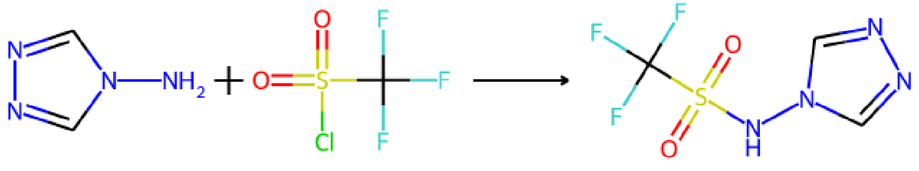

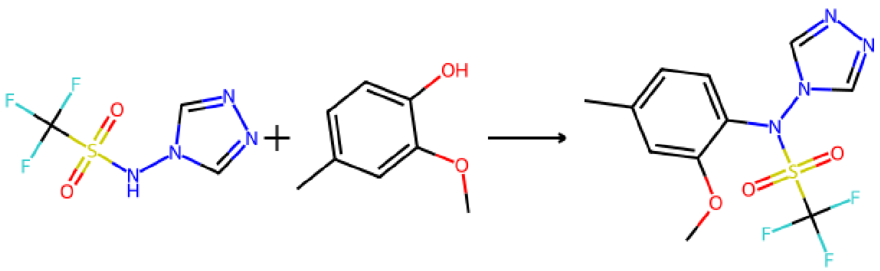

Product 142

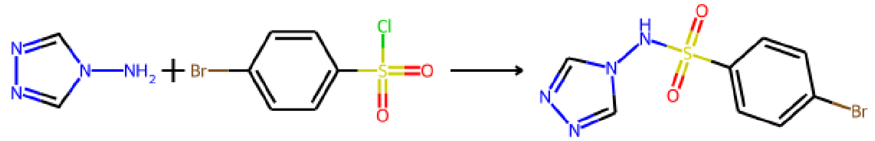

Product 143

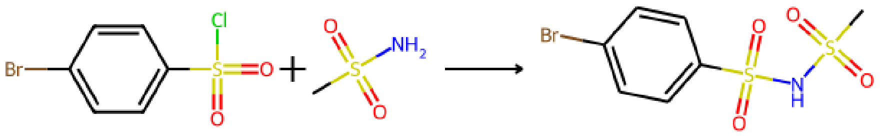

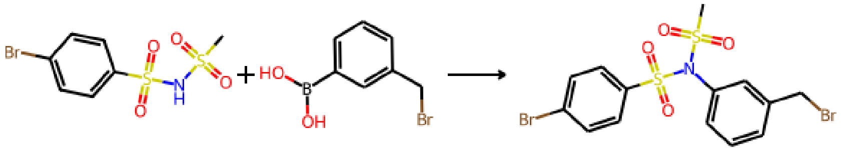

Product 144

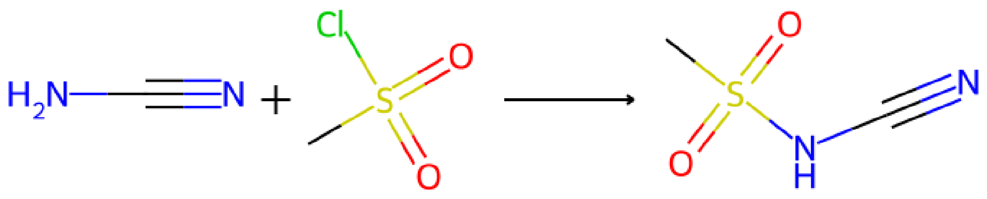

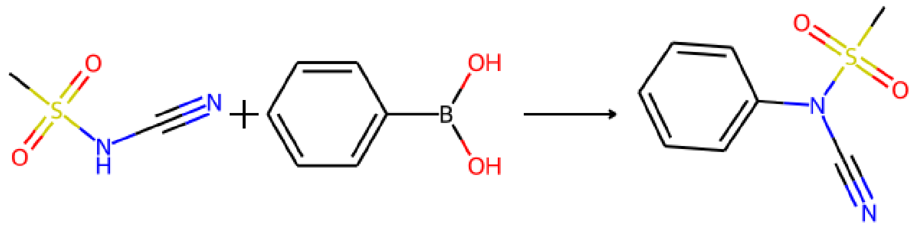

Product 145

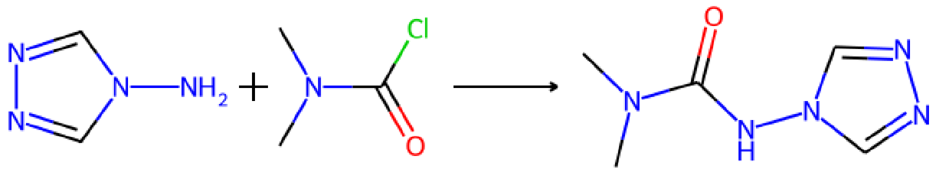

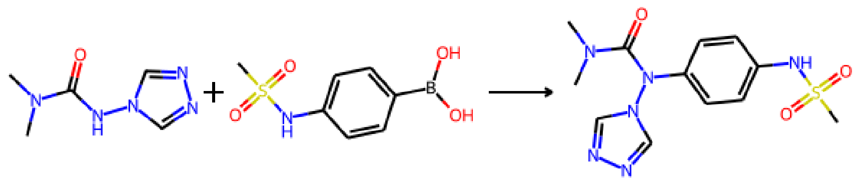

Product 146

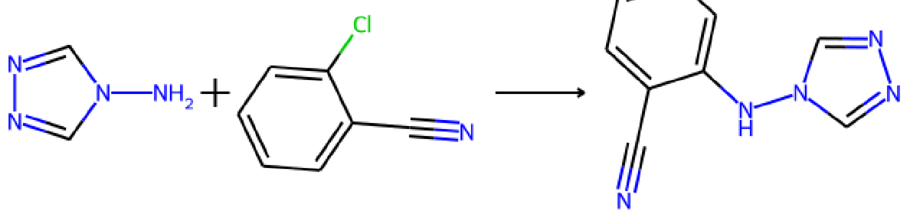

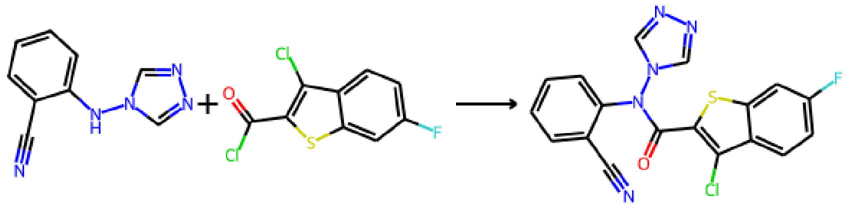

Product 147

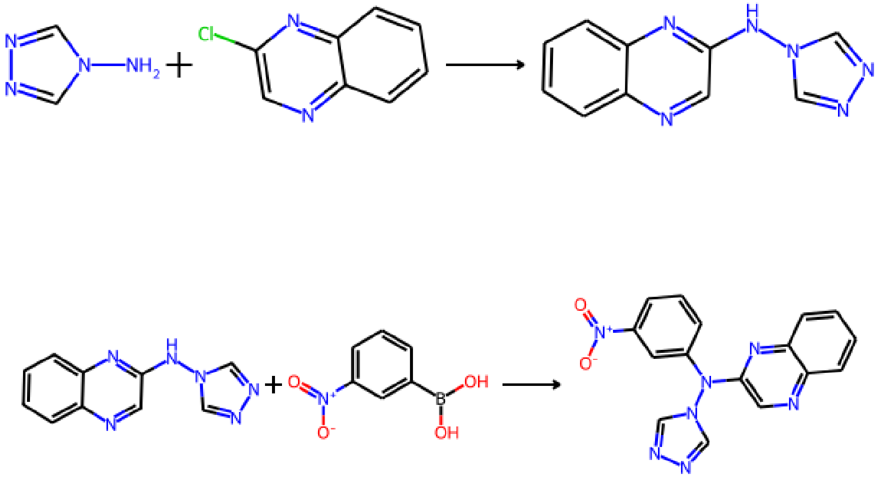

Product 148

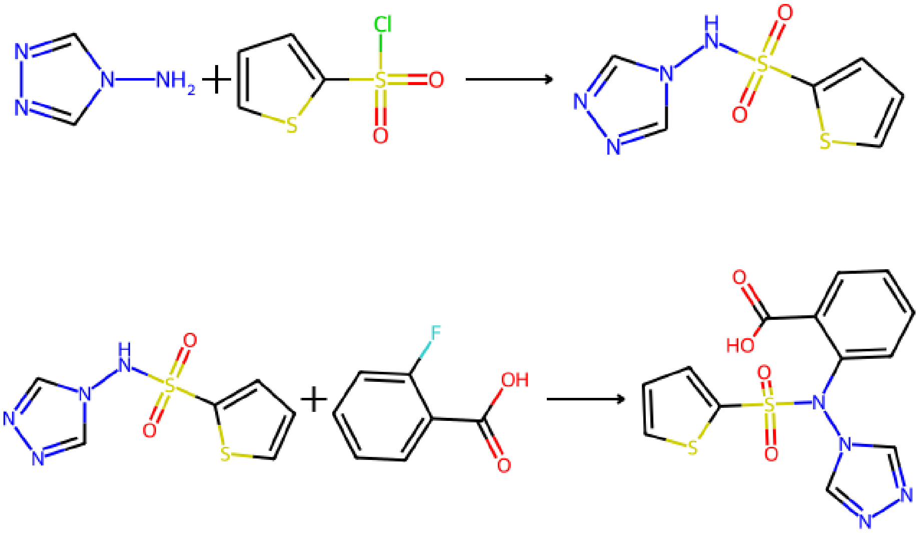

Product 149

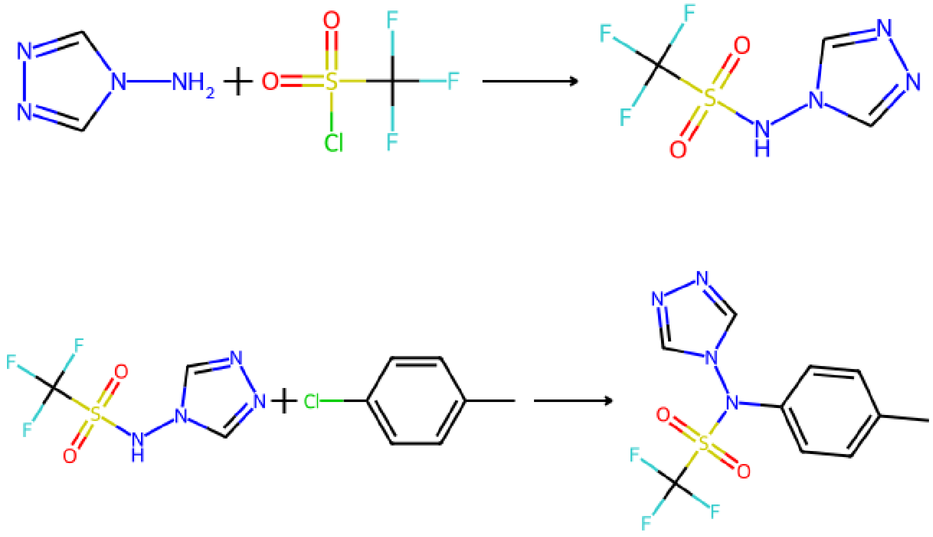

Product 150

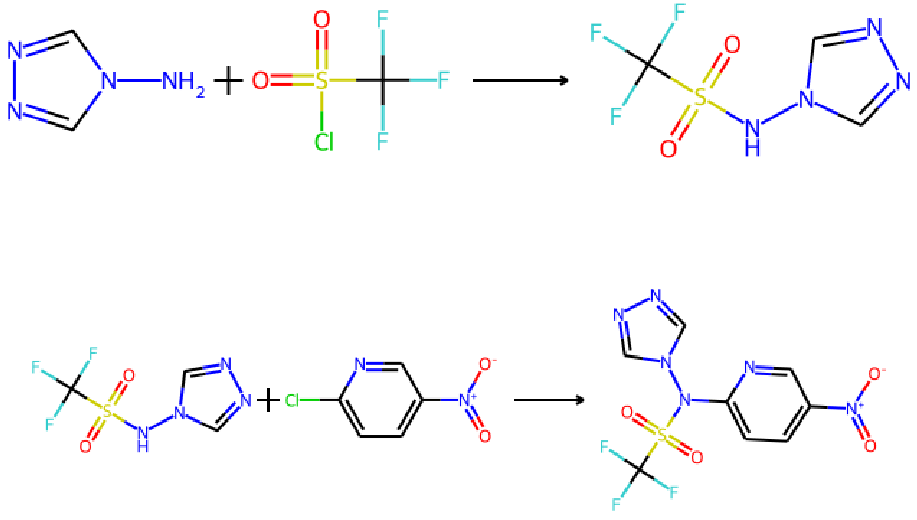

Product 151

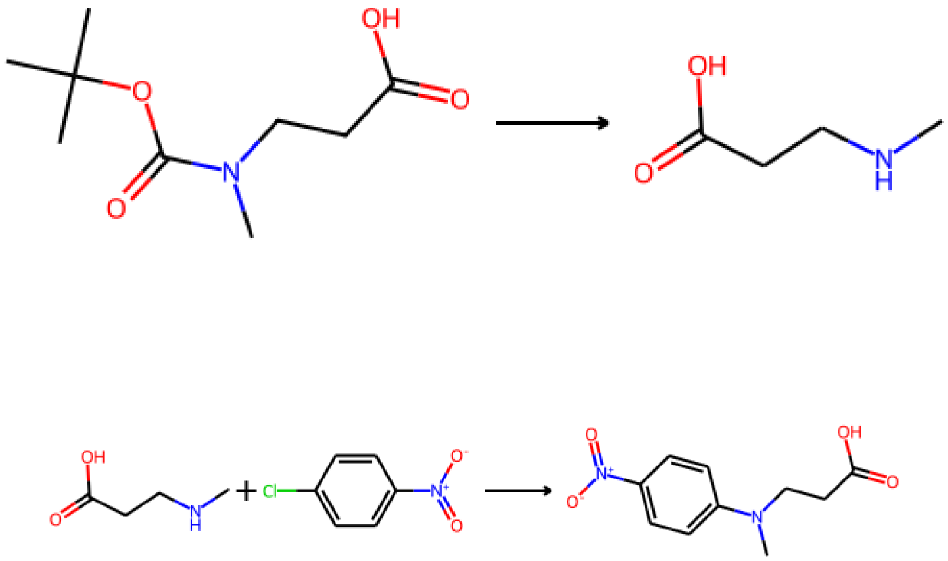

Product 152

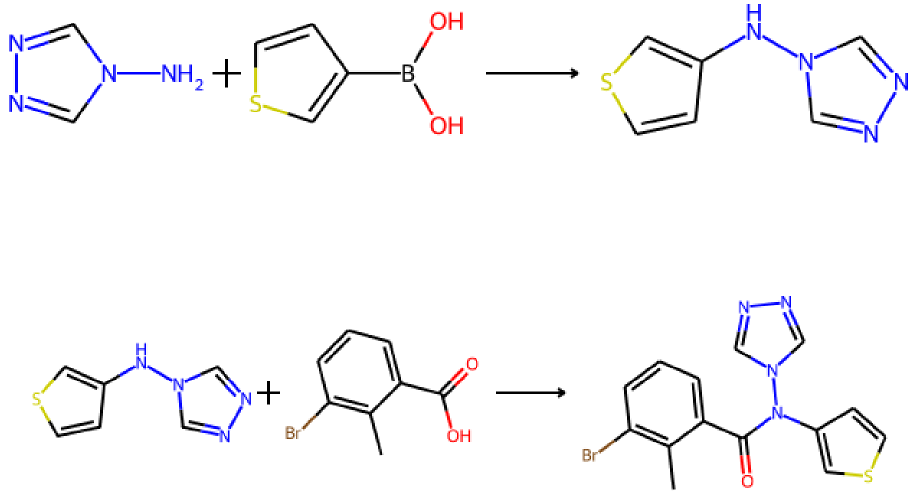

Product 153

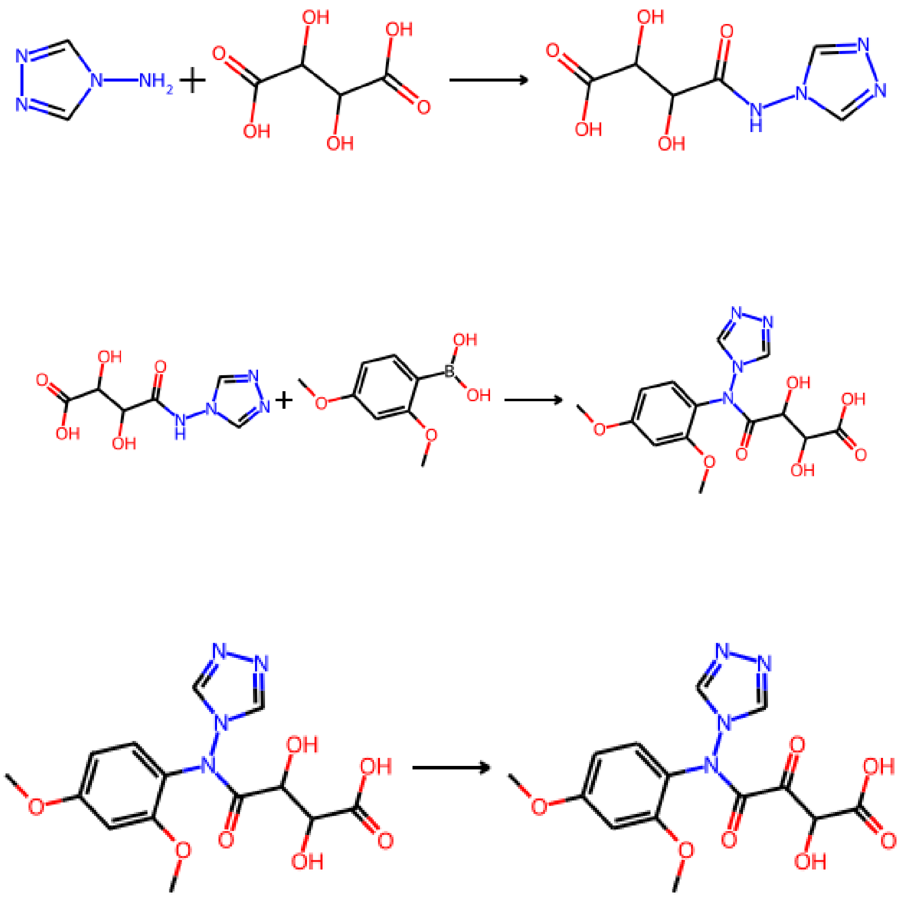

Product 154

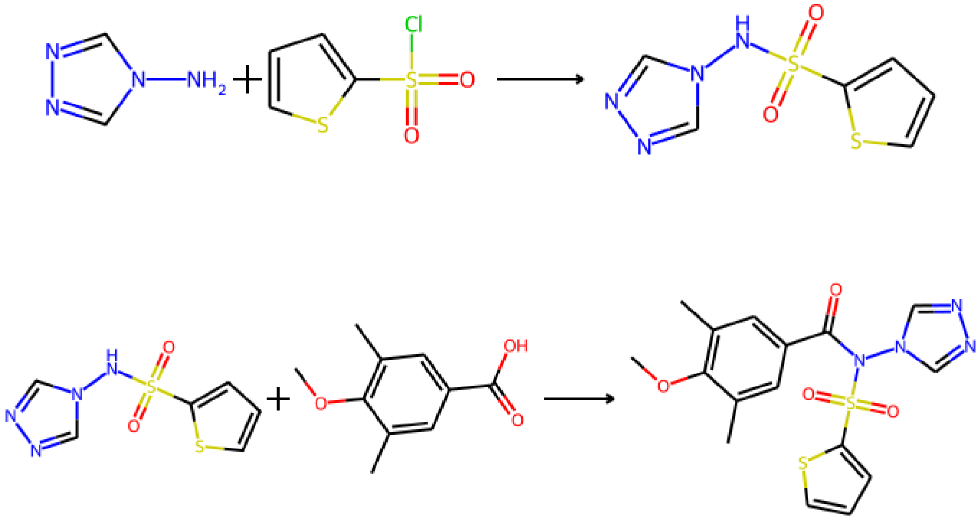

Product 155

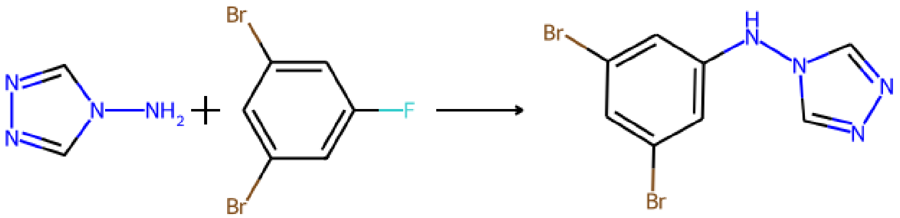

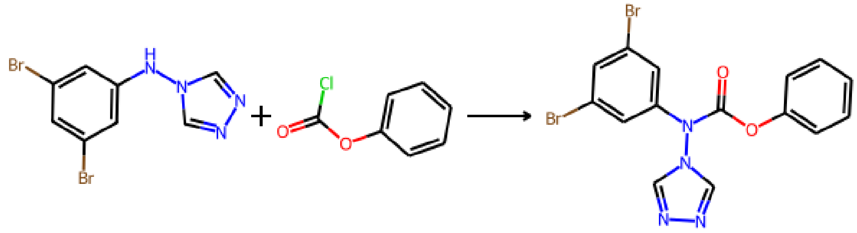

Product 156

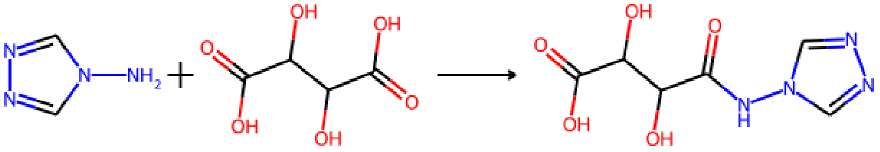

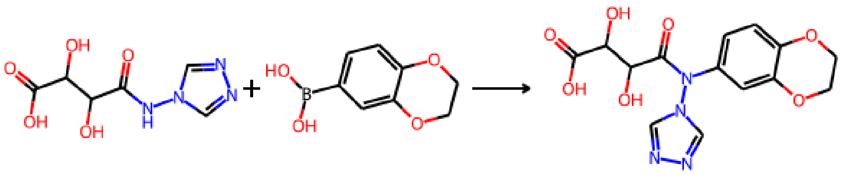

Product 157

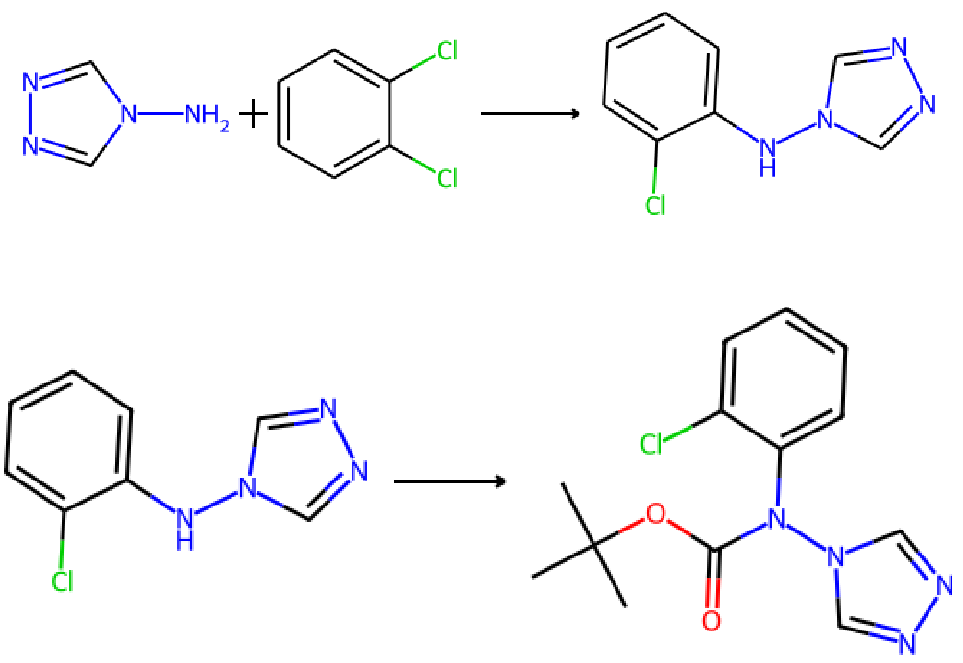

Product 158

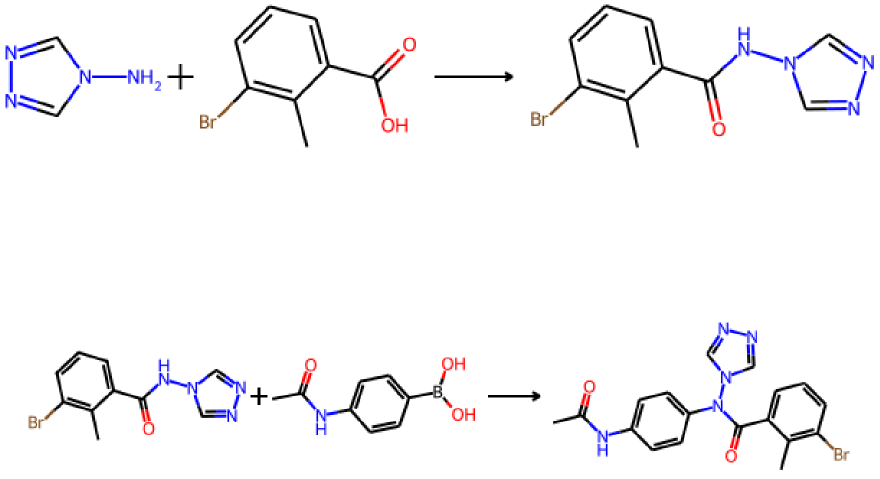

Product 159

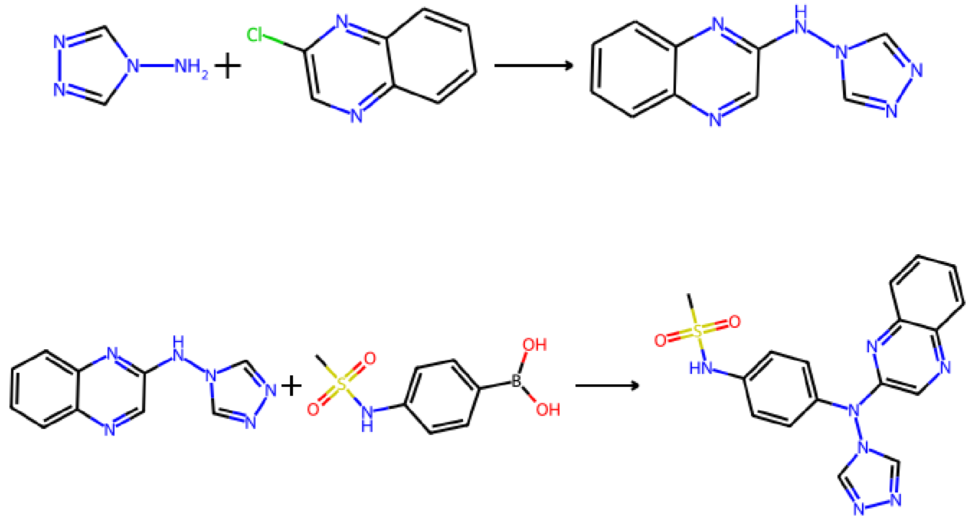

Product 160

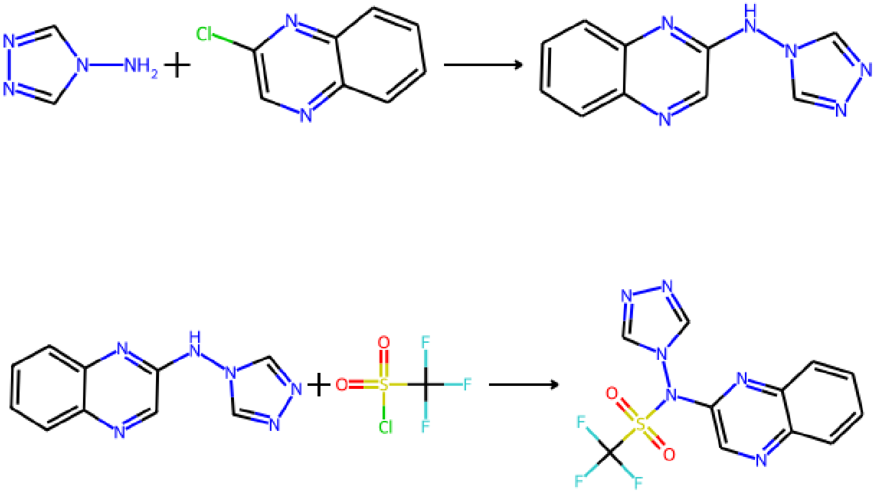

Product 161

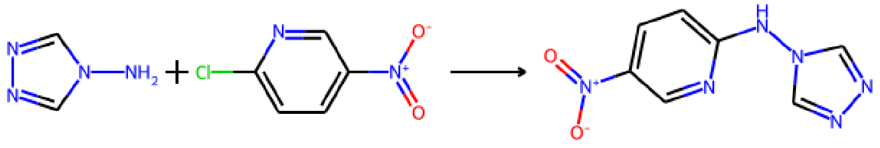

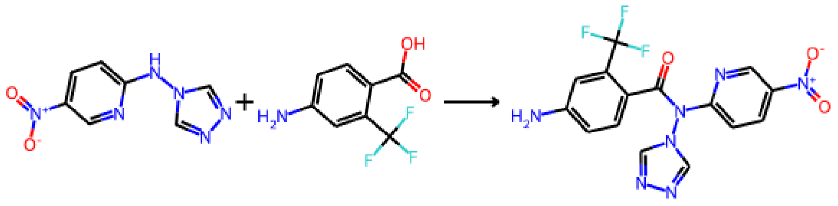

Product 162

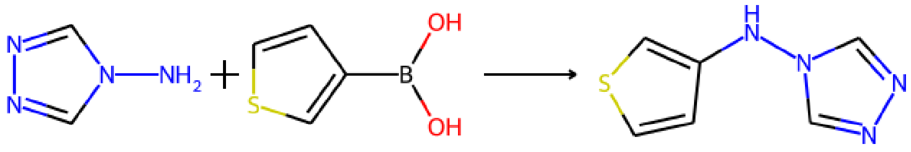

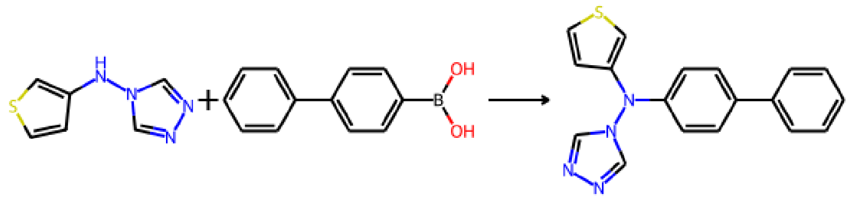

Product 163

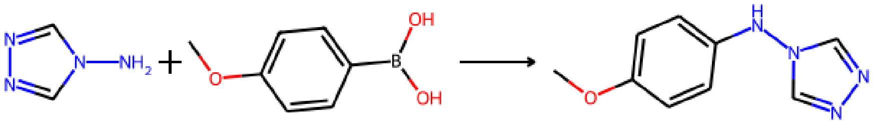

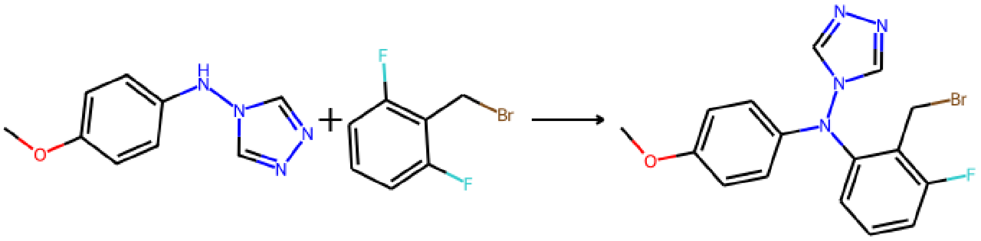

Product 164

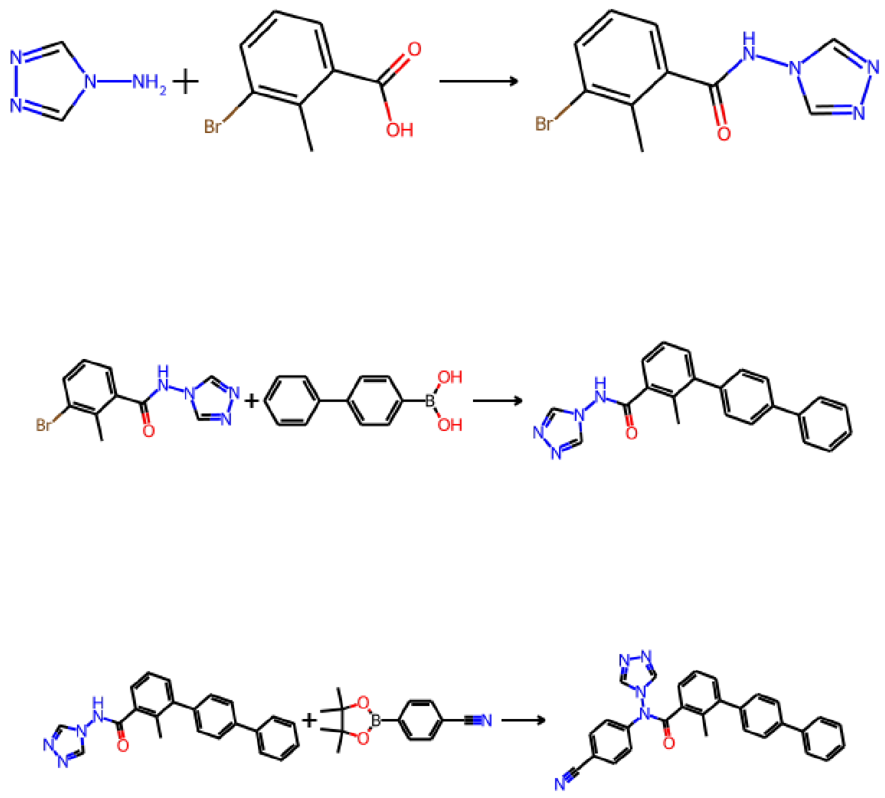

Product 165

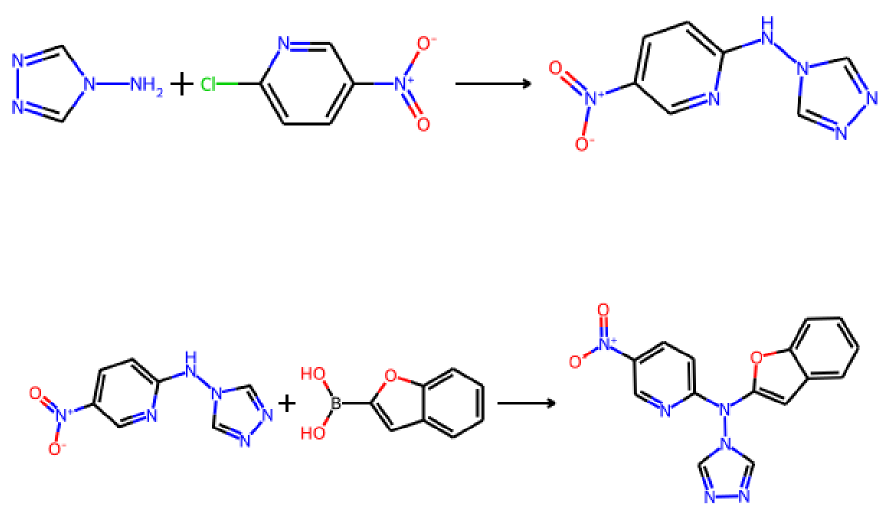

Product 166

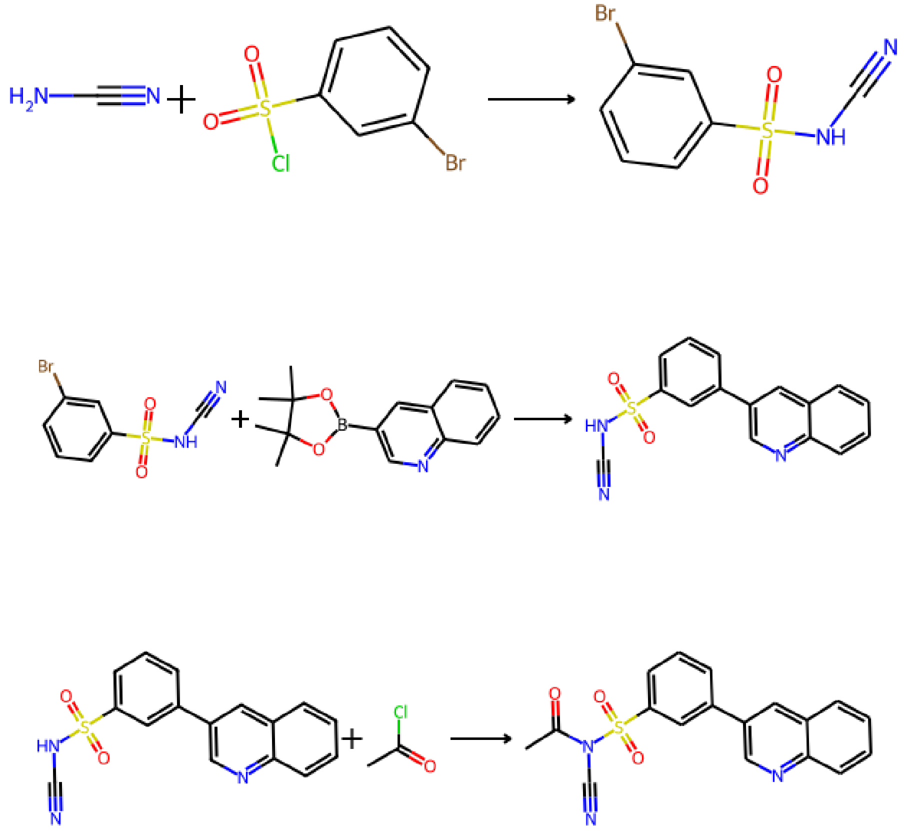

Product 167

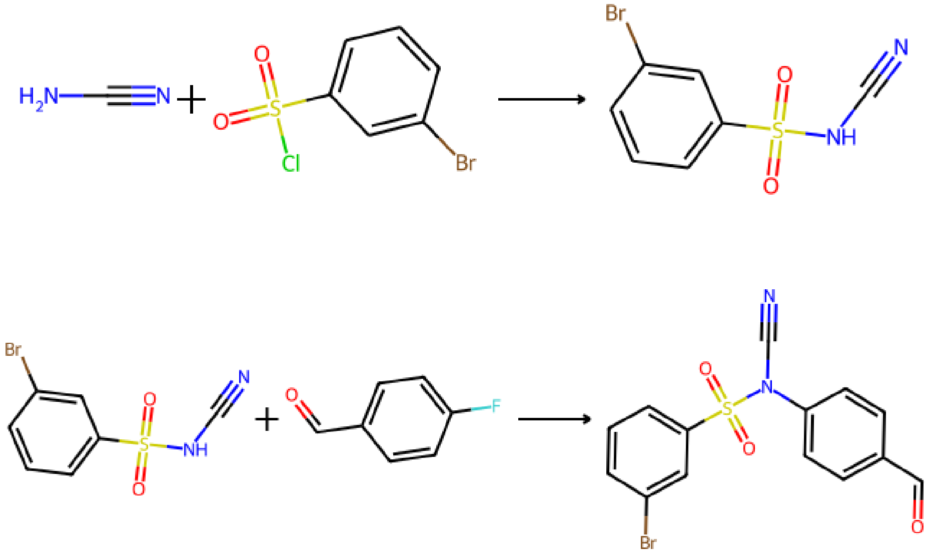

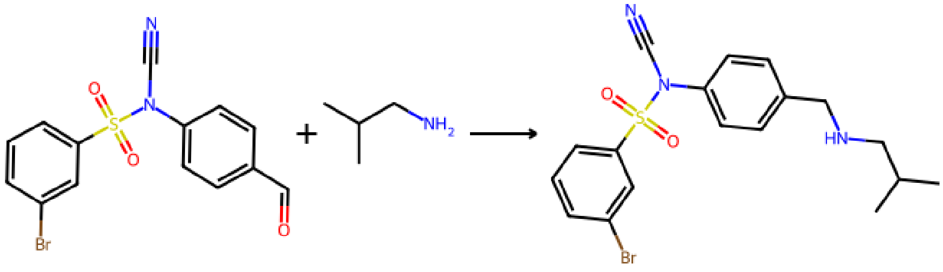

Product 168

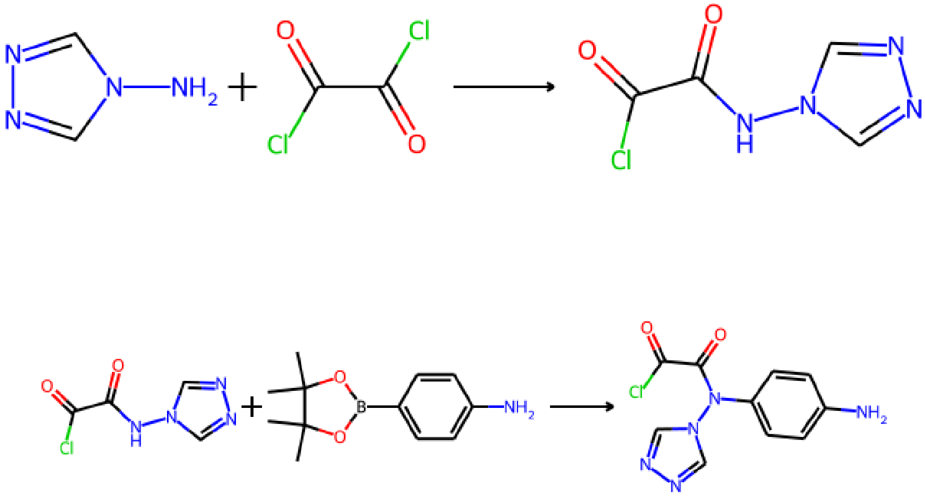

Product 169

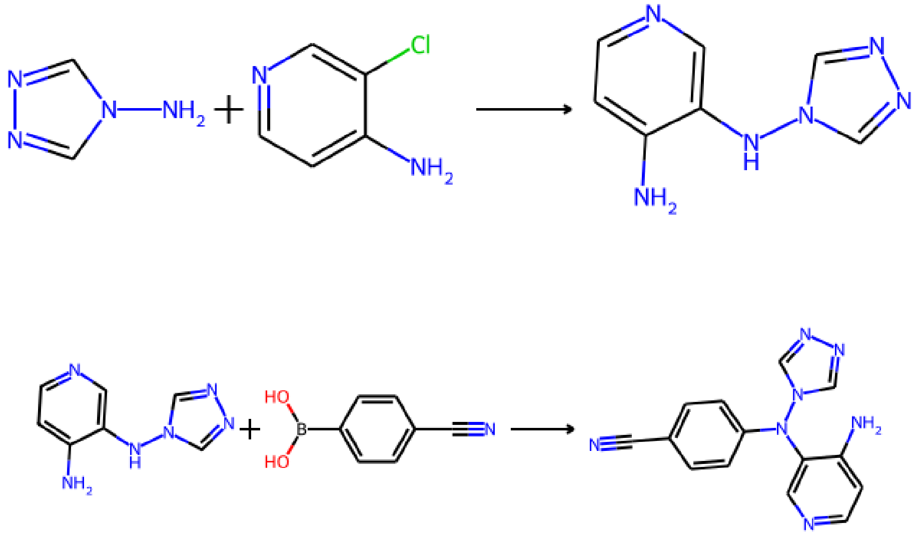

Product 170

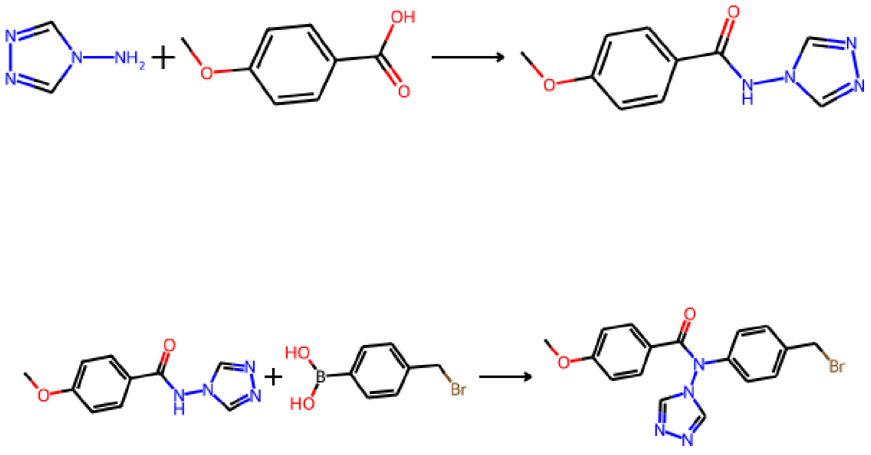

Product 171

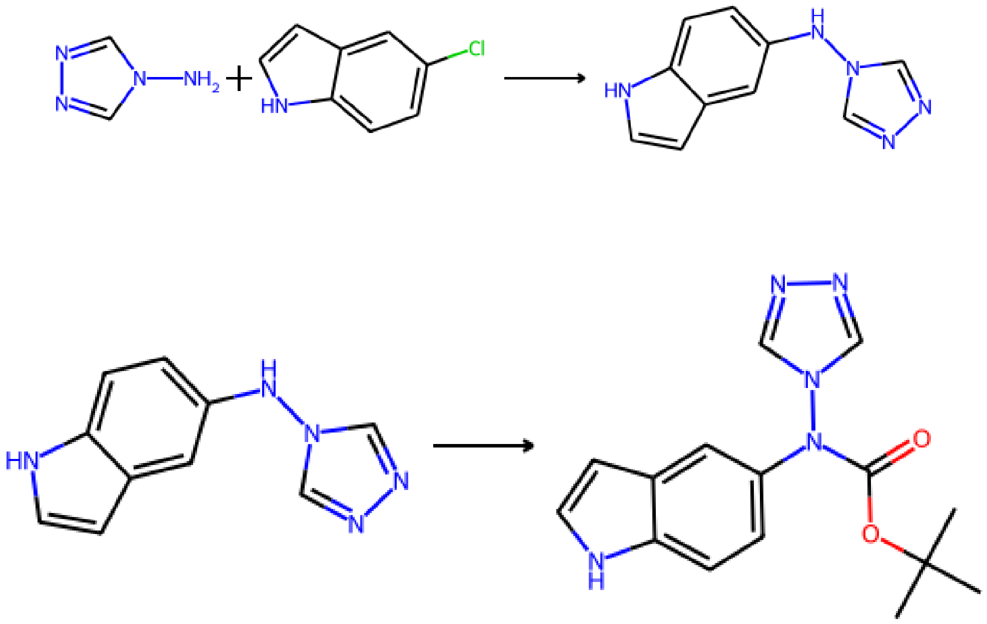

Product 172

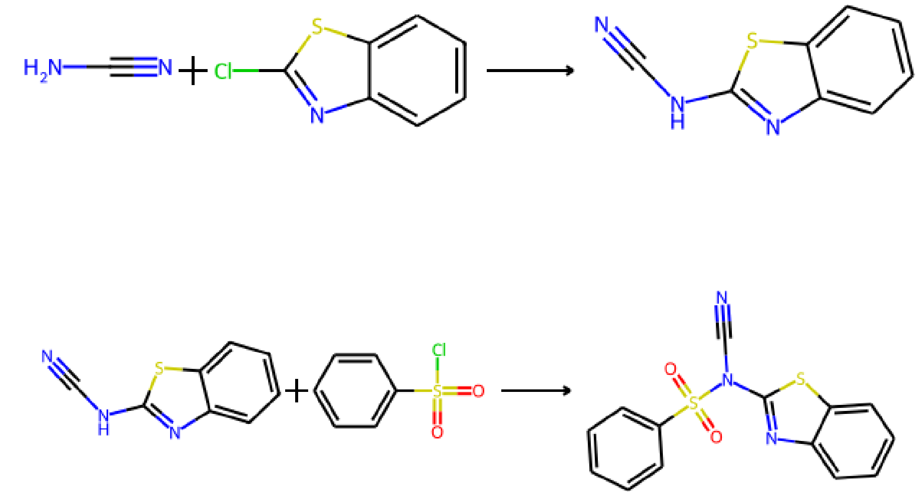

Product 173

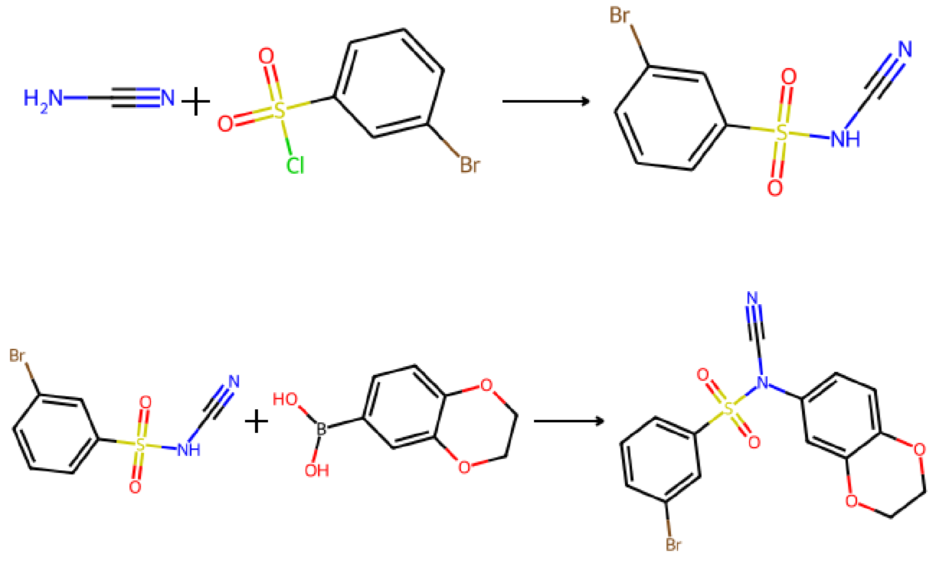

Product 174

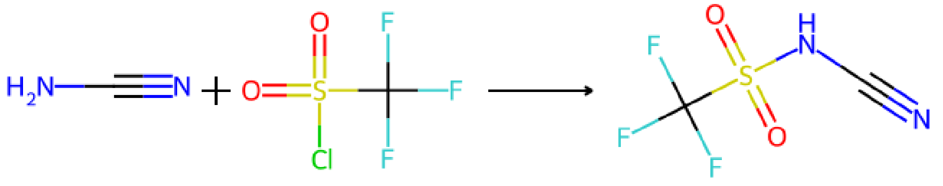

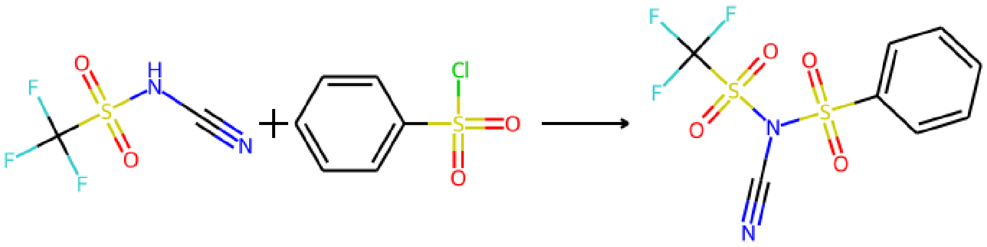

Product 175

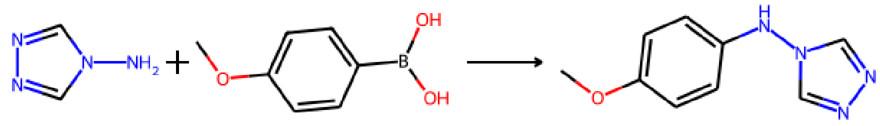

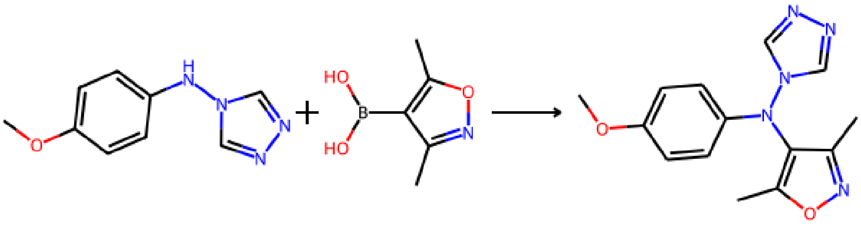

Product 176

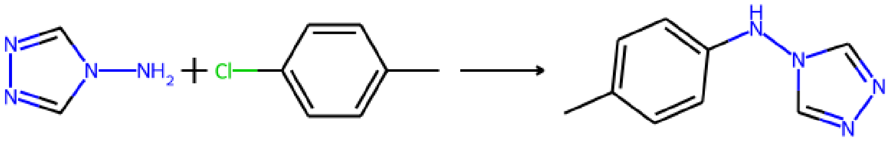

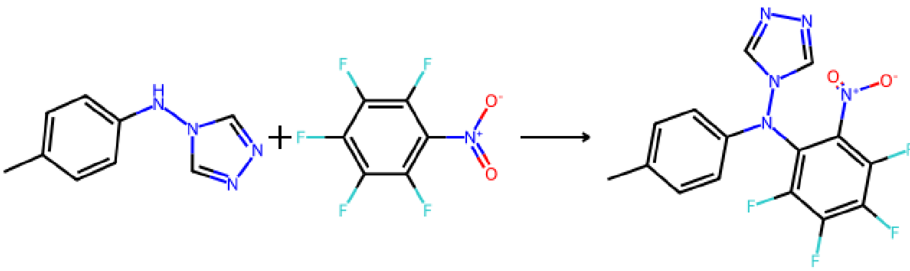

Product 177

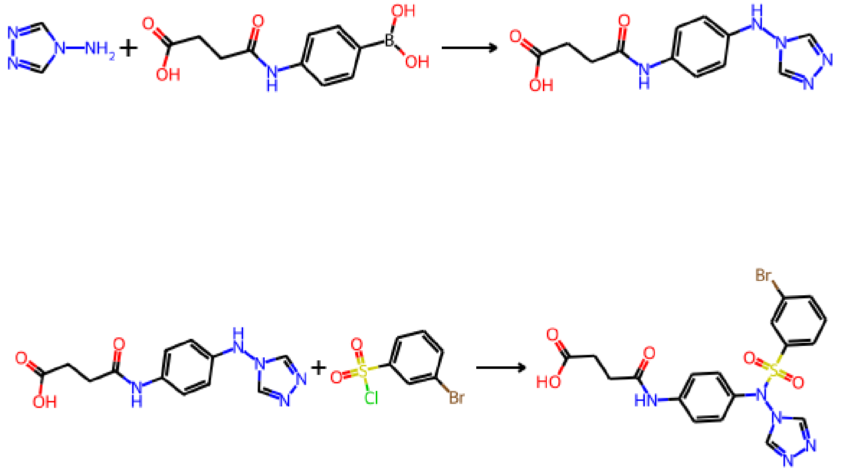

Product 178

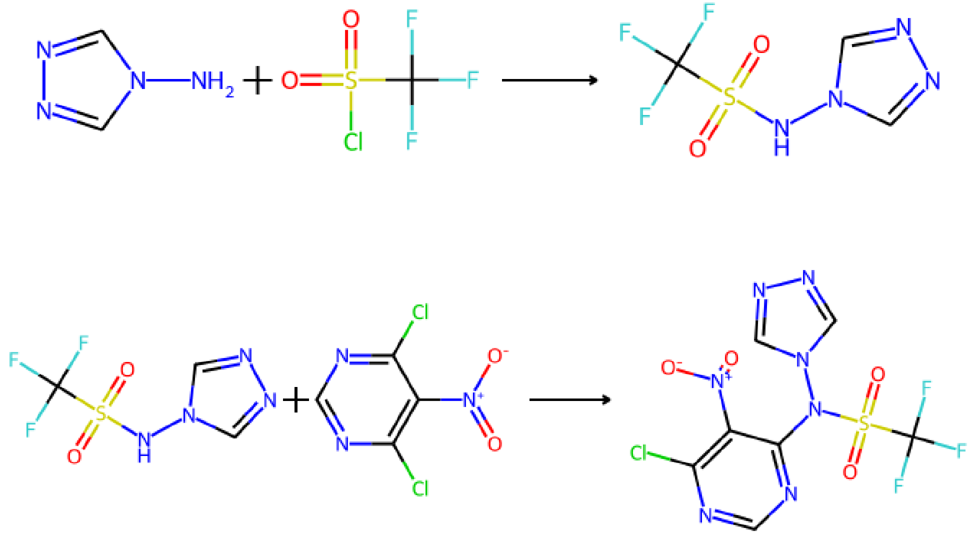

Product 179

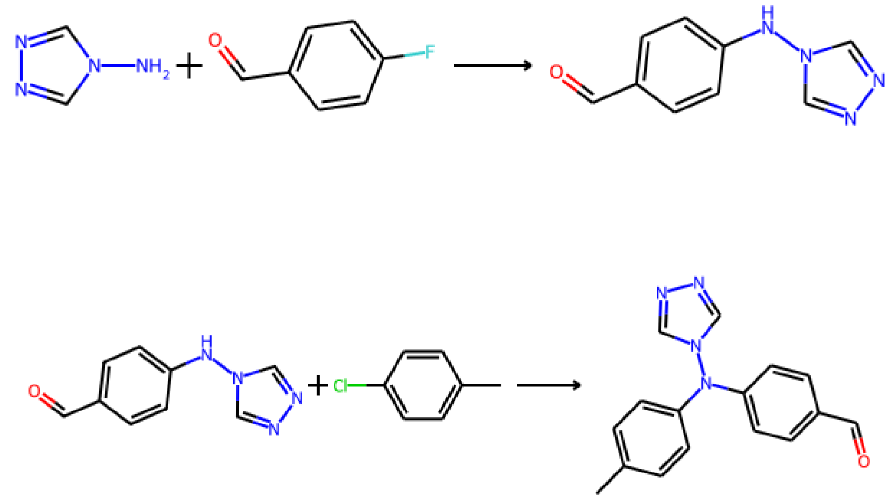

Product 180

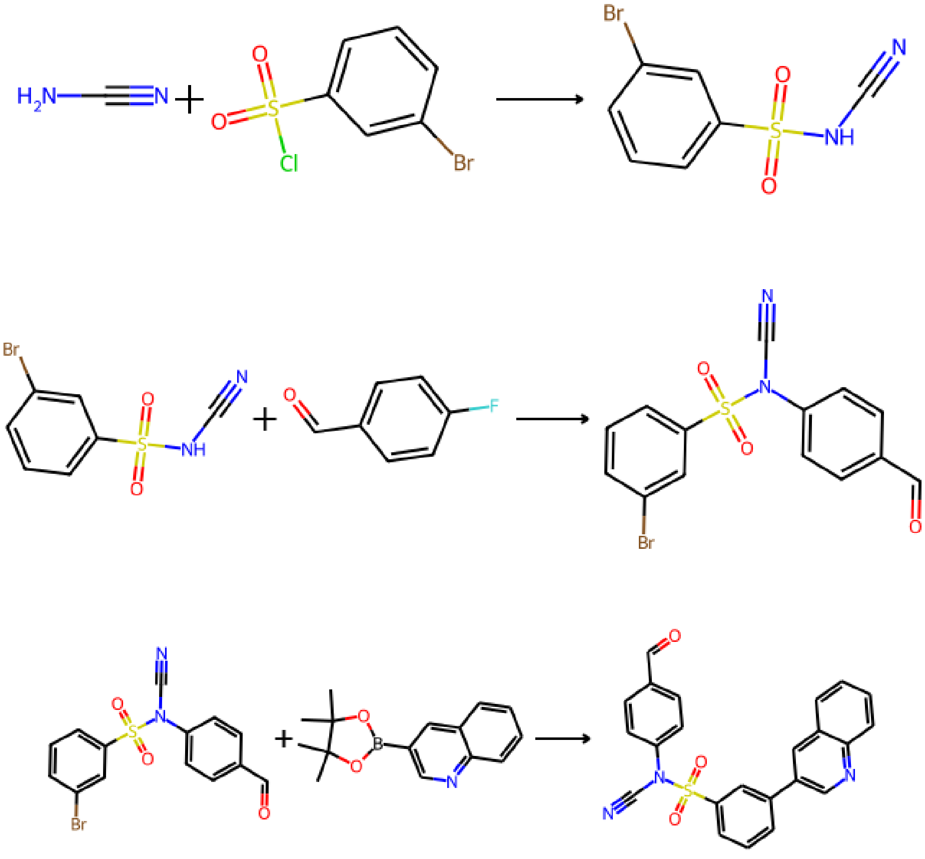

Product 181

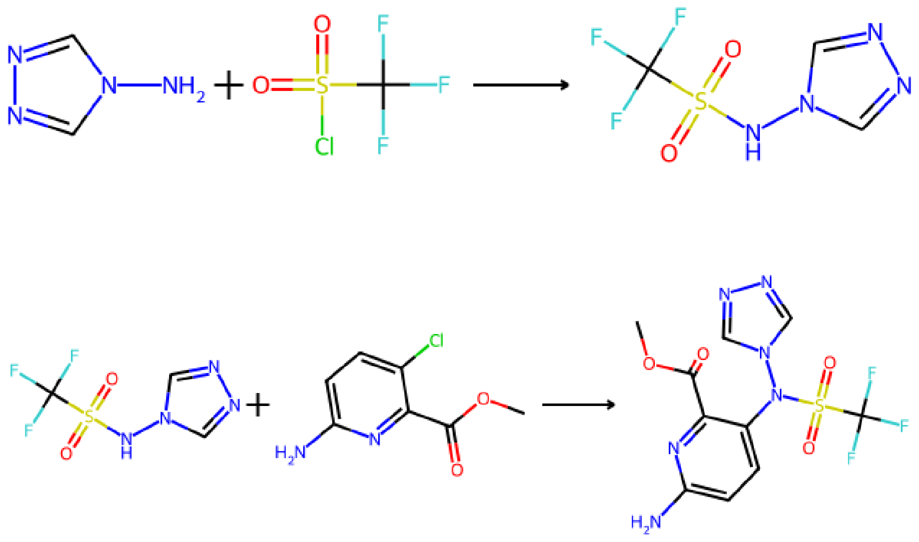

Product 182

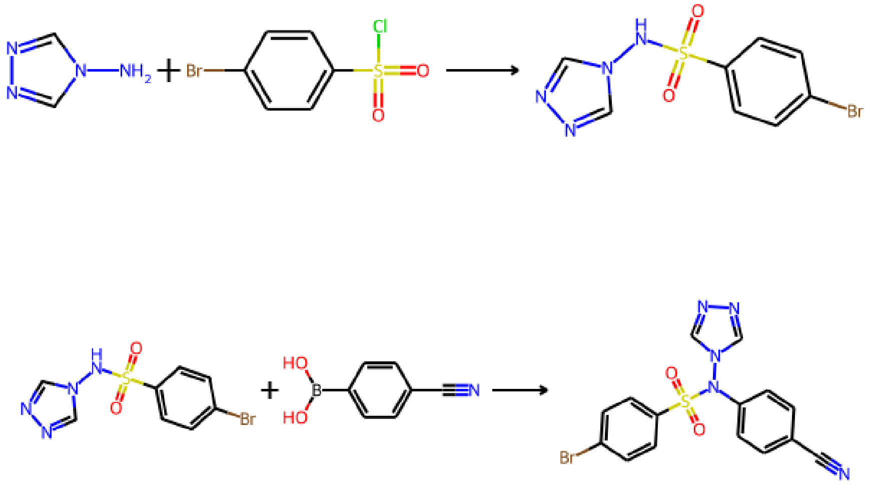

Product 183

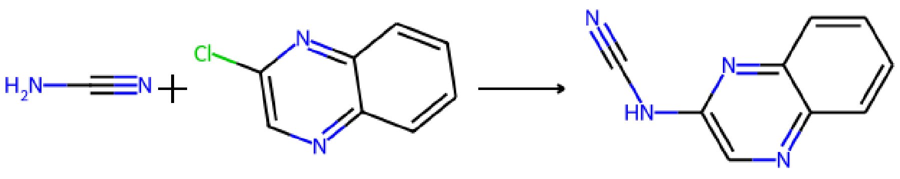

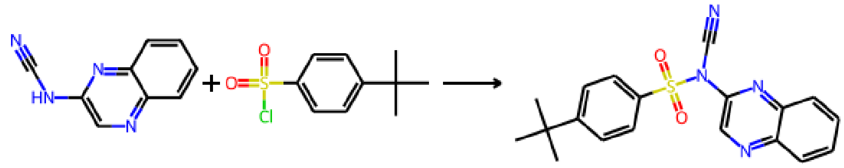

Product 184

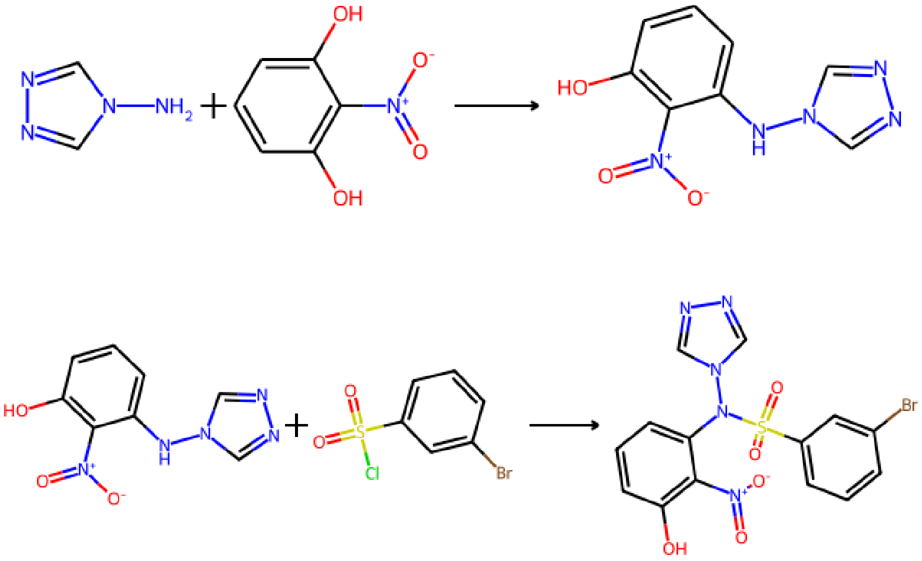

Product 185

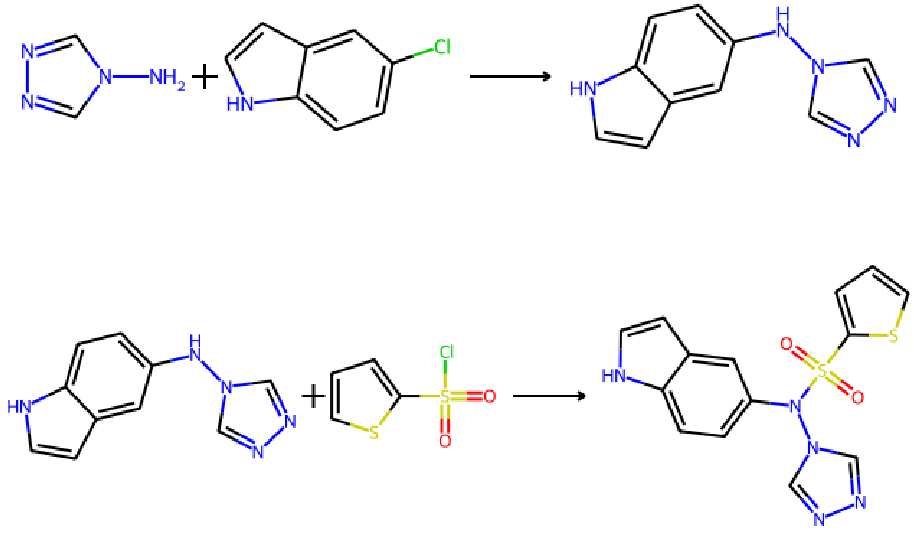

Product 186

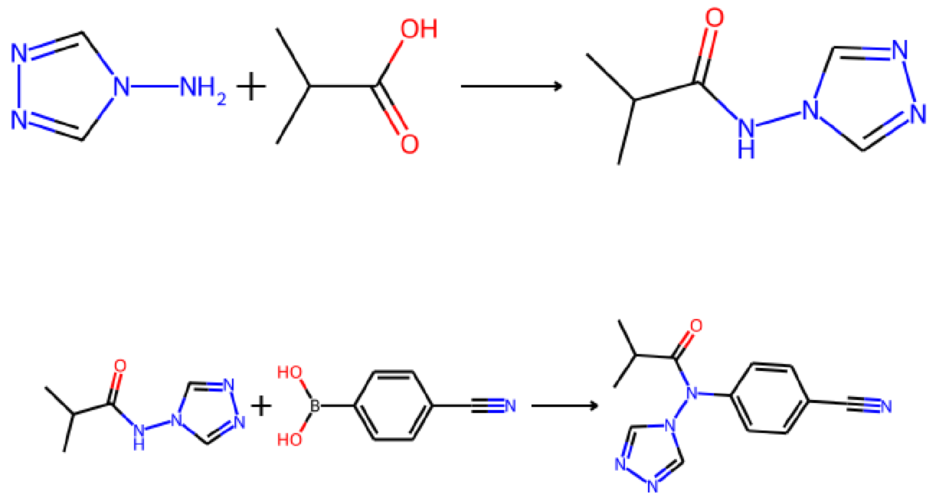

Product 187

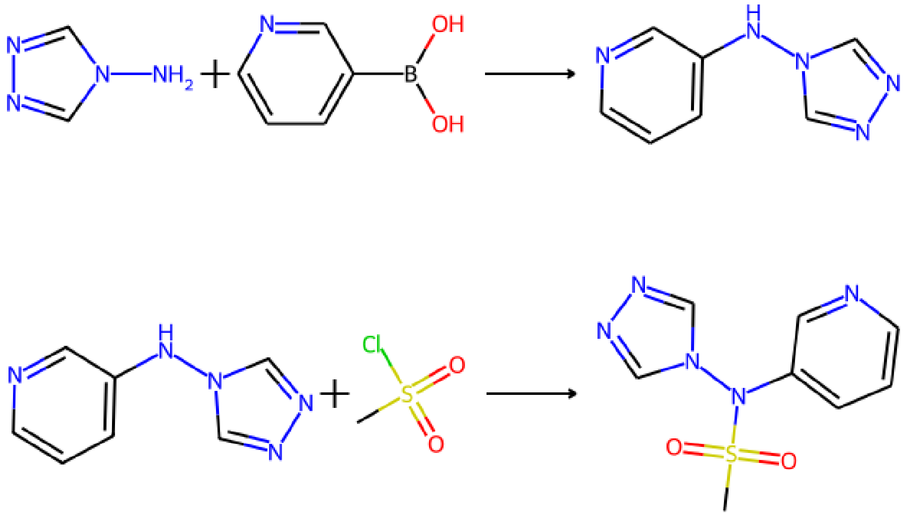

Product 188

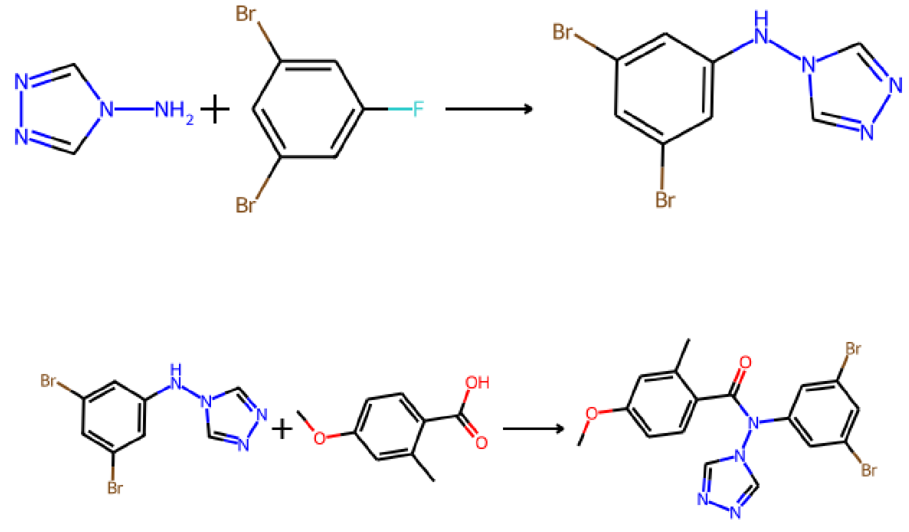

Product 189

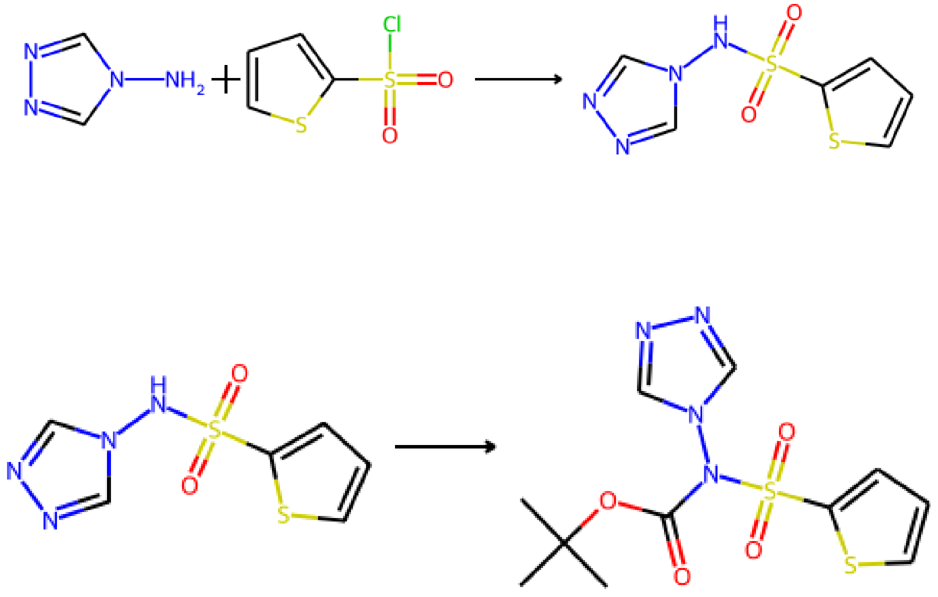

Product 190

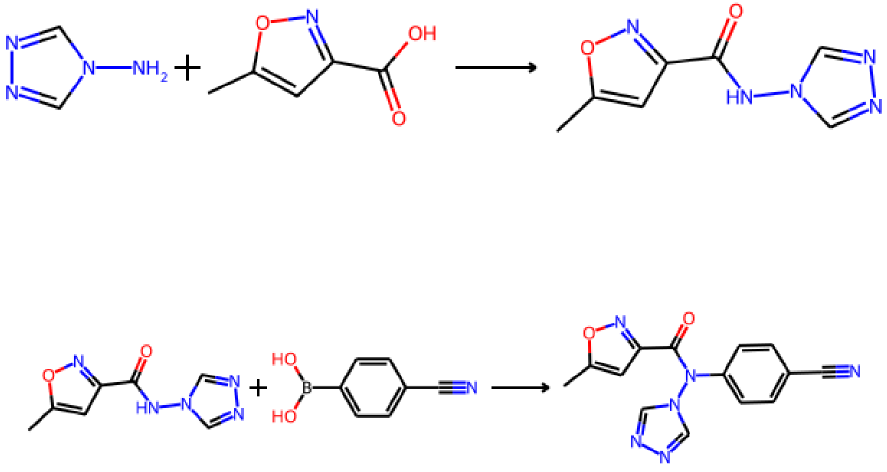

Product 191

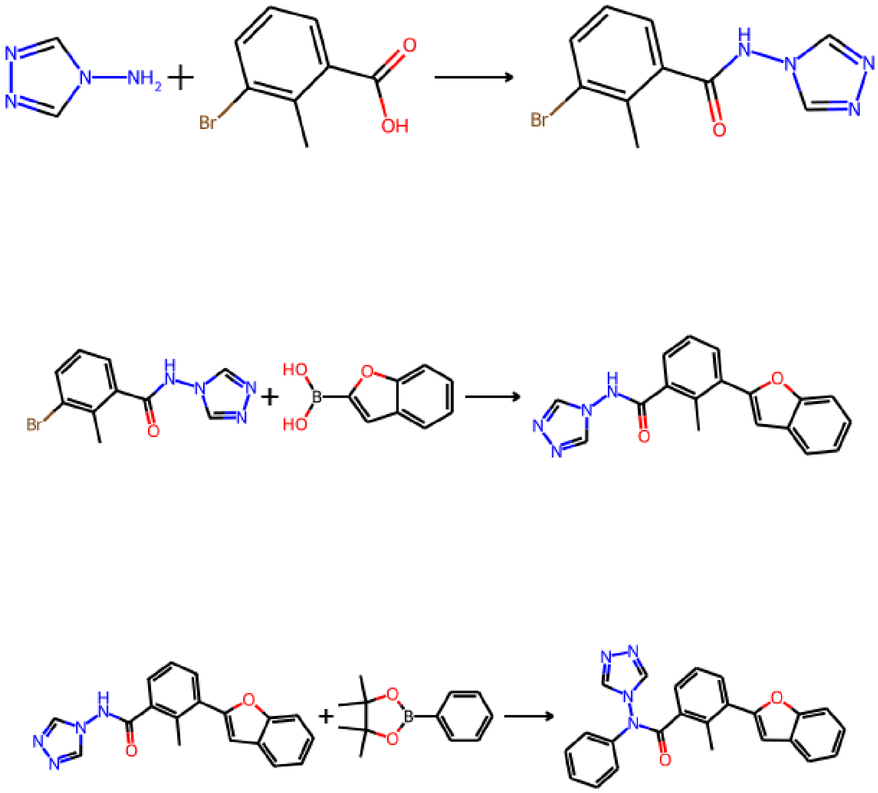

Product 192

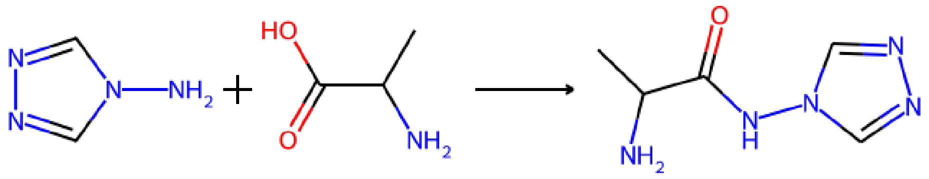

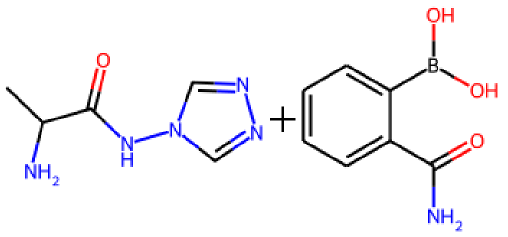

Product 193

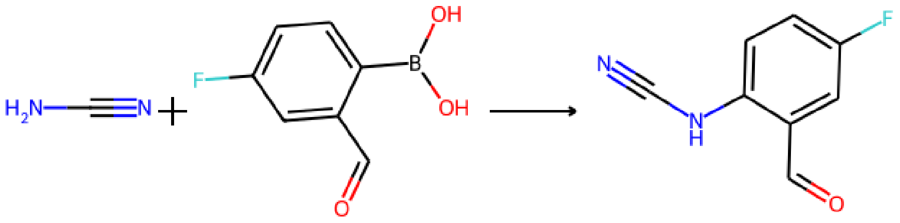

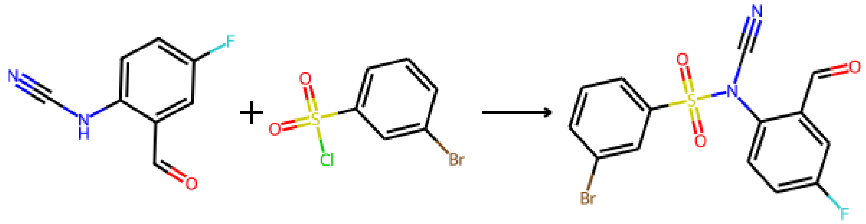

Product 194

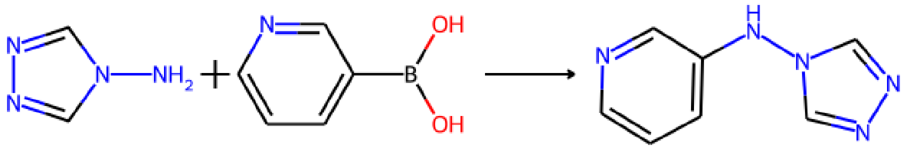

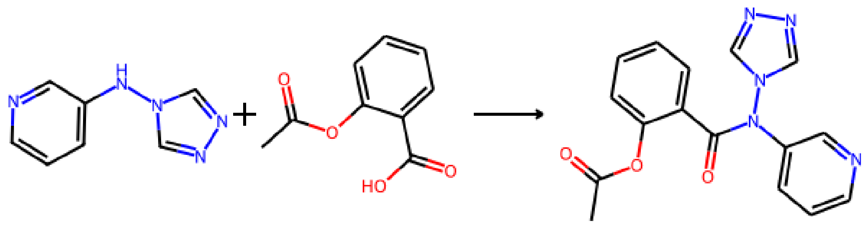

Product 195

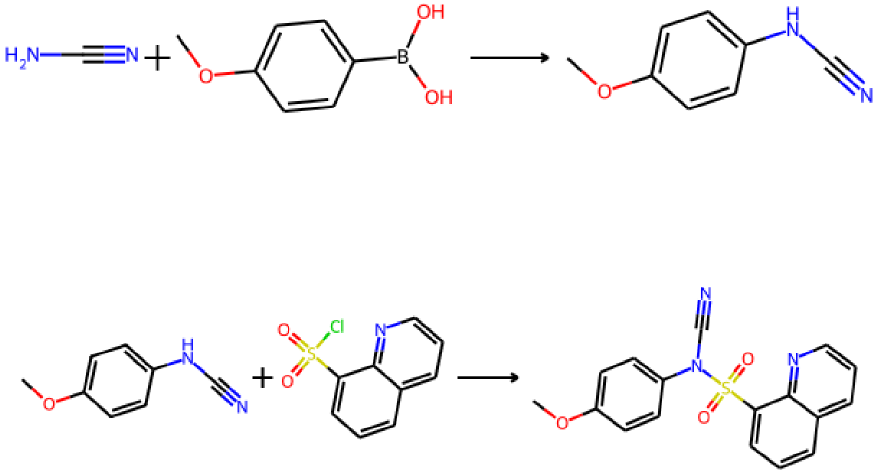

Product 196

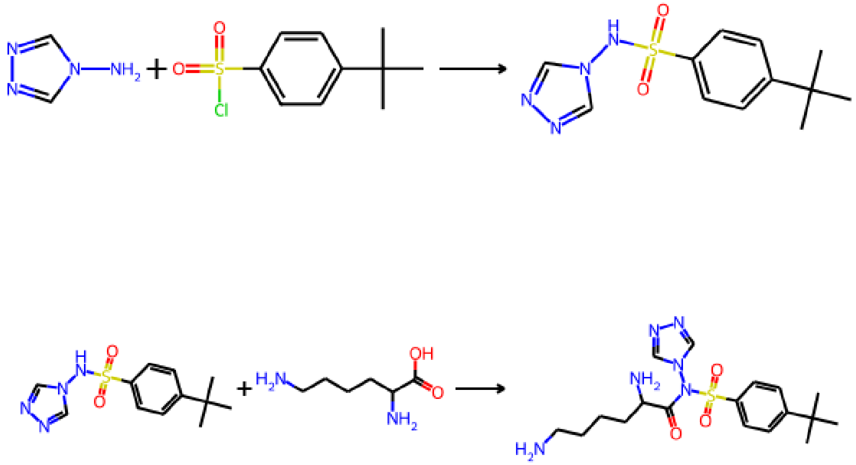

Product 197

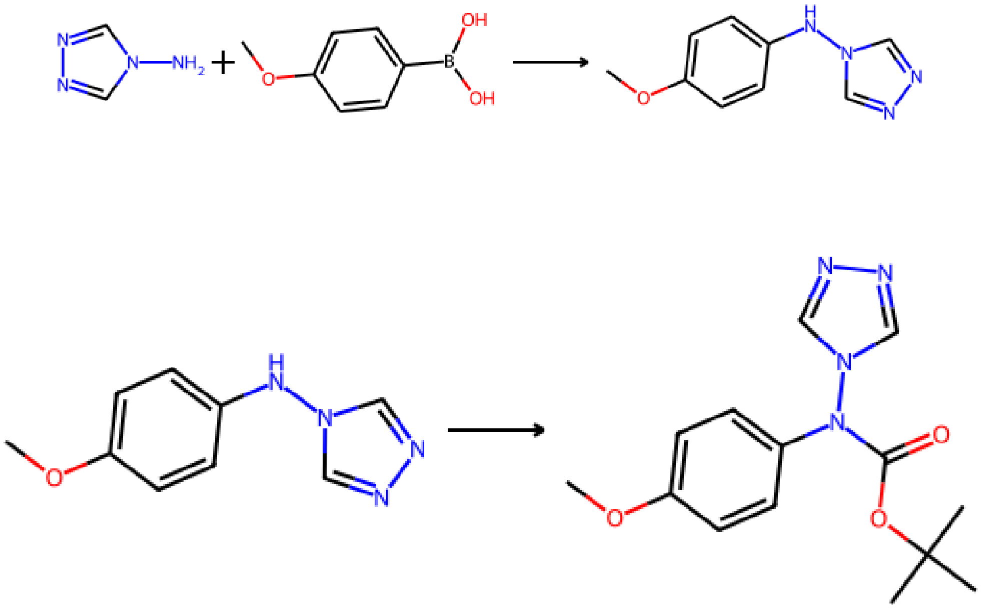

Product 198

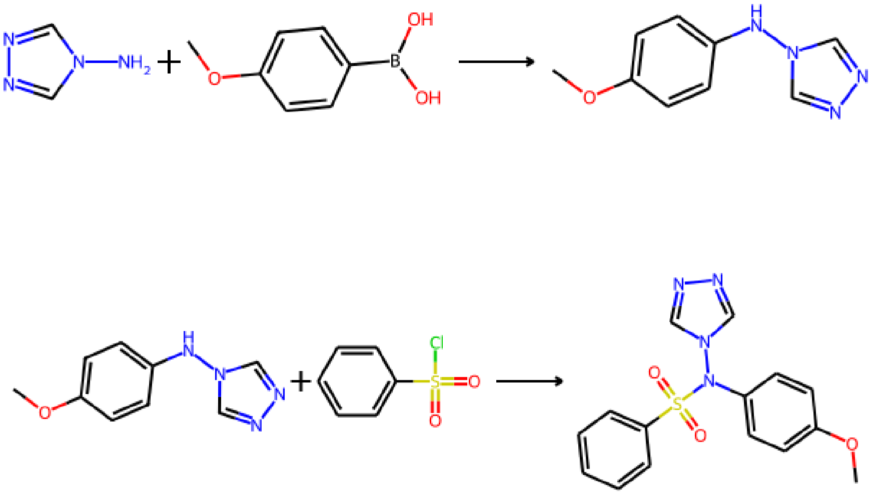

Product 199

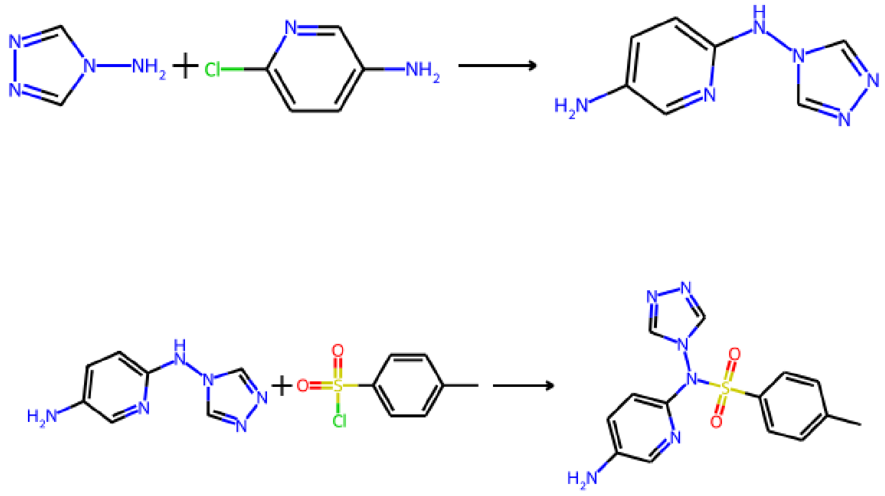

Product 200

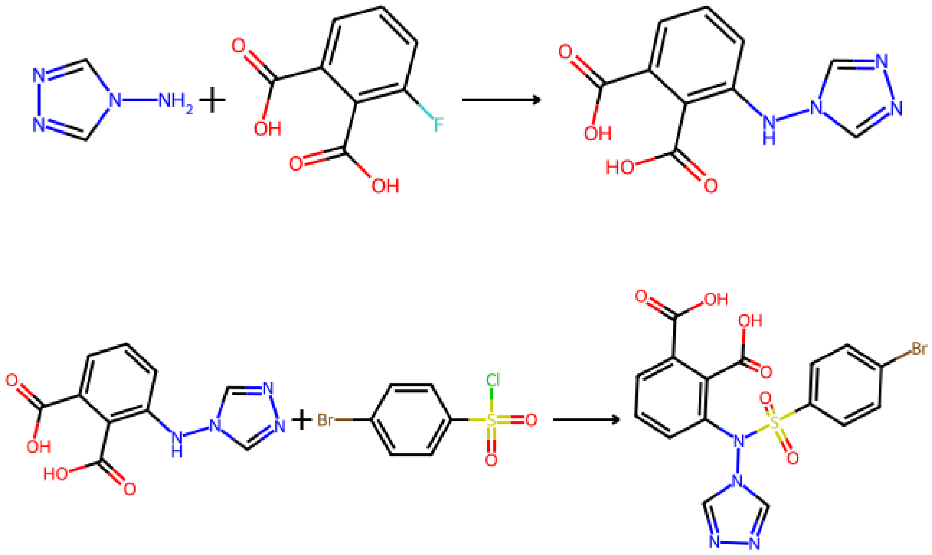

Product 201

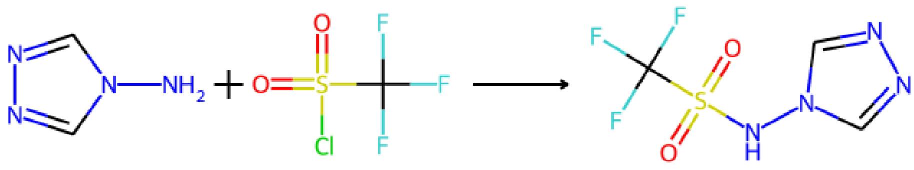

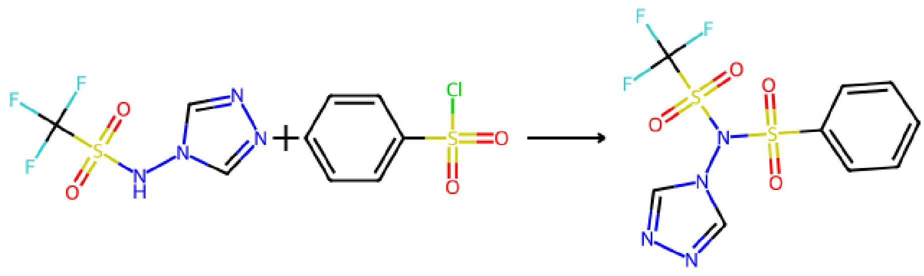

Product 202

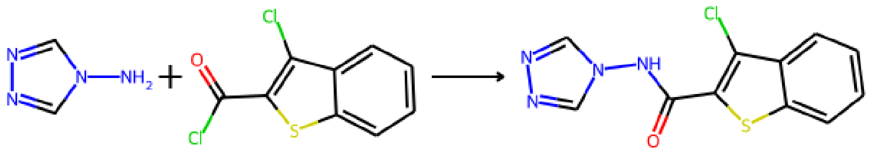

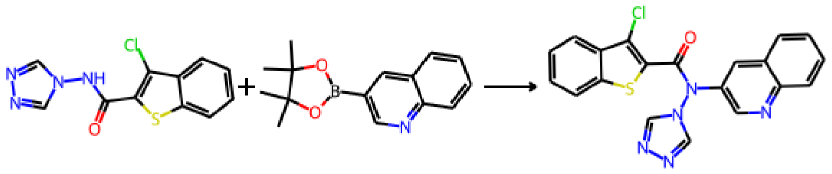

Product 203

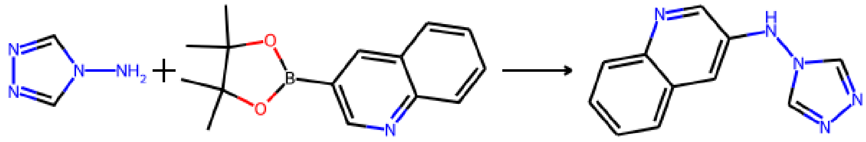

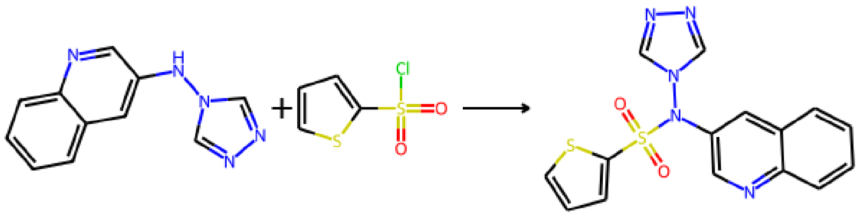

Product 204

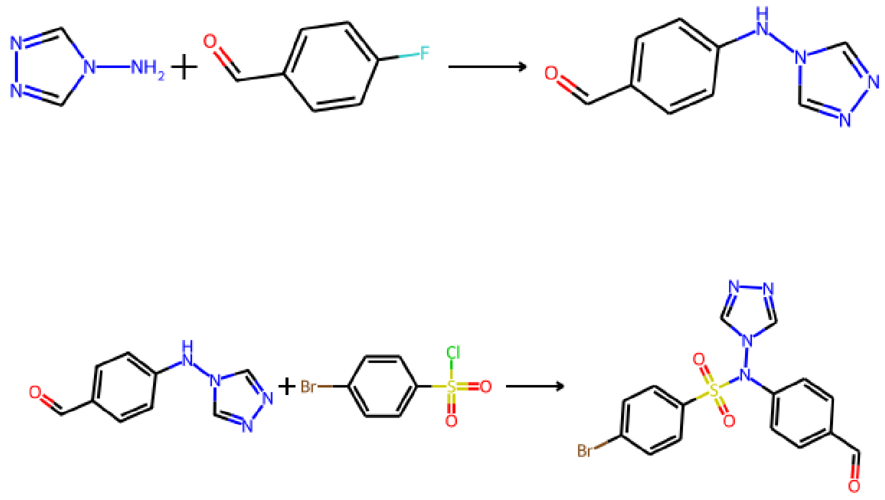

Product 205

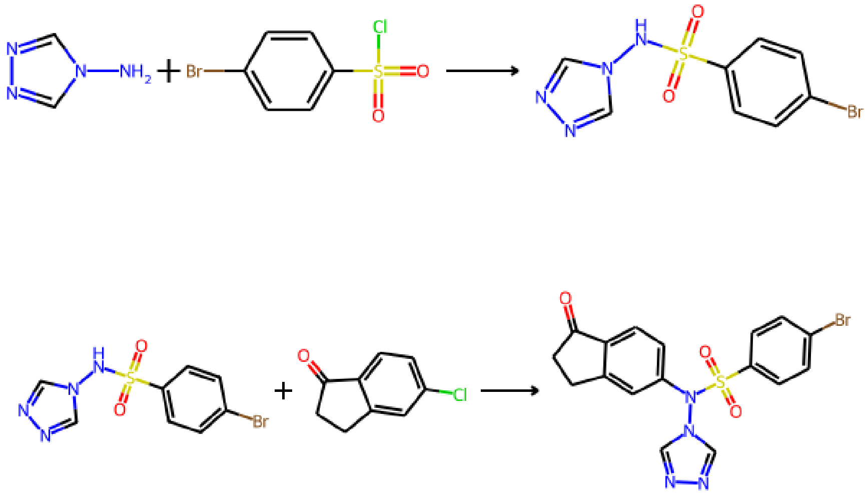

Product 206

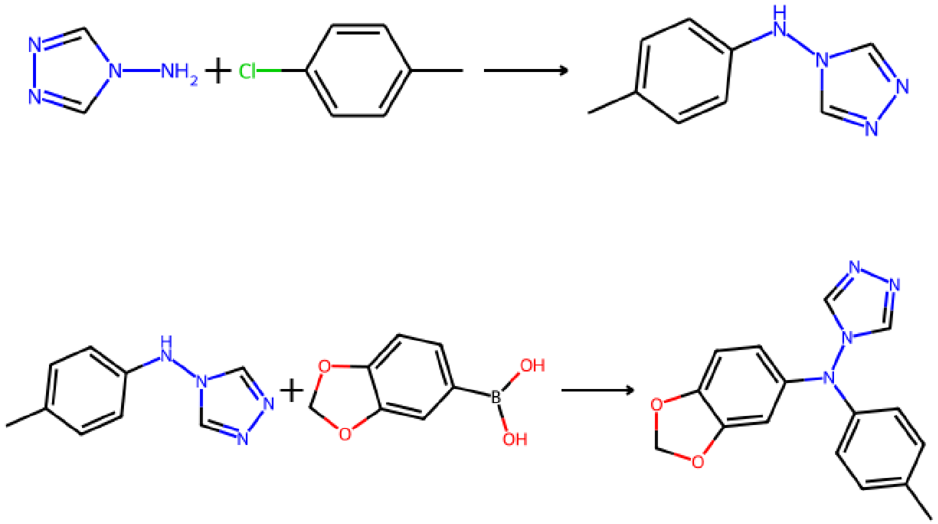

Product 207

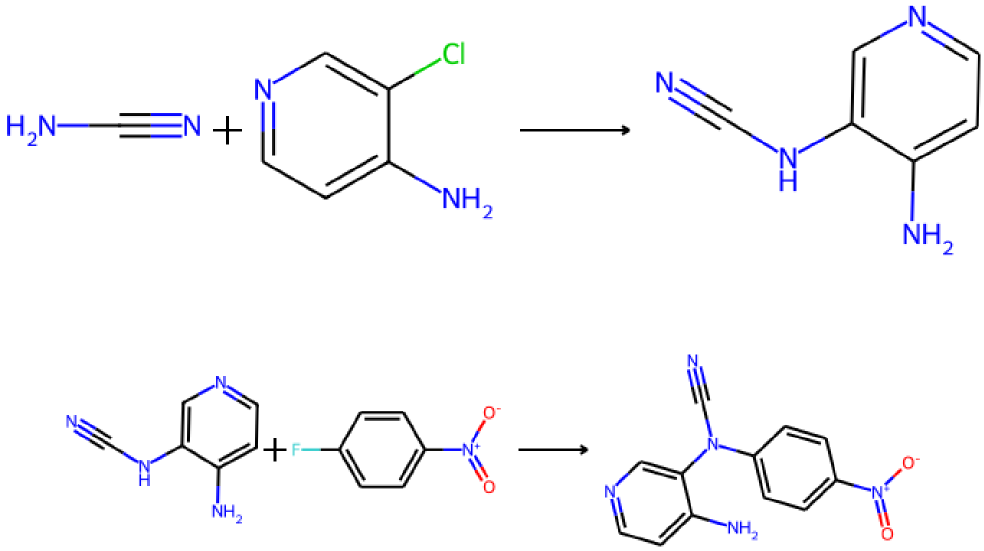

Product 208

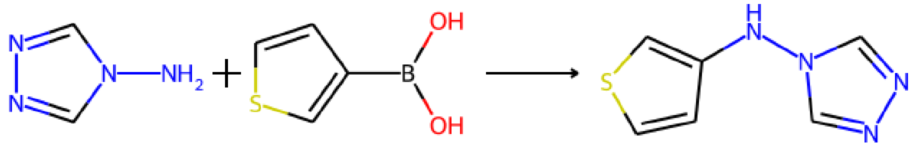

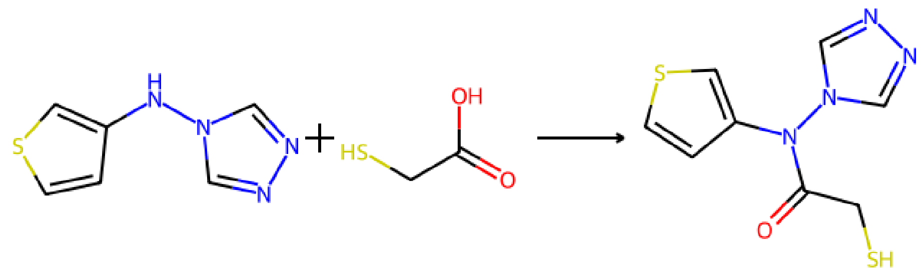

Product 209

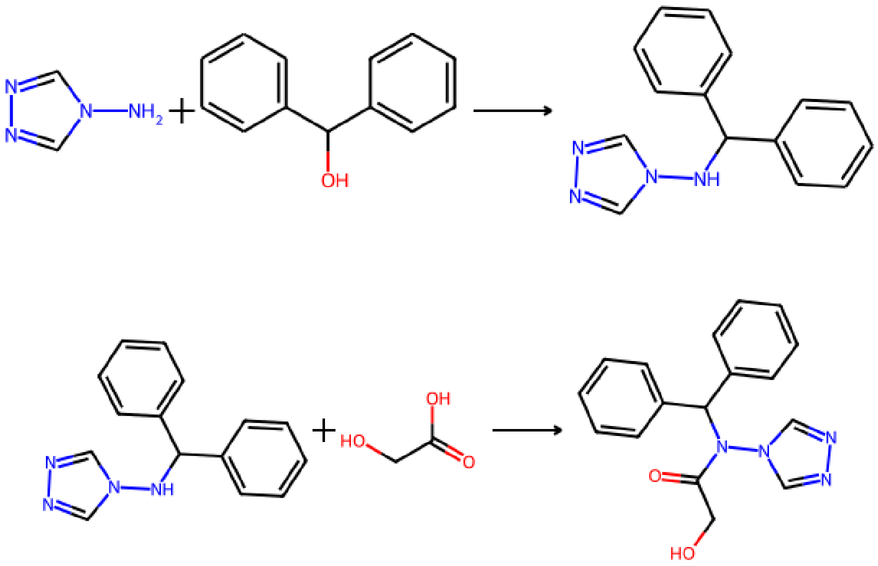

Product 210

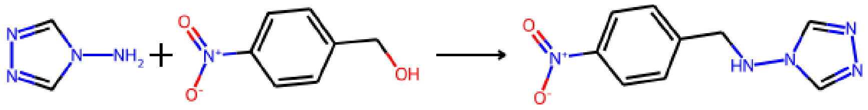

Product 211

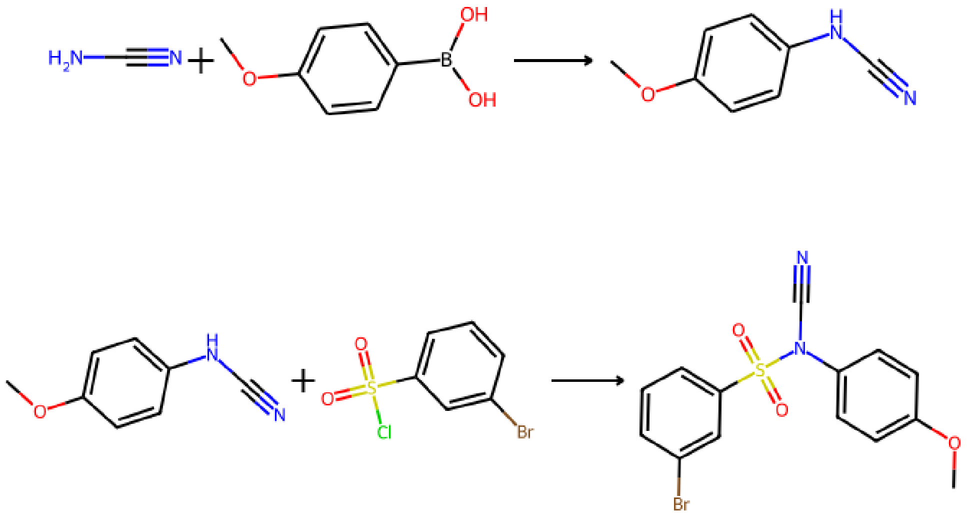

Product 212

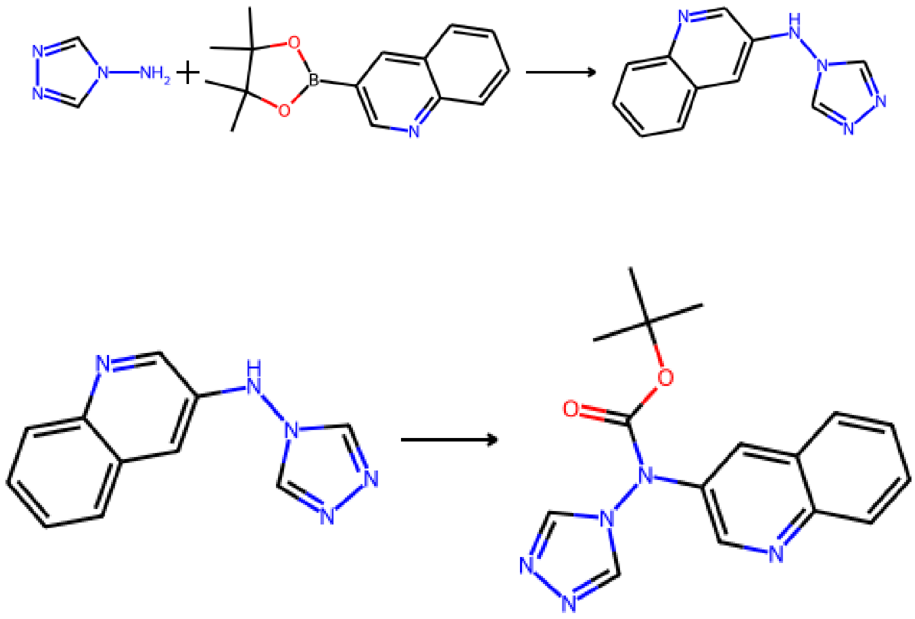

Product 213

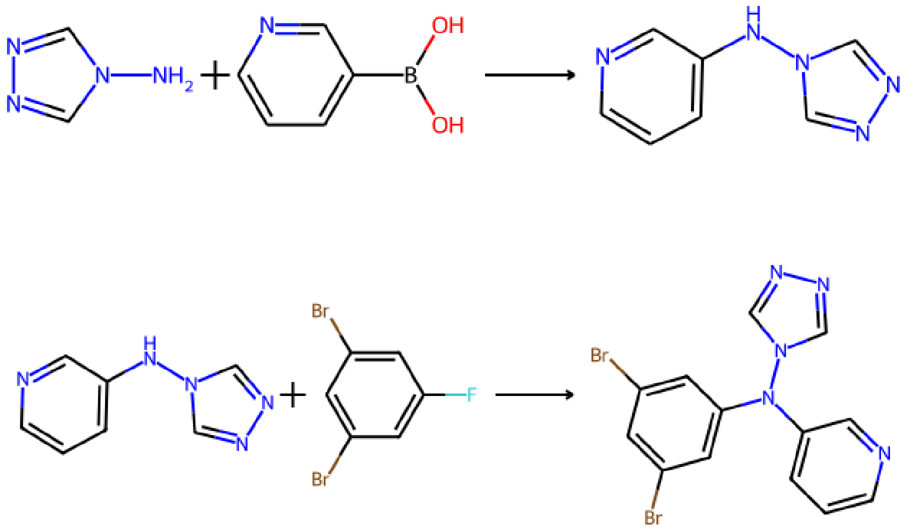

Product 214

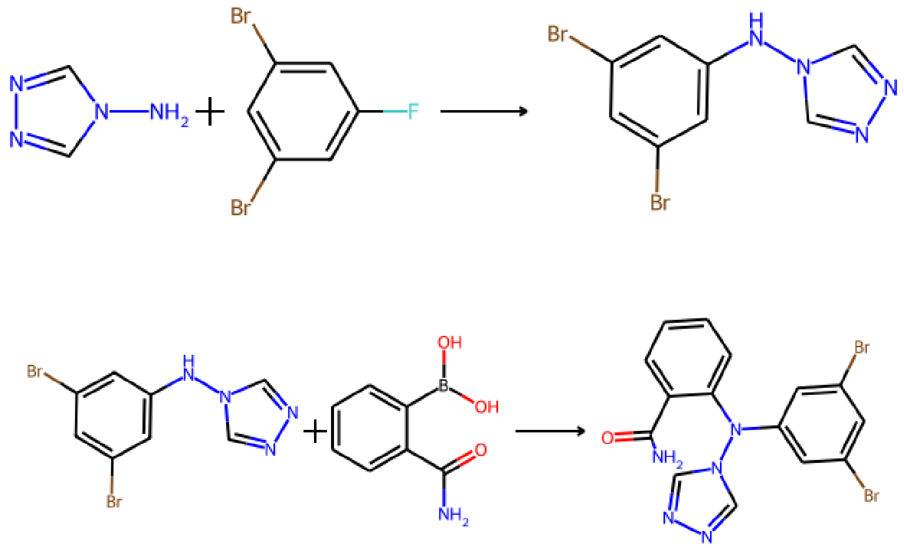

Product 215

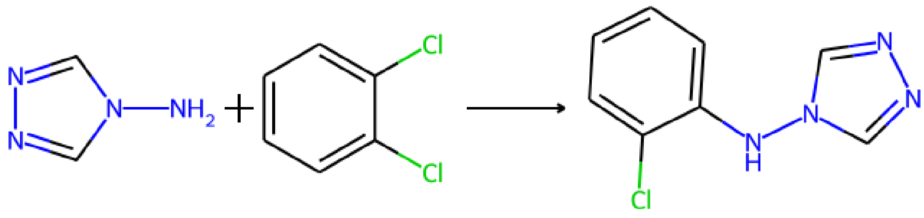

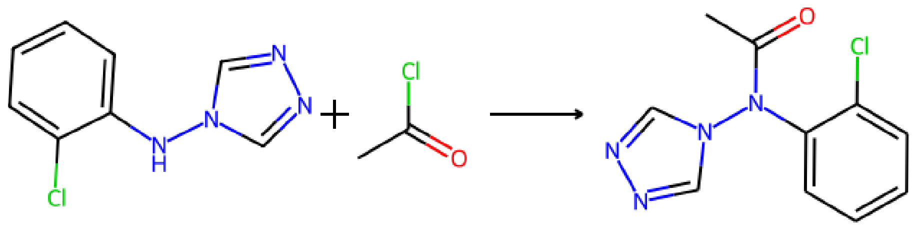

Product 216

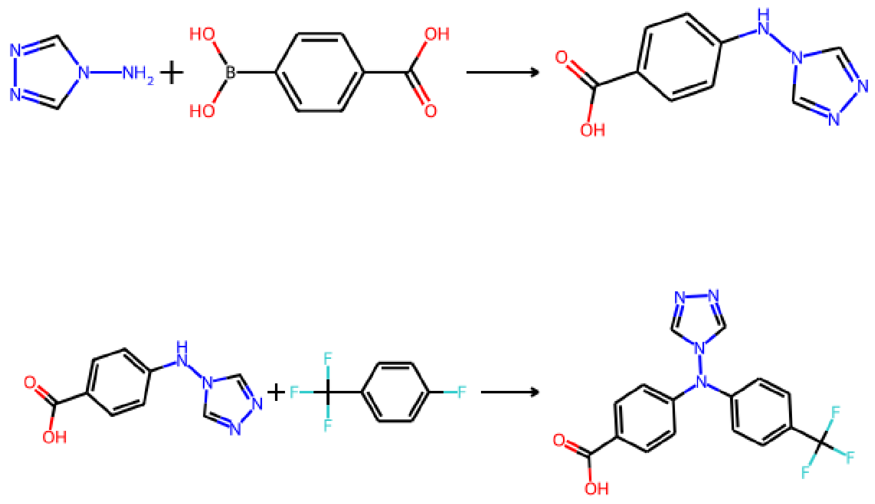

Product 217

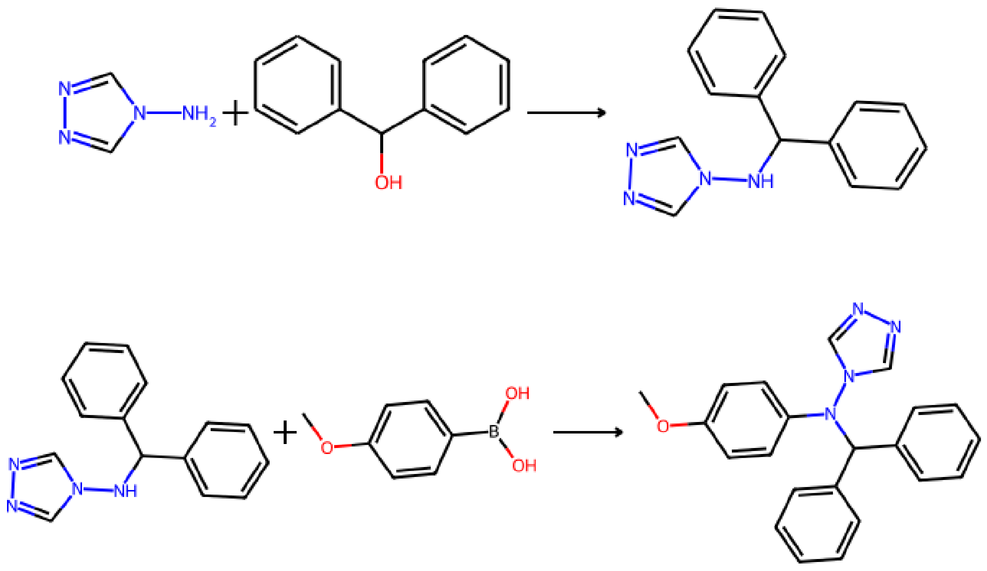

Product 218

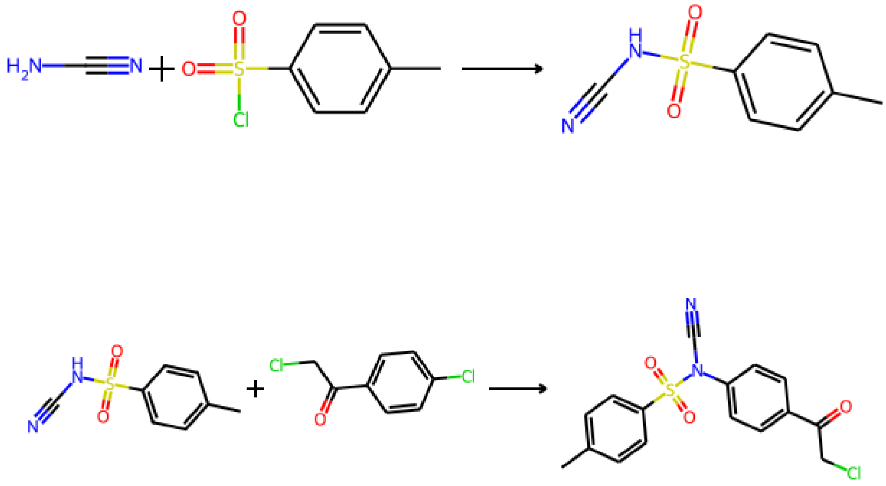

Product 219

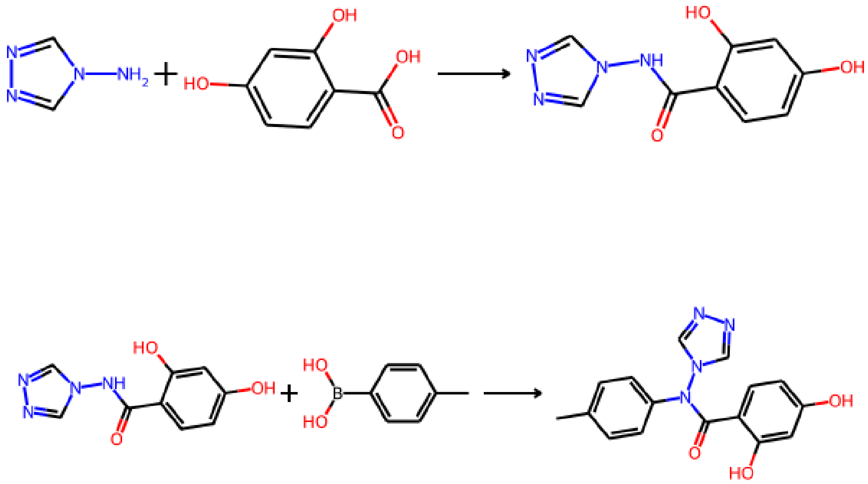

Product 220

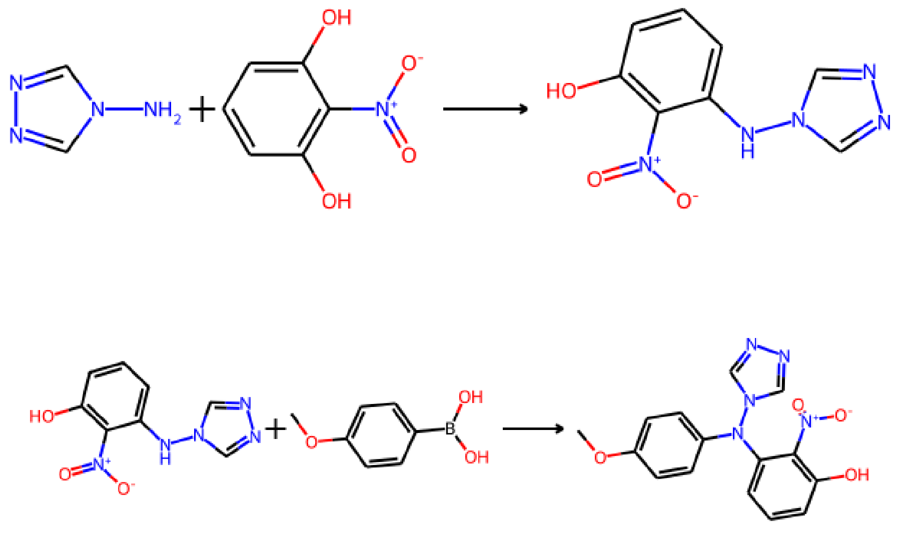

Product 221

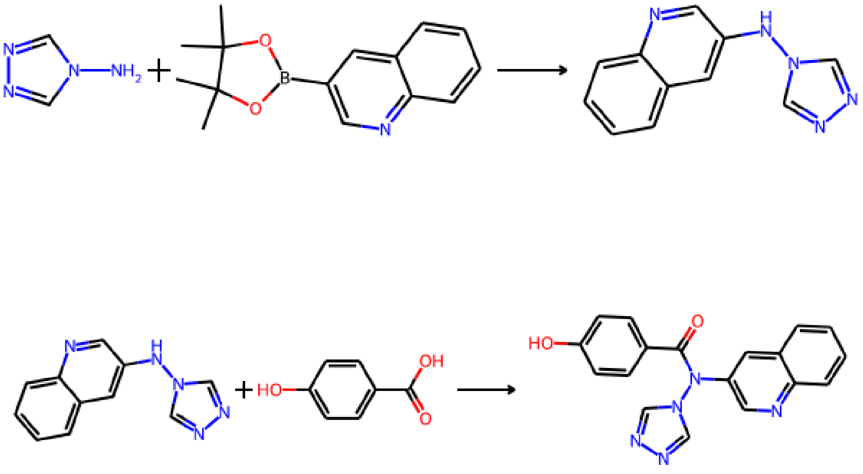

Product 222

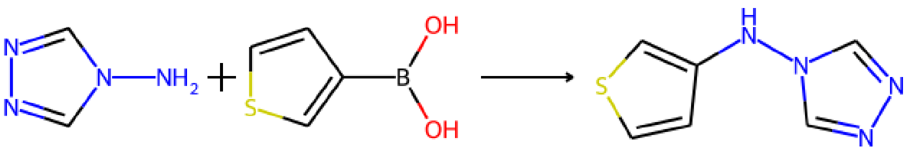

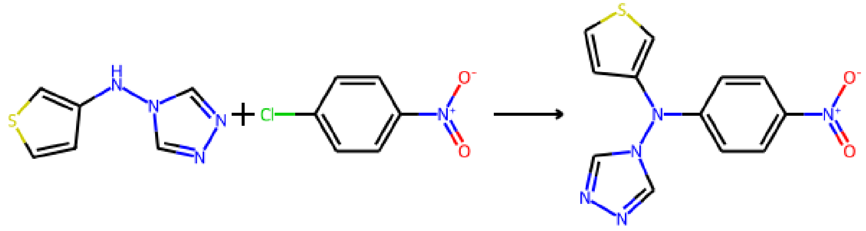

Product 223

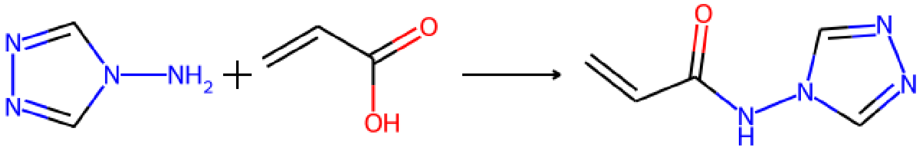

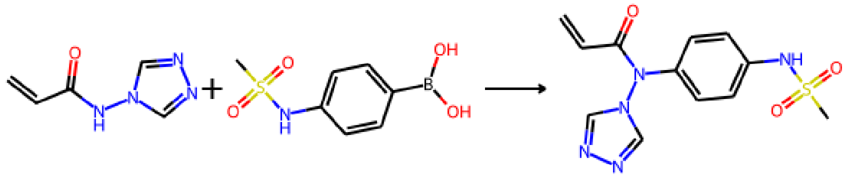

Product 224

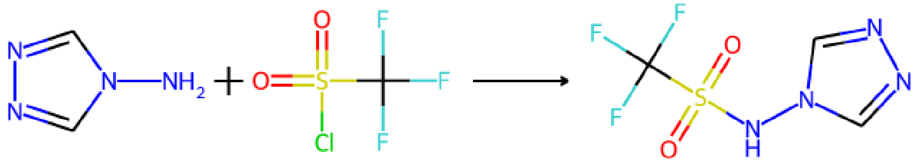

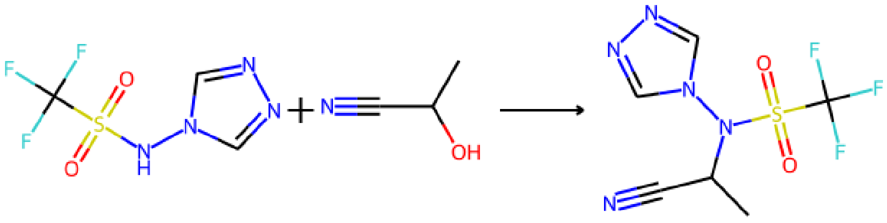

Product 225

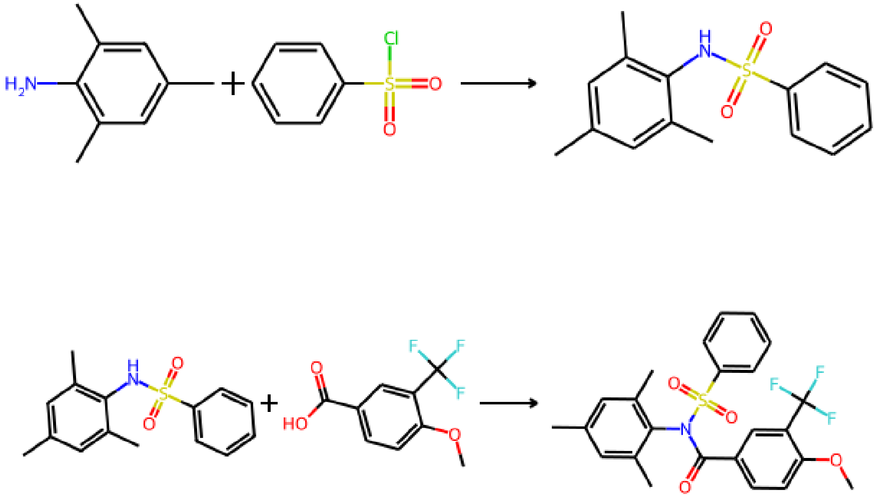

Product 226

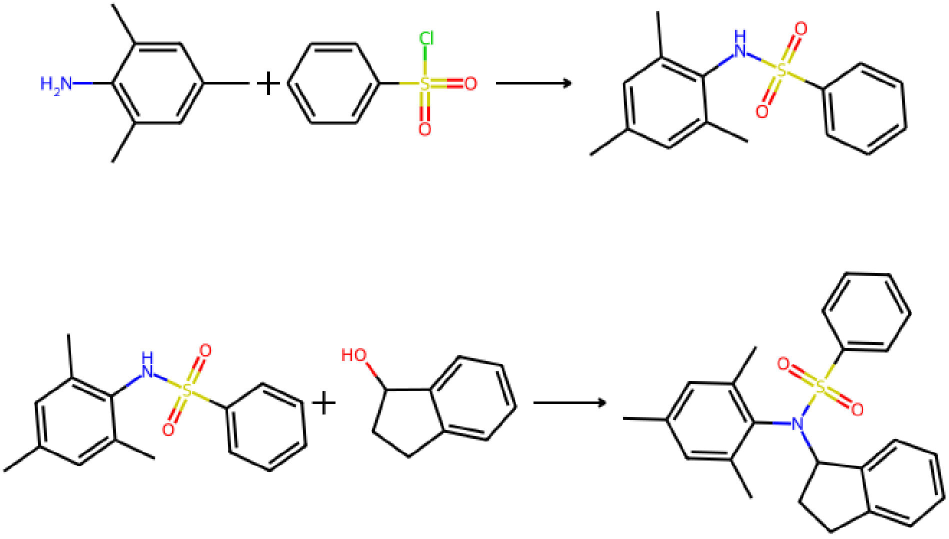

Product 227

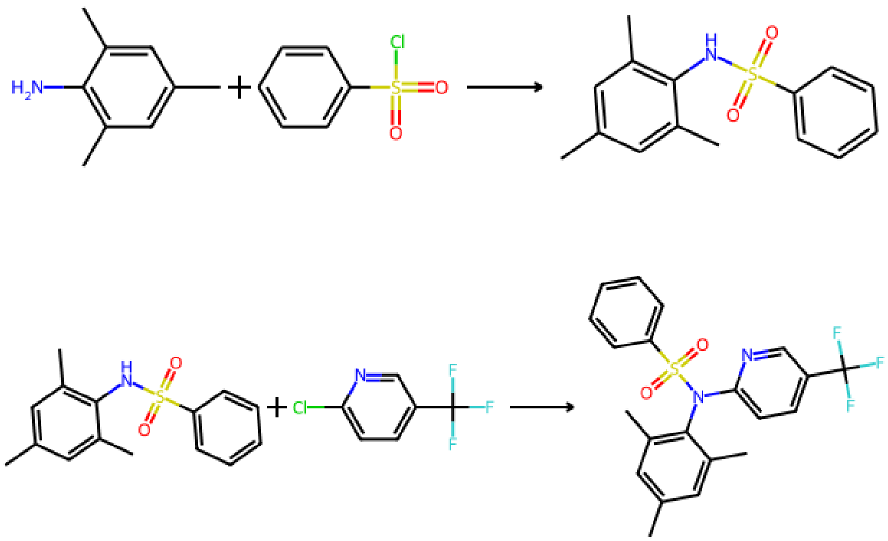

Product 228

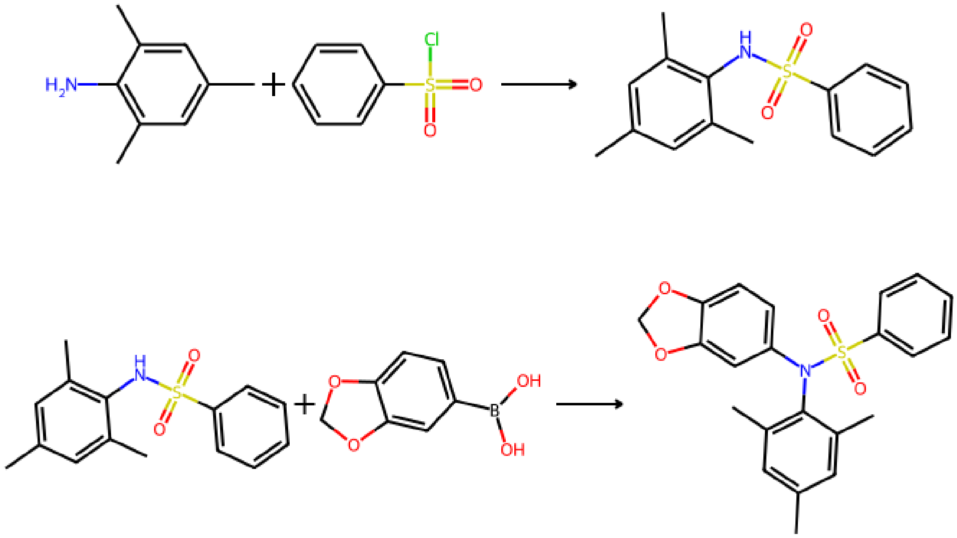

Product 229

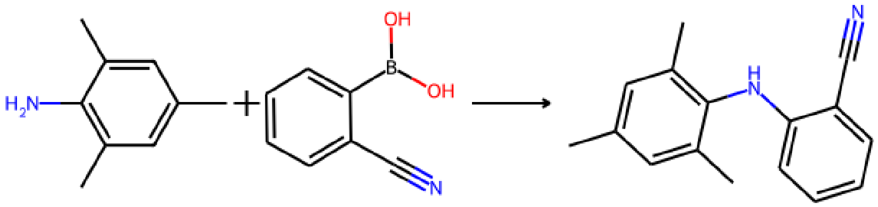

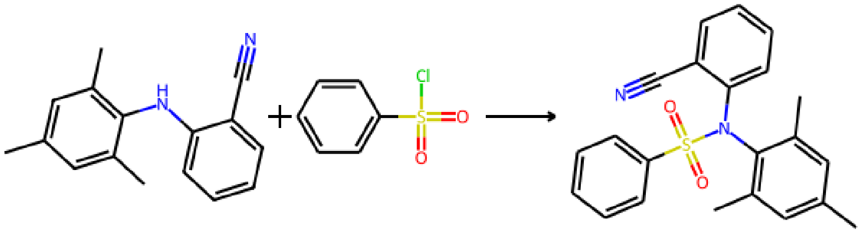

Product 230

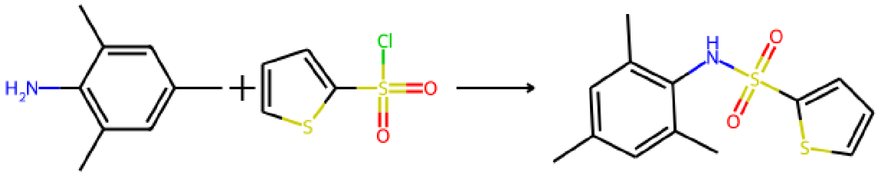

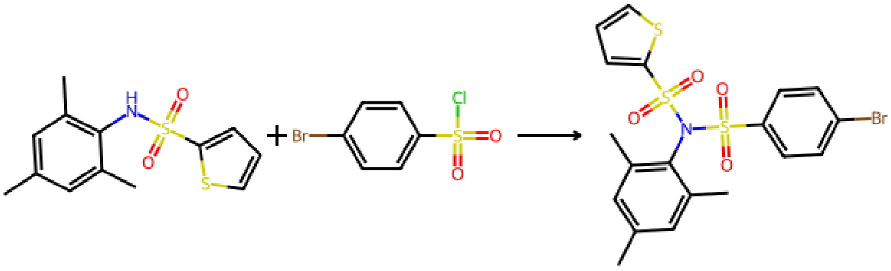

Product 231

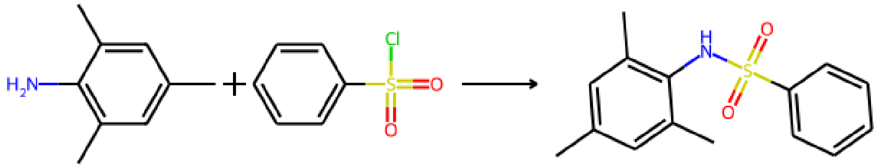

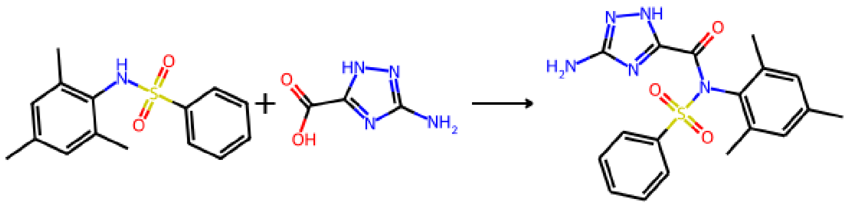

Product 232

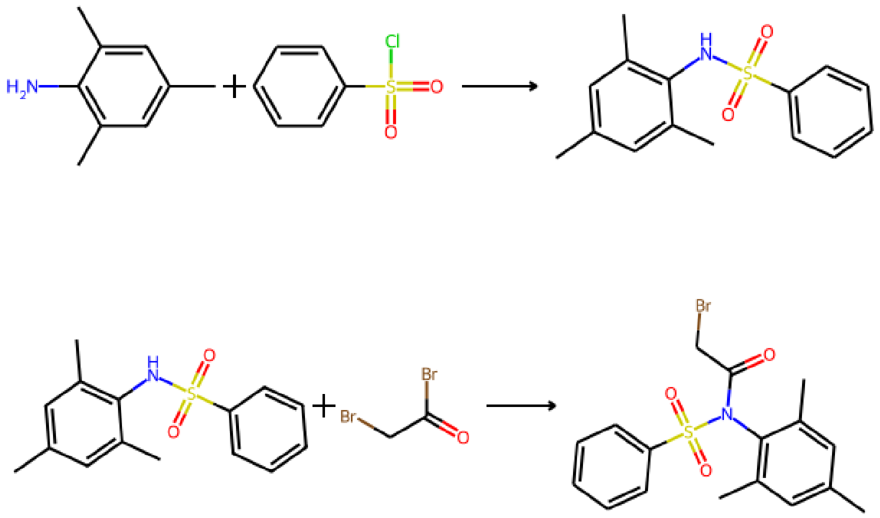

Product 233

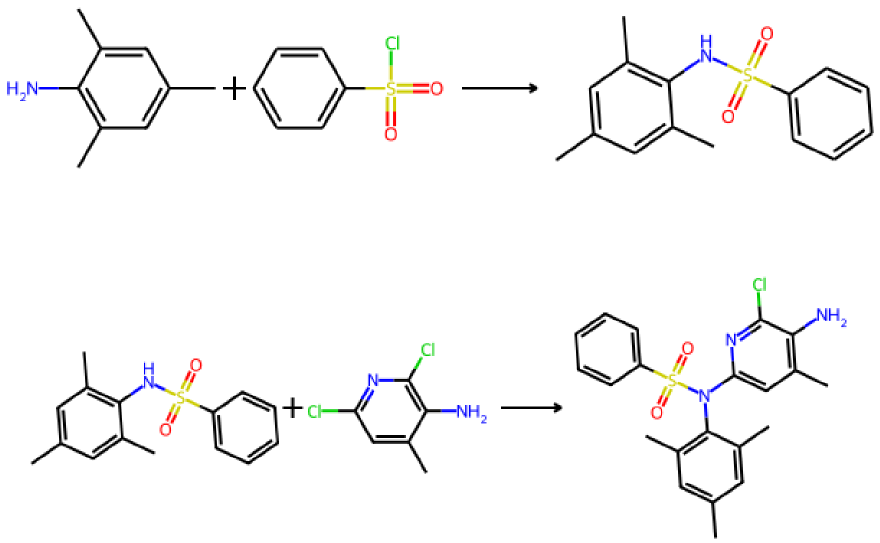

Supplement: Supplementary file 2 — oc4c01991_si_002.pdf [file oc4c01991_si_002.pdf]
