# Supplementary material for: Bayesian Optimization over Multiple Experimental Fidelities Accelerates Automated Discovery of Drug Molecules
Source: ACS Cent Sci. 2025 Feb 5;11(2):346–56. doi: 10.1021/acscentsci.4c01991 (PMC11869128; doi:10.1021/acscentsci.4c01991)

All Synthesis Pathways from iteration 2

Product 1

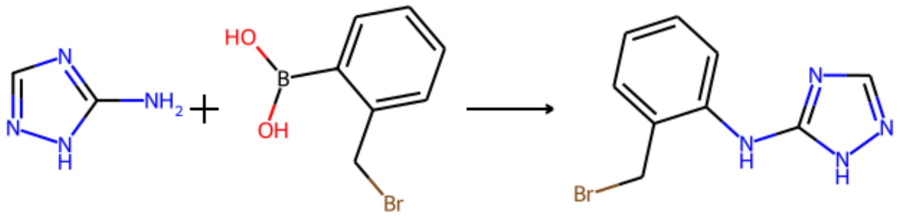

Product 2

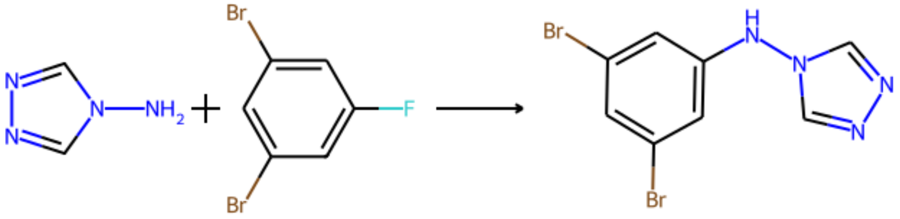

Product 3

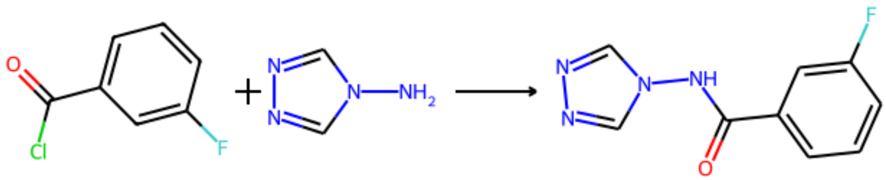

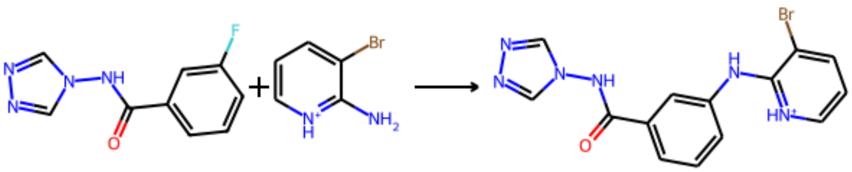

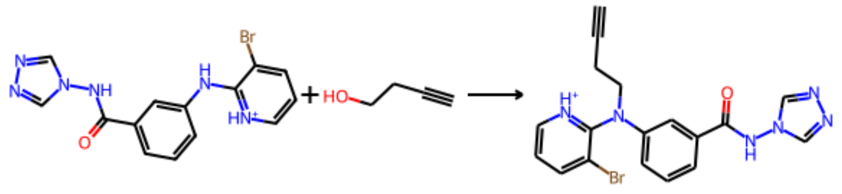

Product 4

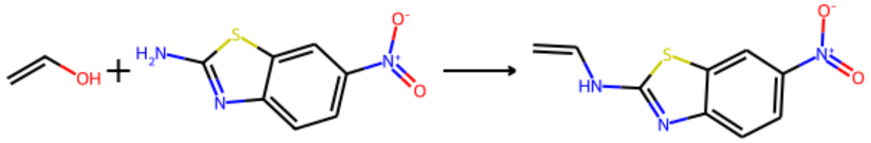

Product 5

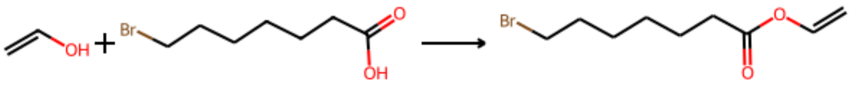

Product 6

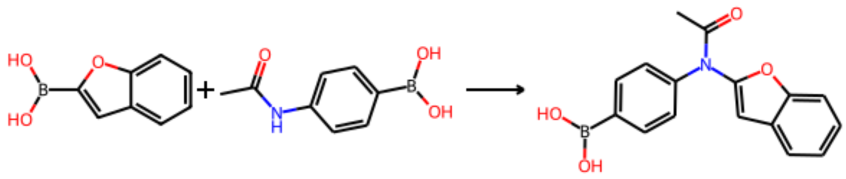

Product 7

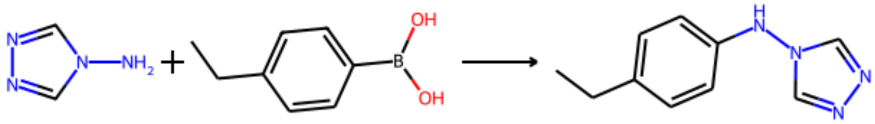

Product 8

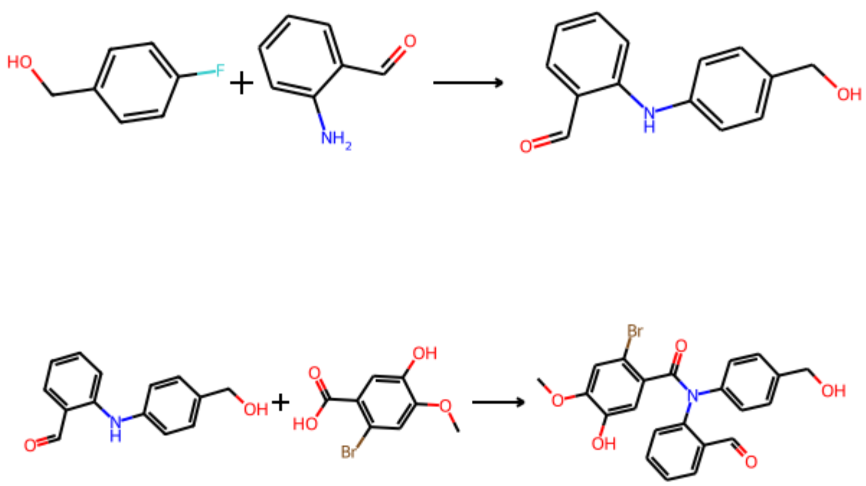

Product 9

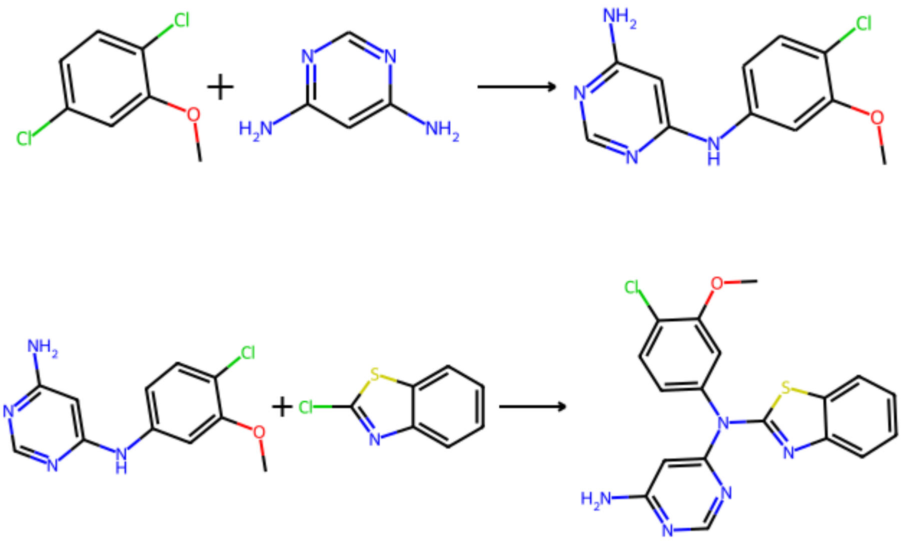

Product 10

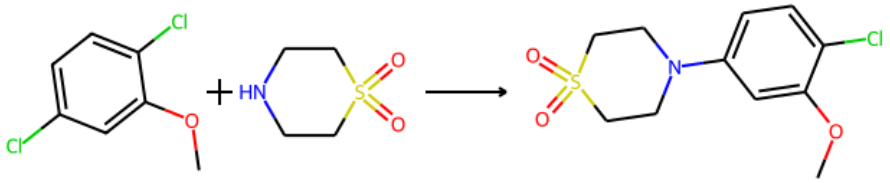

Product 11

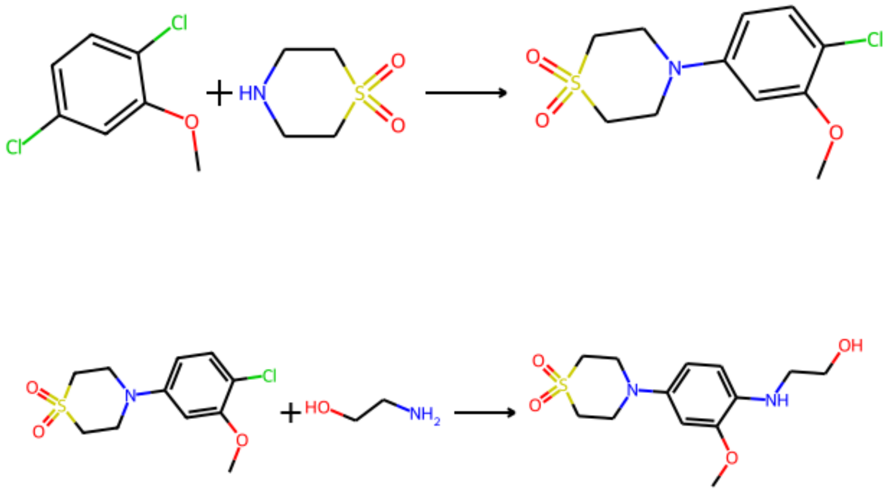

Product 12

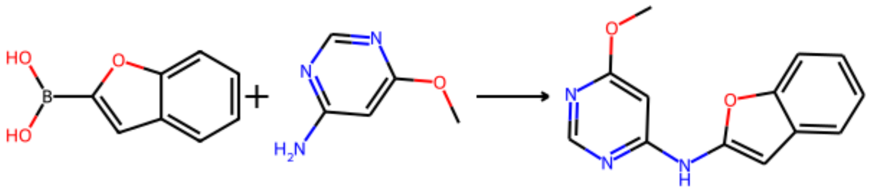

Product 13

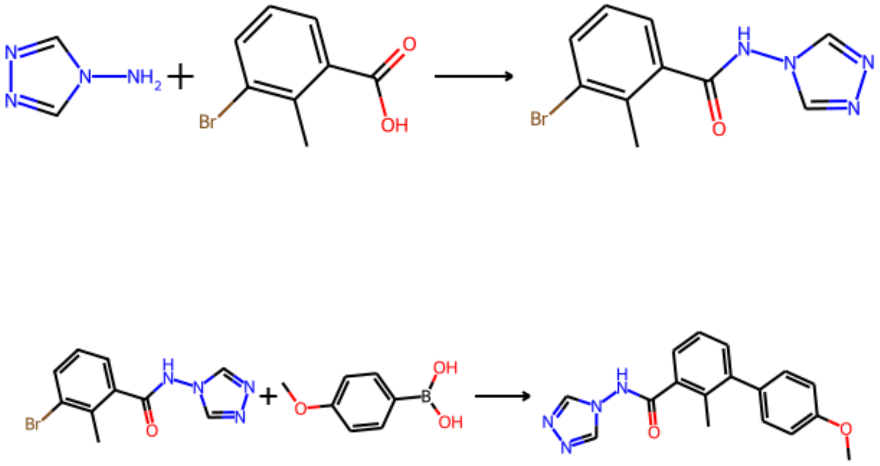

Product 14

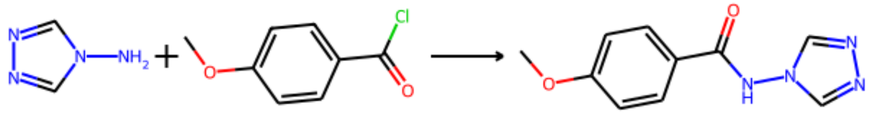

Product 15

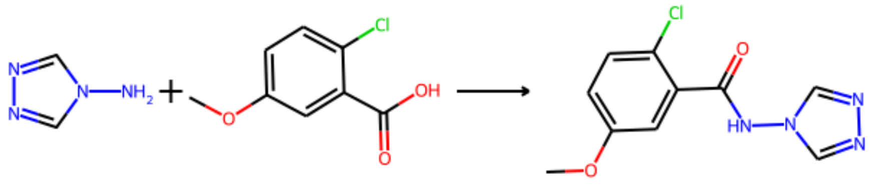

Product 16

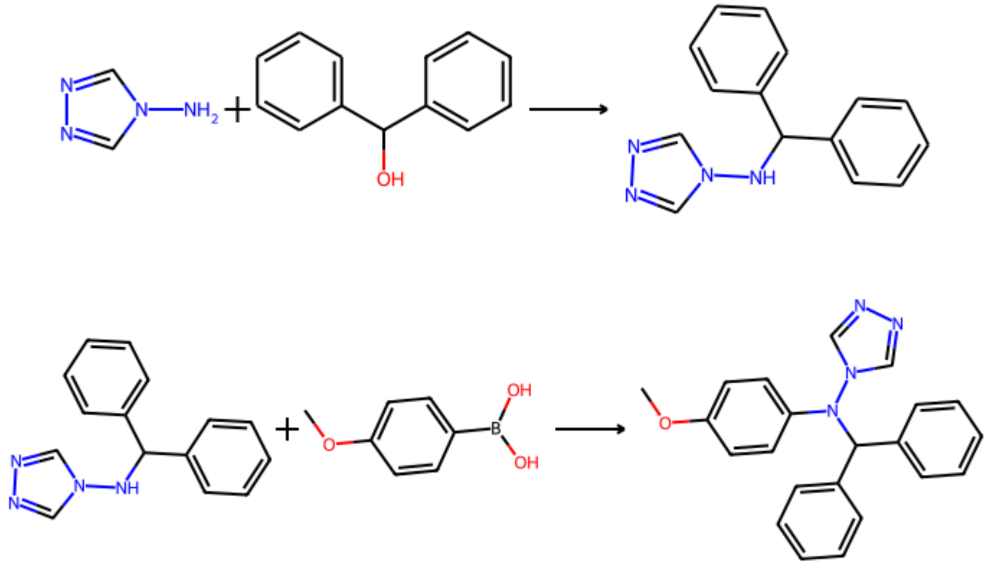

Product 17

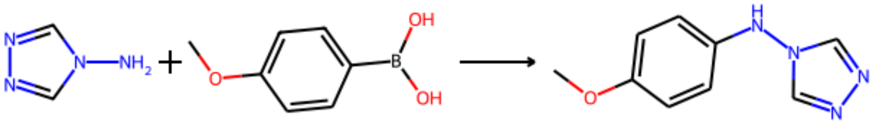

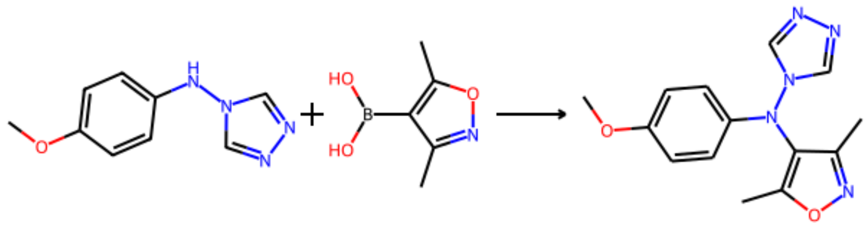

Product 18

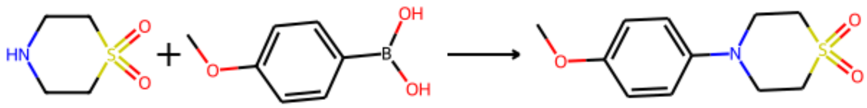

Product 19

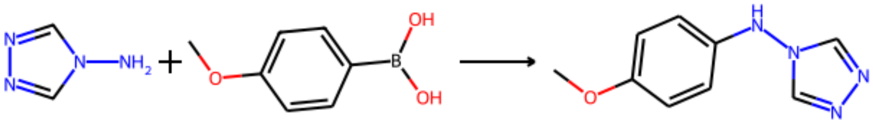

Product 20

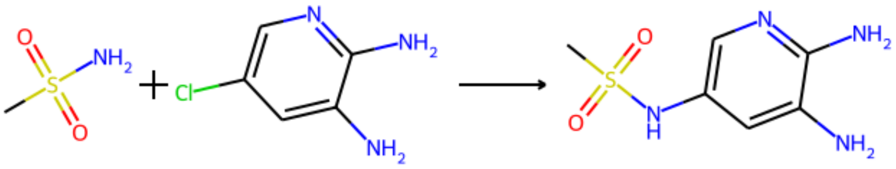

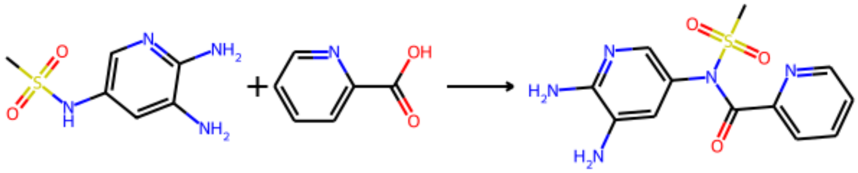

Product 21

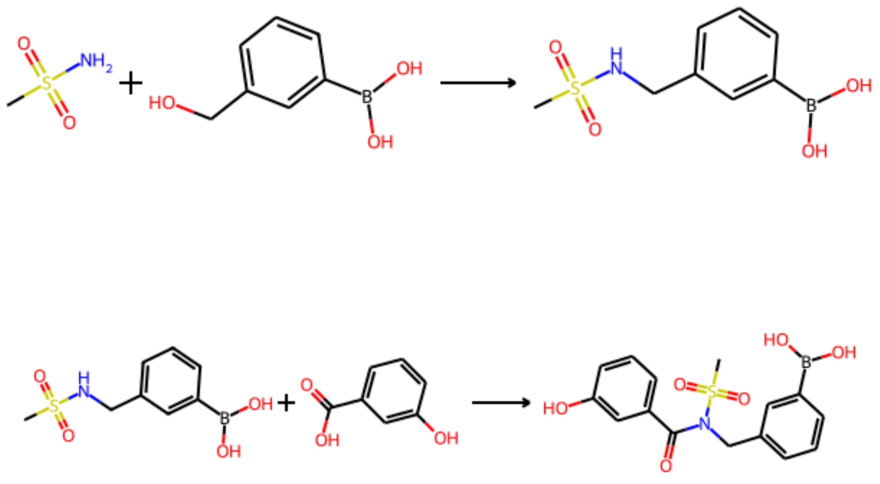

Product 22

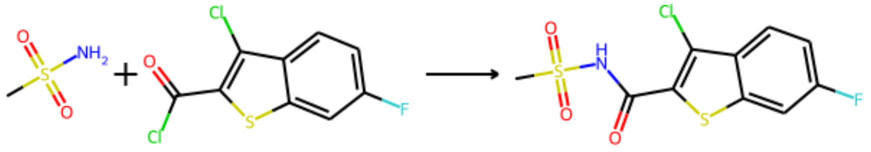

Product 23

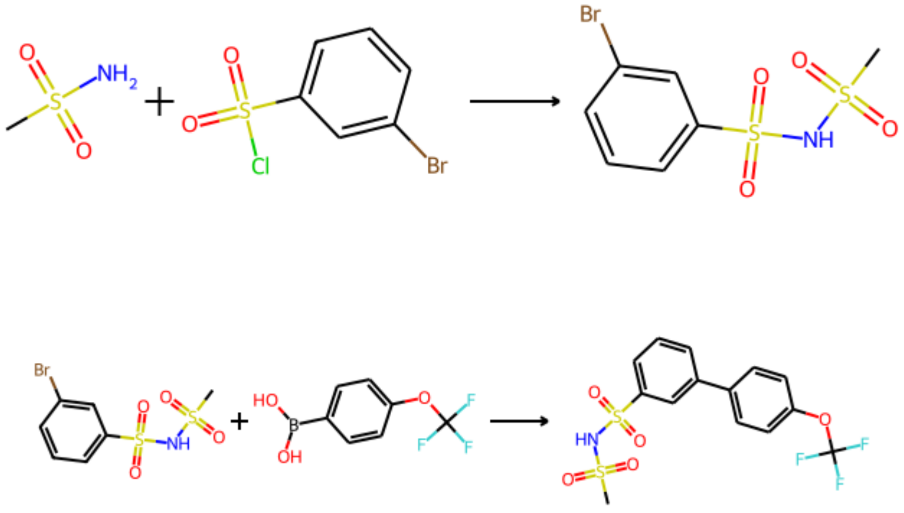

Product 24

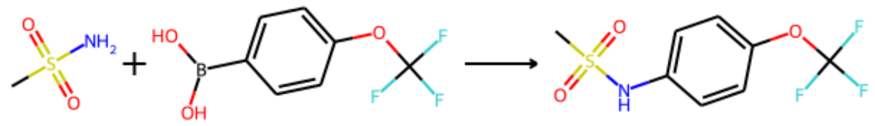

Product 25

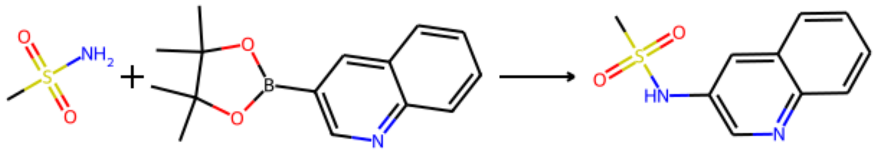

Product 26

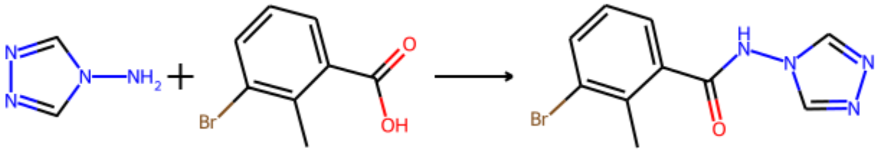

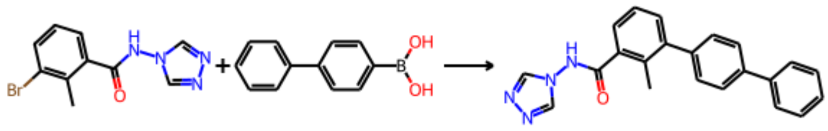

Product 27

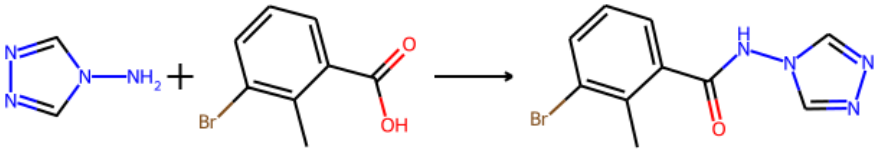

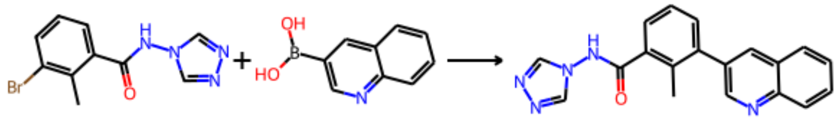

Product 28

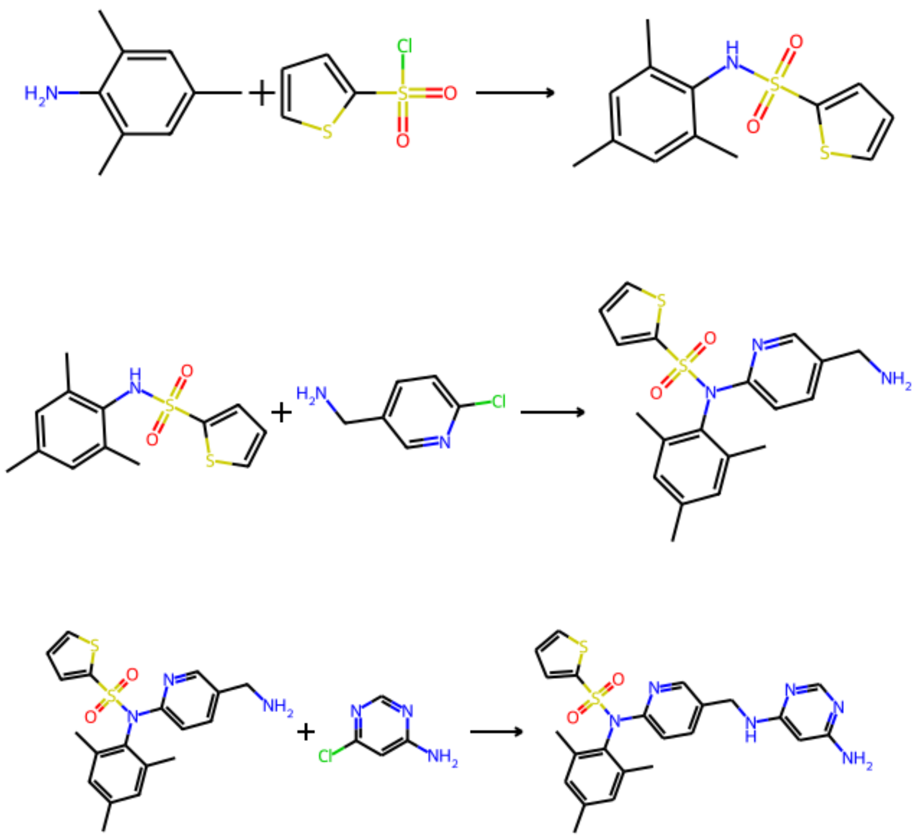

Product 29

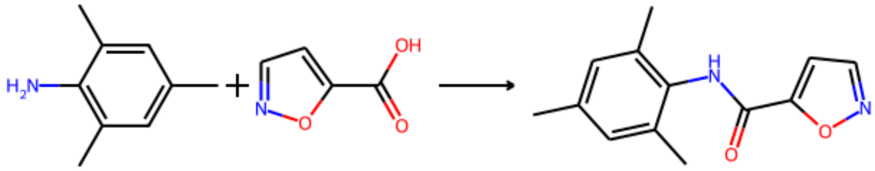

Product 30

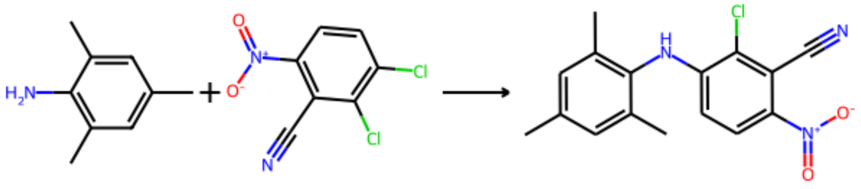

Product 31

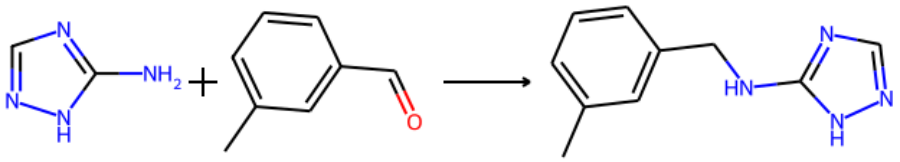

Product 32

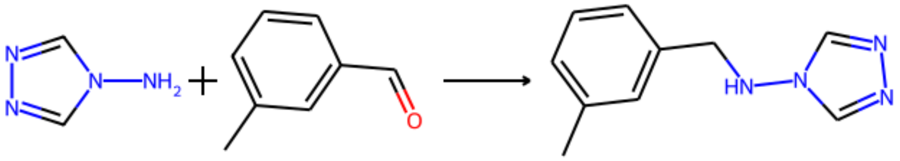

Product 33

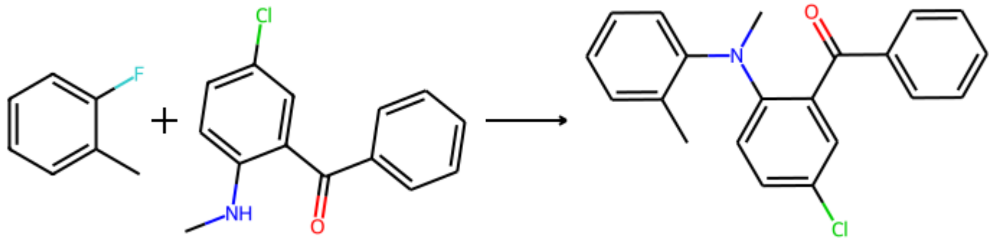

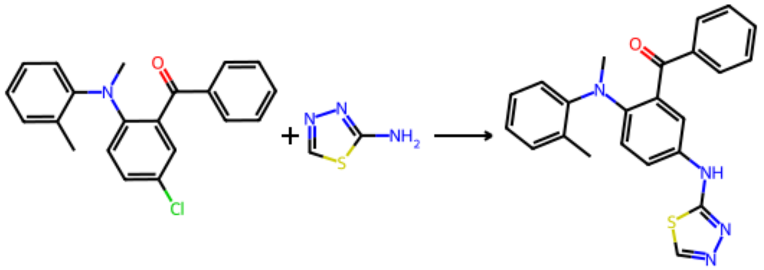

Product 34

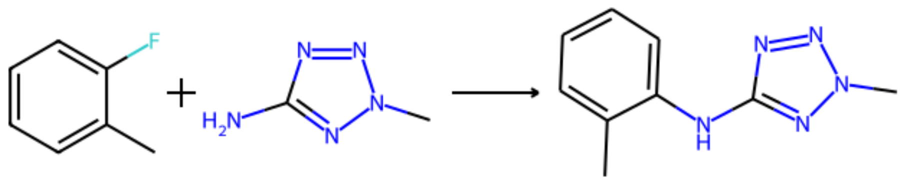

Product 35

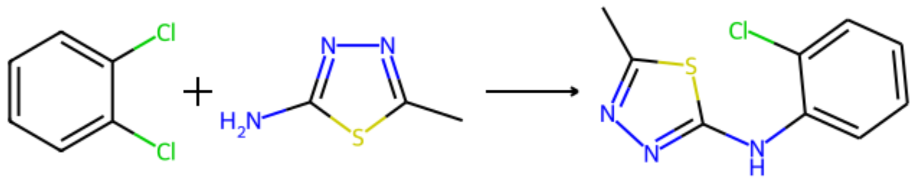

Product 36

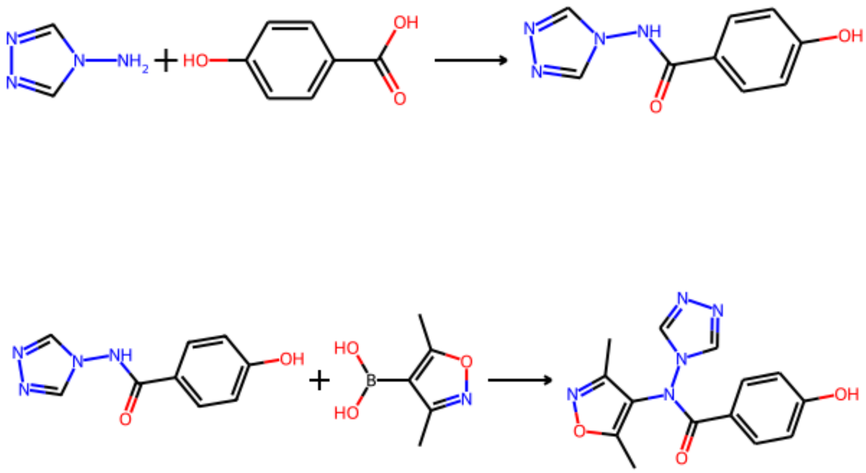

Product 37

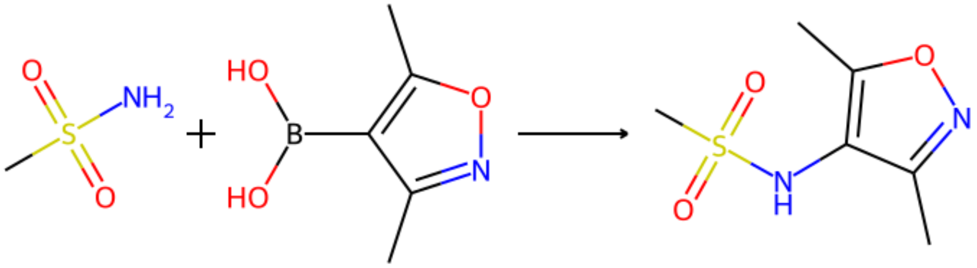

Product 38

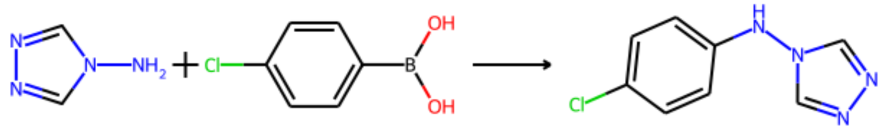

Product 39

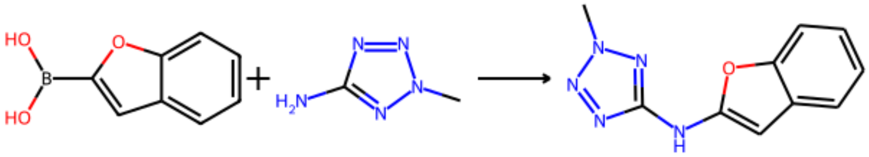

Product 40

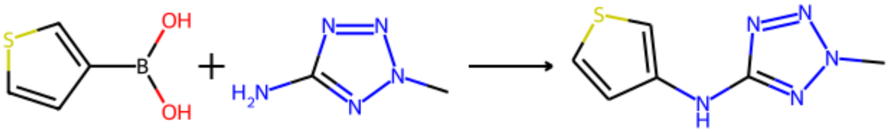

Product 41

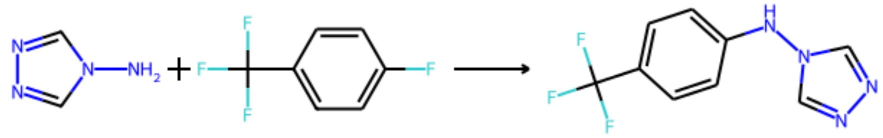

Product 42

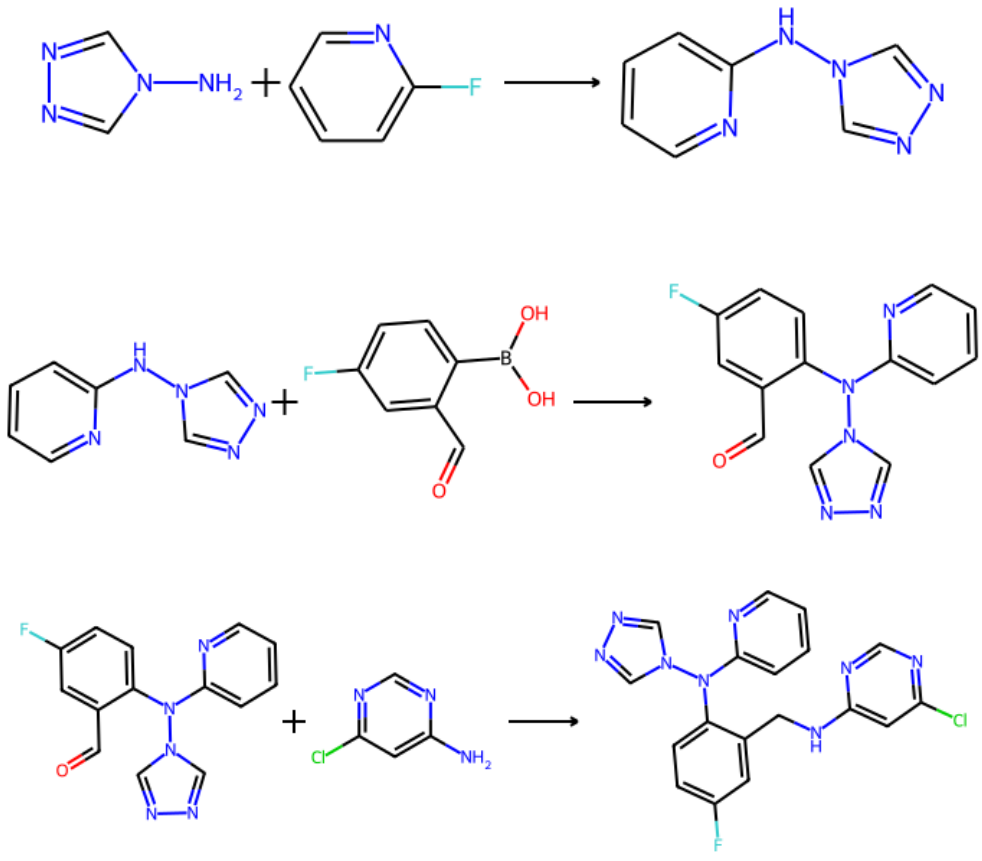

Product 43

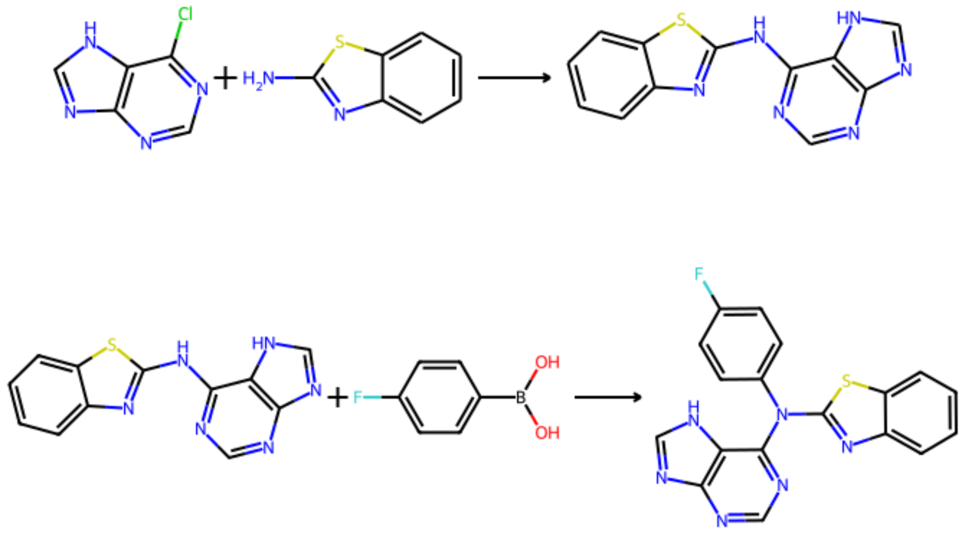

Product 44

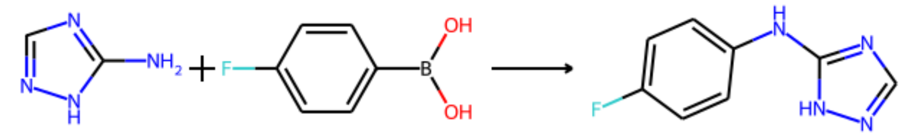

Product 45

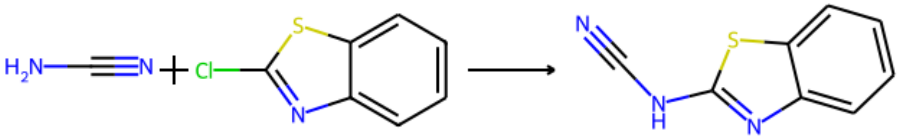

Product 46

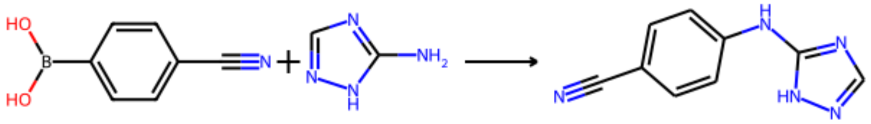

Product 47

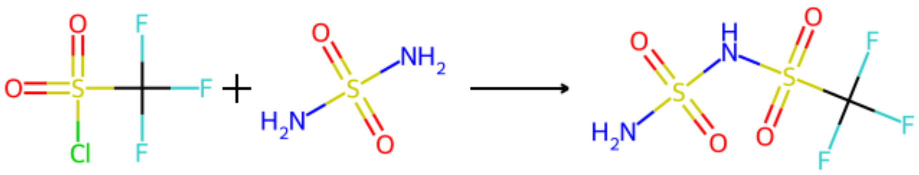

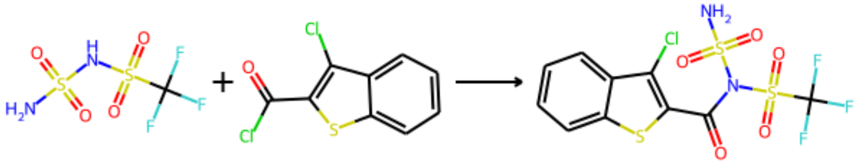

Product 48

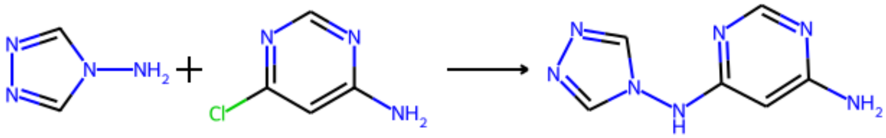

Product 49

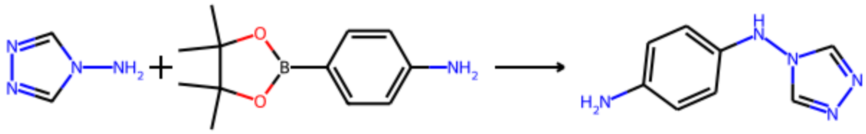

Product 50

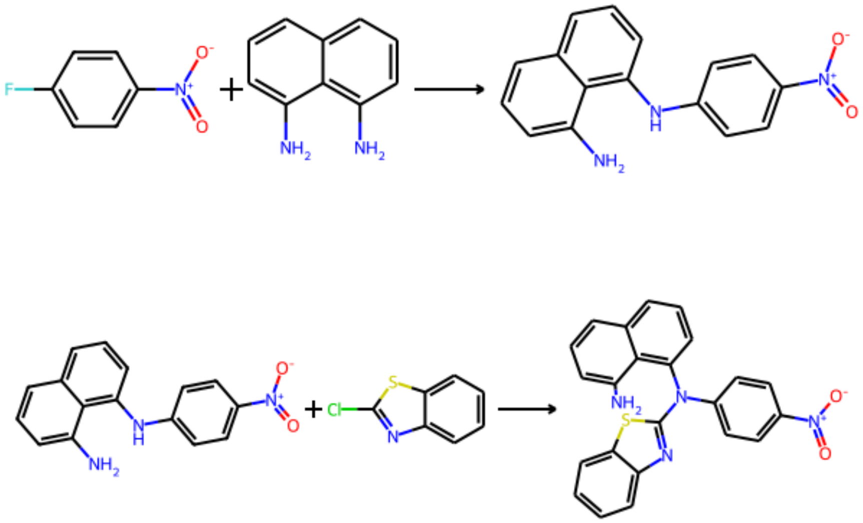

Product 51

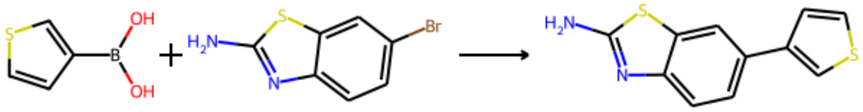

Product 52

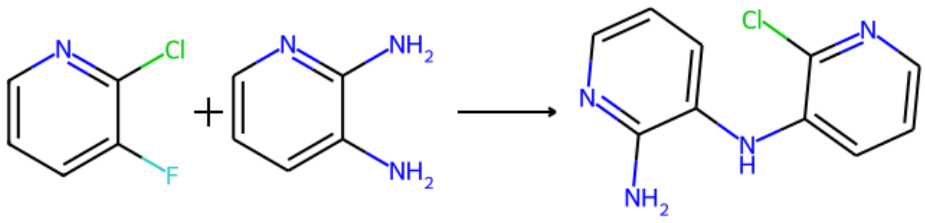

Product 53

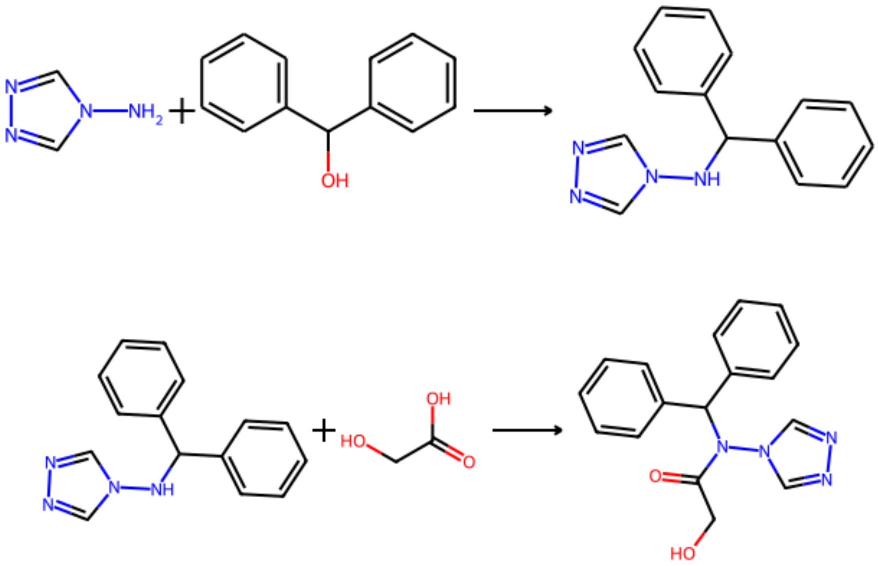

Product 54

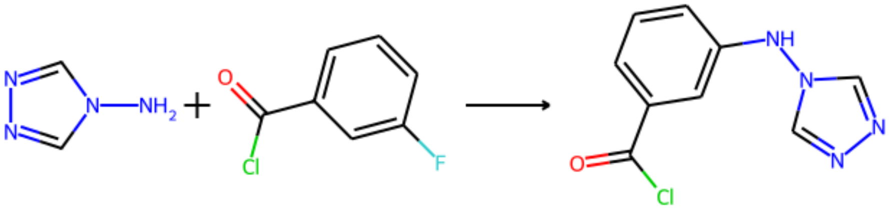

Product 55

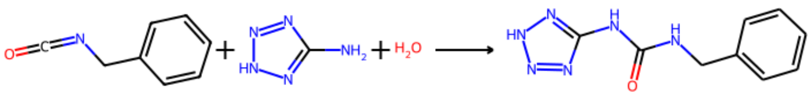

Product 56

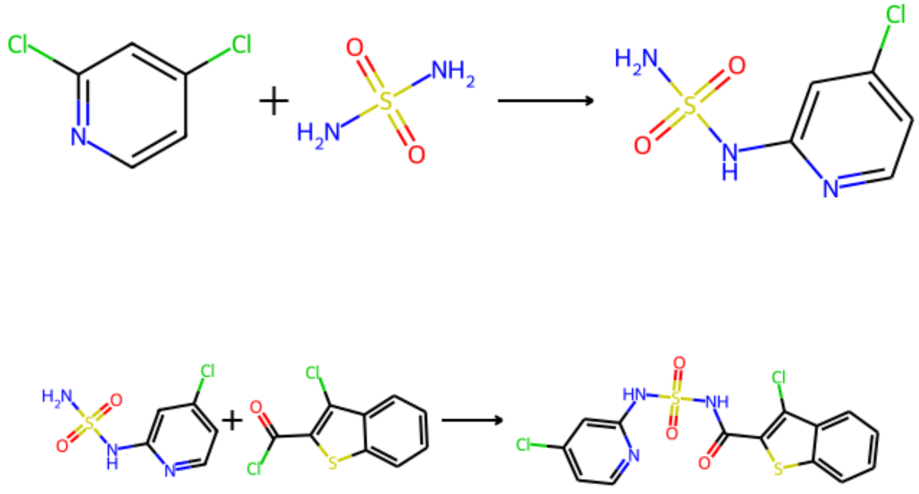

Product 57

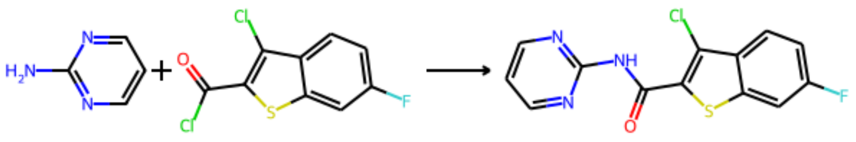

Product 58

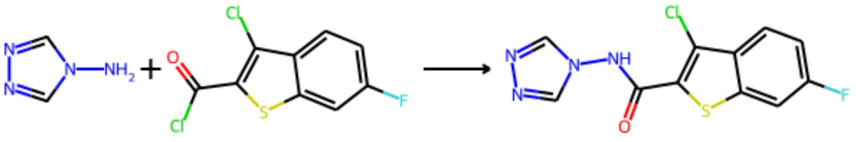

Product 59

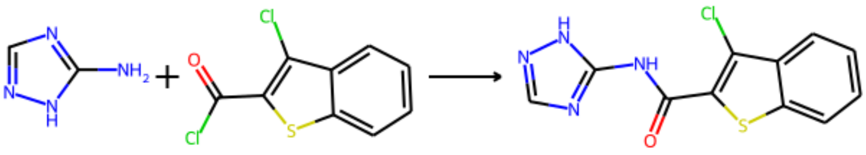

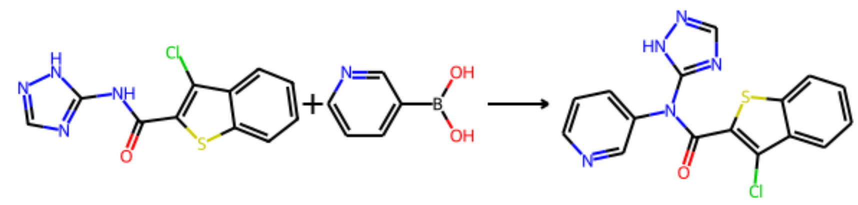

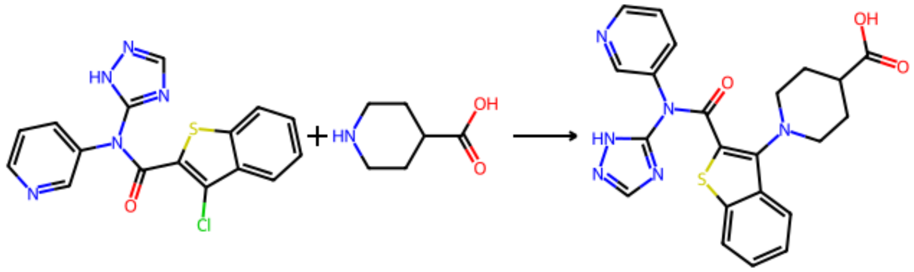

Product 60

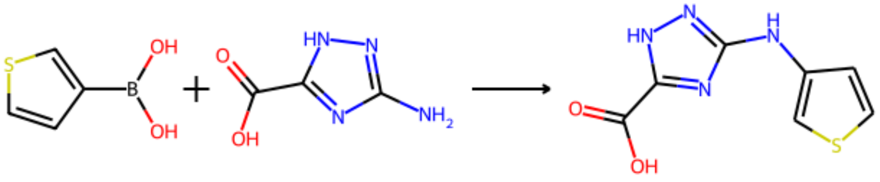

Product 61

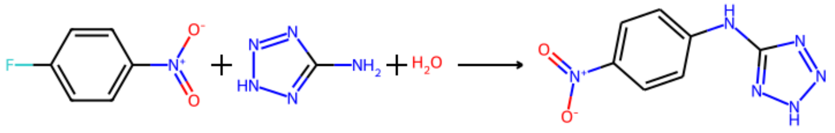

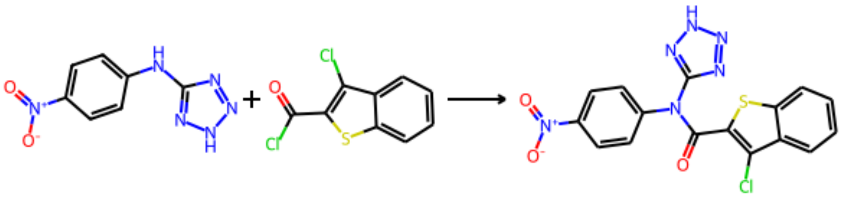

Product 62

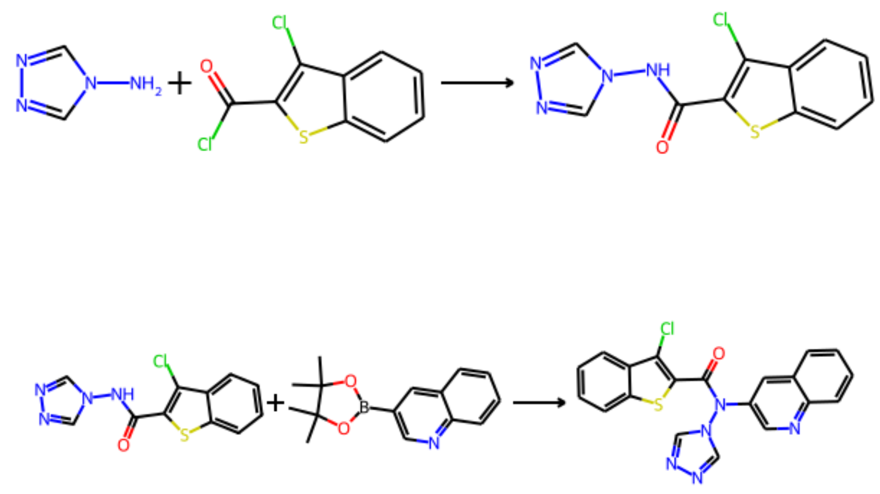

Product 63

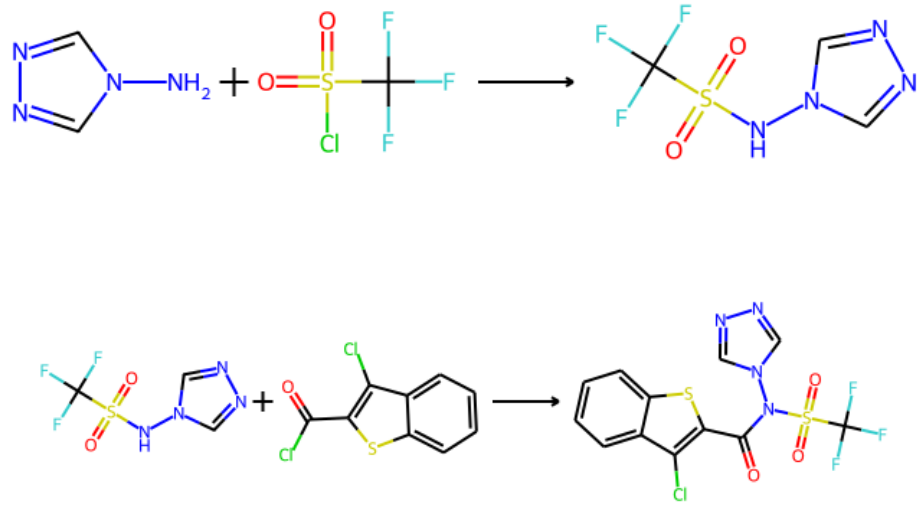

Product 64

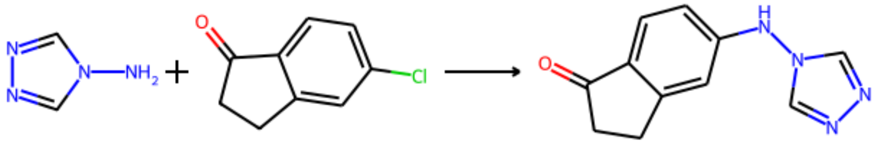

Product 65

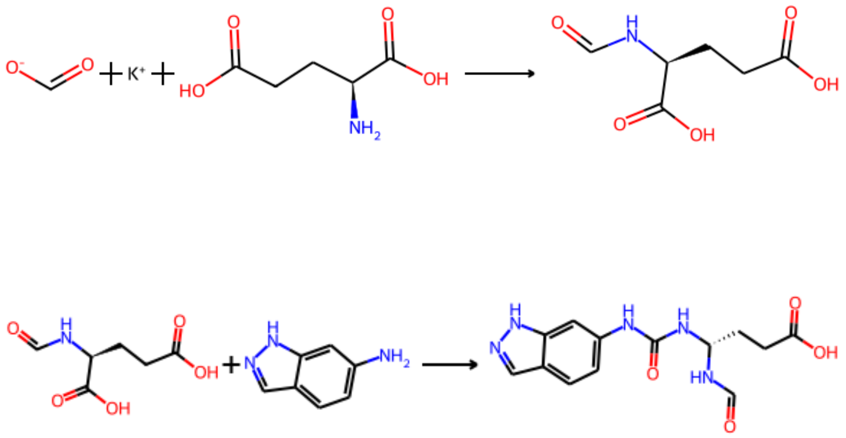

Product 66

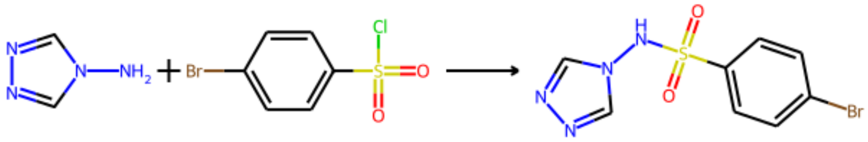

Product 67

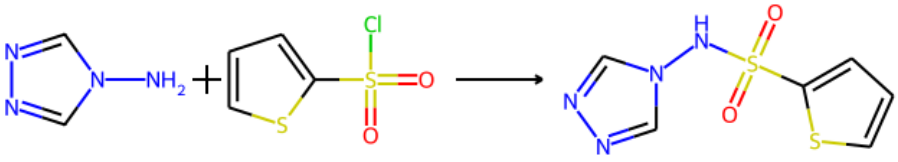

Product 68

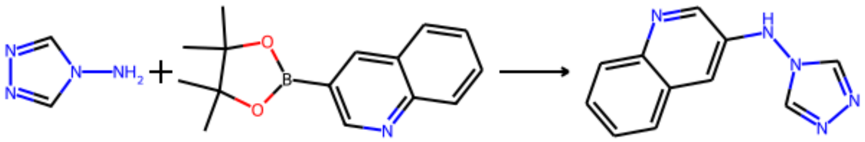

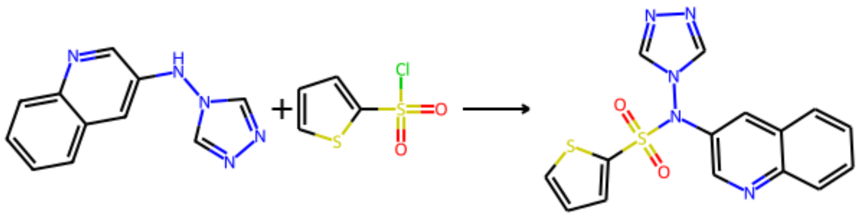

Product 69

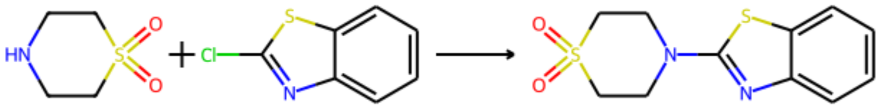

Product 70

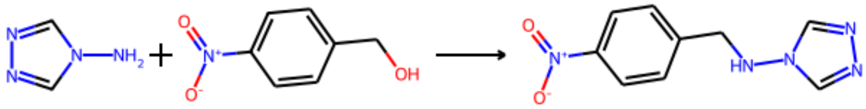

Product 71

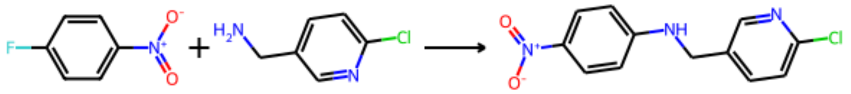

Product 72

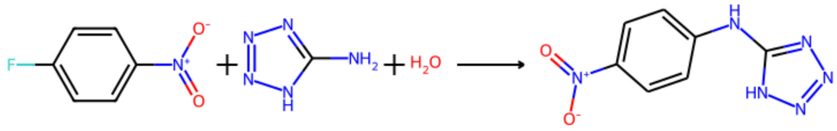

Product 73

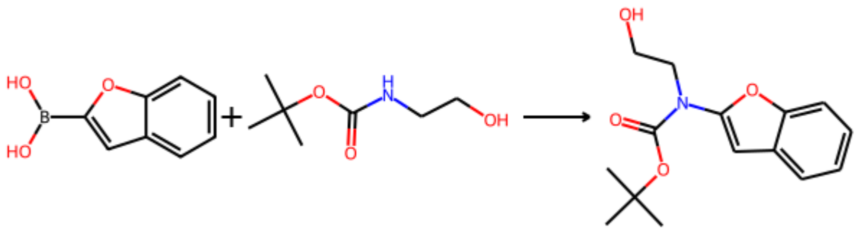

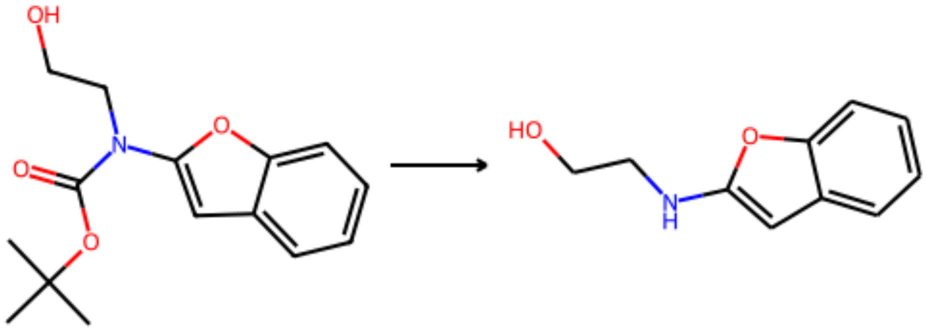

Product 74

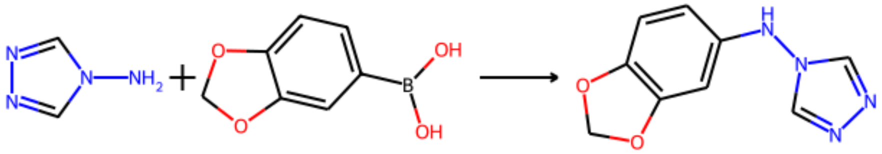

Product 75

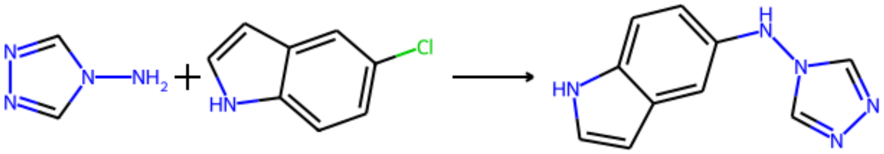

Product 76

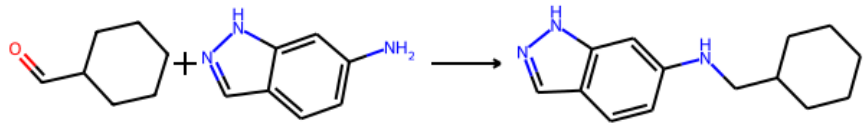

Product 77

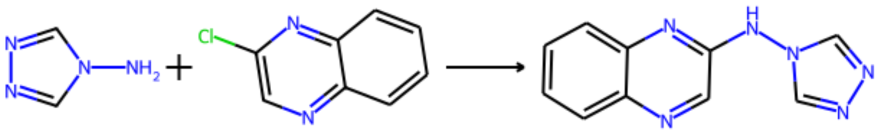

Product 78

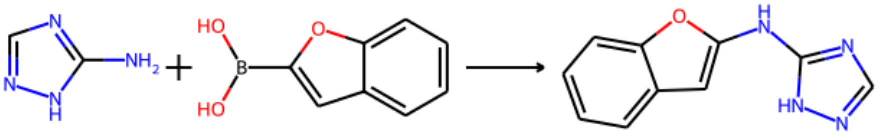

Product 79

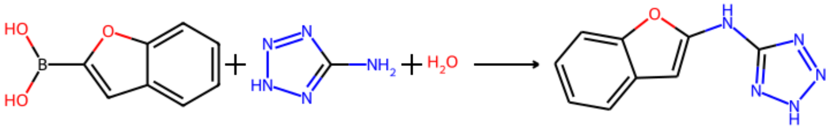

Product 80

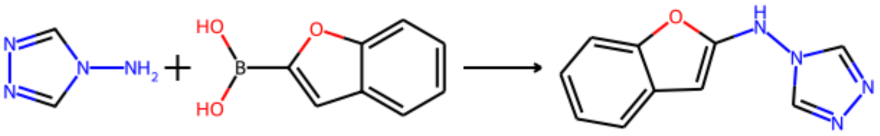

Product 81

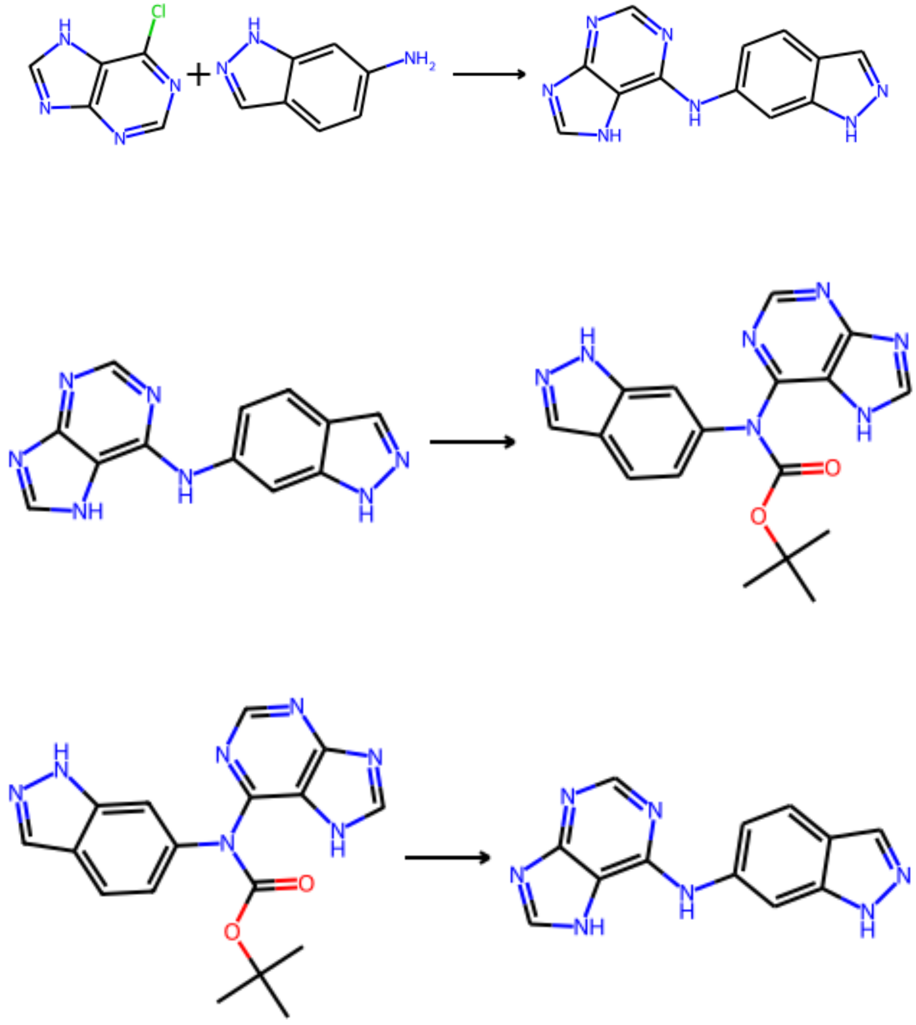

Product 82

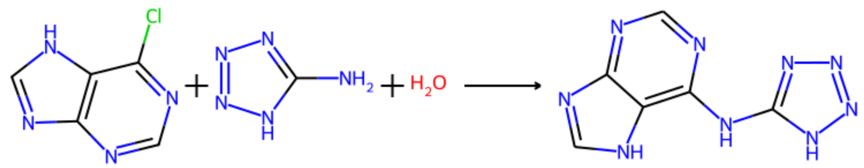

Product 83

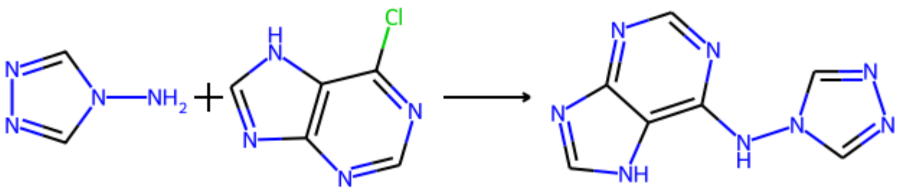

Product 84

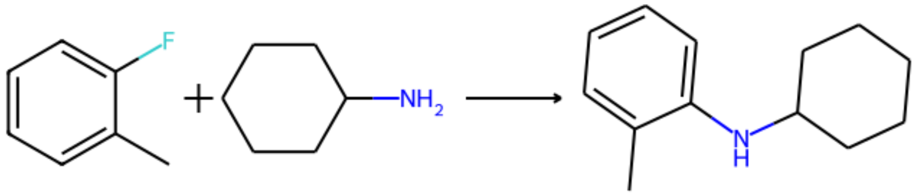

Product 85

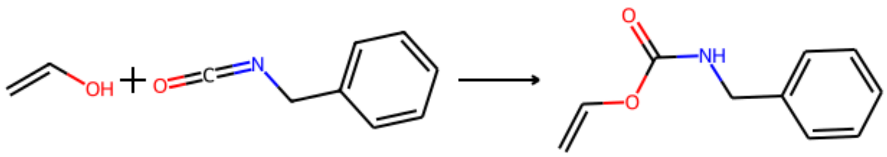

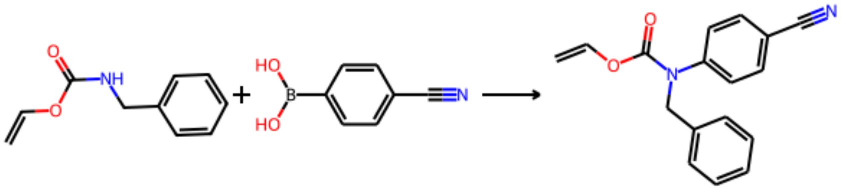

Product 86

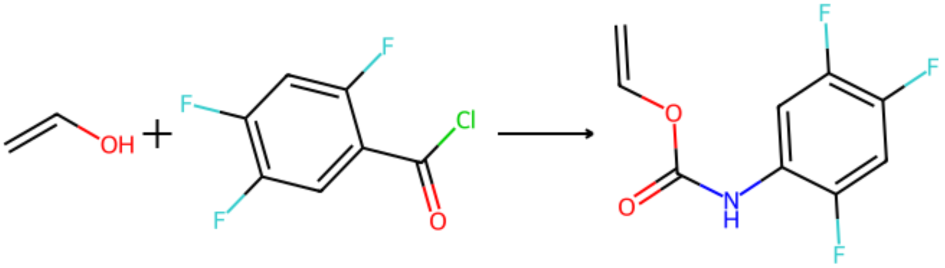

Product 87

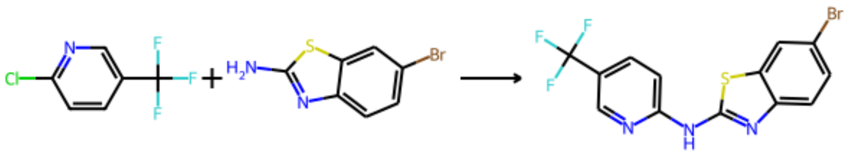

Product 88

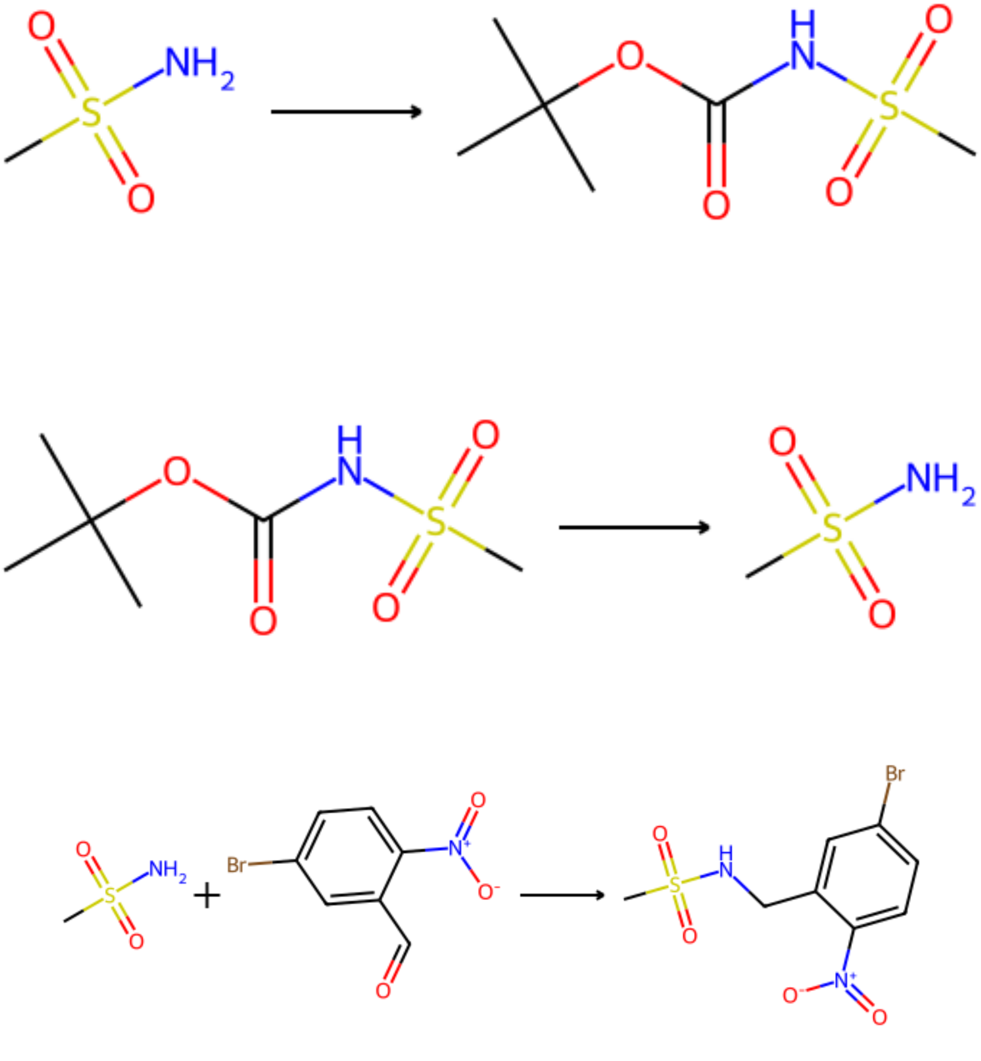

Product 89

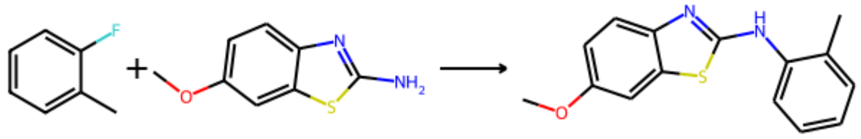

Product 90

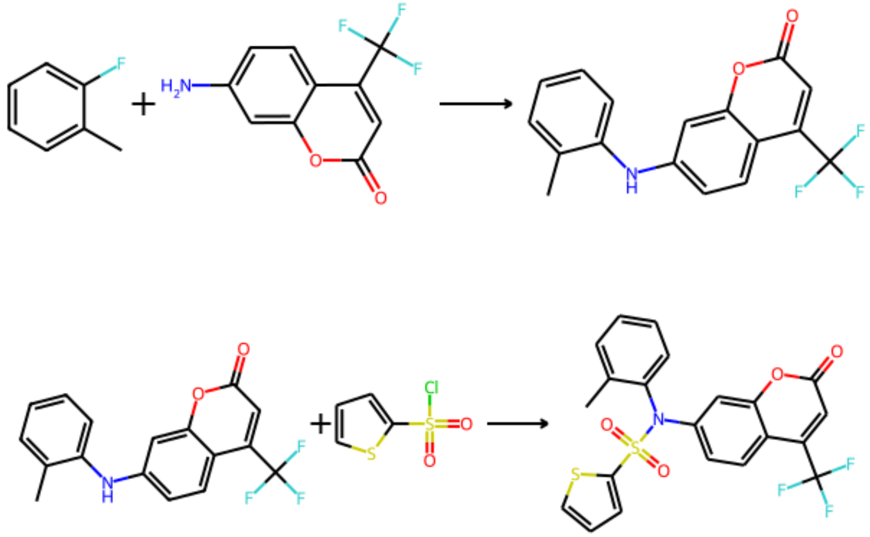

Product 91

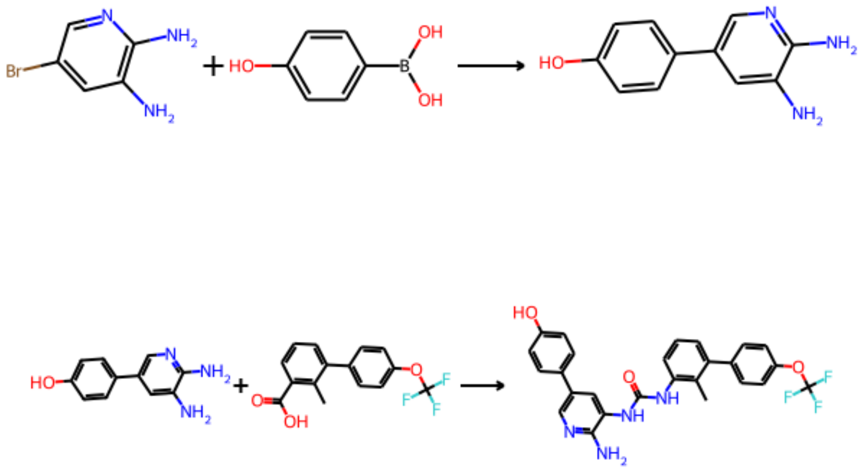

Product 92

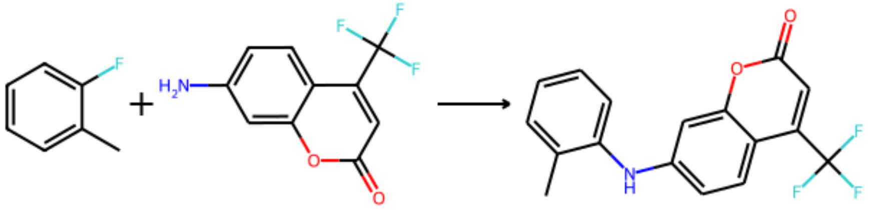

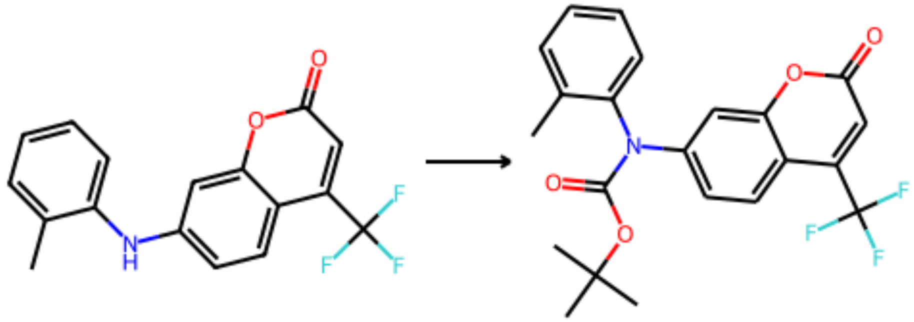

Product 93

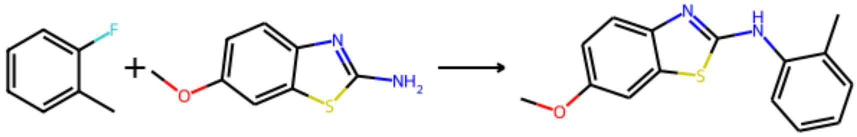

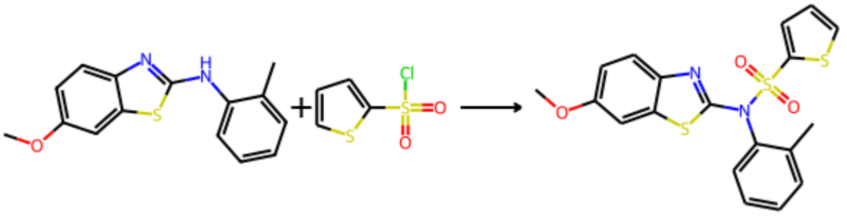

Product 94

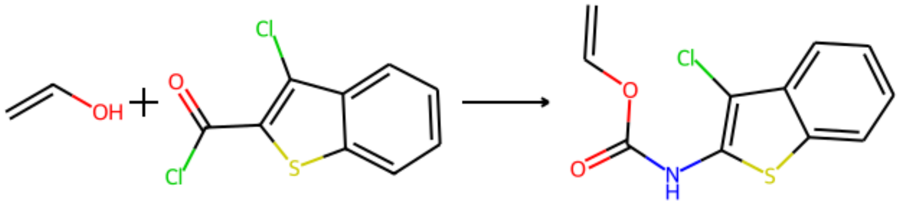

Product 95

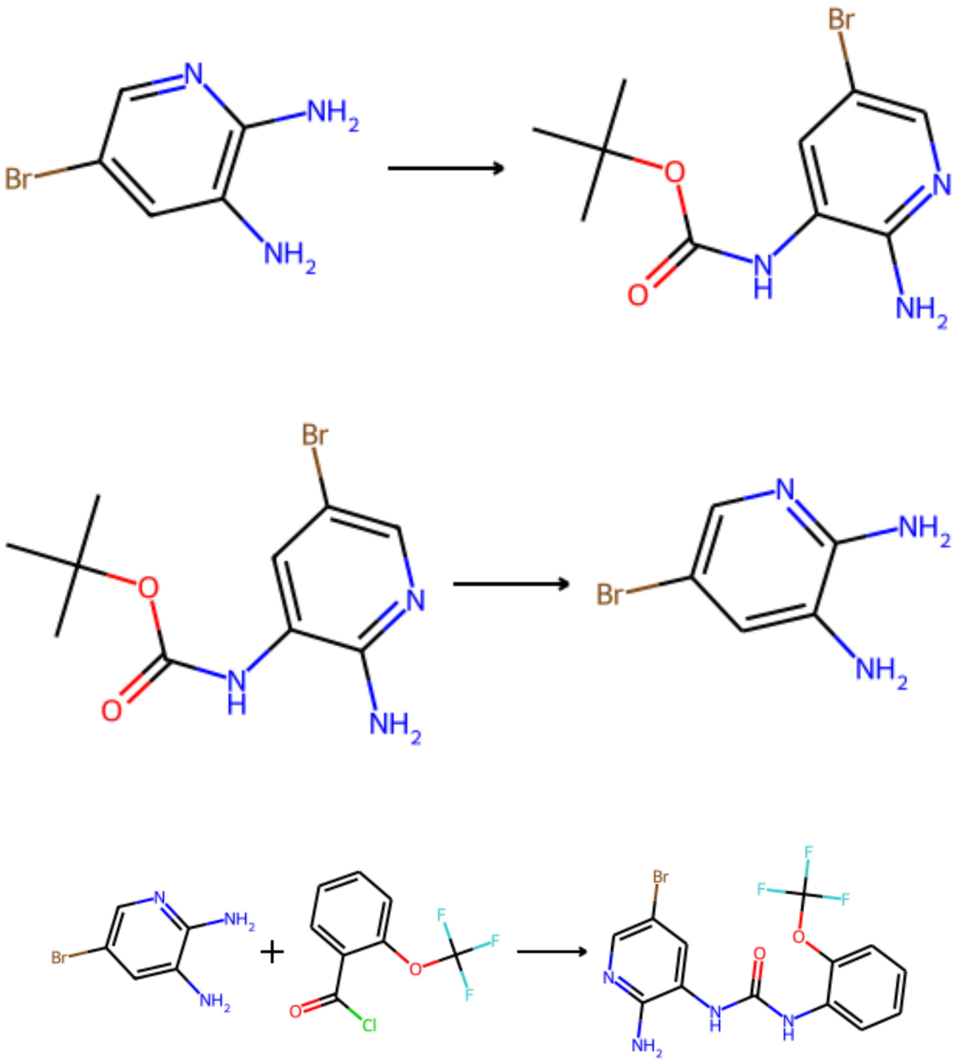

Product 96

C1=C(C=NC(=C1N)N)Br.OB(O)c1ccc(Cl)cc1>>Nc1cc(-c2ccc(Cl)cc2)cnc1N

Nc1cc(-c2ccc(Cl)cc2)cnc1N.CCC/C=C/C=O>>CCC/C=C/CNc1cc(-c2ccc(Cl)cc2)cnc1N

CCC/C=C/CNc1cc(-
c2ccc(Cl)cc2)cnc1N.COc1ccc(B(O)O)cc1OC>>CCC/C=C/CN(c1ccc(OC)c(OC)c1)c1cc(-
c2ccc(Cl)cc2)cnc1N

Product 97

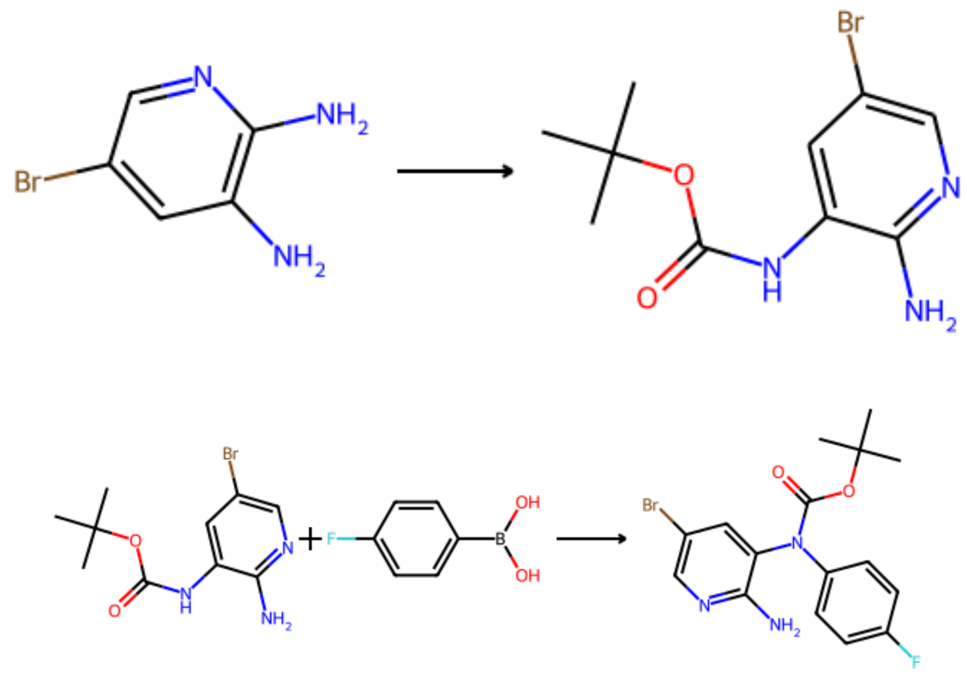

Product 98

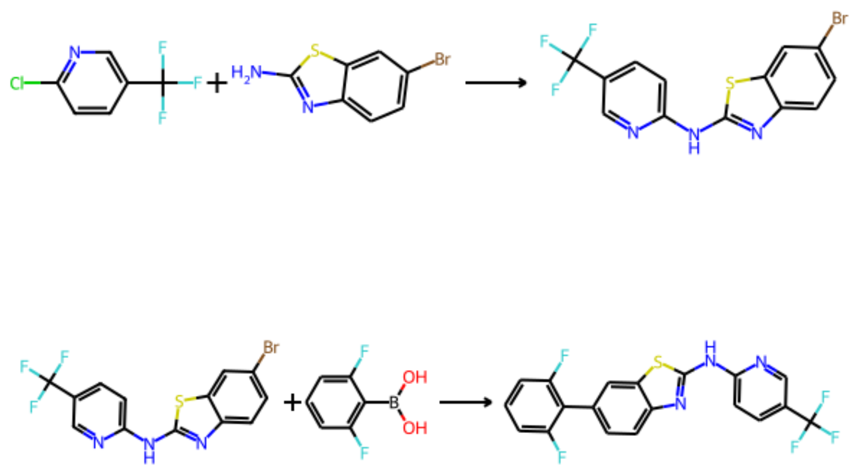

Product 99

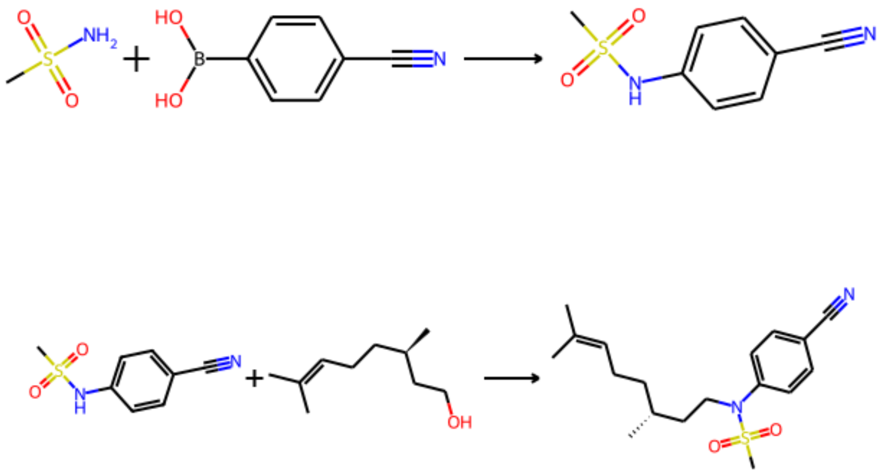

Product 100

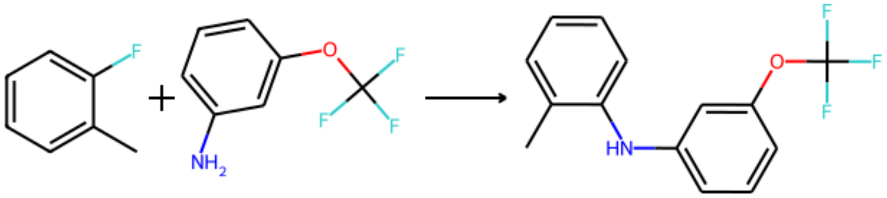

Product 101

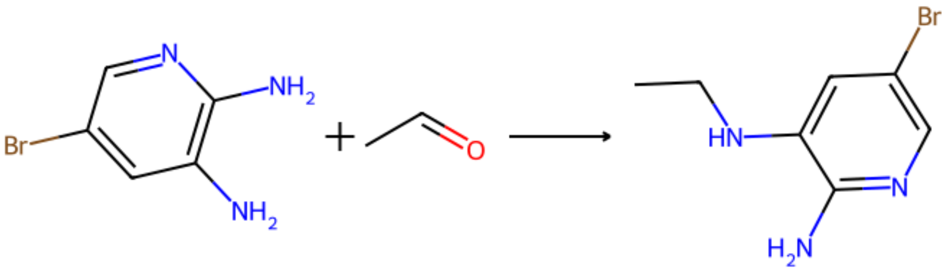

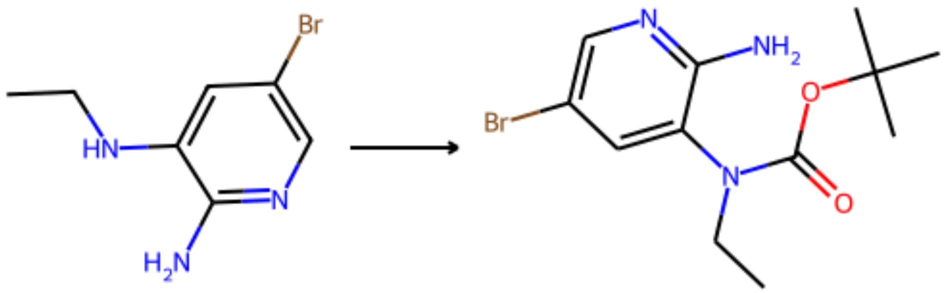

Product 102

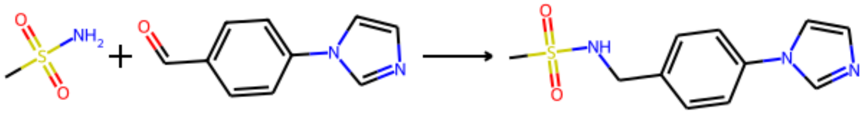

Product 103

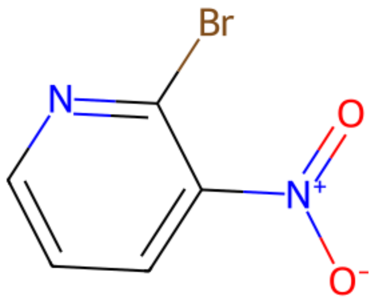

Product 104

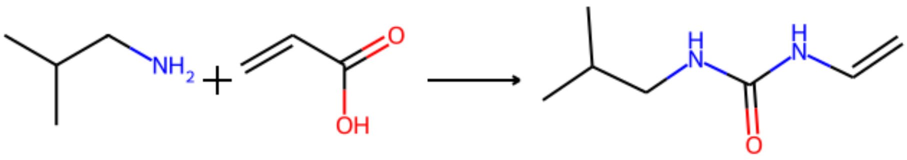

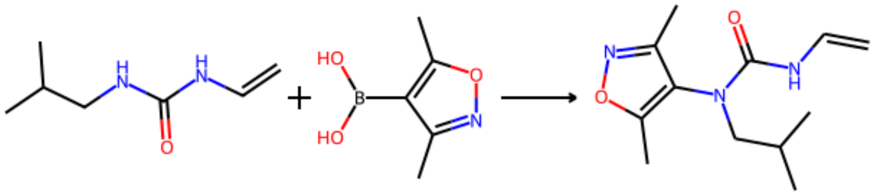

Product 105

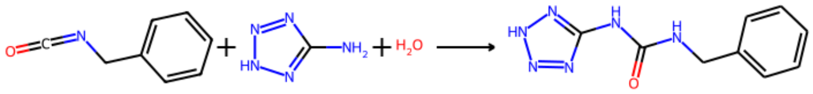

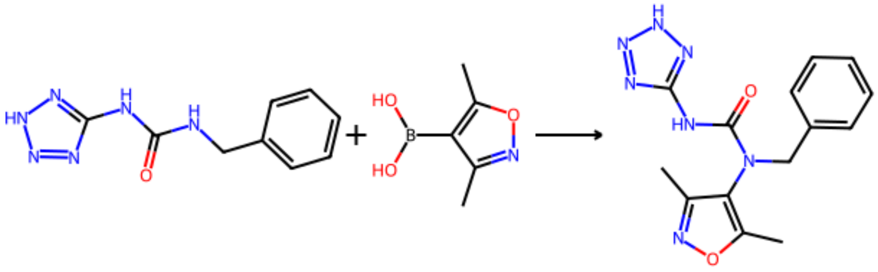

Product 106

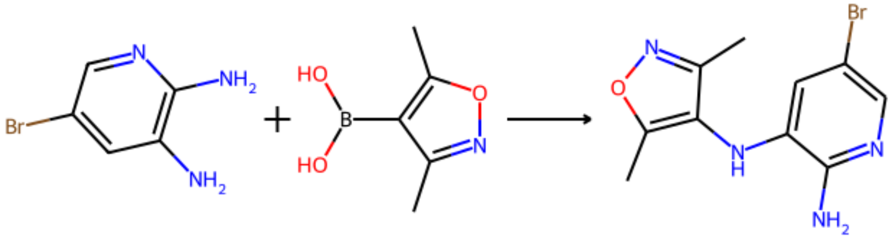

Product 107

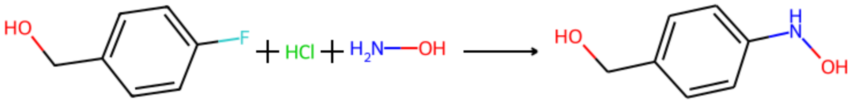

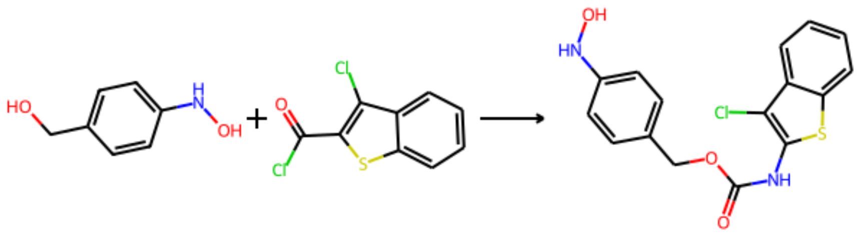

Product 108

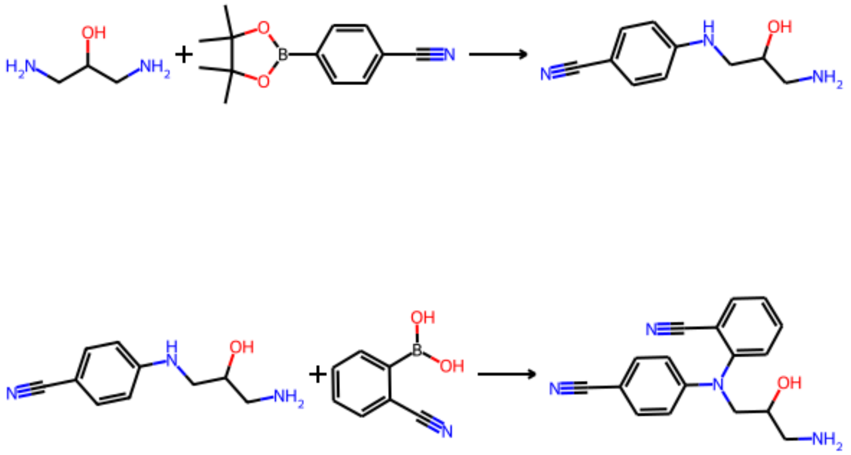

Product 109

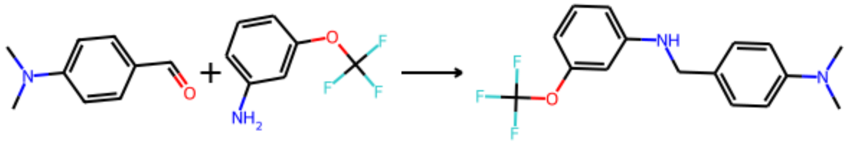

Product 110

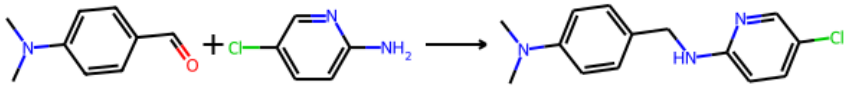

Product 111

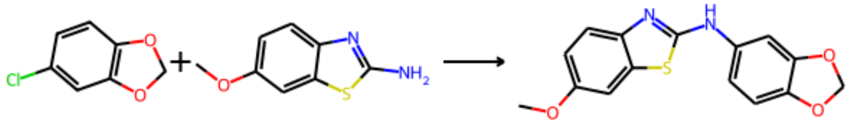

Product 112

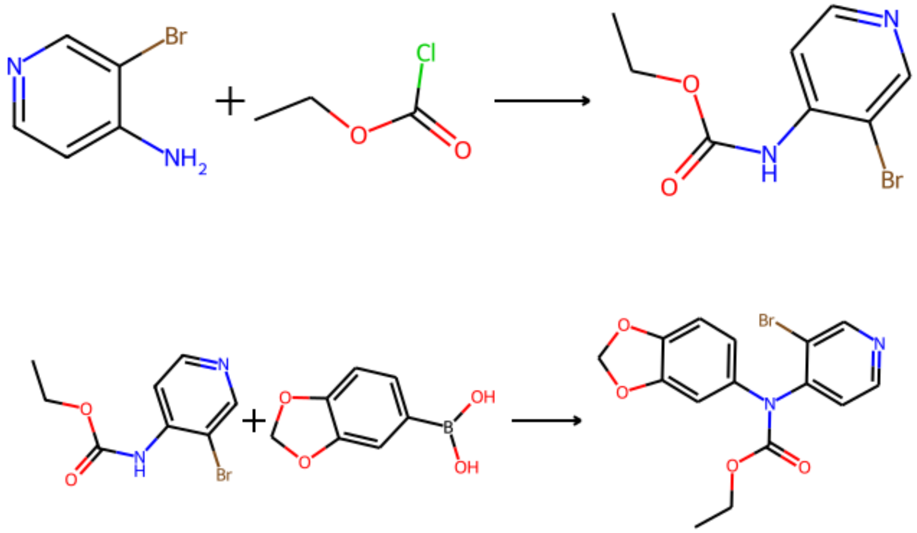

Product 113

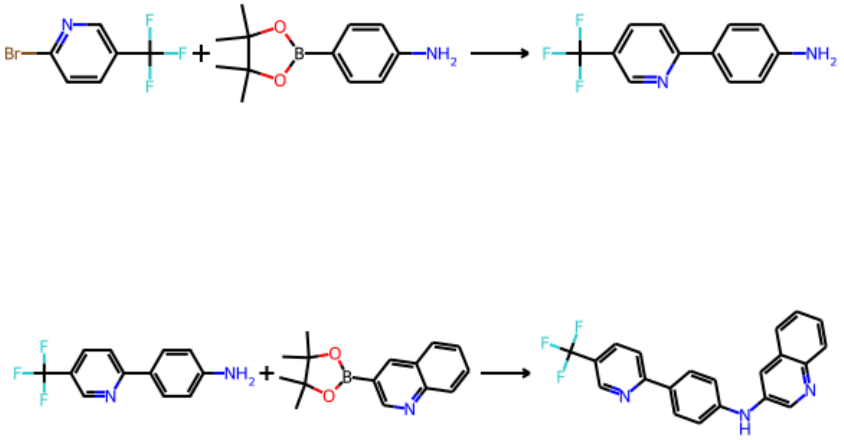

Product 114

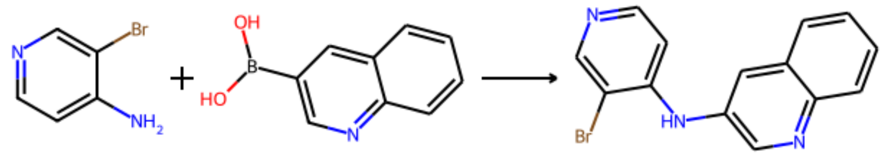

Product 115

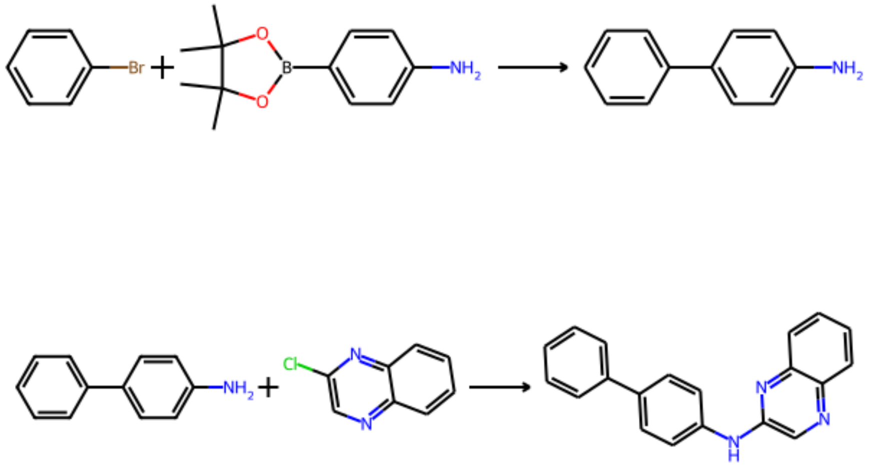

Product 116

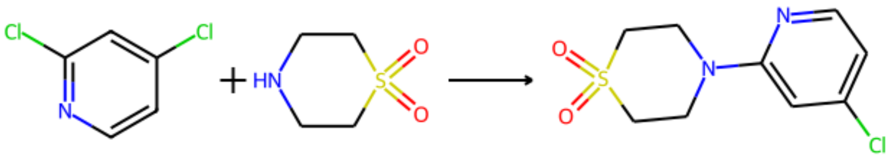

Product 117

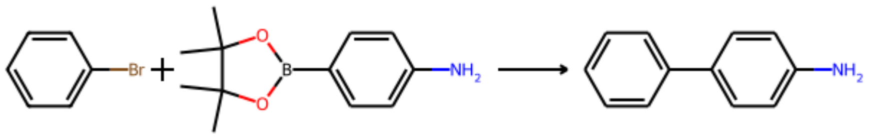

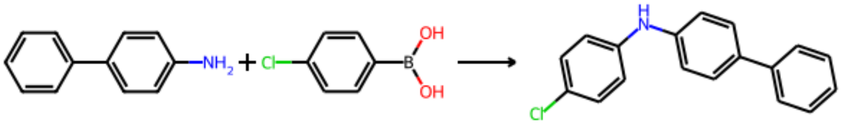

Product 118

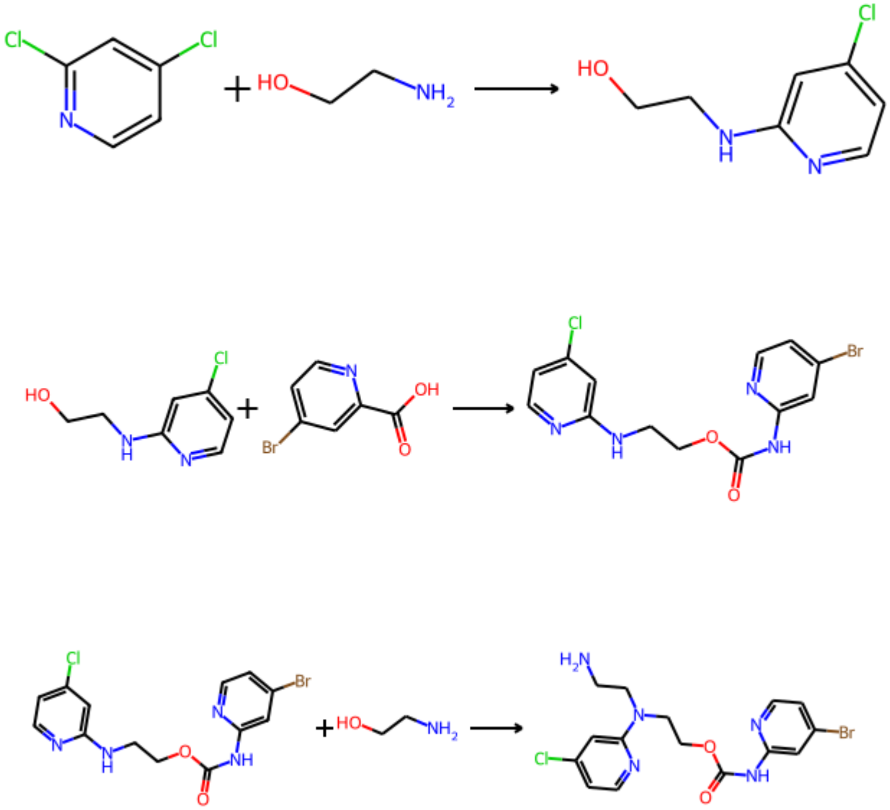

Product 119

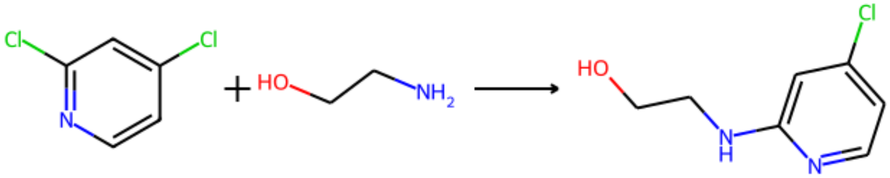

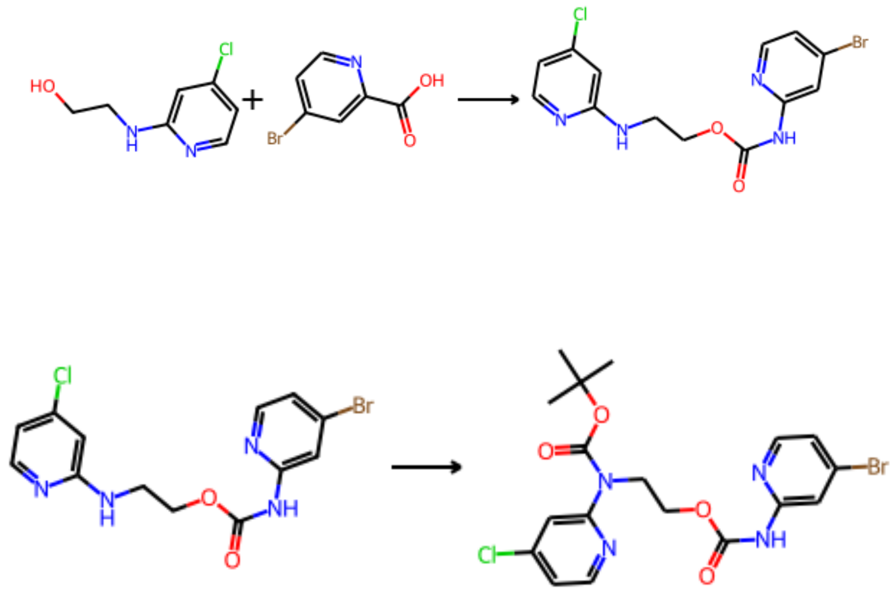

Product 120

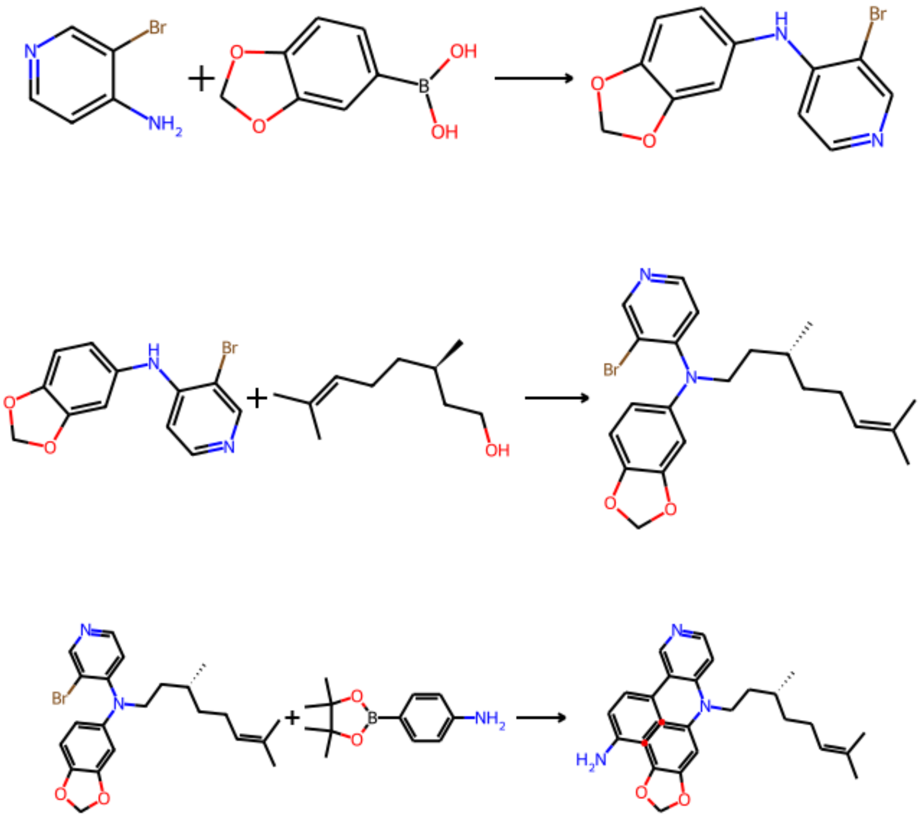

Product 121

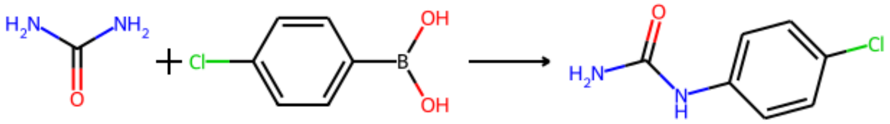

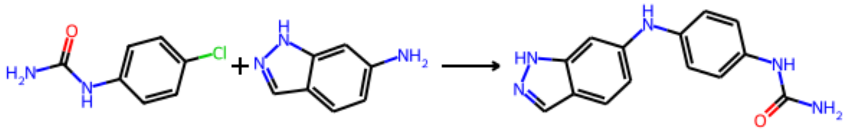

Product 122

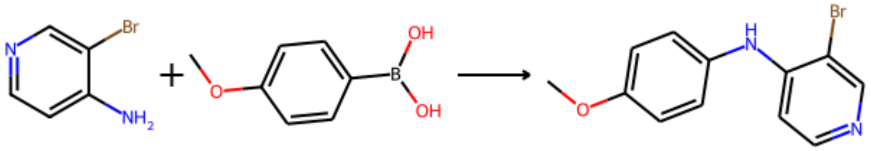

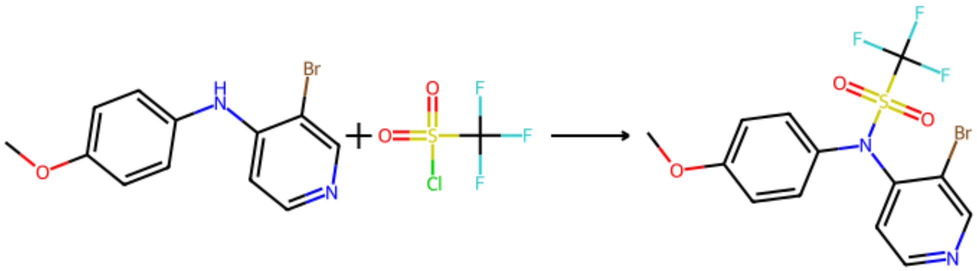

Product 123

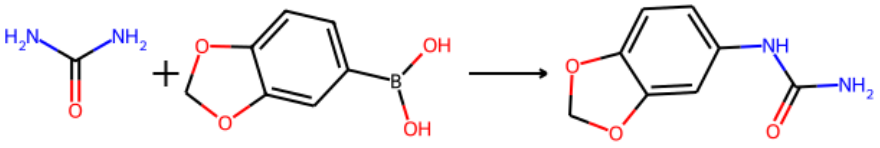

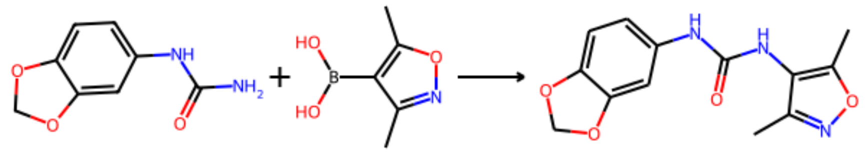

Product 124

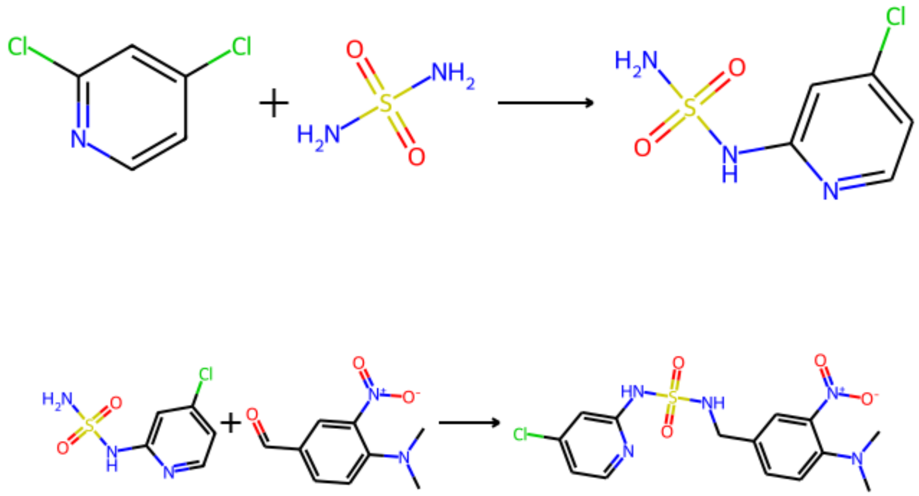

Product 125

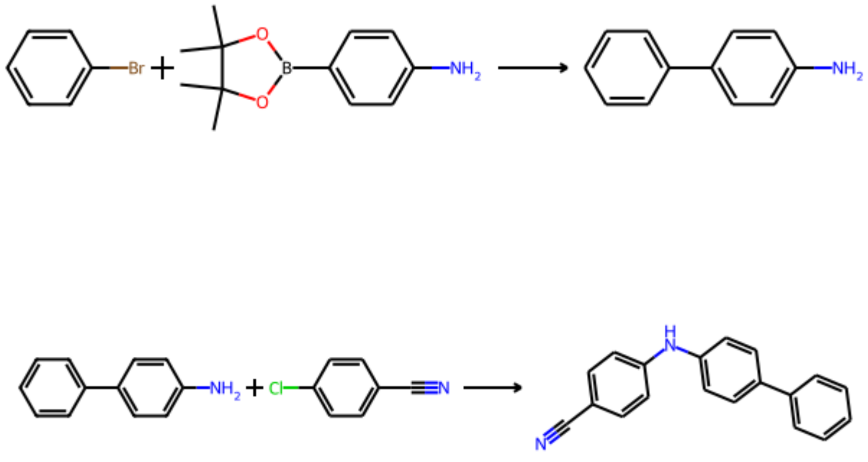

Product 126

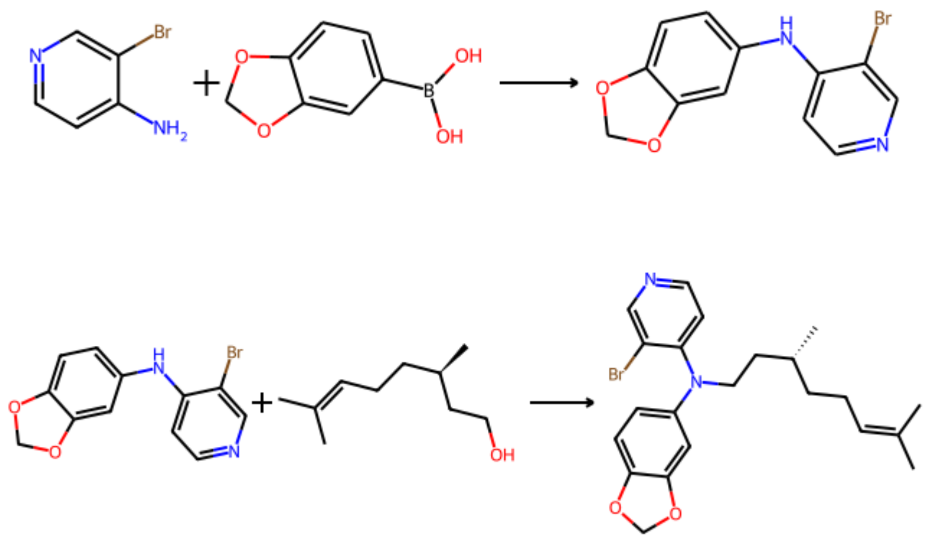

Product 127

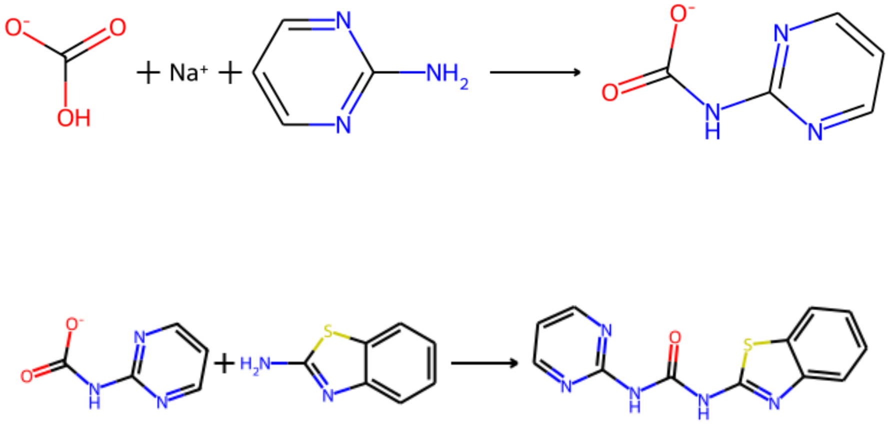

Product 128

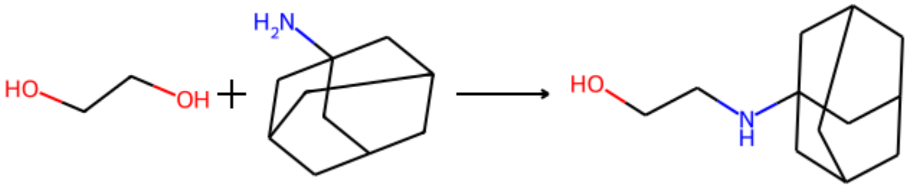

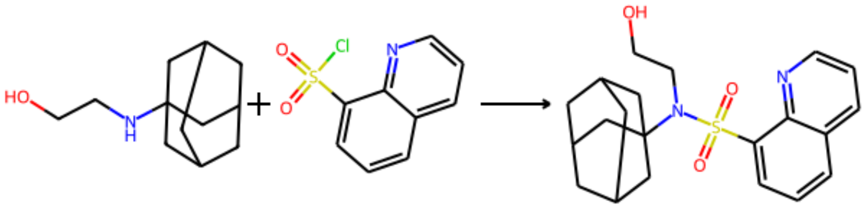

Product 129

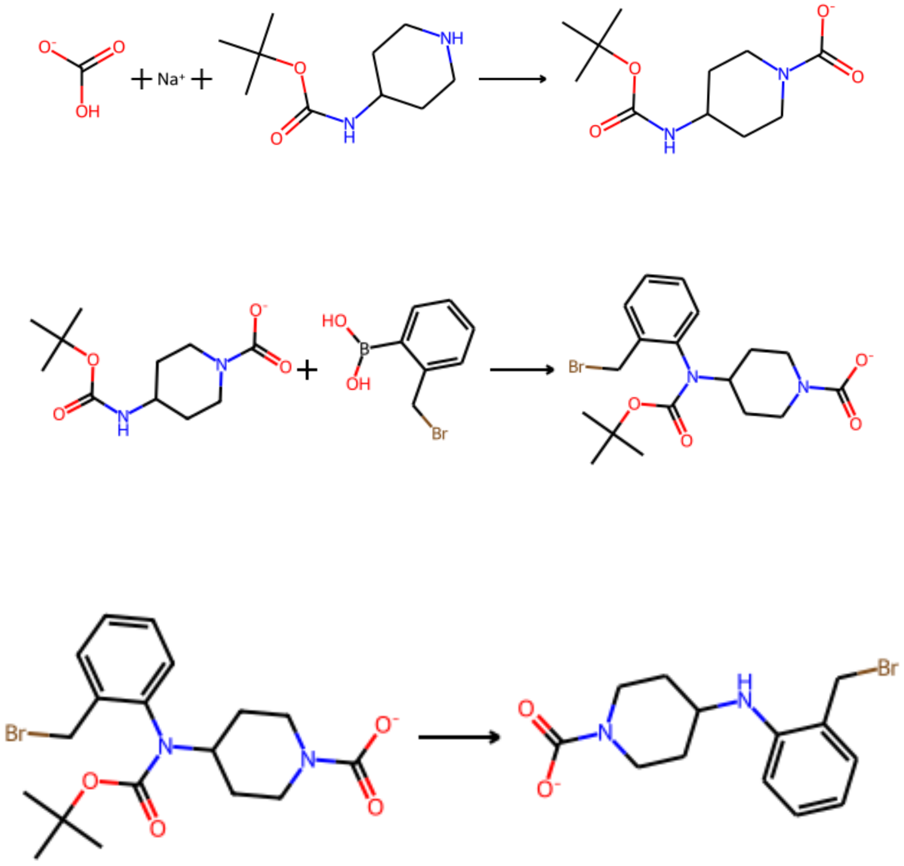

Product 130

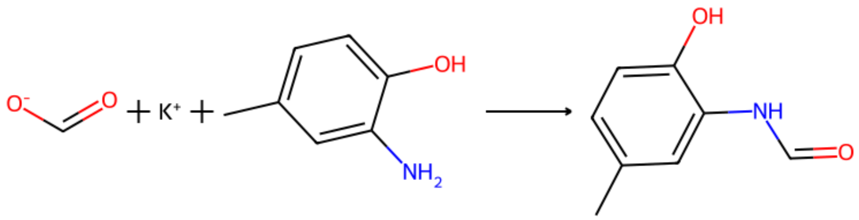

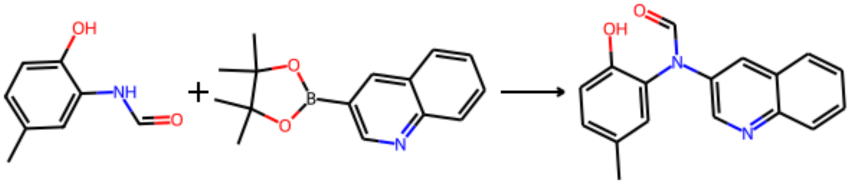

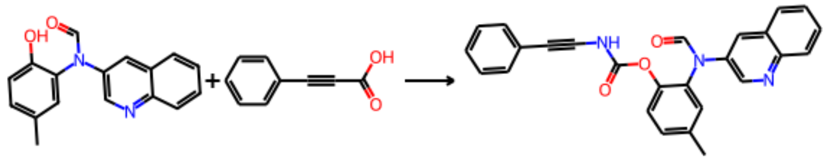

Product 131

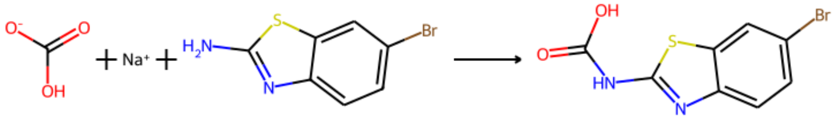

Product 132

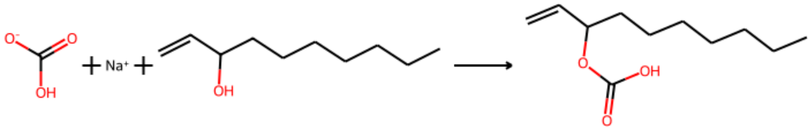

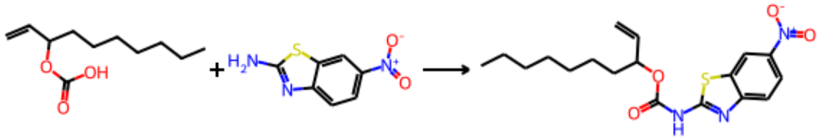

Product 133

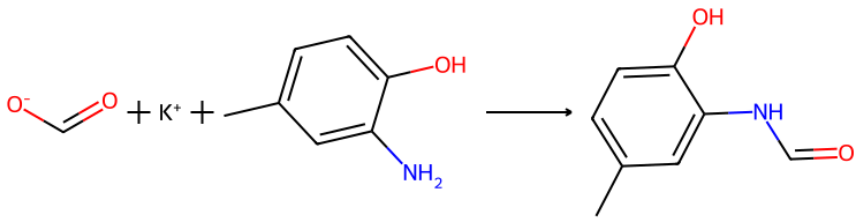

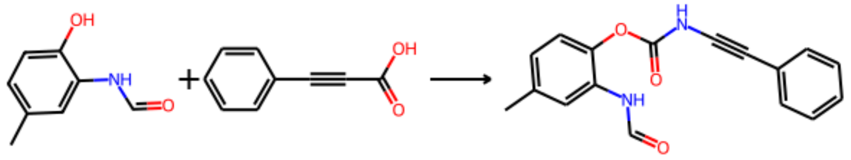

Product 134

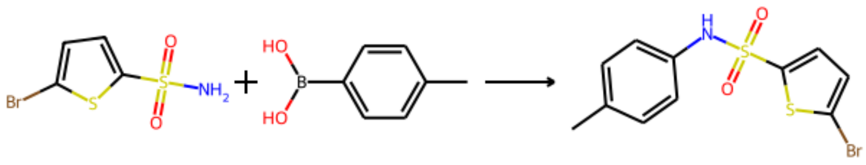

Product 135

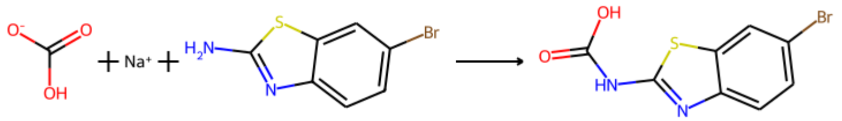

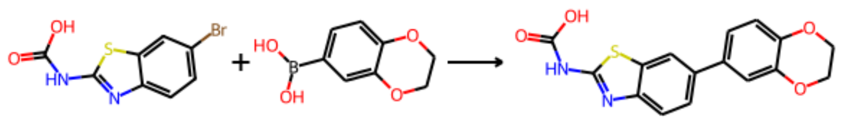

Product 136

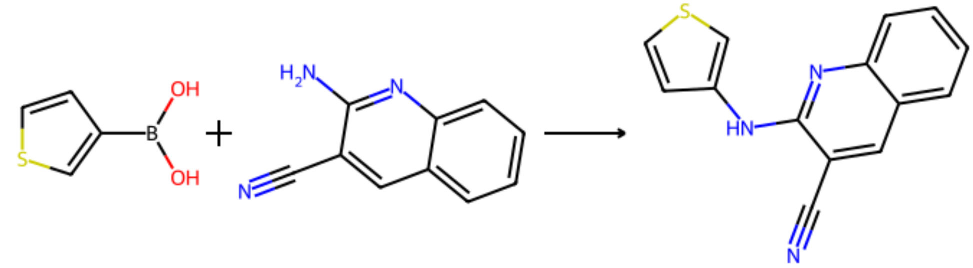

Product 137

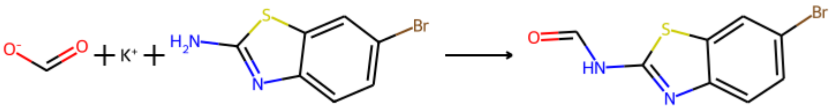

Product 138

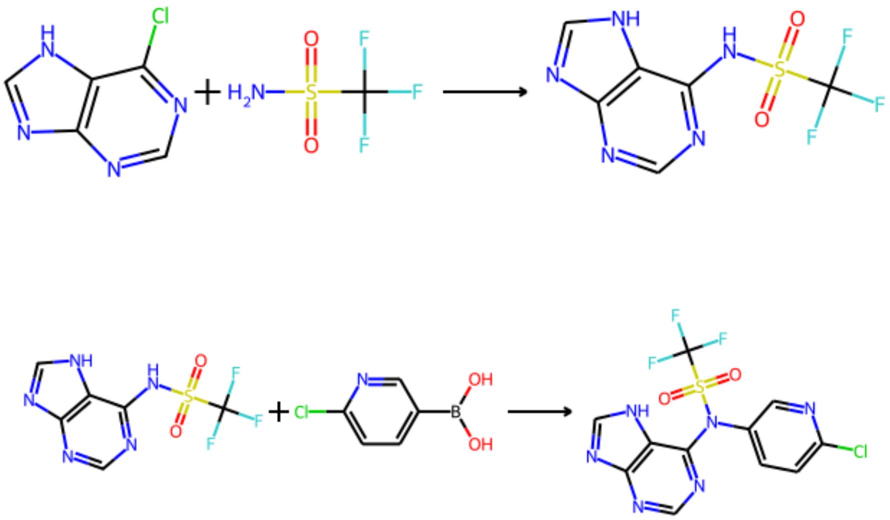

Product 139

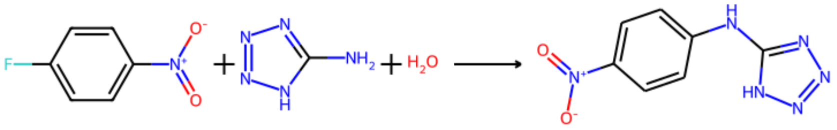

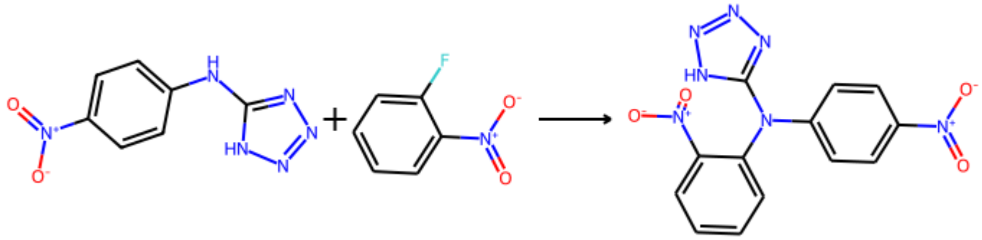

Product 140

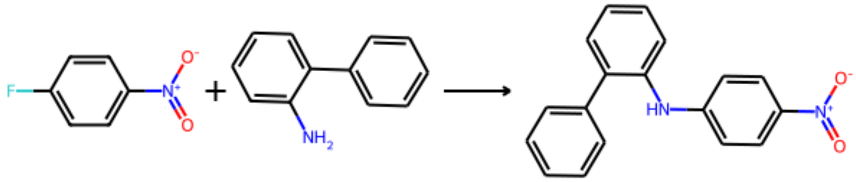

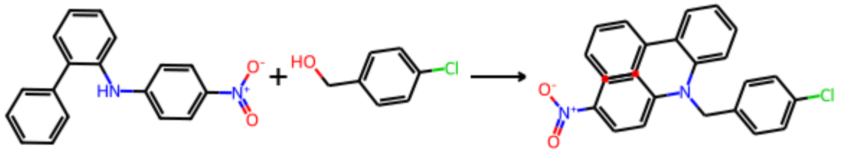

Product 141

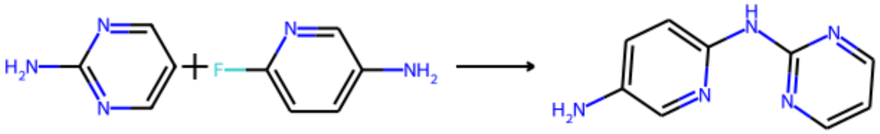

Product 142

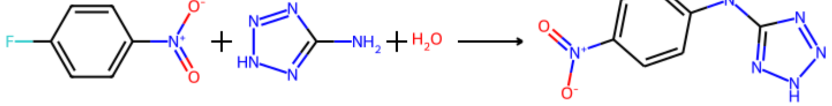

Product 143

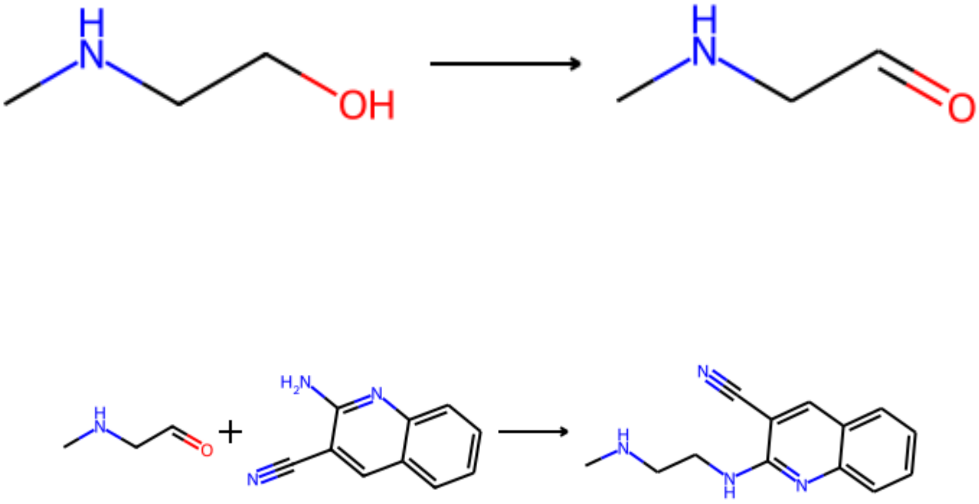

Product 144

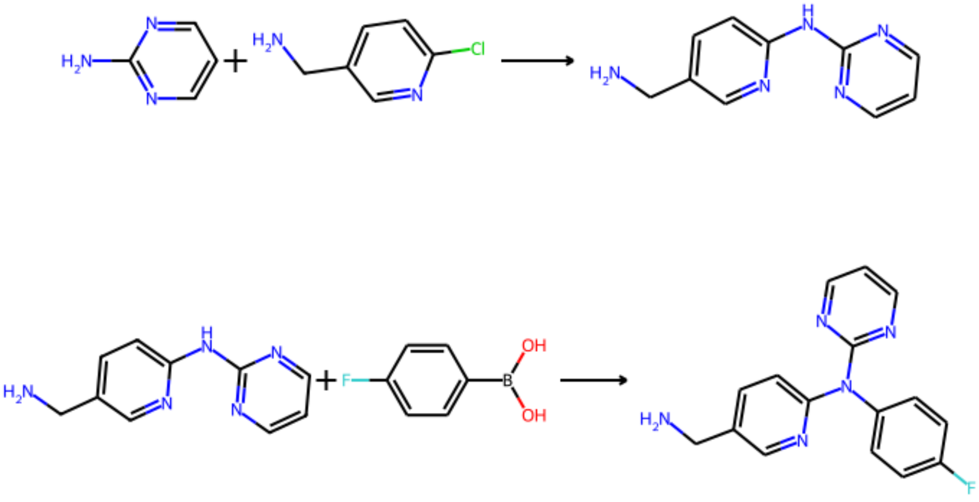

Product 145

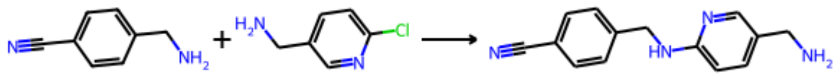

Product 146

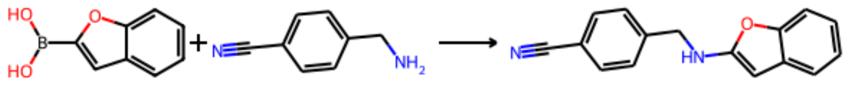

Product 147

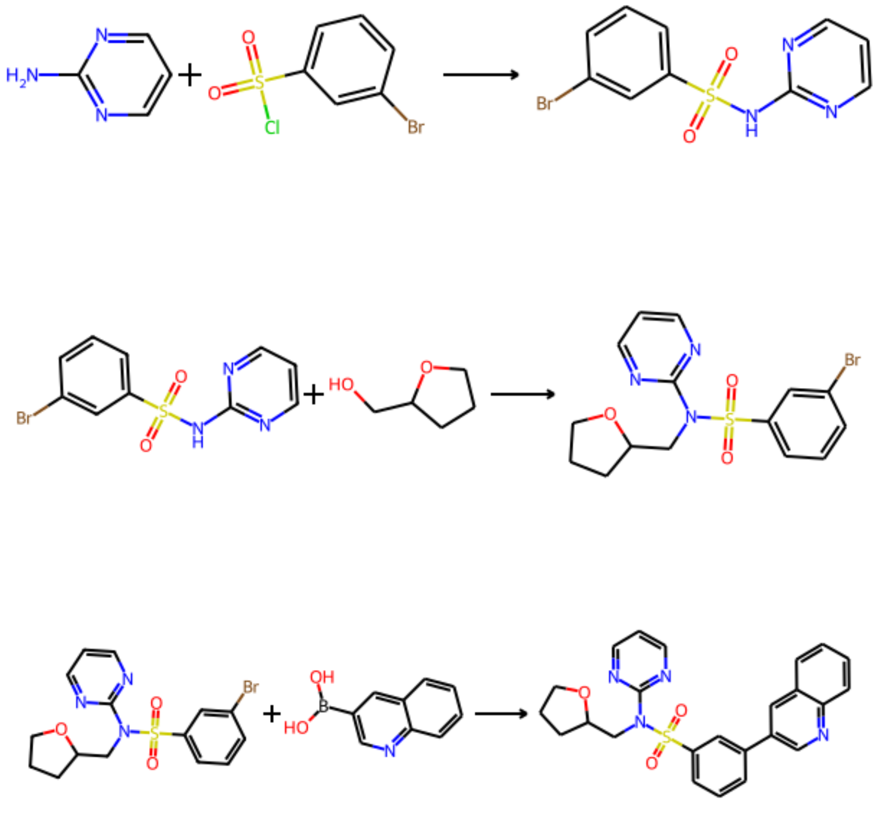

Product 148

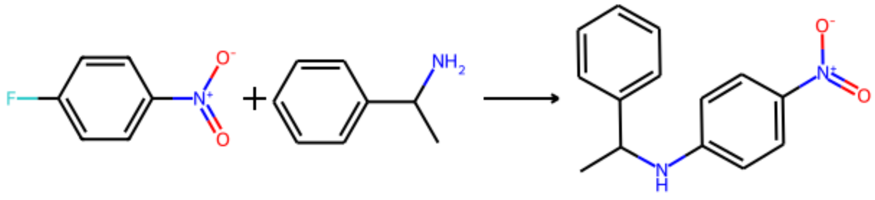

Product 149

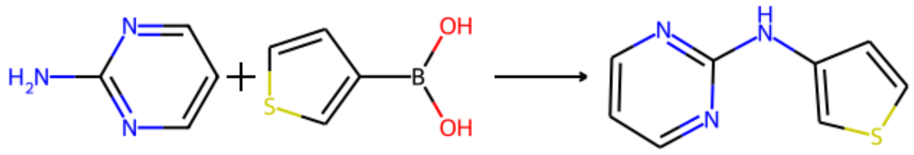

Product 150

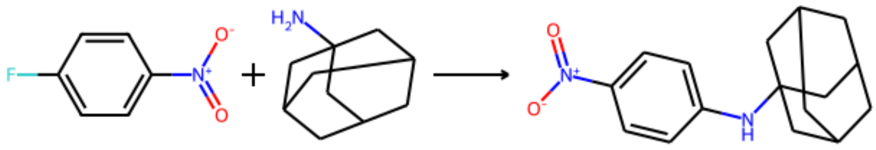

Product 151

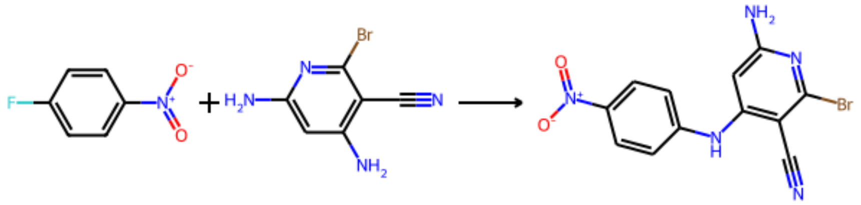

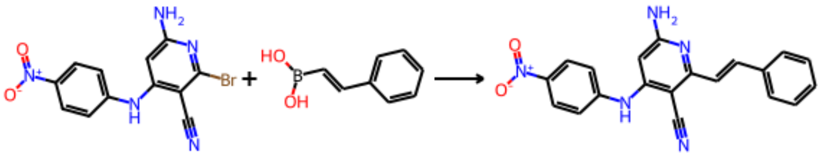

Product 152

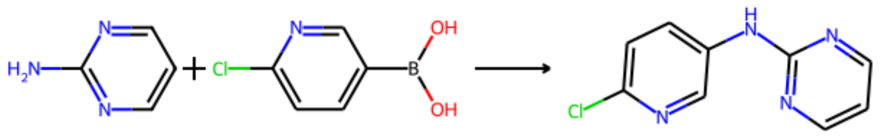

Product 153

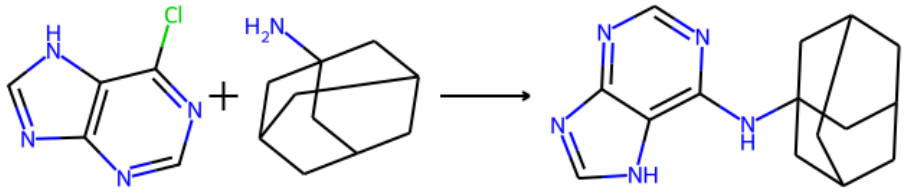

Product 154

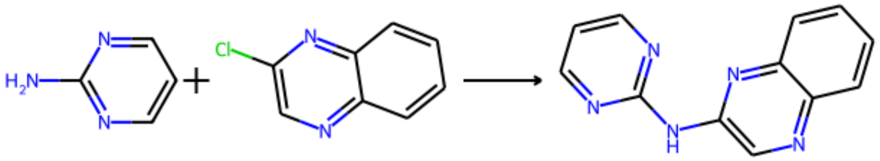

Product 155

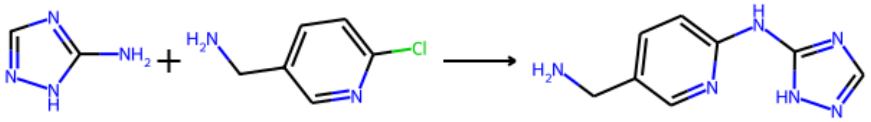

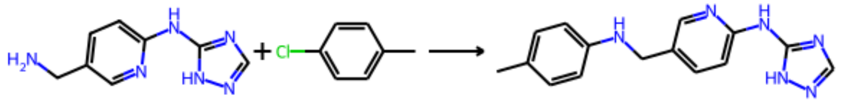

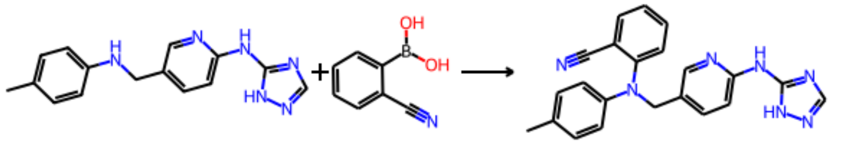

Product 156

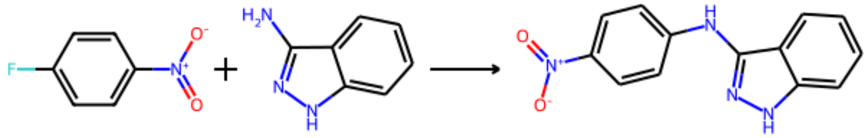

Product 157

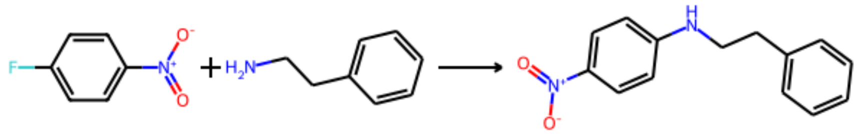

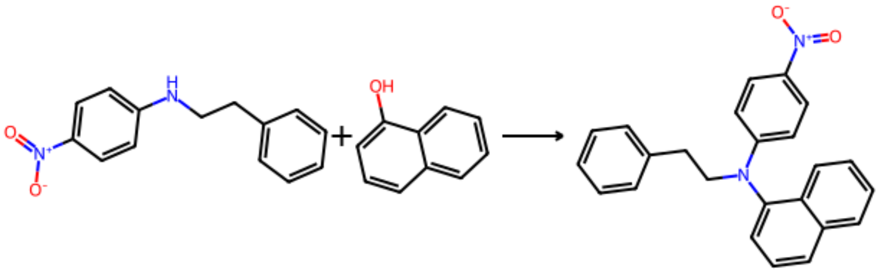

Product 158

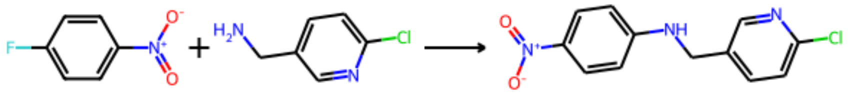

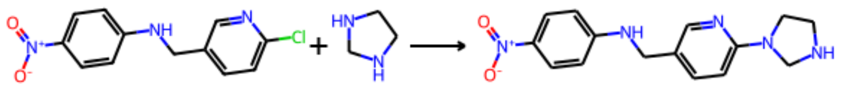

Product 159

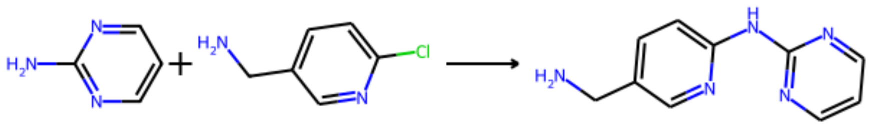

Product 160

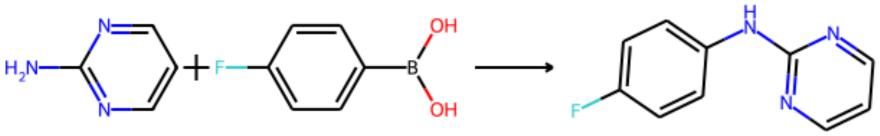

Product 161

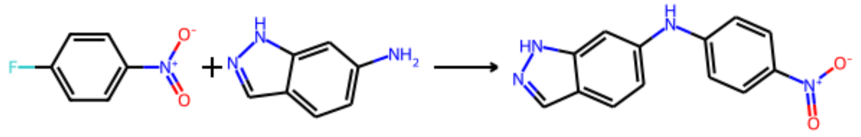

Product 162

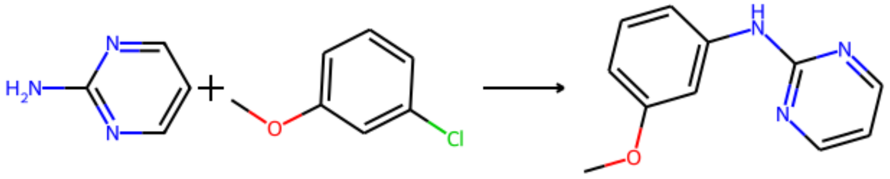

Product 163

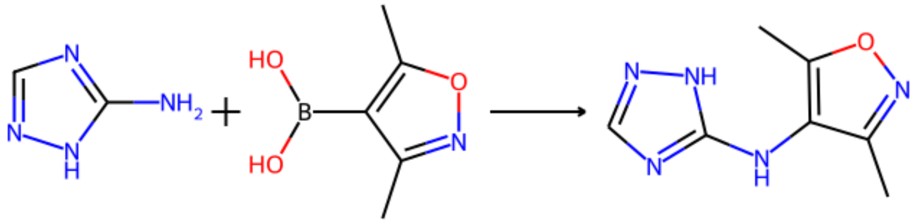

Product 164

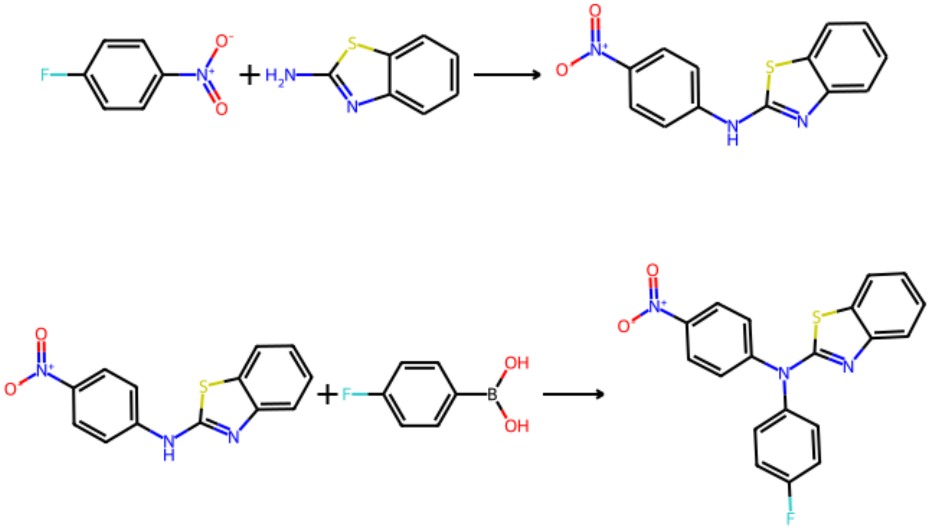

Product 165

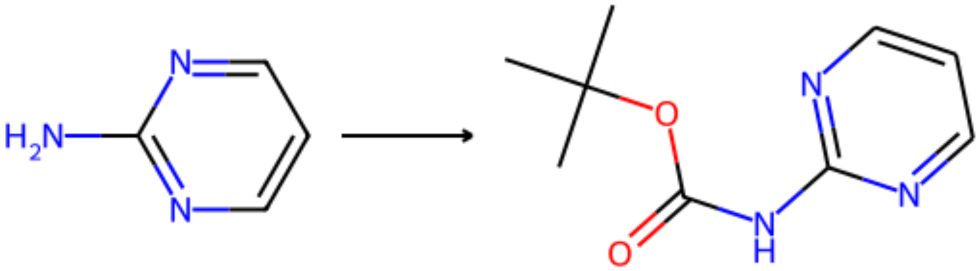

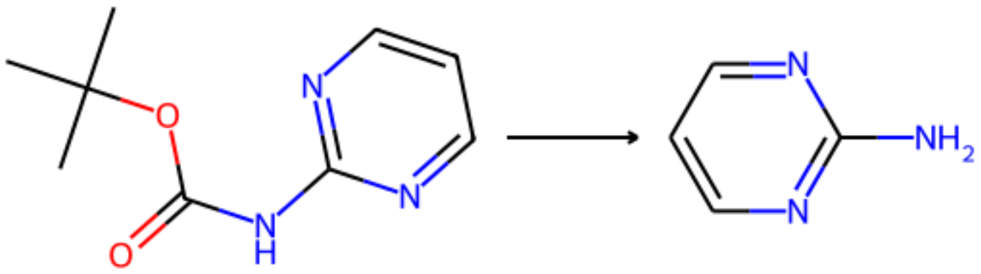

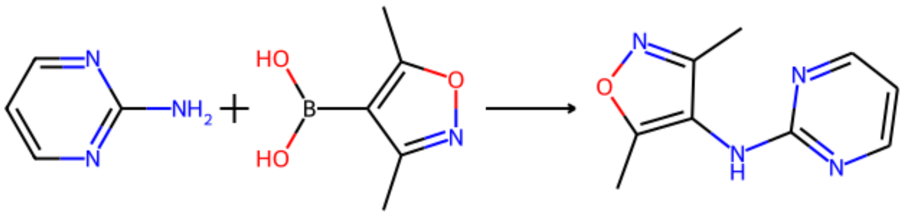

Product 166

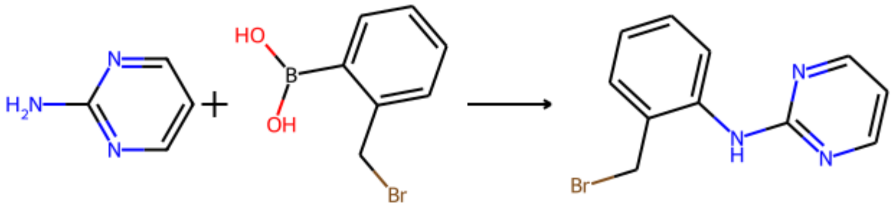

Product 167

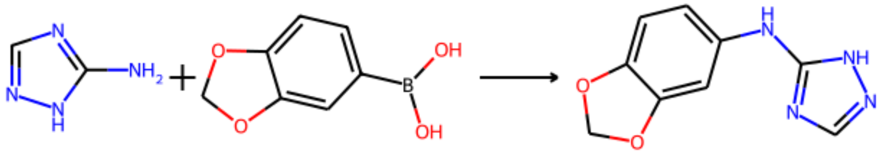

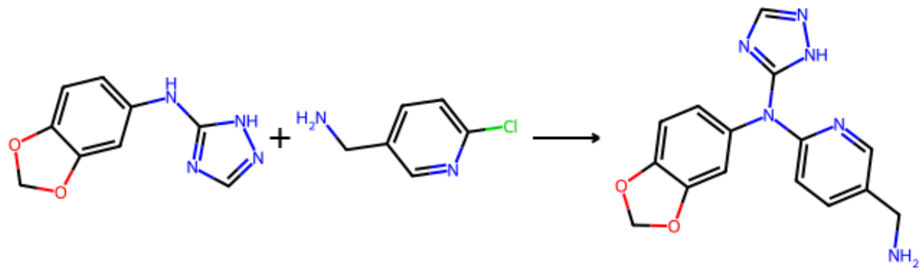

Product 168

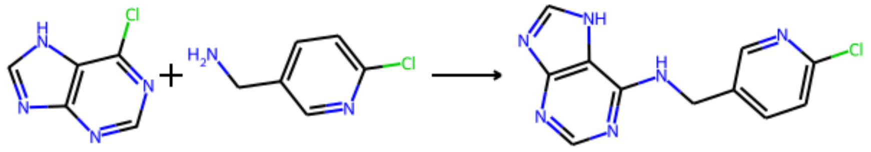

Product 169

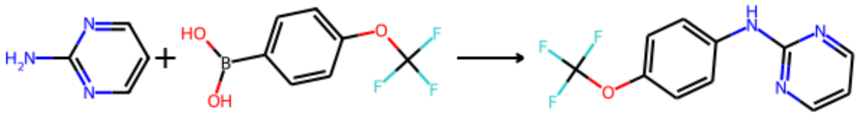

Product 170

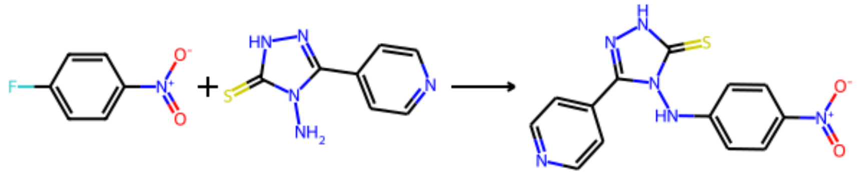

Product 171

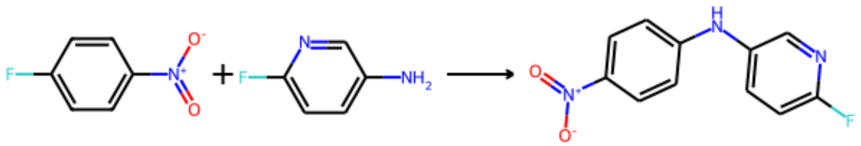

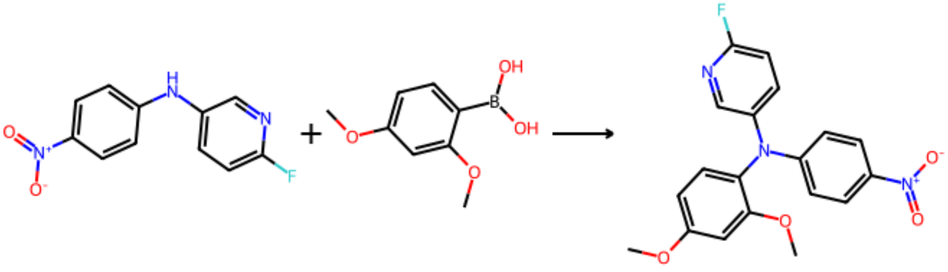

Product 172

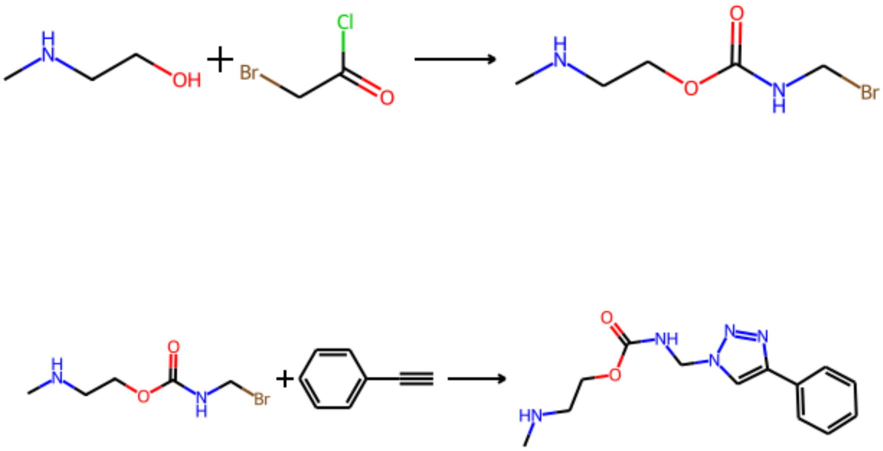

Product 173

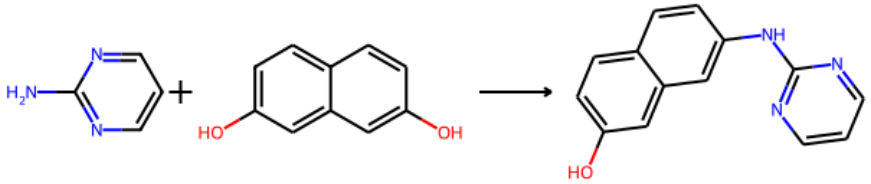

Product 174

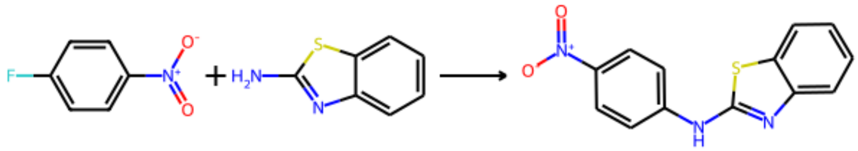

Product 175

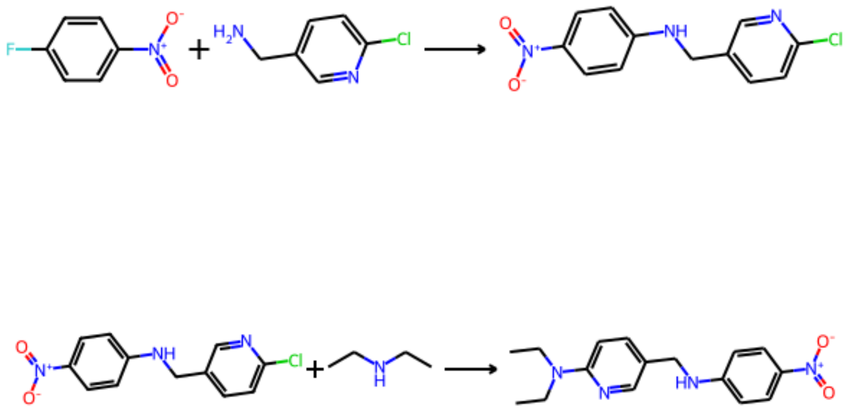

Product 176

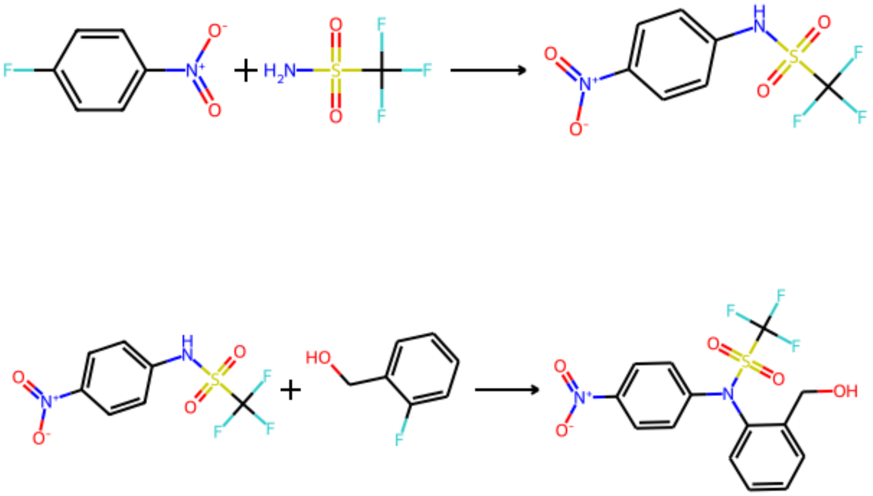

Product 177

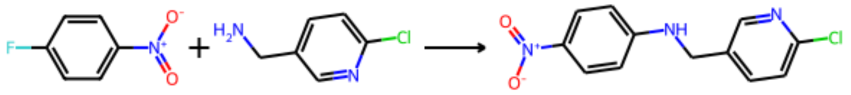

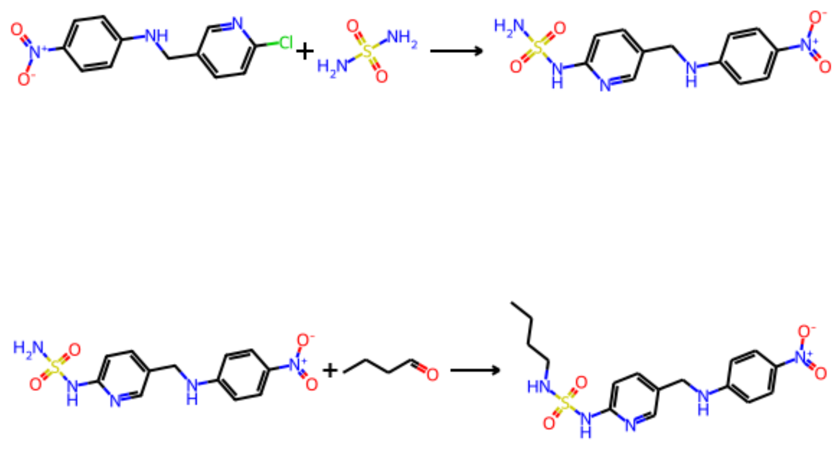

Product 178

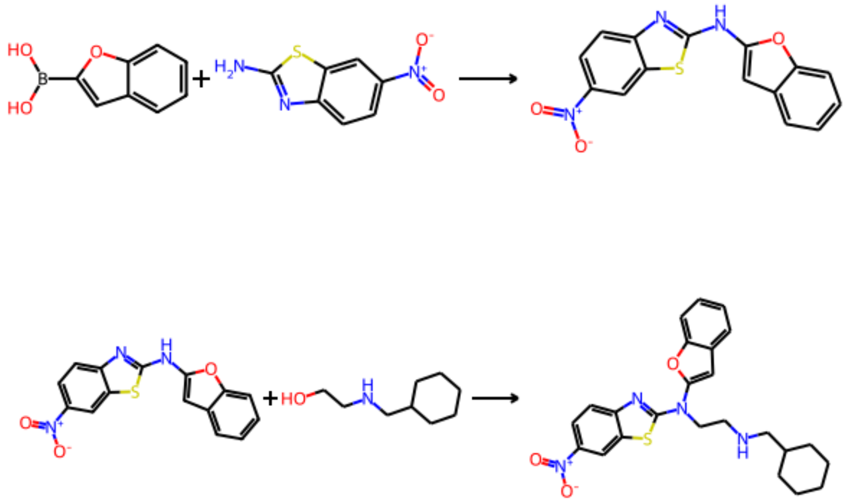

Product 179

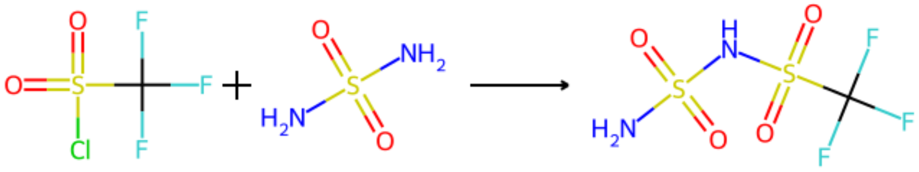

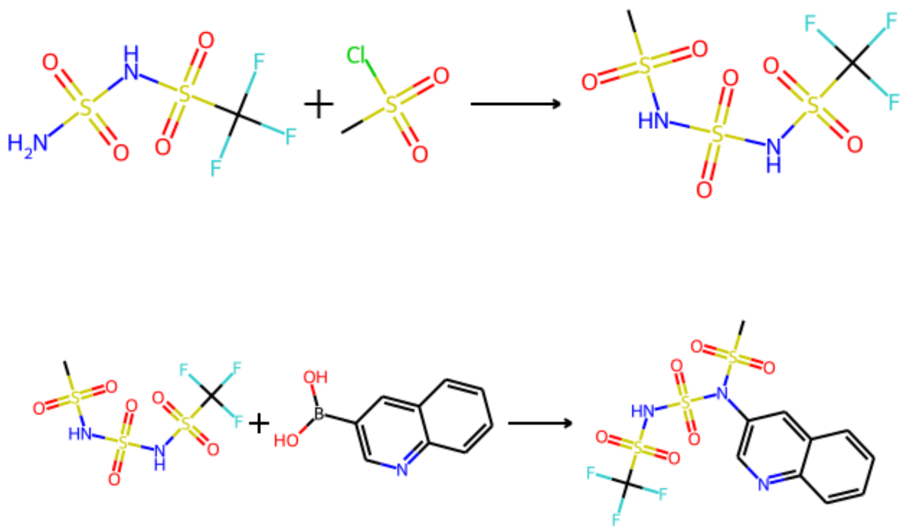

Product 180

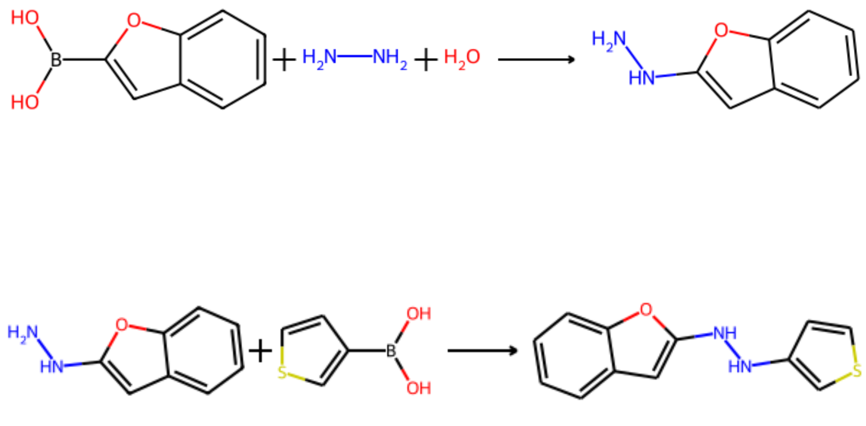

Product 181

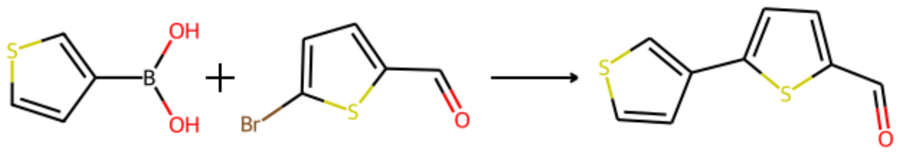

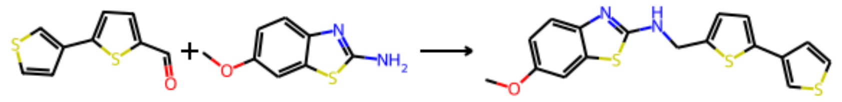

Product 182

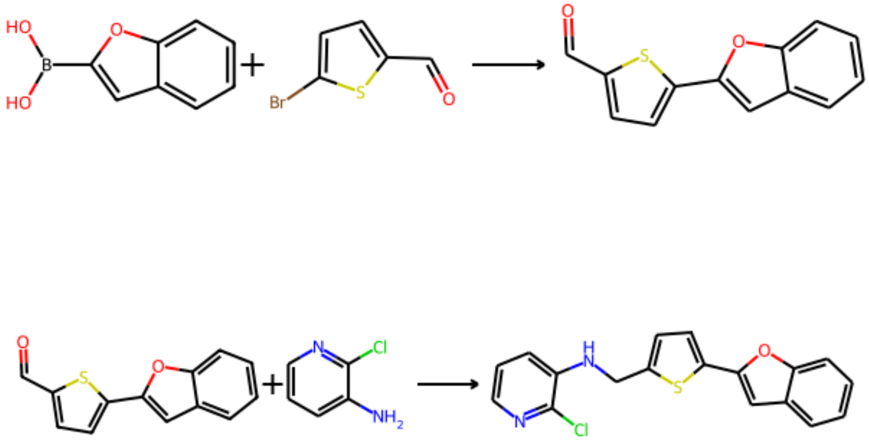

Product 183

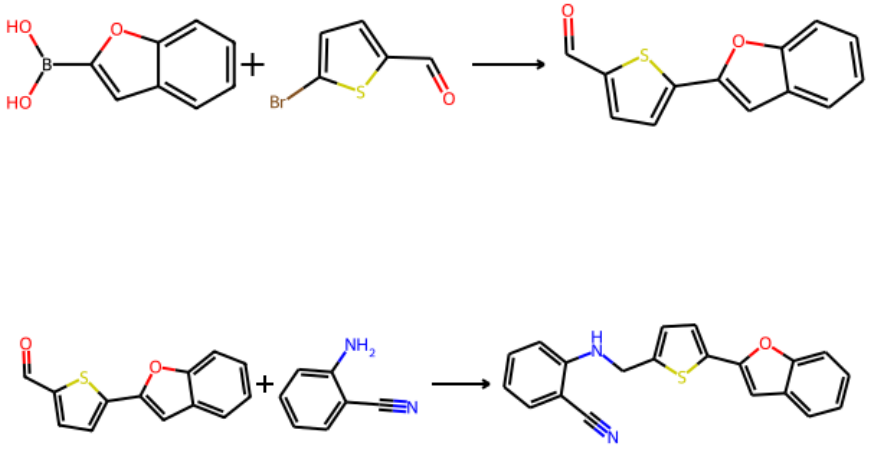

Product 184

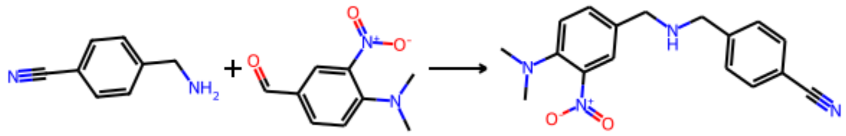

Product 185

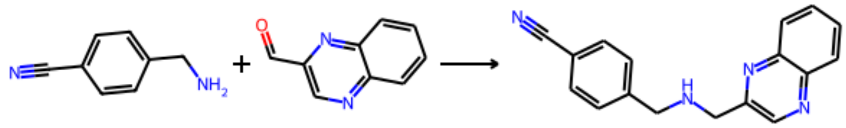

Product 186

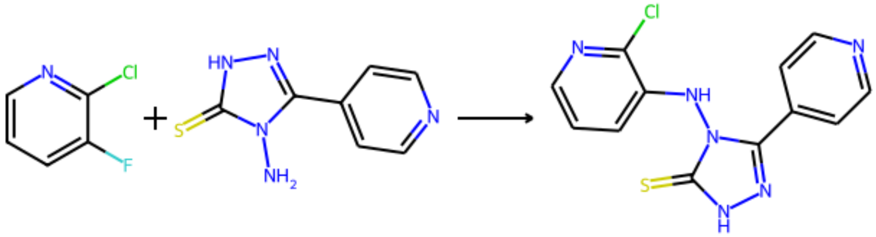

Product 187

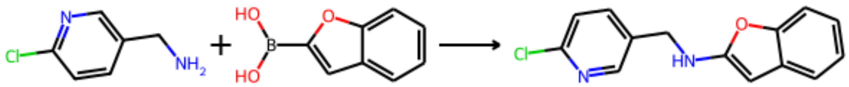

Product 188

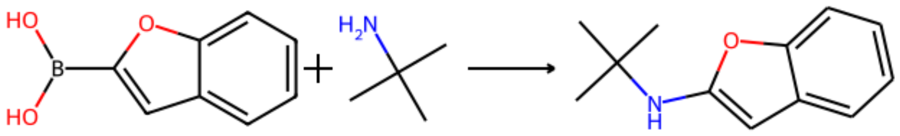

Product 189

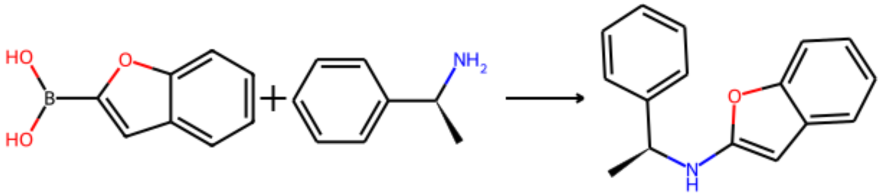

Product 190

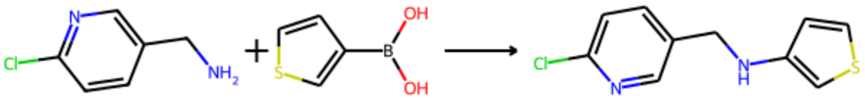

Product 191

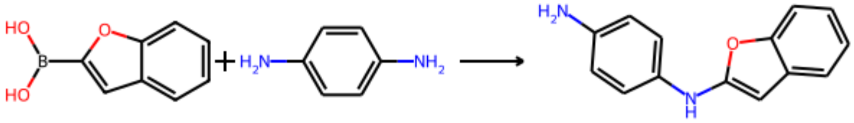

Product 192

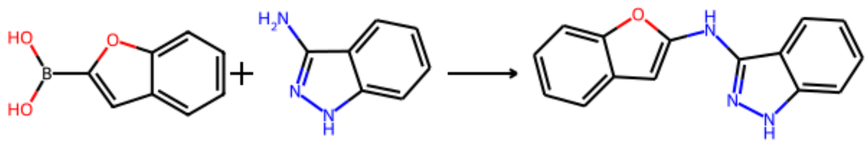

Product 193

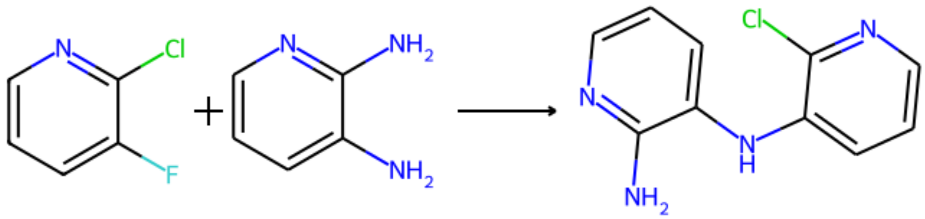

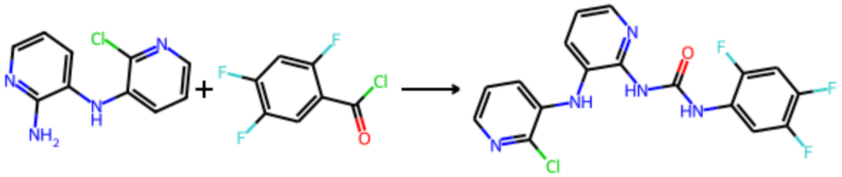

Product 194

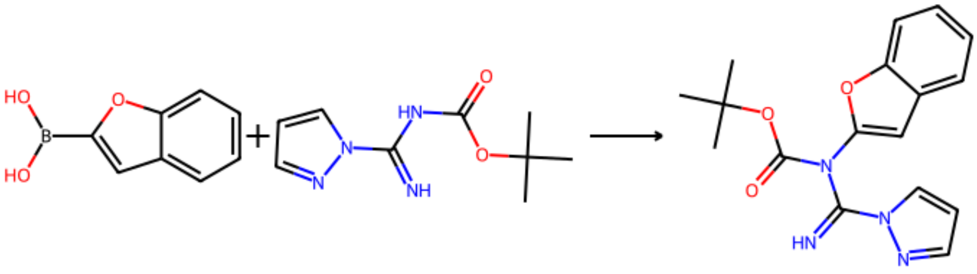

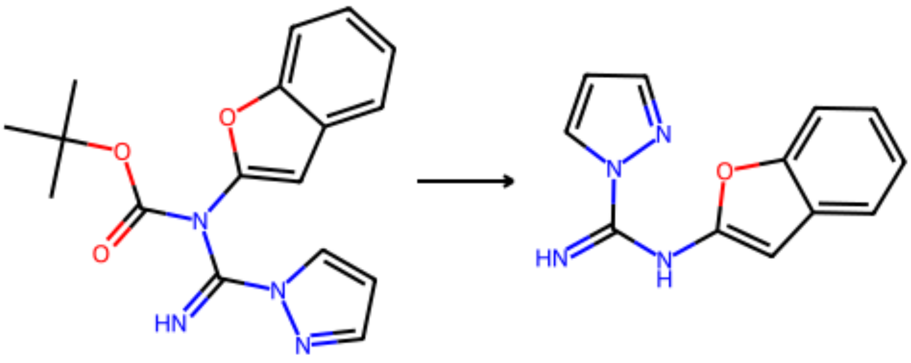

Product 195

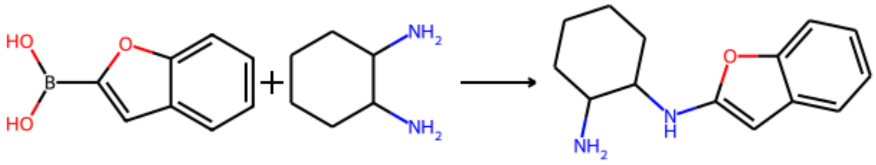

Product 196

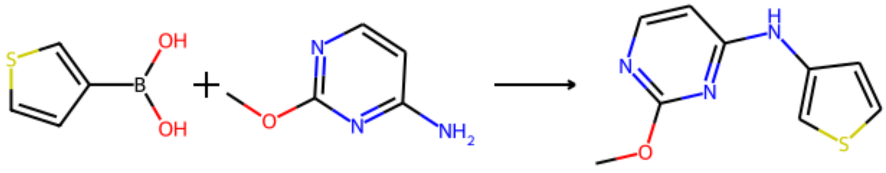

Product 197

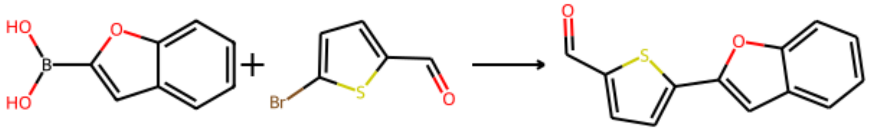

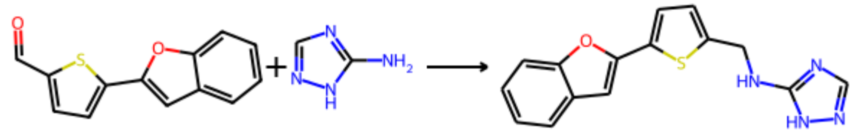

Product 198

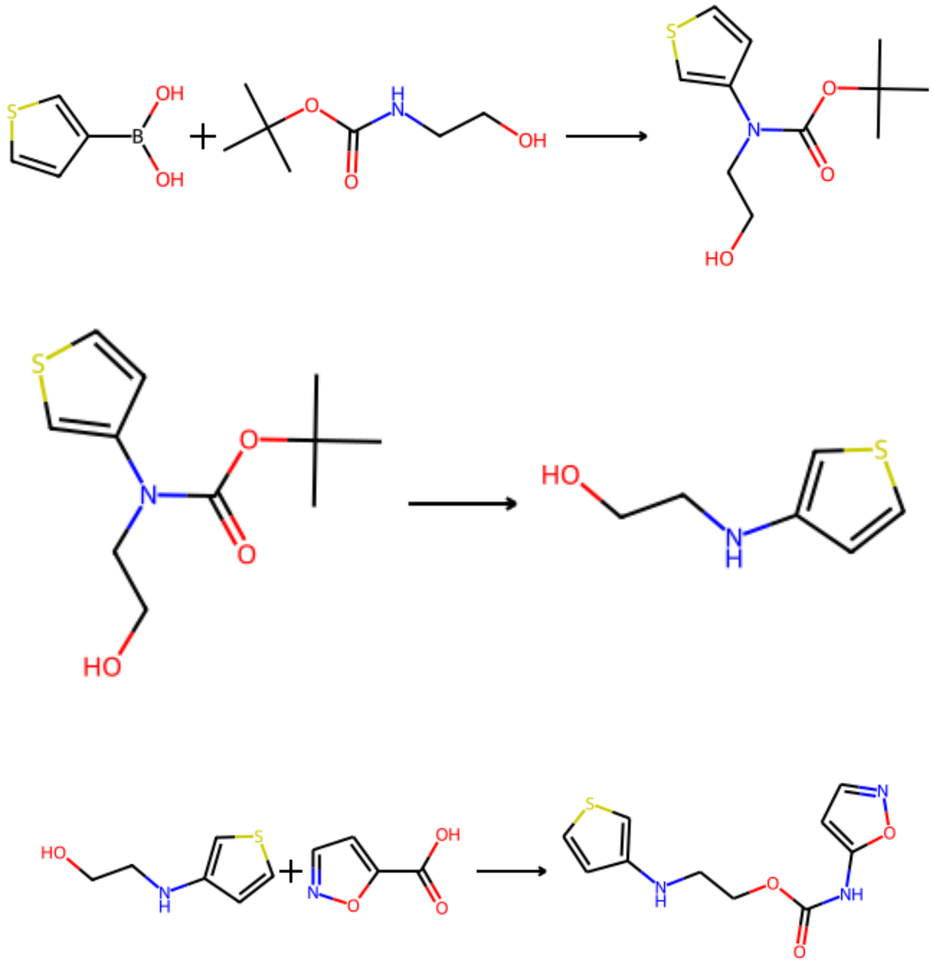

Product 199

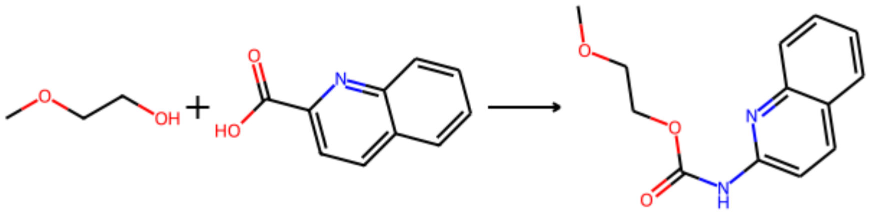

Product 200

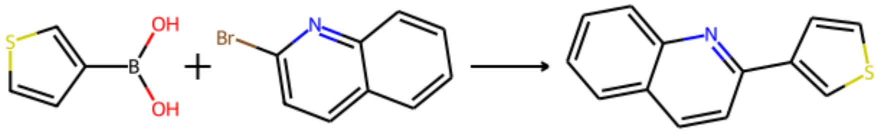

Product 201

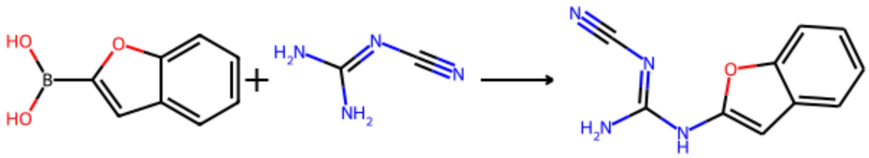

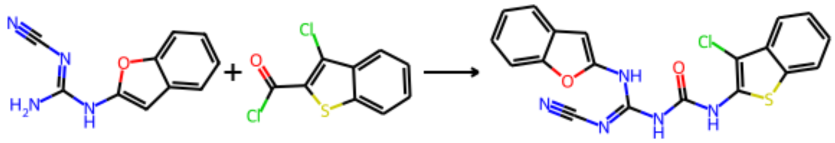

Product 202

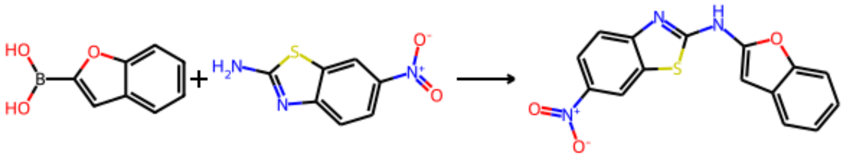

Product 203

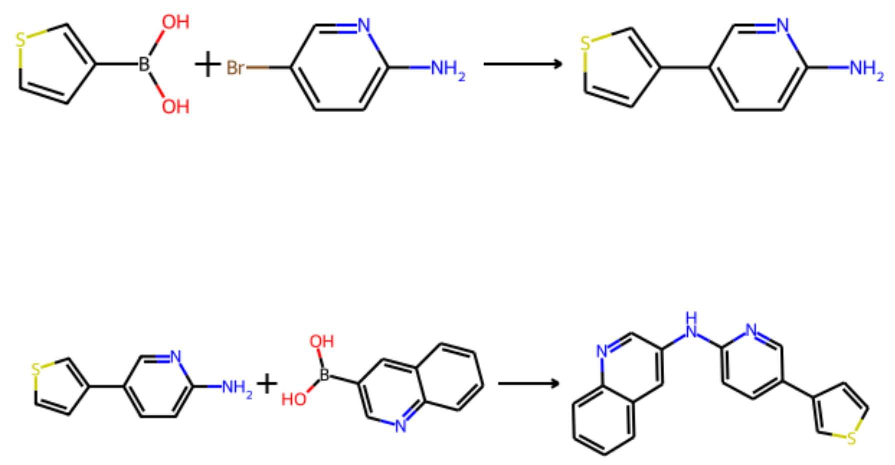

Product 204

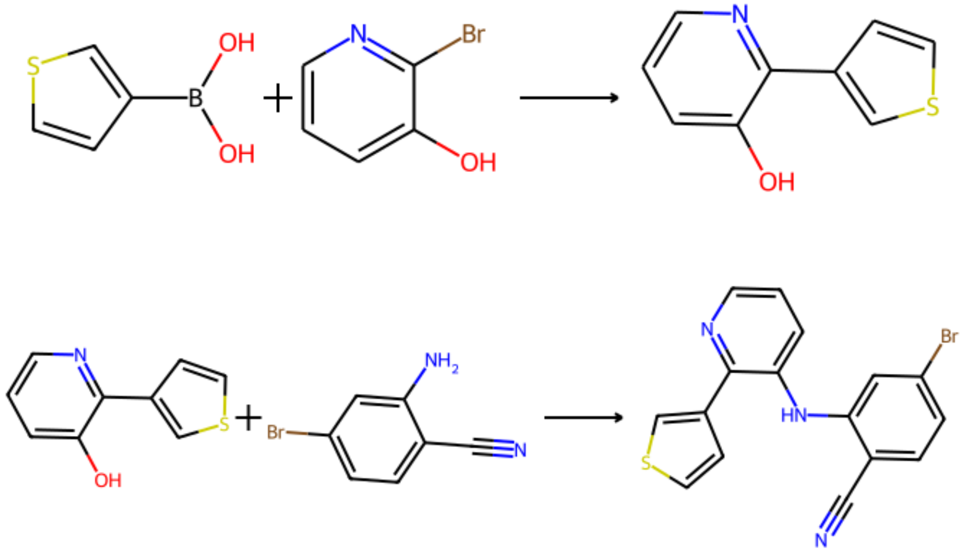

Product 205

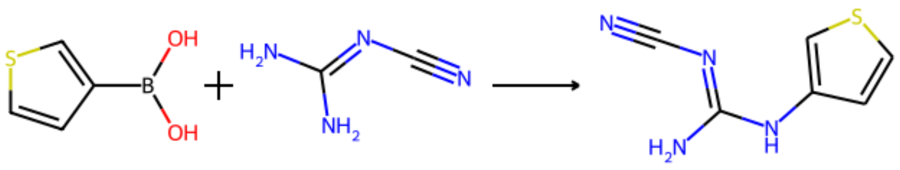

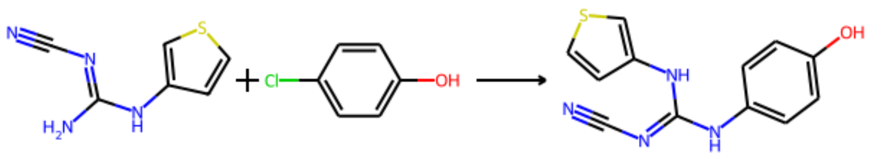

Product 206

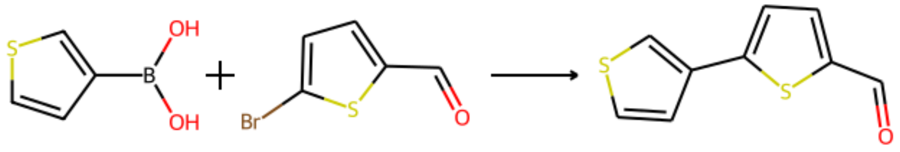

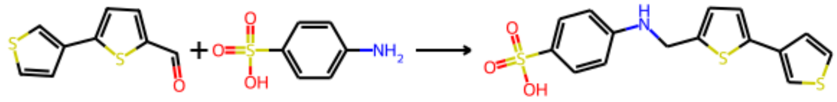

Product 207

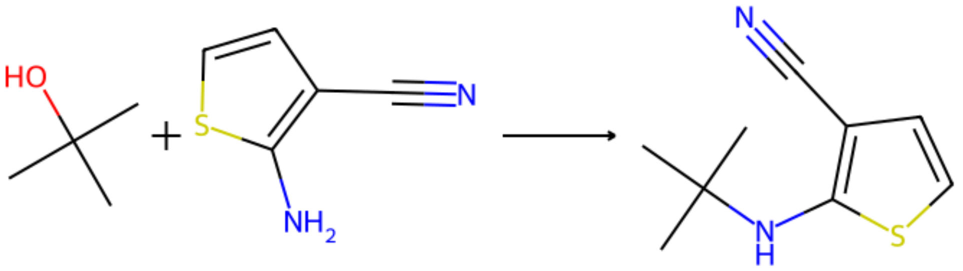

Product 208

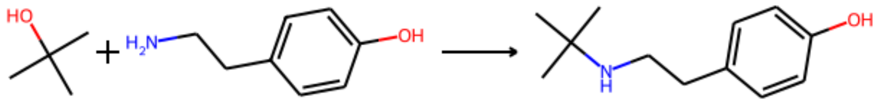

Product 209

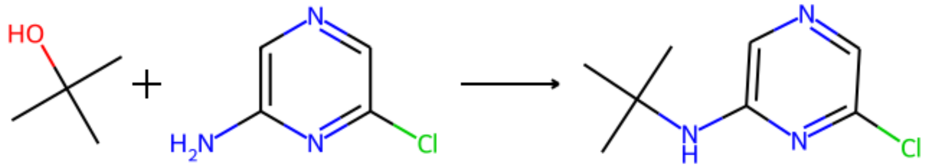

Product 210

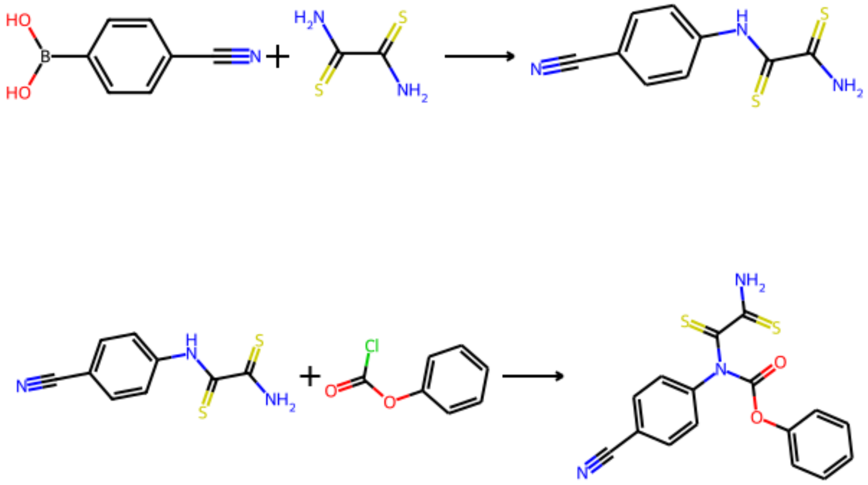

Product 211

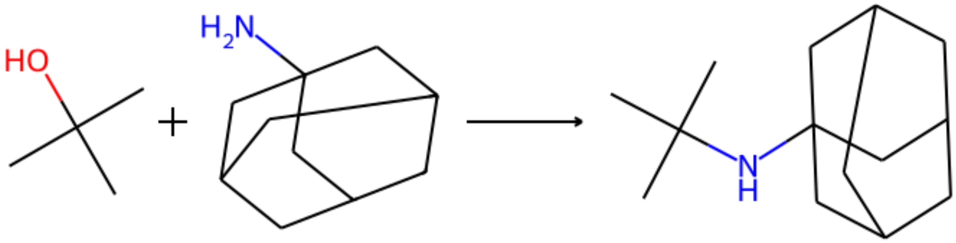

Product 212

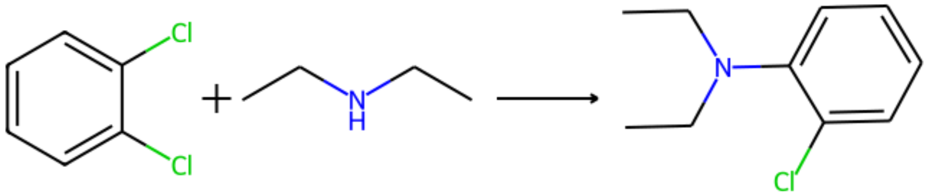

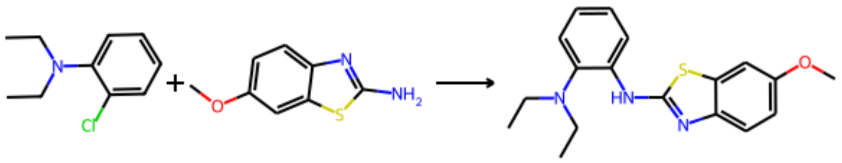

Product 213

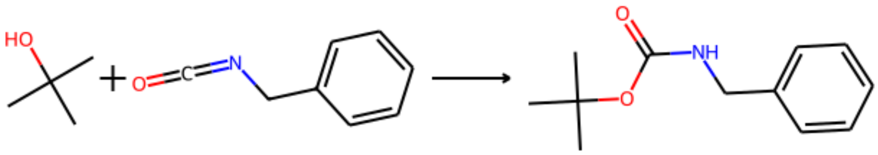

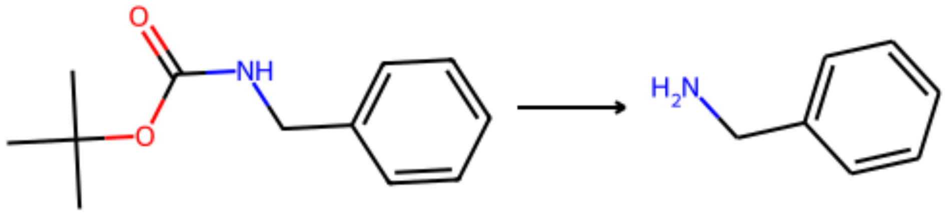

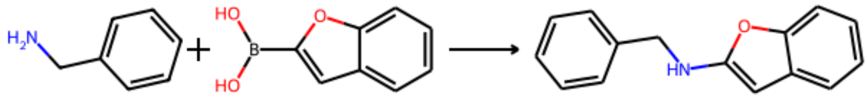

Product 214

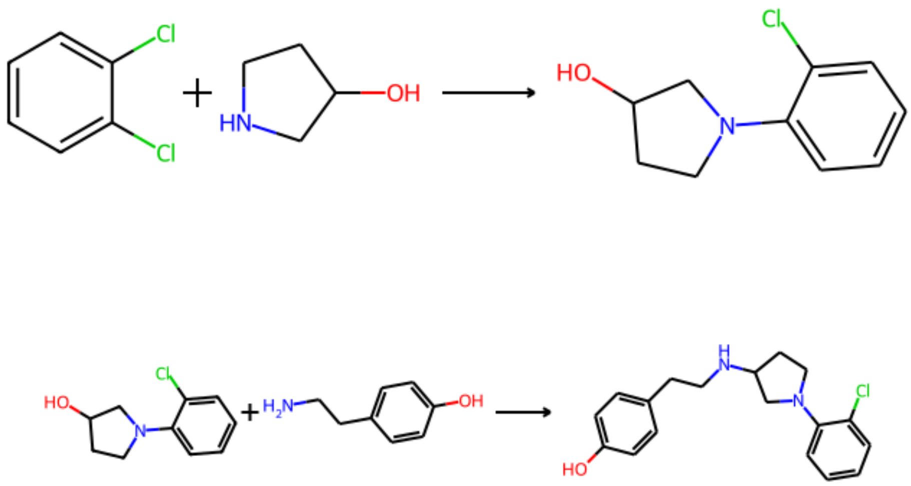

Product 215

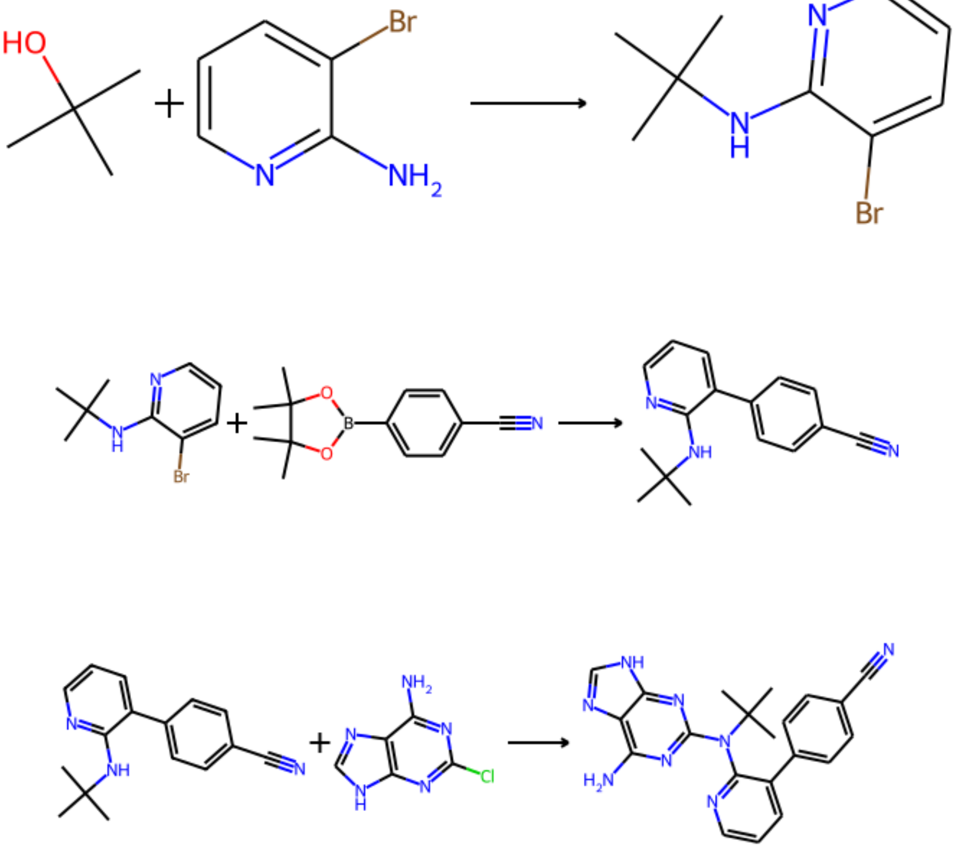

Product 216

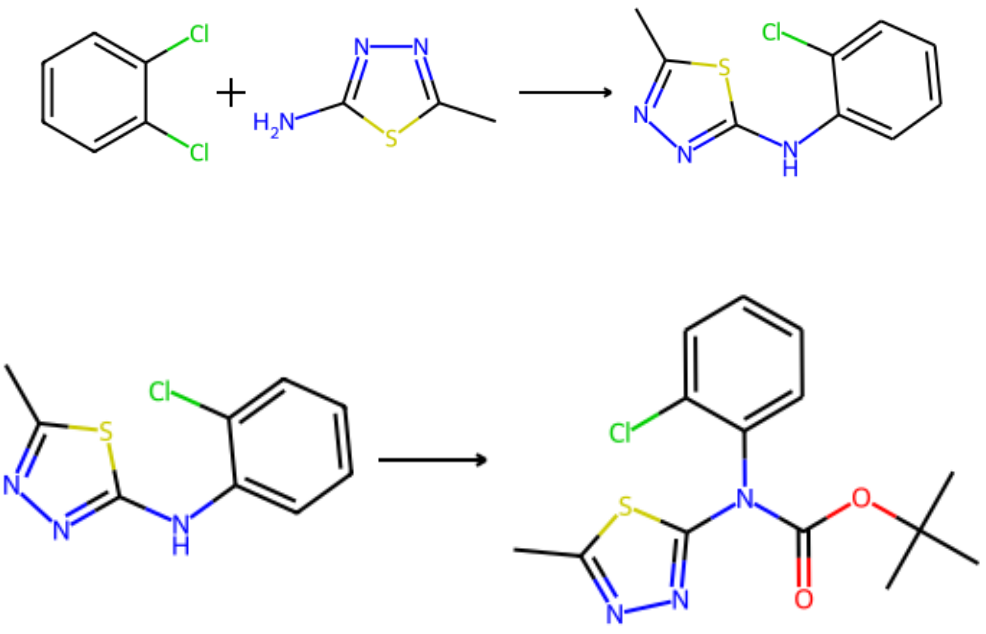

Product 217

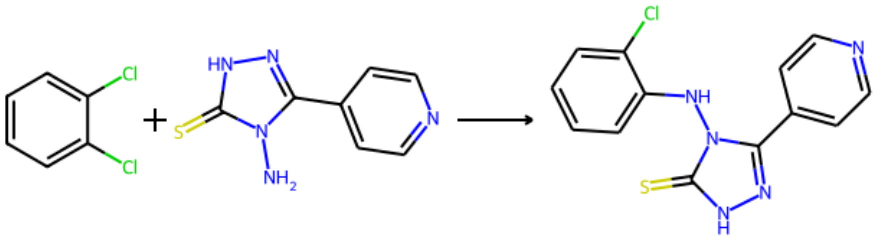

Product 218

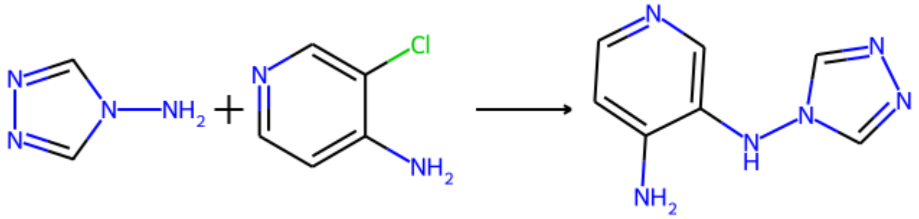

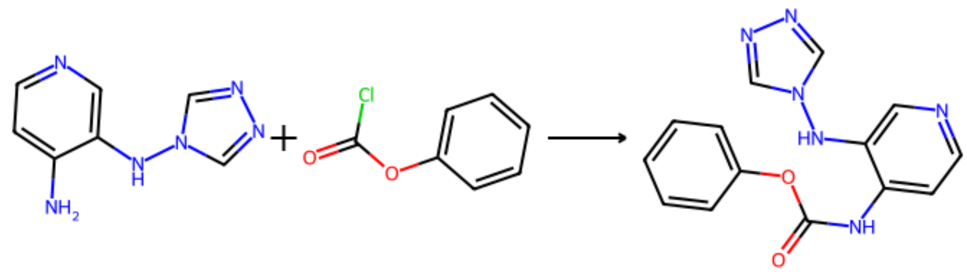

Product 219

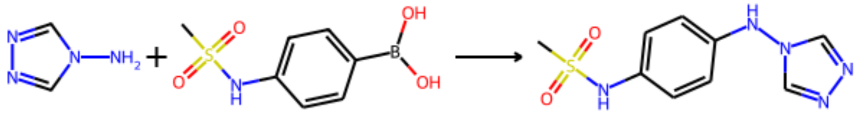

Product 220

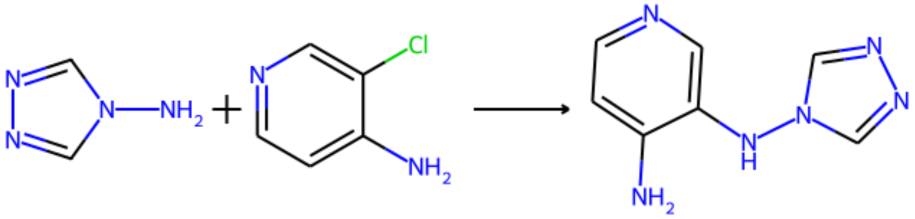

Product 221

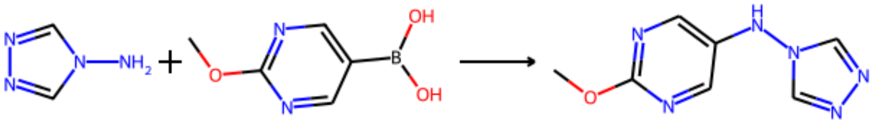

Product 222

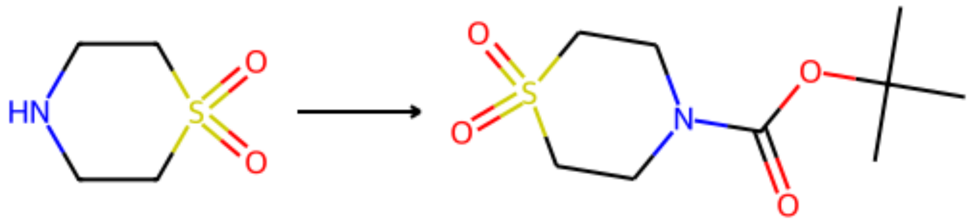

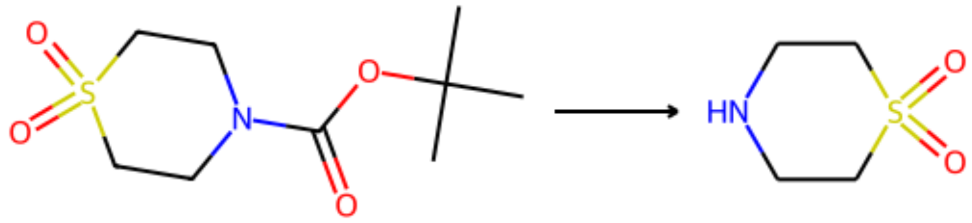

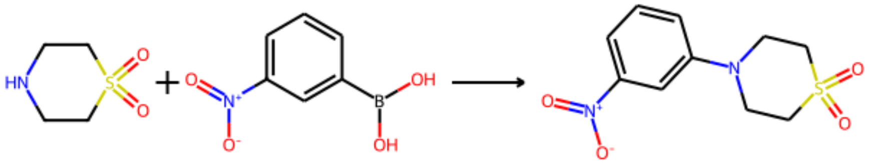

Product 223

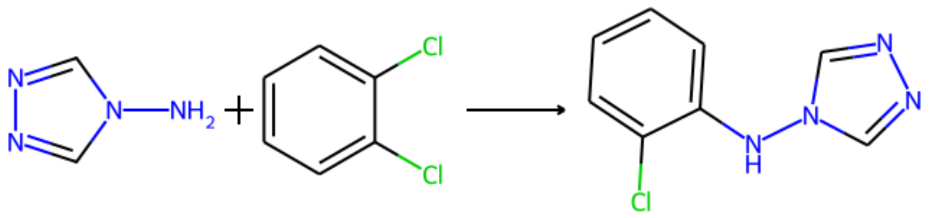

Product 224

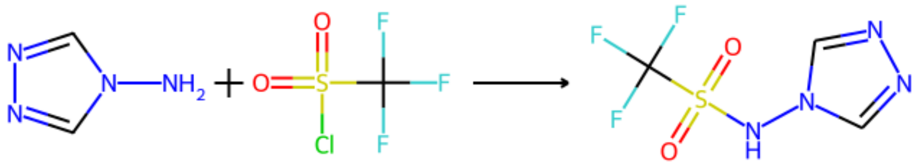

Product 225

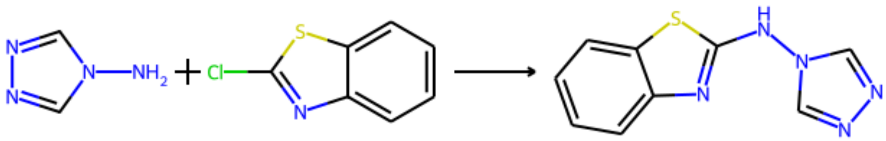

Product 226

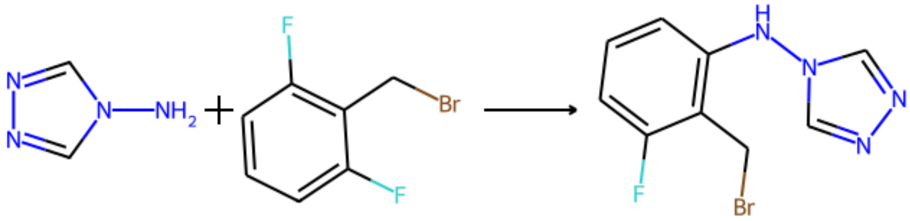

Product 227

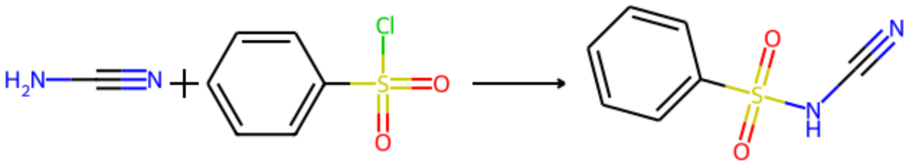

Product 228

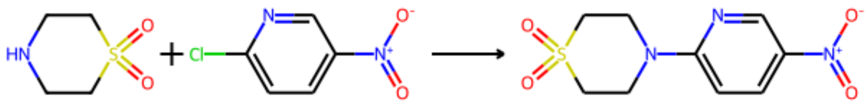

Product 229

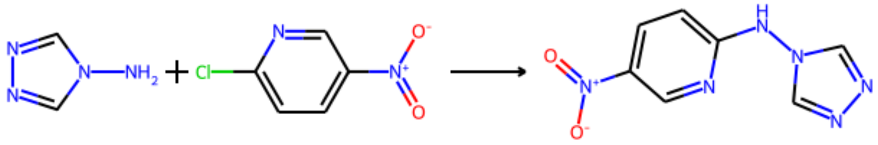

Product 230

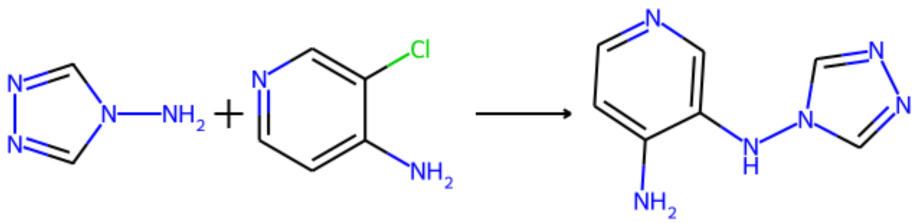

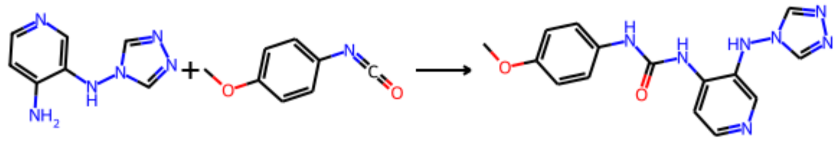

Product 231

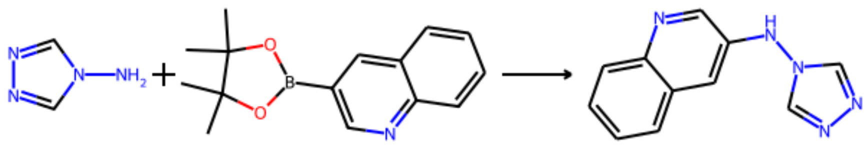

Product 232

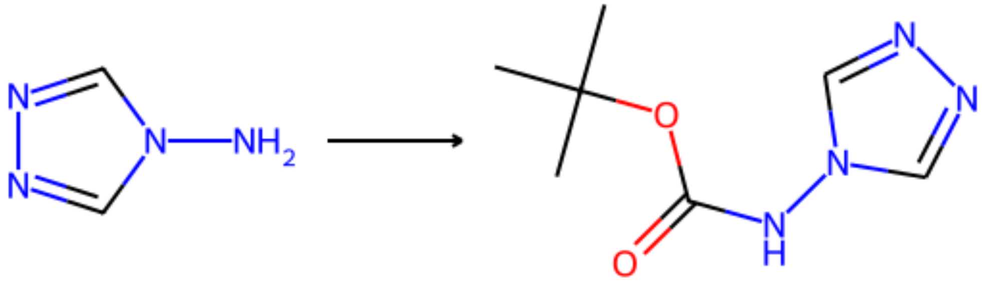

Product 233

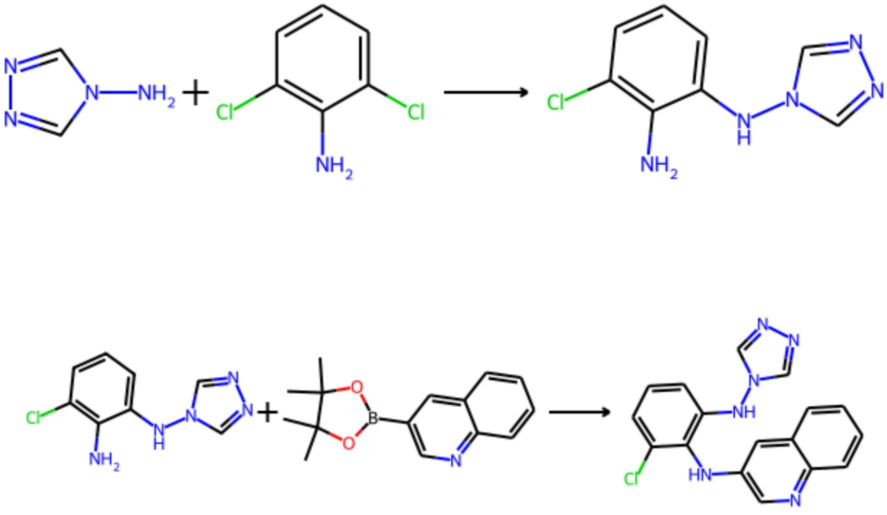

Product 234

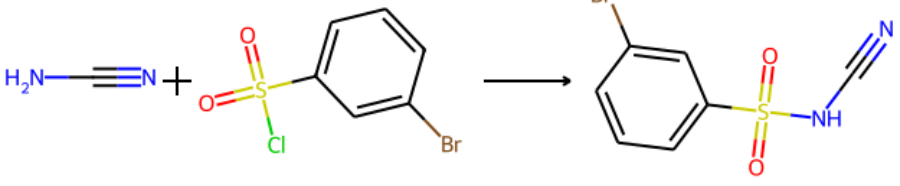

Product 235

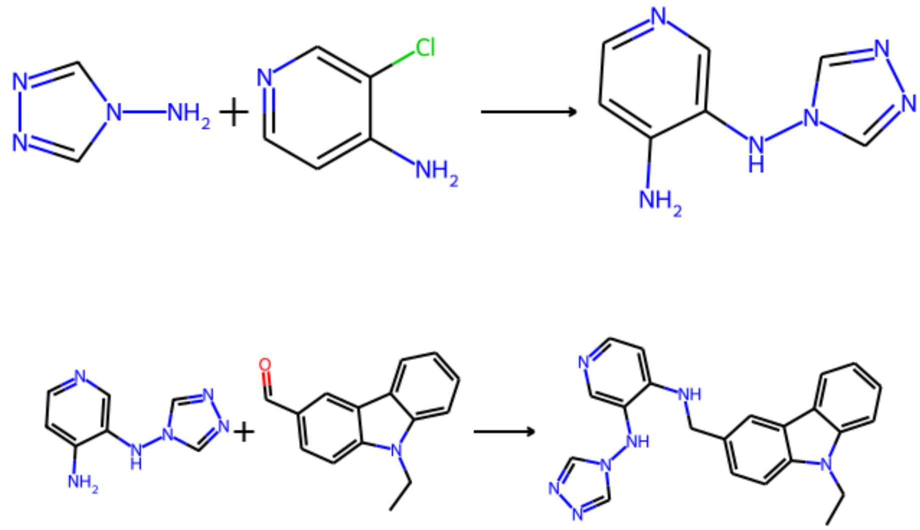

Product 236

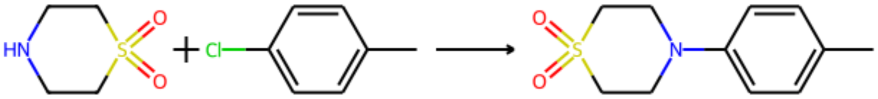

Product 237

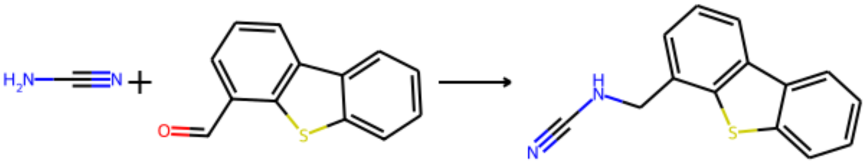

Product 238

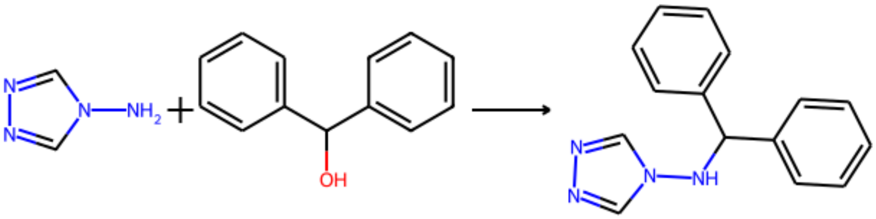

Product 239

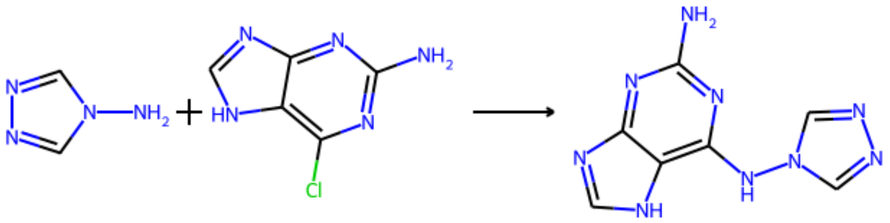

Product 240

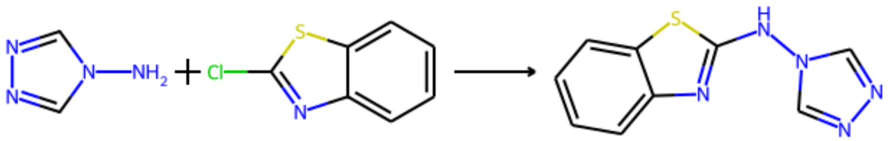

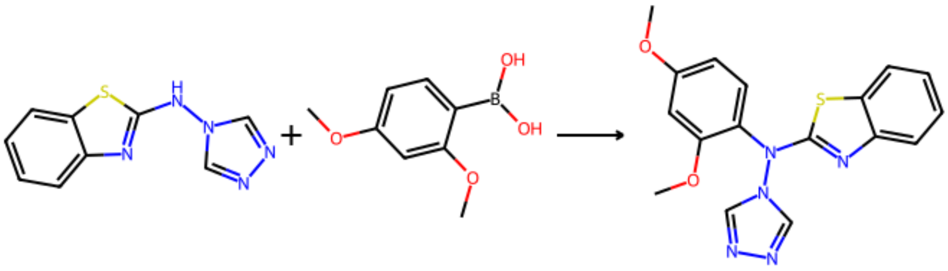

Product 241

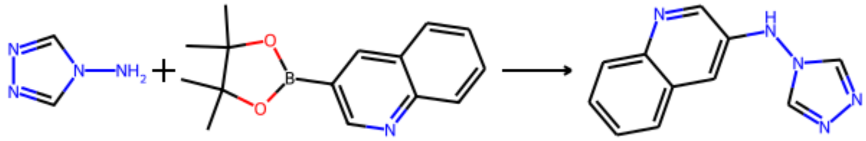

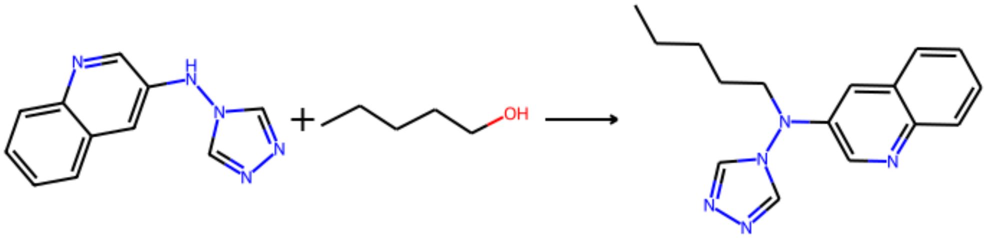

Product 242

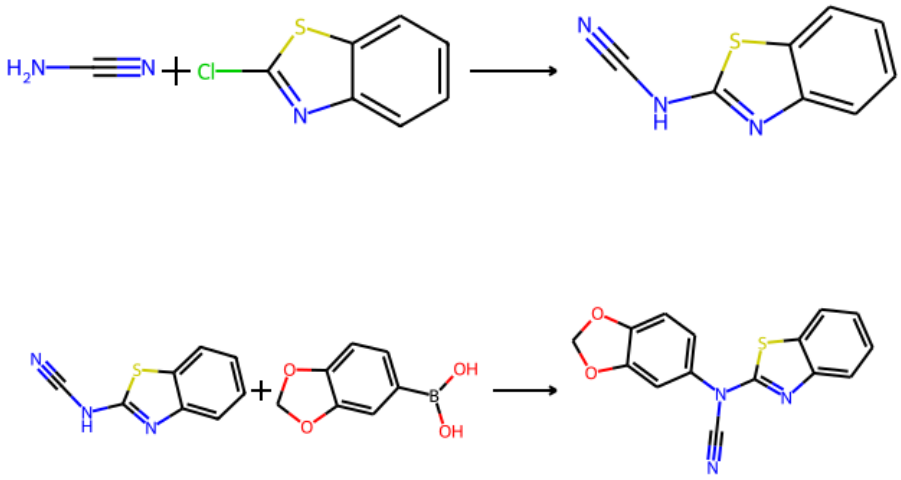

Product 243

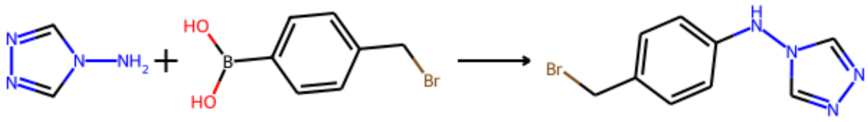

Product 244

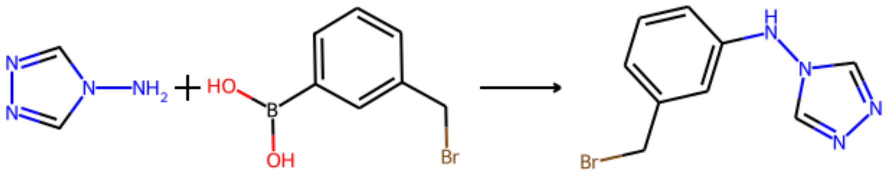

Product 245

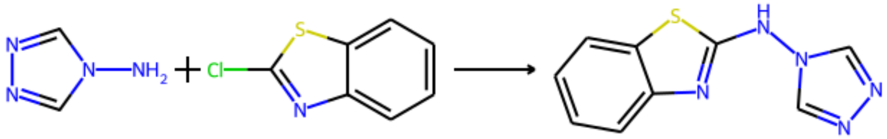

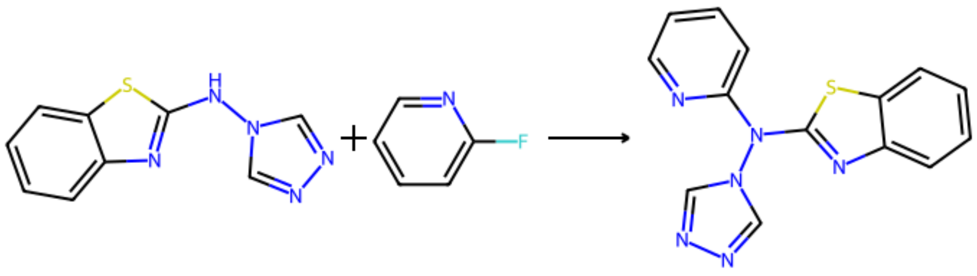

Product 246

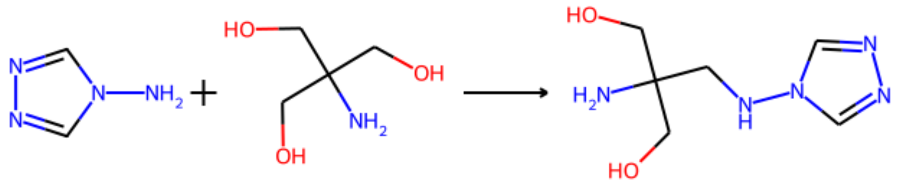

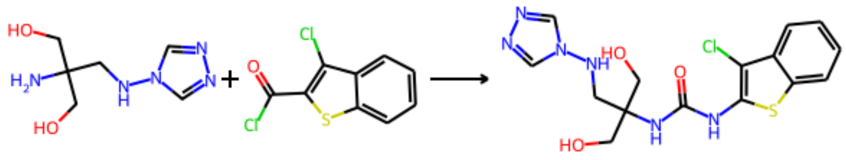

Product 247

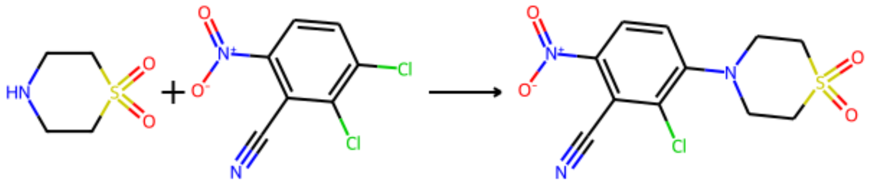

Product 248

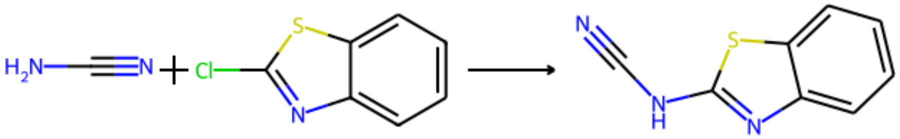

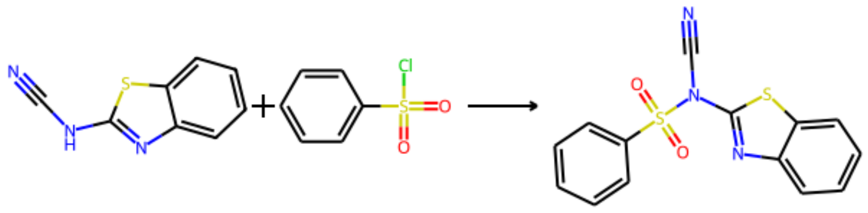

Product 249

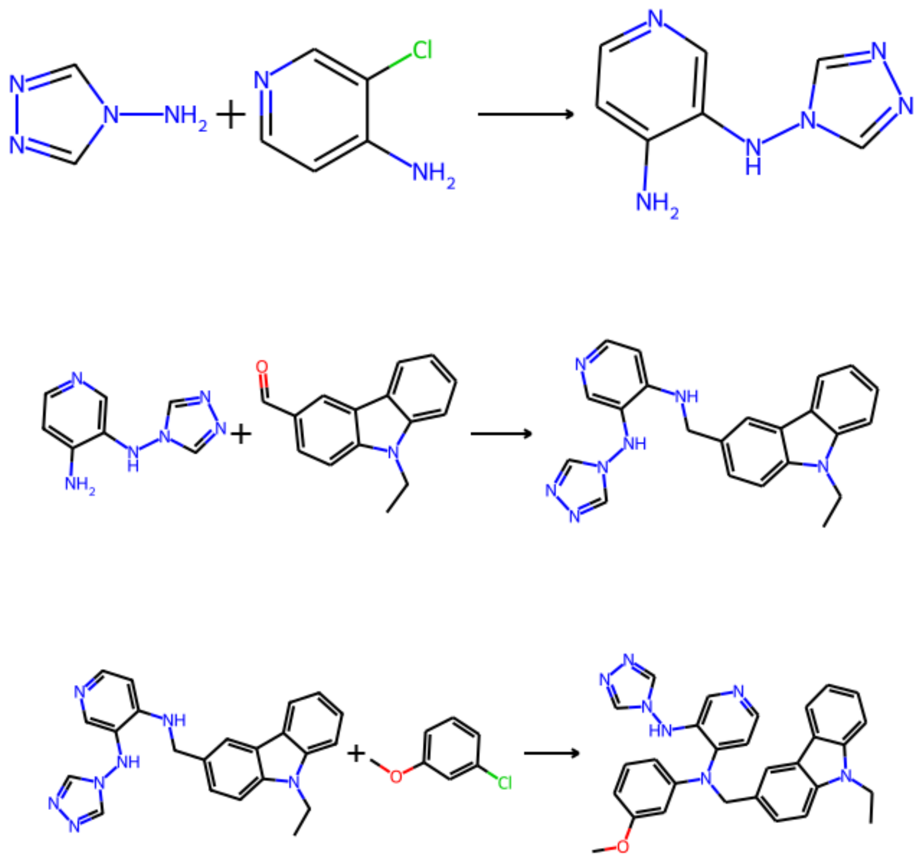

Product 250

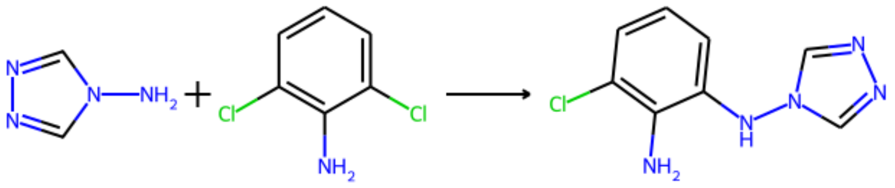

Product 251

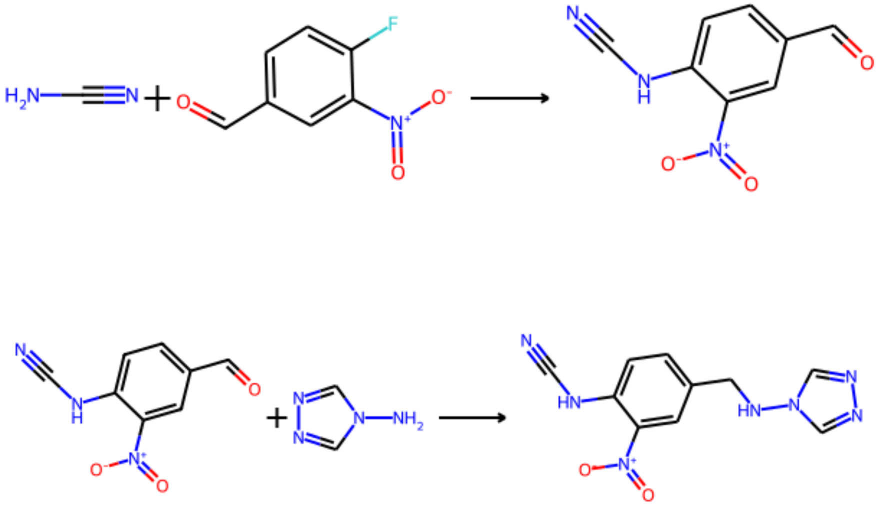

Product 252

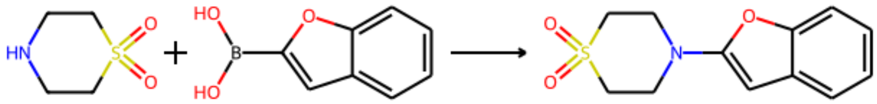

Product 253

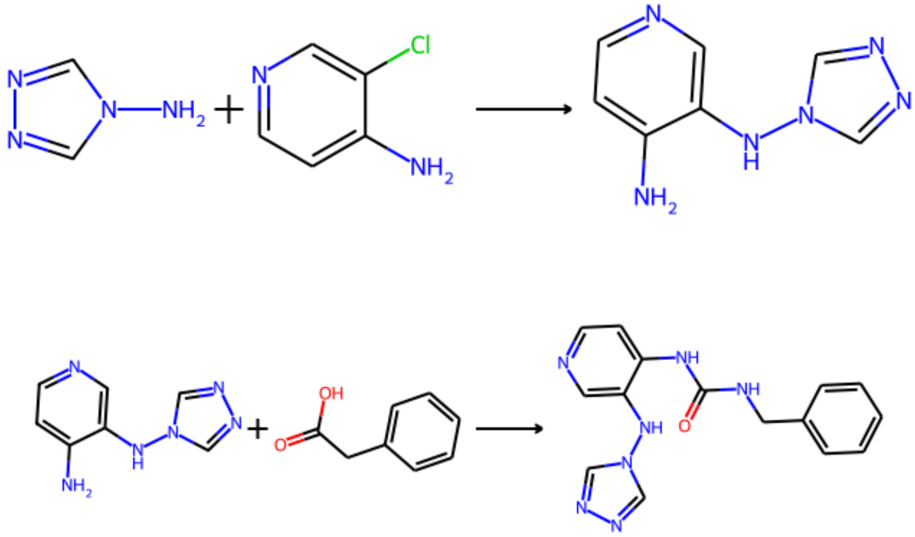

Product 254

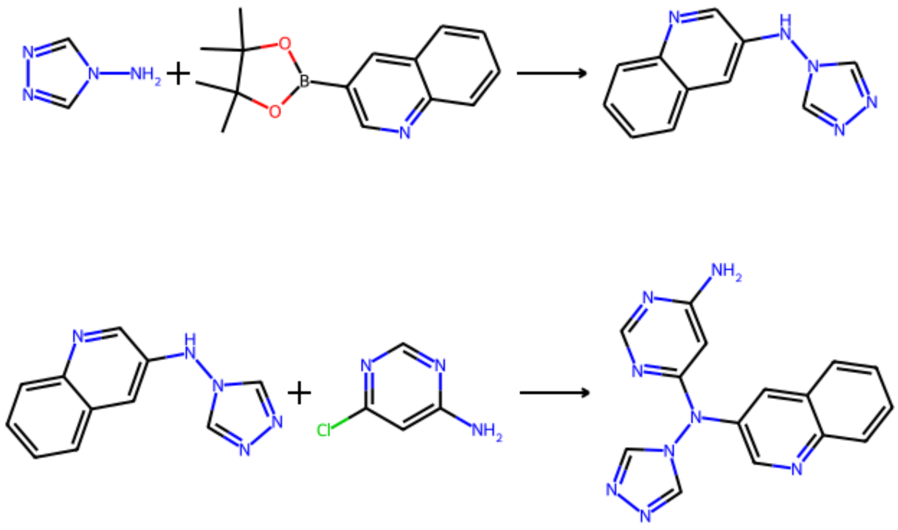

Product 255

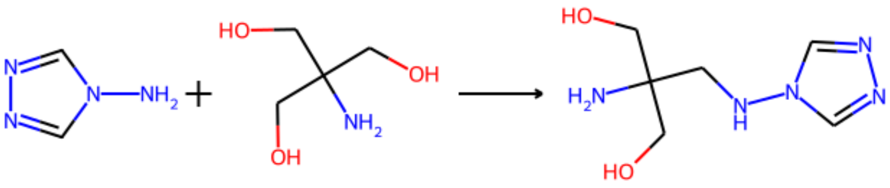

Product 256

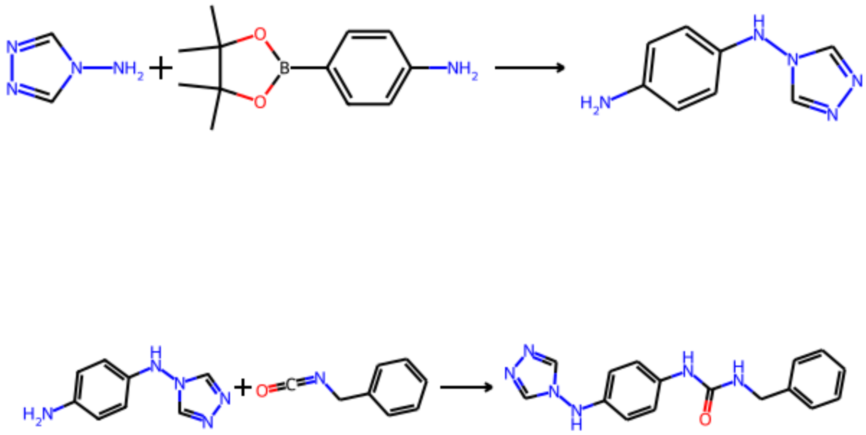

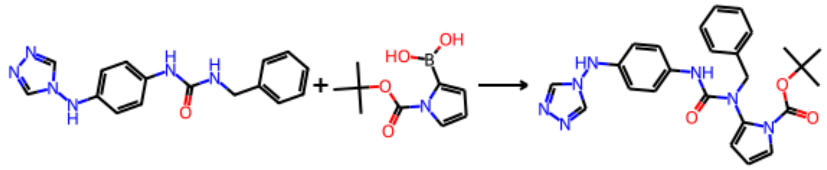

Product 257

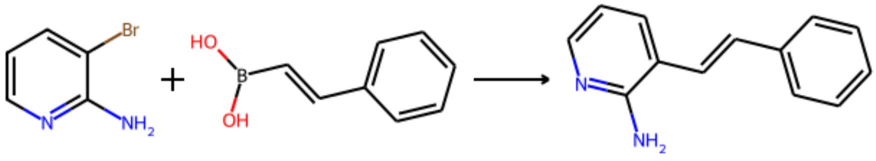

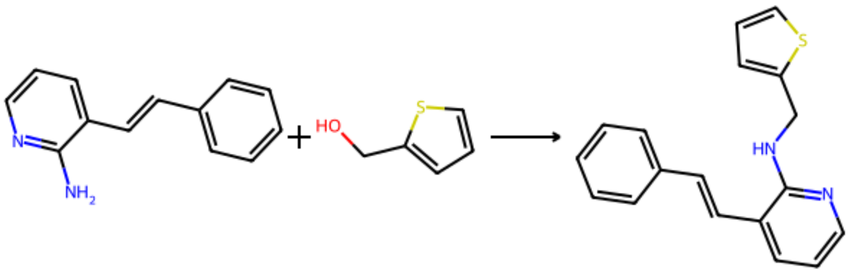

Product 258

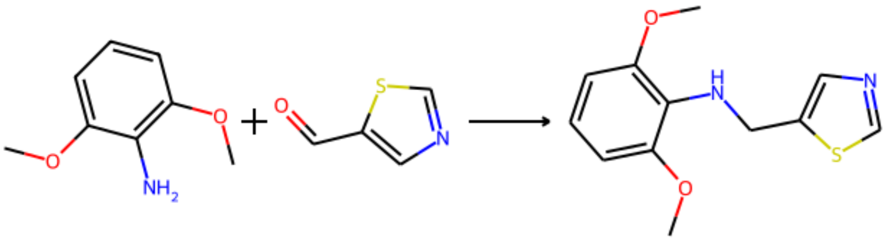

Product 259

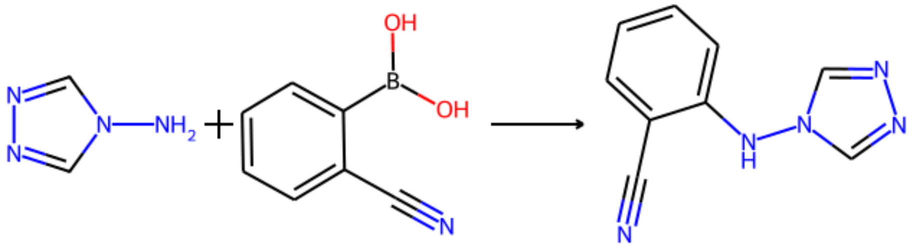

Product 260

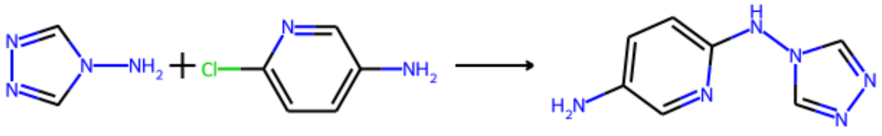

Product 261

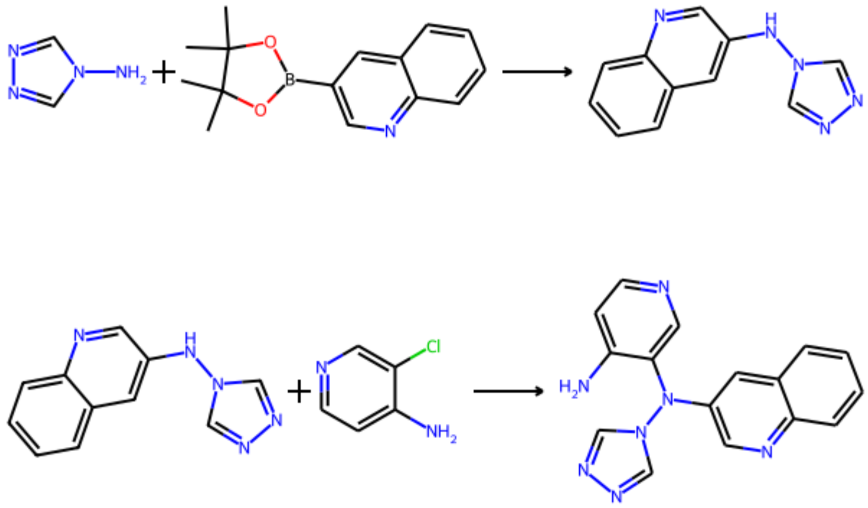

Product 262

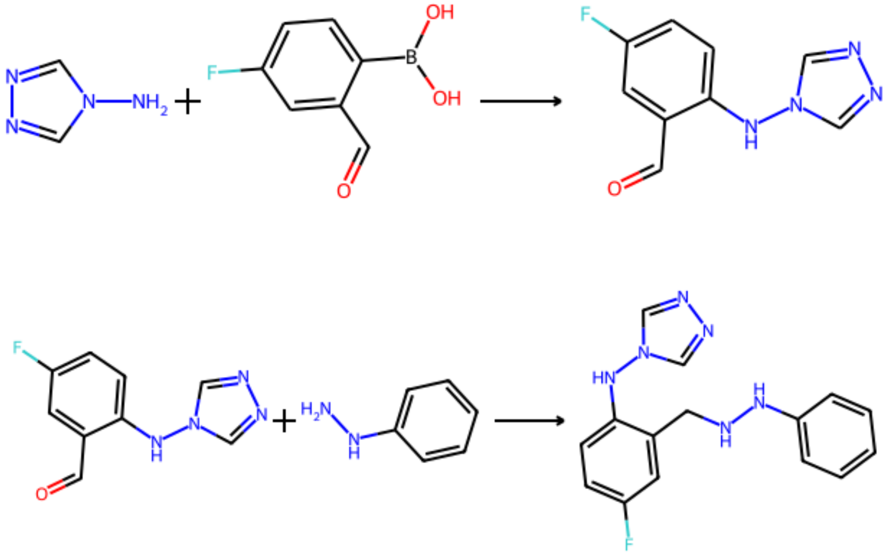

Product 263

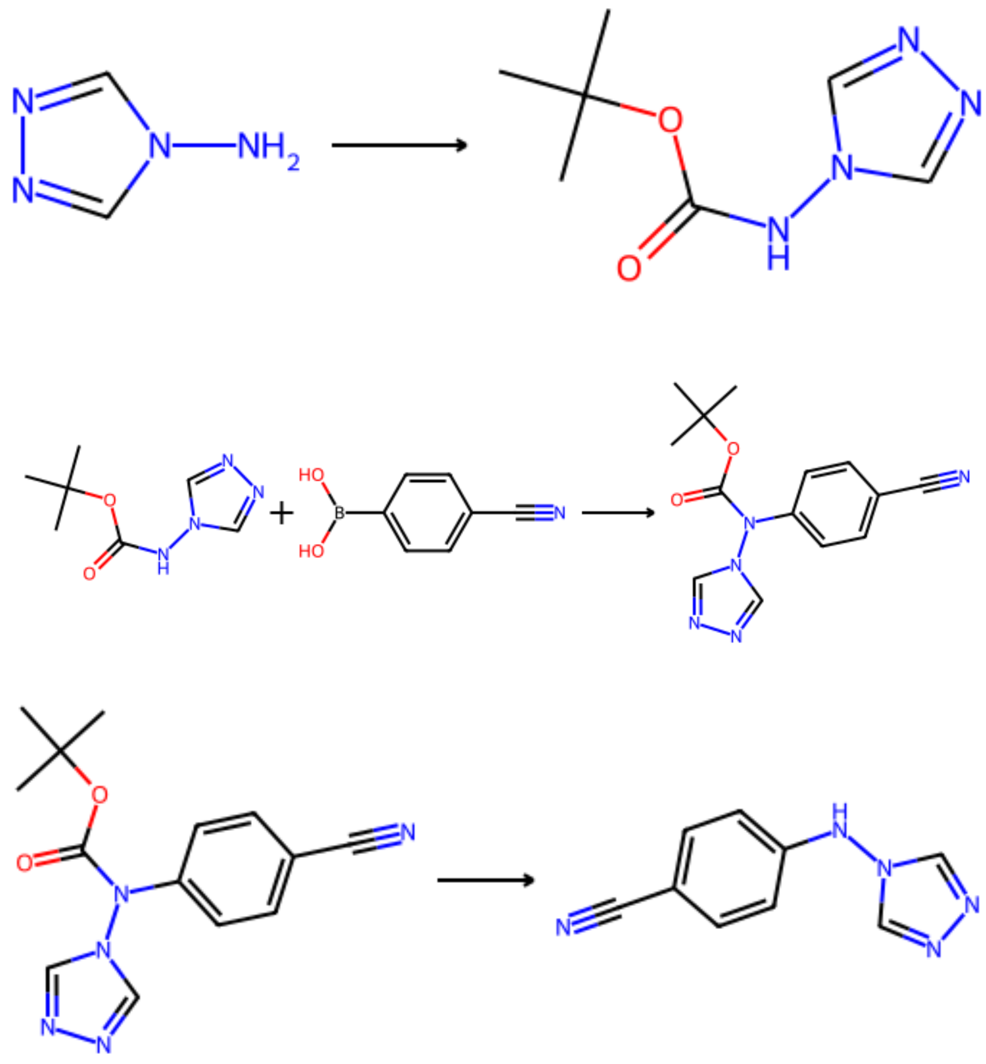

Product 264

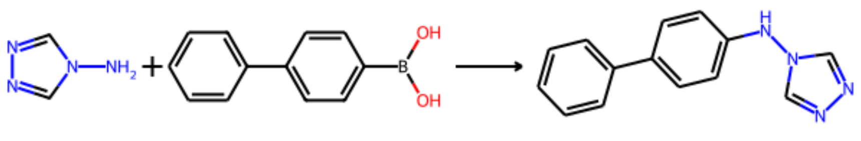

Product 265

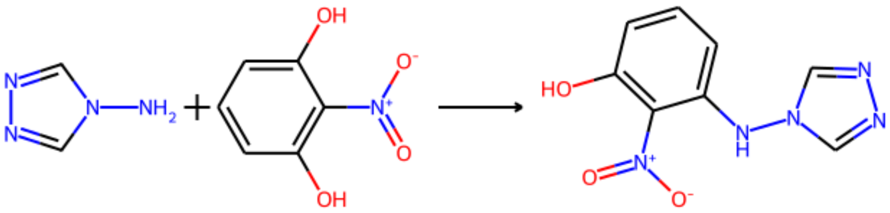

Product 266

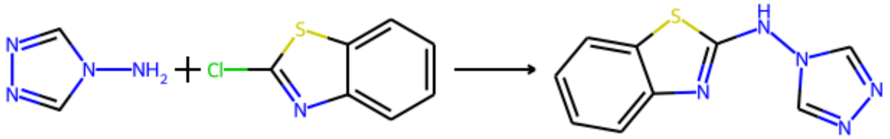

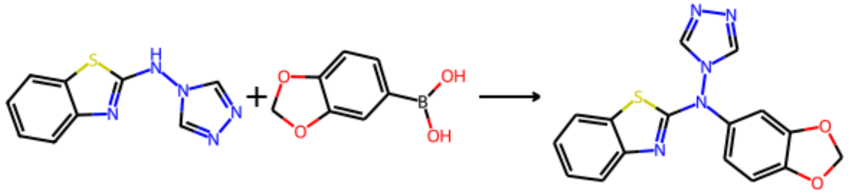

Product 267

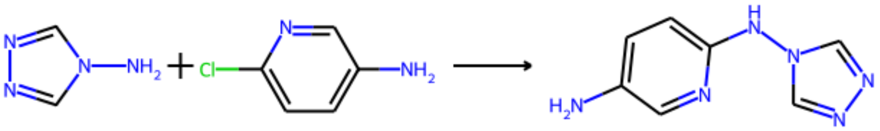

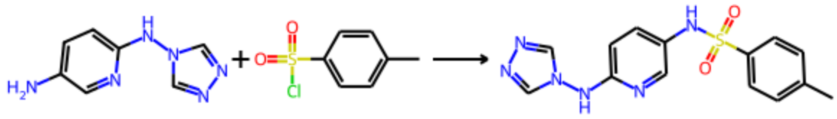

Product 268

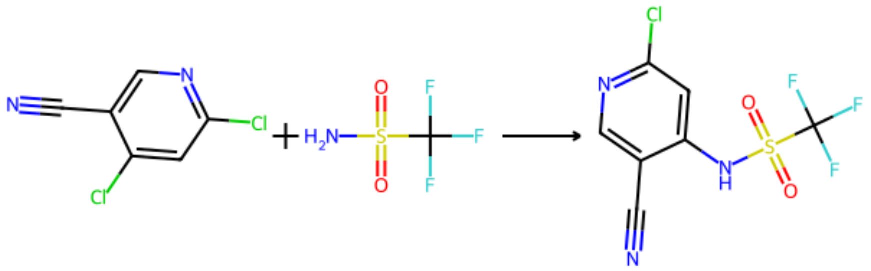

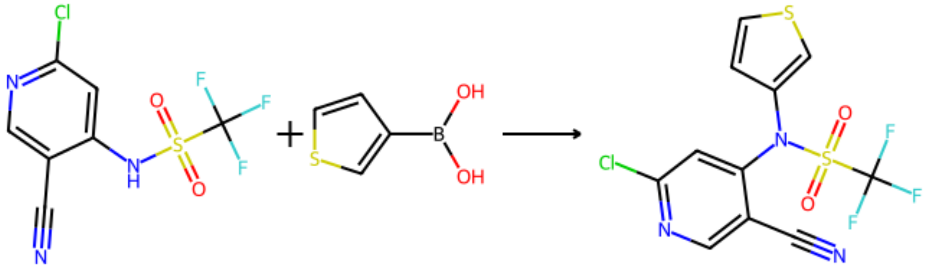

Product 269

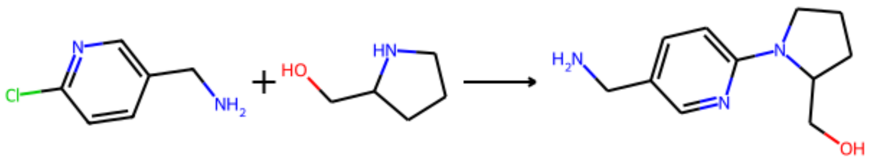

Product 270

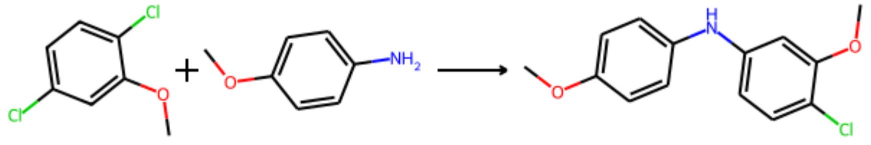

Product 271

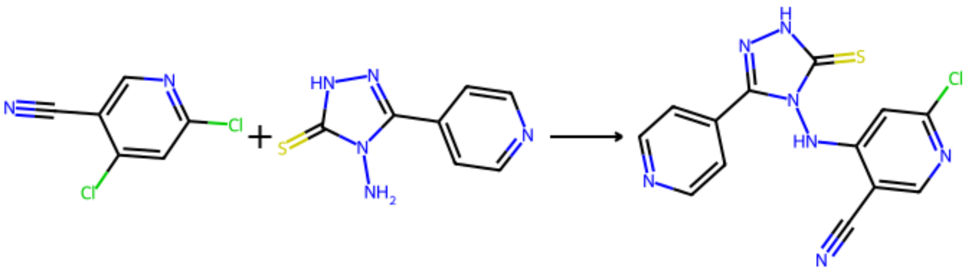

Product 272

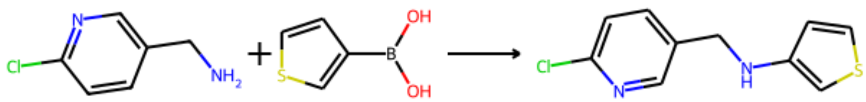

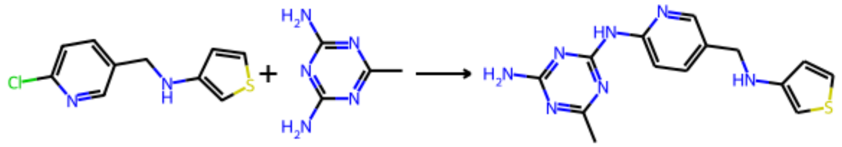

Product 273

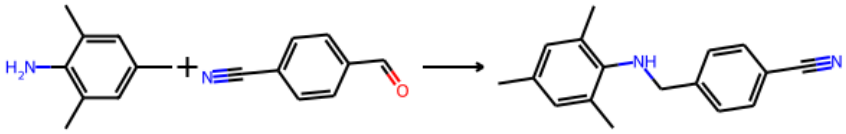

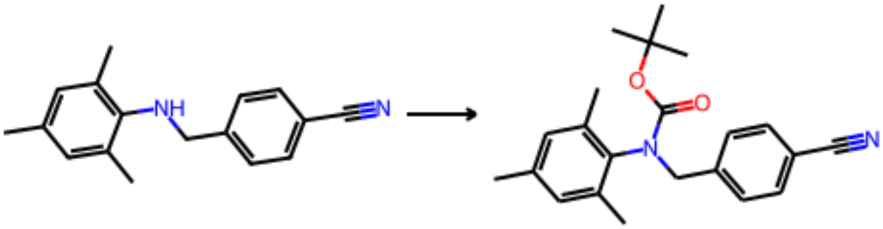

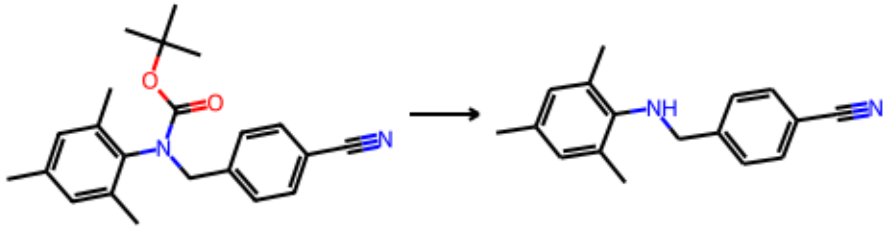

Product 274

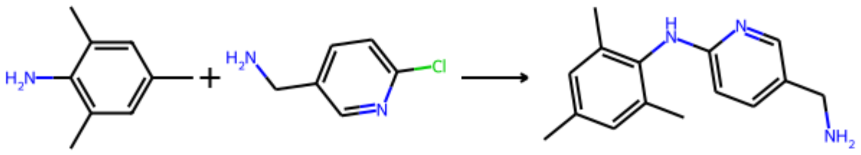

Product 275

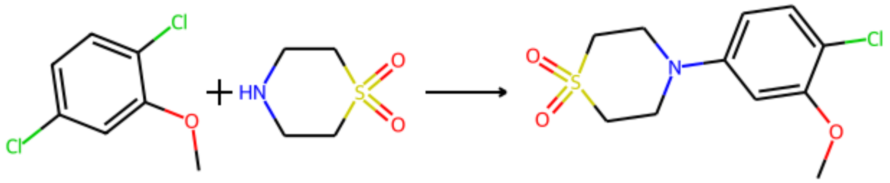

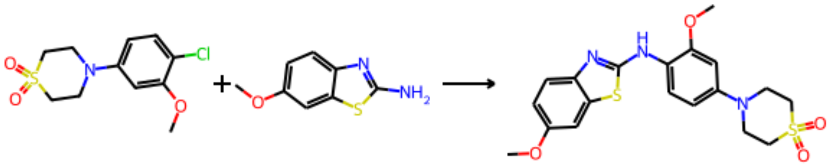

Product 276

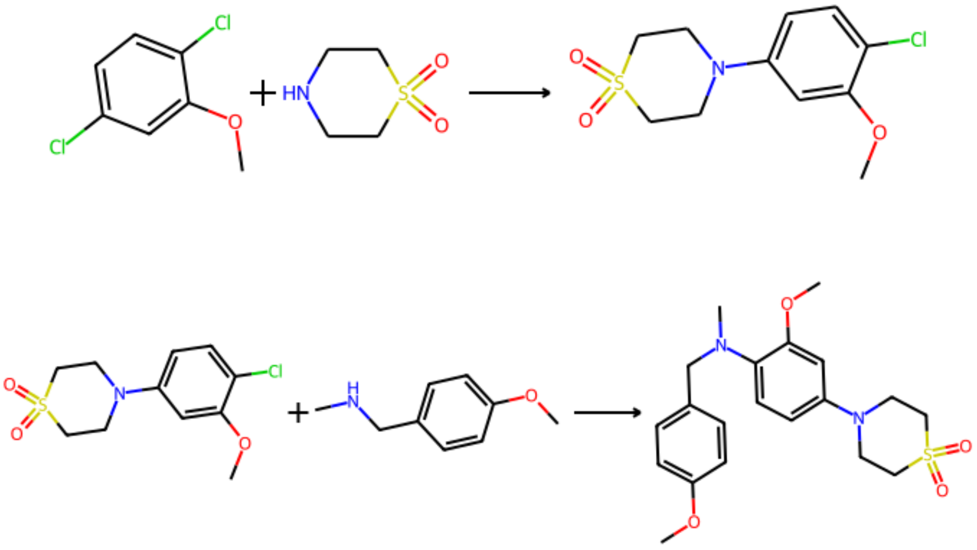

Product 277

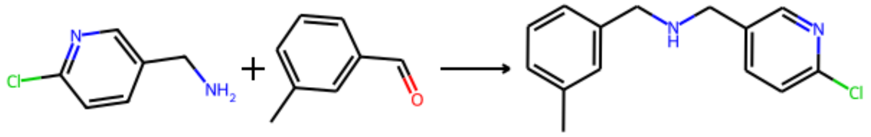

Product 278

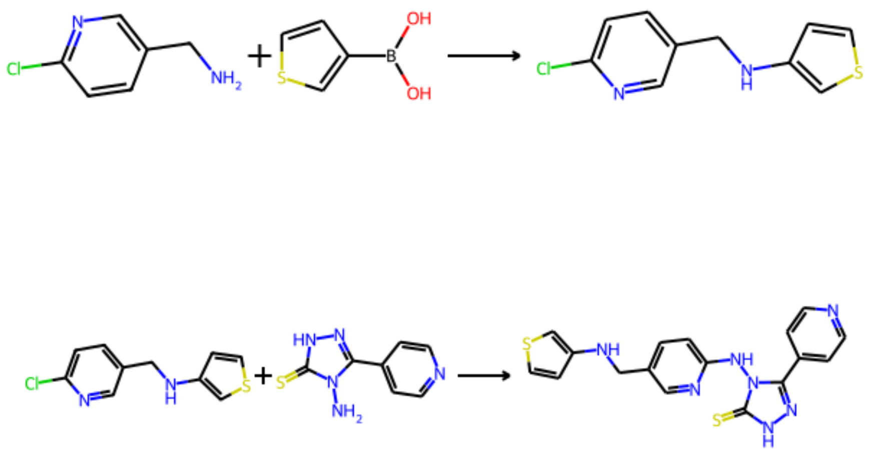

Product 279

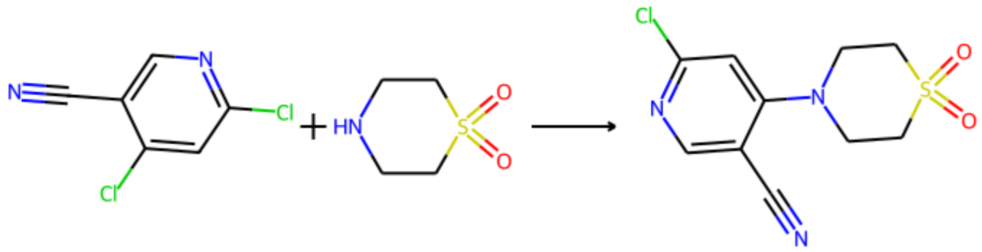

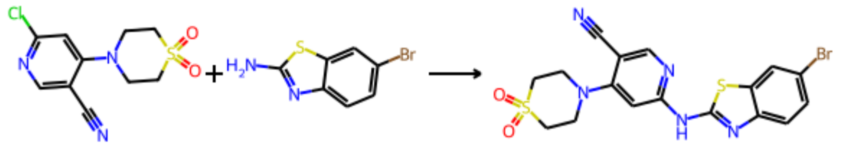

Product 280

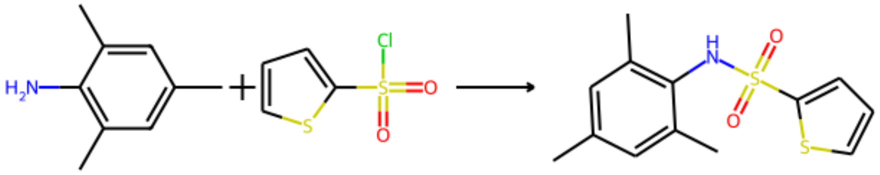

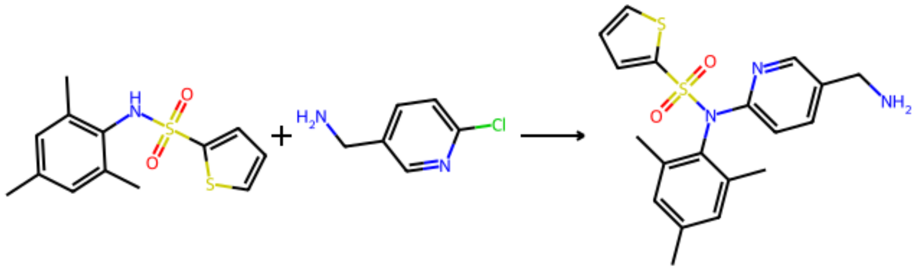

Product 281

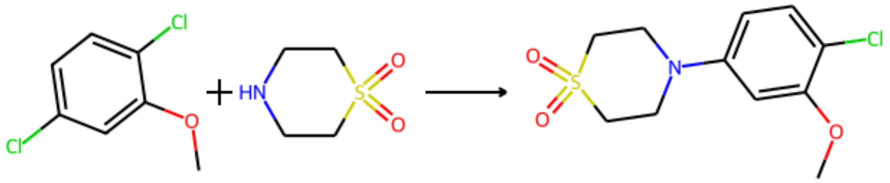

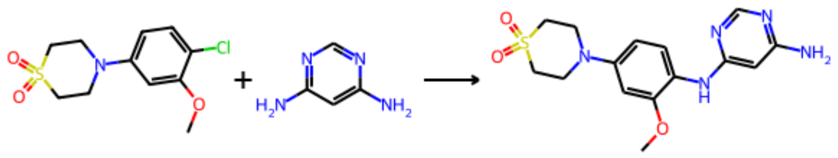

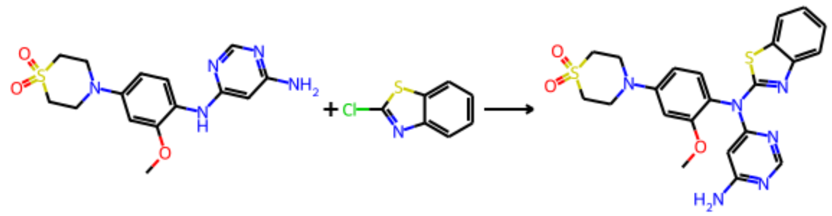

Supplement: Supplementary file 3 — oc4c01991_si_003.pdf [file oc4c01991_si_003.pdf]
